# Li vs Na: Divergent Reaction Patterns between Organolithium and Organosodium Complexes and Ligand-Catalyzed Ketone/Aldehyde Methylenation

**DOI:** 10.1021/jacs.3c01033

**Published:** 2023-03-08

**Authors:** Nathan Davison, Claire L. McMullin, Lu Zhang, Shu-Xian Hu, Paul G. Waddell, Corinne Wills, Casey Dixon, Erli Lu

**Affiliations:** † Chemistry−School of Natural and Environmental Sciences, Newcastle University, Newcastle upon Tyne NE1 7RU, U.K.; ‡ Department of Chemistry, University of Bath, Claverton Down, Bath BA2 7AY, U.K.; § School of Mathematics and Physics, University of Science and Technology Beijing, Beijing 100083, P. R. China

## Abstract

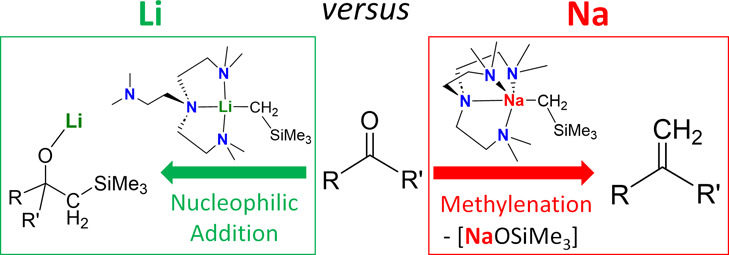

Organosodium chemistry is underdeveloped compared with organolithium chemistry, and all the reported organosodium complexes exhibit similar, if not identical, reactivity patterns to their lithium counterparts. Herein, we report a rare organosodium monomeric complex, namely, [Na(CH_2_SiMe_3_)(Me_6_Tren)] (**1**-Na) (Me_6_Tren: tris[2-(dimethylamino)ethyl]amine) stabilized by a *tetra*-dentate neutral amine ligand Me_6_Tren. Employing organo-carbonyl substrates (ketones, aldehydes, amides, ester), we demonstrated that **1**-Na features distinct reactivity patterns compared with its lithium counterpart, [Li(CH_2_SiMe_3_)(Me_6_Tren)] (**1**-Li). Based on this knowledge, we further developed a ligand-catalysis strategy to conduct ketone/aldehyde methylenations, using [NaCH_2_SiMe_3_]_∞_ as the CH_2_ feedstock, replacing the widely used but hazardous/expensive C=O methylenation methods, such as Wittig, Tebbe, Julia/Julia-Kocieński, Peterson, and so on.

## Introduction

Organosodium complexes were first reported in 1858 by James Alfred Wanklyn, describing a reaction between ethyl iodide and sodium metal.^1,2^ In the following centuries, despite discrete reports of their synthesis and structures,^3−21^ as well as their applications in organic,^2,22−26^ inorganic,^27^ and polymer^28−33^ syntheses, organosodium chemistry is largely overshadowed by the dominating organolithium chemistry,^34−40^ which was first reported in 1917,^34^ nearly 60 years later than Wanklyn’s report in 1858. The underdeveloped status of organosodium chemistry was widely attributed to the highly reactive nature and the corresponding low thermostability of these complexes.^2^ The presumed low thermostability, however, is a moot point in our understanding. For example, a starting material in this work, [NaCH_2_SiMe_3_]_∞_, was found to be stable even at elevated temperature (see the Supporting Information for details).

Over 160 years after Wanklyn’s inaugural report,^1^ the beginning of the 2020s witnesses the renaissance of organosodium chemistry. This latest trend is largely driven by the community’s efforts to seek more sustainable and economically efficient alternatives to the ubiquitous organolithium reagents. Sodium is much more abundant than lithium in the Earth’s crust (Na 2.36% vs Li 0.002%^41^) and also more environmentally benign.^42^ Moreover, driven by the fast rising demand of lithium-ion batteries and the unstable global geopolitical environment, the lithium price (battery-grade LiOH) has increased by 150% since the beginning of 2022 to $25,000 per metric ton, and the price is projected to further increase to $36,000 in 2023.^43^ Partially motivated by these factors, since the late 2010s, synthetic chemistry community has been re-visiting organosodium complexes as sustainable and economical replacement for the widely applied organolithium reagents. In 2019, Asako et al. reported that in situ formed organosodium complexes (from halogenated arenes/alkanes reacting with Na metal) served as transmetallation reagents in Pd-catalyzed cross-coupling reactions.^44,45^ In a 2021 follow-up study, the same group reported a facile access to organosodium complexes via halogen-sodium exchange reactions.^46,47^ In 2022, based on their previous work,^25^ Knochel and co-workers reported the preparation of benzylic sodium complexes in a continuous flow setup.^48^ In this case, the continuous flow technique was adopted as a countermeasure against the presumed low stability of the organosodium complexes.^25,26,48^

Nevertheless, a centuries-old paradigm in organo-alkali metal chemistry is that they may differ in activity (fast or slow reaction) and even selectivity (e.g., different deprotonation sites), yet organolithium and organosodium complexes follow the same reaction patterns. For example, they may act as Brønsted-Lowry bases, nucleophiles, transmetallation reagents (the organolithium version is Murahashi coupling^49^) or undergo halogen-metal exchange. However, for the equivalent set of substrates, the organolithium and organosodium complexes have always behaved in the same pattern. Breaking the paradigm, i.e., developing strategies to tune organolithium and organosodium complexes for diversified reaction patterns, could unlock an immense unexplored chemical space and provide new sustainable and readily available tools for synthetic chemists. However, to the best of our knowledge, such a strategy is still unknown. During the preparation of this manuscript, Hevia and co-workers reported that lower aggregates of NaCH_2_SiMe_3_, which were stabilized by Lewis bases *N*,*N*,*N*′,*N*″,N″*-*pentamethyldiethylenetriamine (PMDTA) and tris[2-(dimethylamino)ethyl]amine (Me_6_Tren), can facilitate benzylic C^sp3^–H bond metalation and subsequent aroylation with Weinreb amides, where the corresponding organolithium complex (LiCH_2_SiMe_3_ + PMDTA) failed to deliver such reactivity.^50^ It is interesting for us to note that the benzylic C^sp3^–H bond metalation is concentration-dependent: while we reported in 2022 that the combination of LiCH_2_SiMe_3_ + Me_6_Tren can deliver such metalation with neat toluene,^51^ Hevia and co-workers observed that LiCH_2_SiMe_3_ + Me_6_Tren/PMDTA cannot activate toluene in hexane solution.^50^

Herein, we report that a rare organosodium monomeric complex, [Na(CH_2_SiMe_3_)(Me_6_Tren)] (**1**-Na), which was simultaneously reported by Hevia and co-workers,^50^ exhibits distinct reactivity patterns with organic carbonyl substrates, compared with our previously reported organolithium counterpart, i.e., [Li(CH_2_SiMe_3_)(Me_6_Tren)] (**1**-Li):^51^**1**-Na converts a C=O bond in ketone/aldehyde into a C=CH_2_ bond, i.e., conducts ketone/aldehyde methylenation. In comparison, **1**-Li reacts with such substrates following the conventional and predictable nucleophilic addition route. Based on these observations, we designed and realized ligand-catalyzed organosodium-mediated ketone/aldehyde methylenations, providing a sustainable and easy-to-operate alternative to the classic methylenation methods (Wittig, Julia, Tebbe, Peterson, etc.). Moreover, by treating **1**-Na with amide and ester, we demonstrate the versatility of its reactivity profile, where the N- or O-substitution was found altering the reaction outcomes profoundly. These findings are elaborated in the following sections.

## Results and Discussion

### Synthesis and Characterization of the Organosodium Monomeric Complex (**1**-Na)

**Scheme 1:**
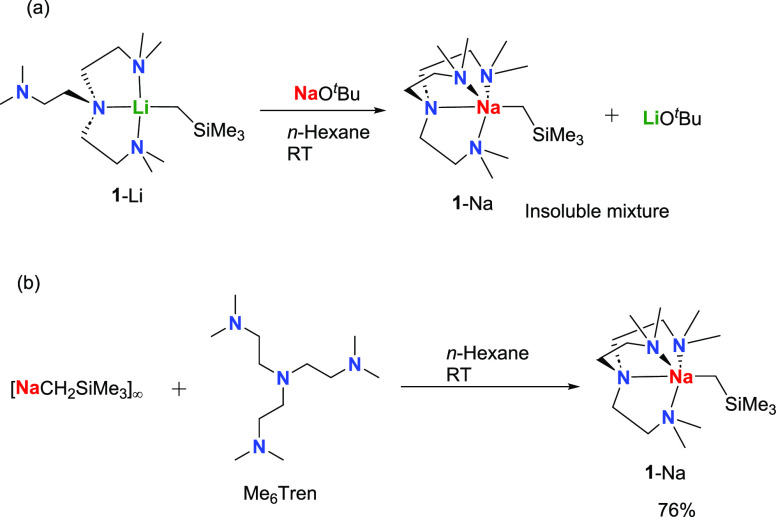
Two Synthetic Routes of **1**-Na (a) Metal exchange between **1**-Li and NaO^*t*^Bu produces a mixture of **1**-Na and LiO^*t*^Bu. (b) Coordination between [NaCH_2_SiMe_3_]_∞_ and Me_6_Tren produces clean **1**-Na.

We first treated **1**-Li with one equivalent of sodium *tert*-butoxide (NaO^*t*^Bu), aimed at utilizing the NaO^*t*^Bu to activate the monomeric Li–C bond in **1**-Li and isolating a Li–Na heterobimetallic complex. Activation of polar organometallic reagents with alkali metal alkoxides is a well-documented methodology, dating back to the 1960s when the widely used LIC-KOR superbases (Lochmann–Schlosser bases) were discovered by Lochmann^52^ and Schlosser.^53^ The interest in this field has continued,^54^ and the field was reviewed by Lochmann^55^ and more recently by Mulvey and co-workers,^56^ as well as by Bole and Hevia.^57^ However, in our case, instead of the expected Li–Na heterobimetallic complex, we isolated **1**-Na, along with LiO^*t*^Bu (Scheme 1a). Here, a Li–Na interchange occurred.^55^ Similar solubilities of **1**-Na and LiO^*t*^Bu hindered the effort to isolate these two complexes. Nevertheless, gratifyingly, pure **1**-Na was prepared from an alternative route, i.e., treating [NaCH_2_SiMe_3_]_∞_^58^ with the Me_6_Tren ligand, in 76% crystalline yield (Scheme 1b).

**Figure 1:**
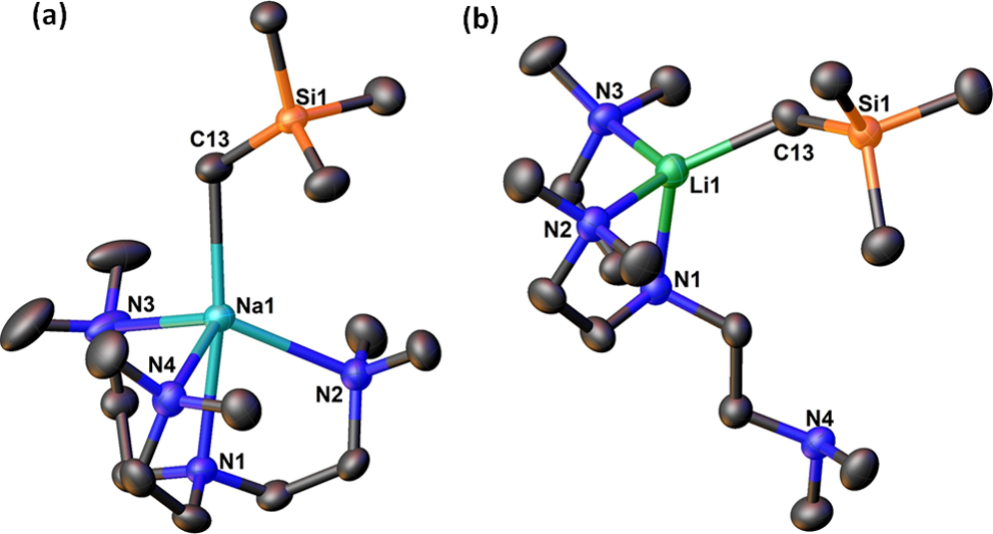
SCXRD structures of (a) **1**-Na and (b) **1**-Li.^51^ Solvent molecules in lattice, minor disorder components, and H atoms are omitted for clarity. Representative bond lengths in comparisons between **1**-Na and **1**-Li (Å): M–C13 2.5054(14) (Na), 2.122(5) (Li); M–N1 2.6137(11) (Na), 2.177(4) (Li); M–N2 2.5232(12) (Na), 2.167(4) (Li); M–N3 2.5588(12) (Na), 2.189(4) (Li); M–N4 2.5484(13) (Na), N/A (Li).

Colorless crystals of **1**-Na suitable for single-crystal X-ray diffraction (SCXRD) analysis were obtained by storing its *n*-hexane solution at −35 °C overnight. The solid-state molecular structure of **1**-Na is shown in Figure 1a and is very similar to the structure very recently reported by Hevia and co-workers:^50^ comparing the crystallographic cell parameters confirms that they are the same crystals. For comparison, the **1**-Li structure from our previous report in 2022^51^ is also displayed (Figure 1b). An obvious difference between the two structures is, in **1**-Na, all the three sidearms of the Me_6_Tren ligand coordinate with the Na^+^ center, while in **1**-Li, only two of them coordinate. This is due to the larger ionic radius of Na^+^ compared with Li^+^. The Na–C and Na–N bond lengths in **1**-Na are 0.4–0.5 Å longer than the corresponding Li–C and Li–N bond lengths in **1**-Li. The proton nuclear magnetic resonance (^1^H NMR) spectrum of **1**-Na in C_6_D_6_ at 298 K exhibits the typical *C*_3_*v* symmetry,^59^ which is consistent with its solid-state structure. Furthermore, we conducted ^1^H diffusion ordered spectroscopy (^1^H DOSY) studies of **1**-Na and its polymeric parent complex [NaCH_2_SiMe_3_]_∞_. **1**-Na is stable in *d*_12_-cyclohexane (C_6_D_12_) at room temperature for at least 3 h with less than 30% decomposition (see SI Figure S9), allowing us to study its ^1^H DOSY spectrum. **1**-Na’s molecular weight in C_6_D_12_ was deduced as 333.12 (assuming expanded disc shape) or 355.57 (assuming dissipated sphere and ellipsoid shape), both are close to the actual molecular weight as a monomer (340.61) determined by SCXRD study, confirming that the solid-state monomeric structure of **1**-Na retains in solution. For comparison, the ^1^H DOSY study of [NaCH_2_SiMe_3_]_∞_ in *d*_6_-benzene allows us to deduce a molecular weight of 416.87 (assuming dissipated sphere and ellipsoid), indicating a tetrameric structure in solution (4 × 110.20 = 448.80). Hence, upon dissolving, the polymeric infinite structure of [NaCH_2_SiMe_3_]_∞_ breaks into tetramer [NaCH_2_SiMe_3_]_4._ The Na–C*H*_2_^1^H NMR signal of **1**-Na appears at −1.42 ppm, which is comparable with the Li–C*H*_2_ signal of **1**-Li (−1.61 ppm).^51^ Natural localized molecular orbitals (NLMO) and topological analyses of the Li–C and Na–C bonds in **1**-Li/Na reveal a similar bonding scenario: both are highly ionic with the electron density mostly residing on the anionic carbon center (see SI for details).

### Stoichiometric Reactions between the Li/Na Monomeric Alkyl Complexes [M(CH_2_SiMe_3_)(Me_6_Tren)] (**1**-Li, **1**-Na) and Ketones/Aldehydes: Nucleophilic Addition, C–H Deprotonation, or C=O Olefination?

**Scheme 2:**
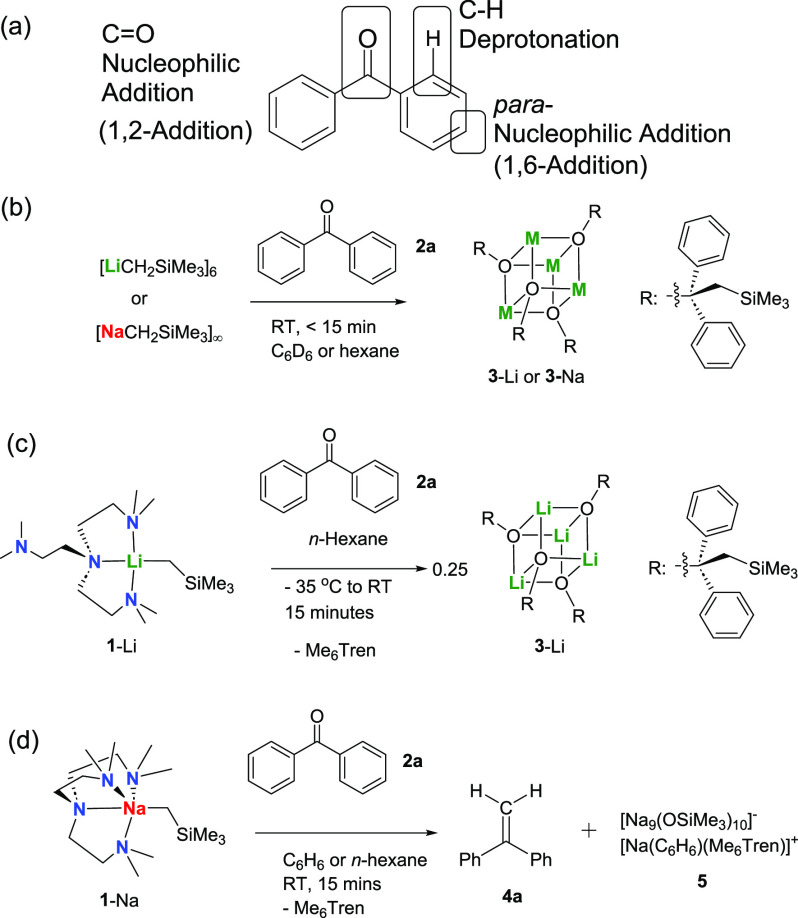
Initial Reactivity Studies of **1**-Li and **1**-Na, Employing Benzophenone (**2a**) as the Model Substrate (a) The reactive sites of benzophenone. Stoichiometric reactions between benzophenone and (b) [LiCH_2_SiMe_3_]_6_ or [NaCH_2_SiMe_3_]_∞_ and (c) **1**-Li or (d) **1**-Na.

Reactions between Group-1 metal complexes and ketones/aldehydes^60^ are not only important tools in organic synthesis, but also can provide insights into the two fundamental and essential reactivity aspects of Group-1 metal complexes: Brønsted basicity and nucleophilicity.^62^ We first employed benzophenone (**2a**) as the model substrate to test the reactivity of **1**-Li and **1**-Na. Benzophenone was chosen as it can provide three reactive sites, covering both nucleophilic addition (1,2- and 1,6-additions^61^) and Brønsted acid–base C–H deprotonation (Scheme 2a).^62,63^ Easily enolizable ketones, such as acetophenone, feature rather acidic α-Hs; hence, their reaction pattern will be predictably monopolized by the α-C–H deprotonation, i.e., enolization (vide infra).

**Figure 2:**
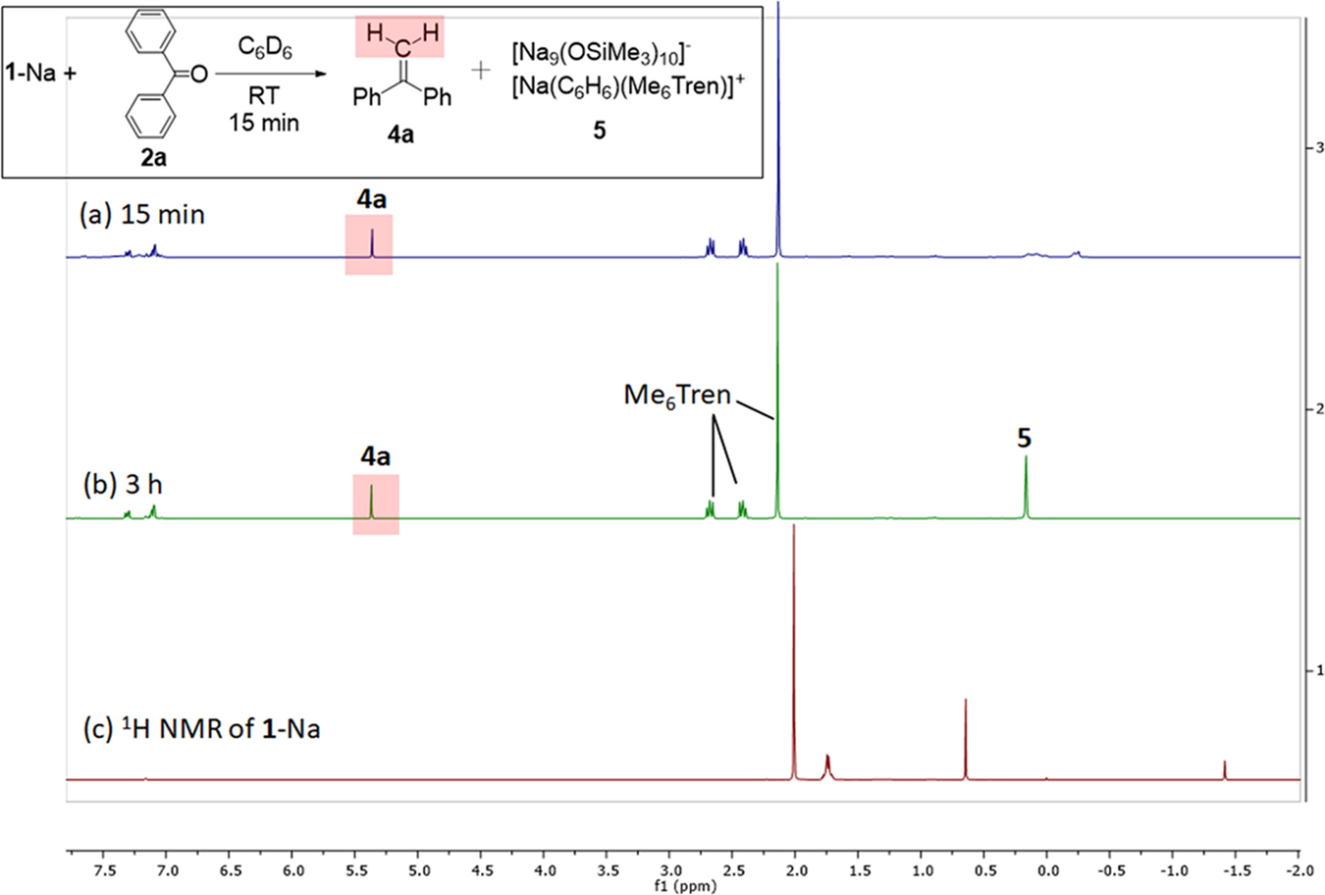
In situ ^1^H NMR spectra of the reaction between **1**-Na and benzophenone **2a** (in C_6_D_6_, 298 K) at 15 min (a) and 3 h (b), in comparison with the ^1^H NMR spectrum of **1**-Na (c).

We first tested the reactions between benzophenone and the parent polymeric/oligomeric complexes, i.e., [LiCH_2_SiMe_3_]_6_ and [NaCH_2_SiMe_3_]_∞_, which unsurprisingly produced the alkoxide tetramers [MO{C(Ph)_2_(CH_2_SiMe_3_)}]_4_ (**3**-Li/Na) (Scheme 2b), the SCXRD structures of which are displayed in the Supporting Information (**3**-Na, Figure S62) or in our previous report (**3**-Li^51^). The formations of **3**-Li/Na are the result of M–C bond nucleophilic addition toward the C=O bond. Regarding the monomers, **1**-Li was reported to react with benzophenone producing **3**-Li as well, where the Me_6_Tren ligand dissociates (Scheme 2c).^51^ Following the abovementioned paradigm, one would expect that the reaction between **1**-Na and benzophenone to produce the nucleophilic addition product **3**-Na as well. However, to our surprise, instead of **3**-Na, in situ ^1^H NMR spectroscopic monitoring exhibited a diagnostic singlet at 5.36 ppm within 15 min at room temperature, accompanied by a complete consumption of **1**-Na and formation of free Me_6_Tren ligand (Figure 2a). The singlet, along with the aromatic Hs, is consistent with 1,1-diphenyl ethylene (**4a**) via a thorough NMR comparison (^1^H, ^13^C, DEPTs, HMQC) with the authentic sample. Another new compound **5** with a ^1^H NMR singlet at approximately 0.2 ppm was formed slowly (within 3 h at room temperature) (Figure 2b). This was later identified by SCXRD study as a cluster byproduct [Na_9_(OSiMe_3_)_10_]^−^[Na(C_6_H_6_)(Me_6_Tren)]^+^ (**5**) (see Figure S61 in SI for **5**’s structure). The reaction was scaled up in benzene and *n*-hexane, which produced the same results.

**Scheme 3:**
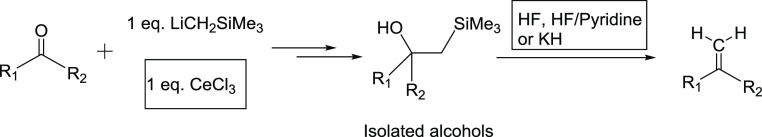
Johnson’s Modification of Peterson Olefination, Using [LiCH_2_SiMe_3_]_6_, CeCl_3_ and Acidic/Basic Conditions^71−73^

The formation of **4a** involves the cleavage of a strong C–Si bond, resulting in the formation of a C=C double bond in place of the C=O bond, reminiscent of the classic methylenation by Tebbe et al.^64^ and Wittig and co-workers.^65,66^ In the organometallic chemistry regime, such C–Si bond cleavage in a M–CH_2_SiMe_3_ linkage (M: transition-metal; f-, p-, or s-block metal) is scarce but was not unknown.^67^ A closely relevant class of reactions is Peterson methylenation.^68−70^ For organo-alkali metal reagents, [LiCH_2_SiMe_3_]_6_ has been used for Peterson methylenation since the 1980s^71^ (Scheme 3), but it has to work with a stoichiometric amount of CeCl_3_ in a stepwise manner, i.e., the intermediate alcohols must be isolated then treated with acidic/basic conditions to yield the olefins.^72,73^

**Scheme 4:**
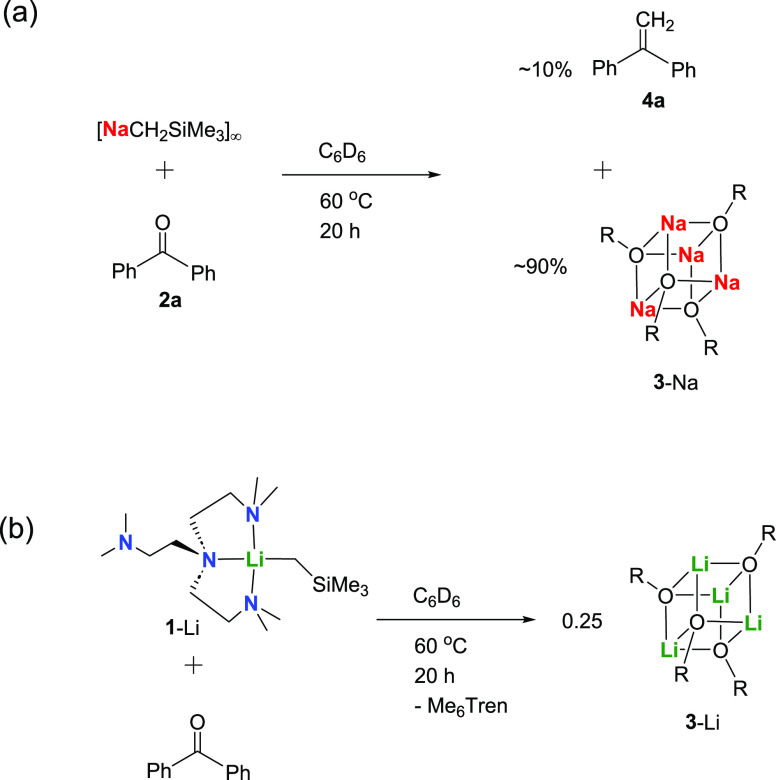
Control Reactions in C_6_D_6_ at 60 °C (a) [NaCH_2_SiMe_3_]_∞_ and benzophenone, without Me_6_Tren; (b) **1**-Li and benzophenone (reported in ref (51). R: −C(Ph_2_)(CH_2_SiMe_3_).

We were intrigued to find out the role of the Me_6_Tren ligand in the **1**-Na-mediated benzophenone methylenation. Since the free Me_6_Tren ligand was observed in the in situ NMR monitoring, one may postulate that the Me_6_Tren does not play any role. However, as mentioned earlier, a control reaction between [NaCH_2_SiMe_3_]_∞_ and benzophenone suggests otherwise (Scheme 4a). In the absence of Me_6_Tren, **3**-Na was the major product with only small amount (∼10%) of the olefin **4a**, even at an elevated temperature (60 °C) for an extended reaction time (20 h) (Scheme 4a). Hence, clearly, although Me_6_Tren appears as coordination-free, it does play an essential role in the methylenation. Another key factor is metal identity. The control reaction between **1**-Li and benzophenone (Scheme 4b) produced **3**-Li and free Me_6_Tren.^51^ Despite the presence of one equivalent of Me_6_Tren, **3**-Li does not convert into the olefin **4a** at all, even after heating at 60 °C for 20 h. Therefore, it is clear that two factors play underpinning roles in the benzophenone methylenation: (1) Me_6_Tren ligand must be present; (2) Na^+^ is essential.

**Scheme 5:**
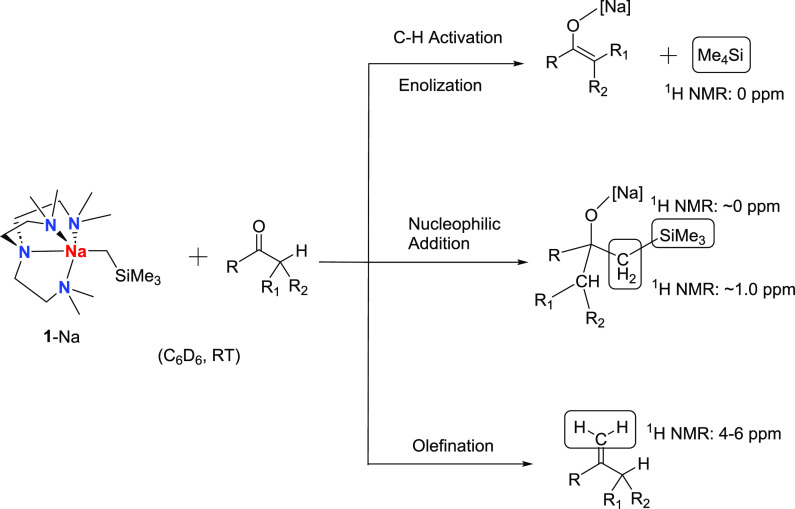
Three Major Competing Reaction Patterns between **1**-Na and Organic Carbonyl Substrates, and Their Corresponding Diagnostic ^1^H NMR Chemical Shifts

Encouraged by the facile benzophenone methylenation, we examined stoichiometric reactions between **1**-Na and various hydrocarbon and fluorinated ketones and aldehydes. We anticipate three major competing reaction patterns (Scheme 5): (1) C–H deprotonation to form enolates (enolization), (2) nucleophilic addition to form alkoxides, and (3) methylenation. The competing reaction patterns were monitored at the NMR scale by their corresponding characteristic ^1^H NMR chemical shifts (Scheme 5).

The results are listed in Table 1. For the easily enolizable ketone acetophenone (**2b**), enolization was observed exclusively. For less enolizable ketones, such as dicyclohexyl ketone (**2c**) and phenyl cyclohexyl ketone (**2d**), methylenation was observed as the dominating reaction pattern. Nonenolizable ketones, such as phenyl *tert*-butyl ketone (**2e**) and adamantanone (**2f**), were observed to undergo methylenation as well. Though slower than the ketones, the methylenation also works for aldehydes (**2g**, **2h**). On the course of the conversion from benzaldehyde (**2g**) to styrene (**4g**), we observed the nucleophilic addition product as the intermediate.^59^ Hence, it is sensible to postulate that, with the presence of Me_6_Tren, the nucleophilic addition product undergoes methylenation. The long methylenation time (3 days) for **2g** can be explained by its more stable nucleophilic addition product as a result of its reduced steric congestion compared with the ketones. In comparison, the methylenation of a bulkier aldehyde **2h** is much faster (room temperature, 30 min). We postulate that the steric factor plays a crucial role here: **1**-Na-mediated methylenation prefers bulkier substrates, because their corresponding nucleophilic addition products (alkoxides) are less stable. The preference of sterically bulky substrates is in sharp contrast with the classic Wittig/Tebbe systems, which operate via a crowded 2 + 2 cycloaddition transition state, hence favoring less bulky substrates.

**Table 1:**
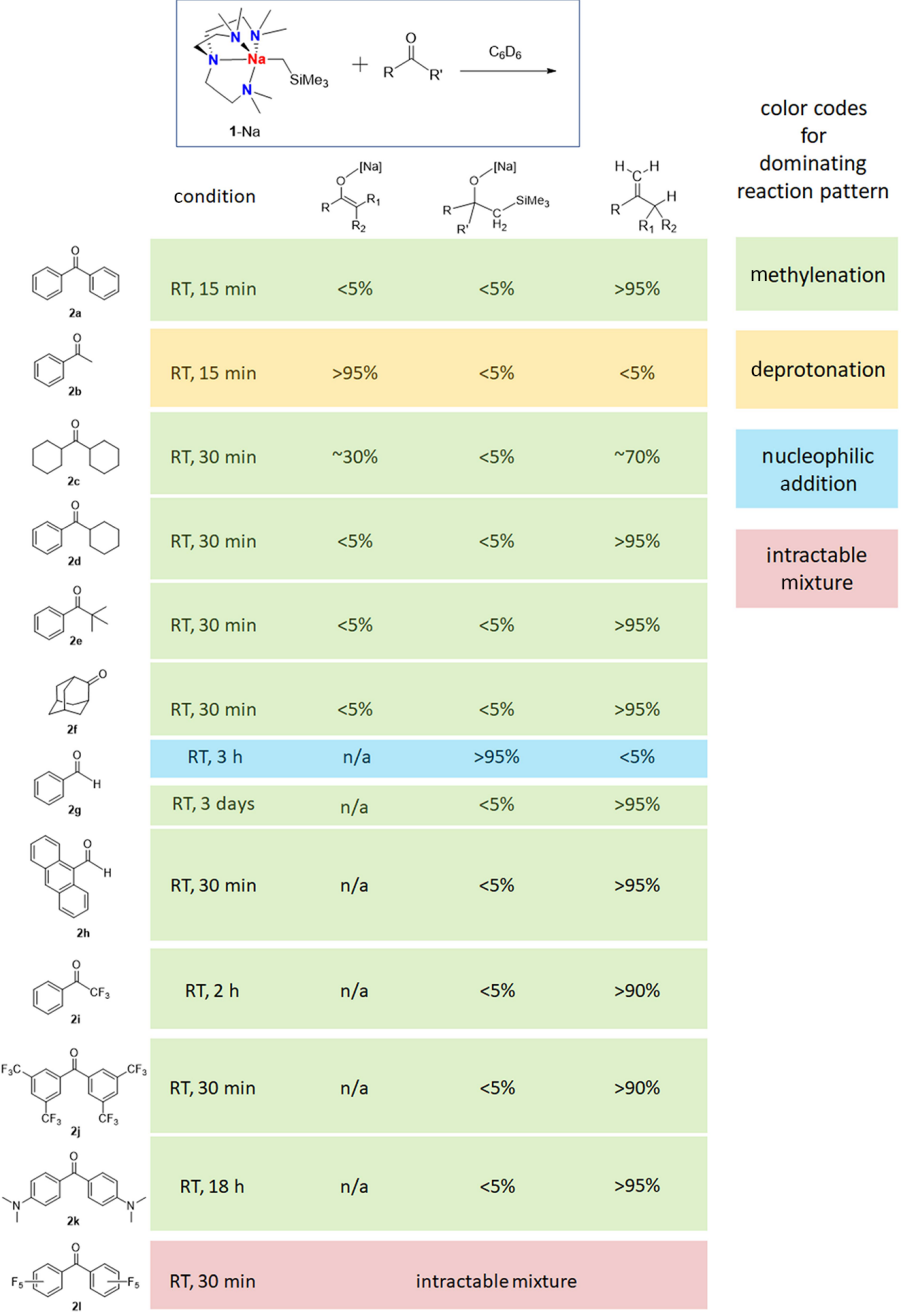
Results of Stoichiometric Reactions between **1**-Na and Ketones/Aldehydes

Beyond the hydrocarbon ketones and aldehydes, we also tested reactions between **1**-Na and ketones with strong electron-withdrawing or electron-donating groups, to examine the influence of C=O bond electronic properties. Reactions between **1**-Na and electron-poor fluorinated ketones, namely, 2,2,2-trifluoroacetophenone (**2i**) and 3,3′,5,5′-tetrakis(trifluoromethyl)benzophenone (**2j**), both produce methylenation products smoothly (Table 1). The electron-rich 4,4′-bis(*N*,*N*-dimethylamino)benzophenone (Michler’s ketone,^74^**2k**) also reacts with **1**-Na to produce the methylenation product. However, perfluorobenzophenone (**2l**) reacts with **1**-Na to produce an intractable mixture, without observable formation of the corresponding olefin, nor the nucleophilic addition product. We attribute the negative result with **2l** to its highly electron-deficient perfluorophenyl groups, which are labile to nucleophilic substitution.^75,76^ Since s-block metal alkyl complexes were reported to undergo nucleophilic substitution with arenes such as benzene,^77,78^ it is sensible to postulate that the Na–C bond in **1**-Na, which is rather nucleophilic, reacts with the electron-deficient −C_6_F_5_ group in **2l** following a nucleophilic route.

### Stoichiometric Reactions between [Na(CH_2_SiMe_3_)(Me_6_Tren)] (**1**-Na) and Ester or Amide: O vs N

Group-1 and 2 metal alkyl complexes were reported to undergo a variety of reactions toward organic amides and esters, spanning from conventional nucleophilic addition^79−82^ and substitution,^83,84^ to less common reductive coupling (in the presence of an redox-active iron cluster).^85^ From an electronic perspective, in esters and amides, there is pronounced electron density donation from the lone pair of N/O atoms to the carbon atom of C=O bonds, which renders the C=O bond less electrophilic compared with ketone/aldehyde, i.e., less labile to nucleophilic addition. This was clearly demonstrated by Beak and Brown as early as in 1982, where the amide functional group acted as a directing group for organolithium-mediated arene *ortho*-lithiation,^86^ where phenyl ring deprotonation occurred, instead of amide nucleophilic addition. Considering the versatile reactivity profile of ester/amide, we were intrigued to examine their reaction patterns with our organosodium monomer complex **1**-Na.

**Figure 3:**
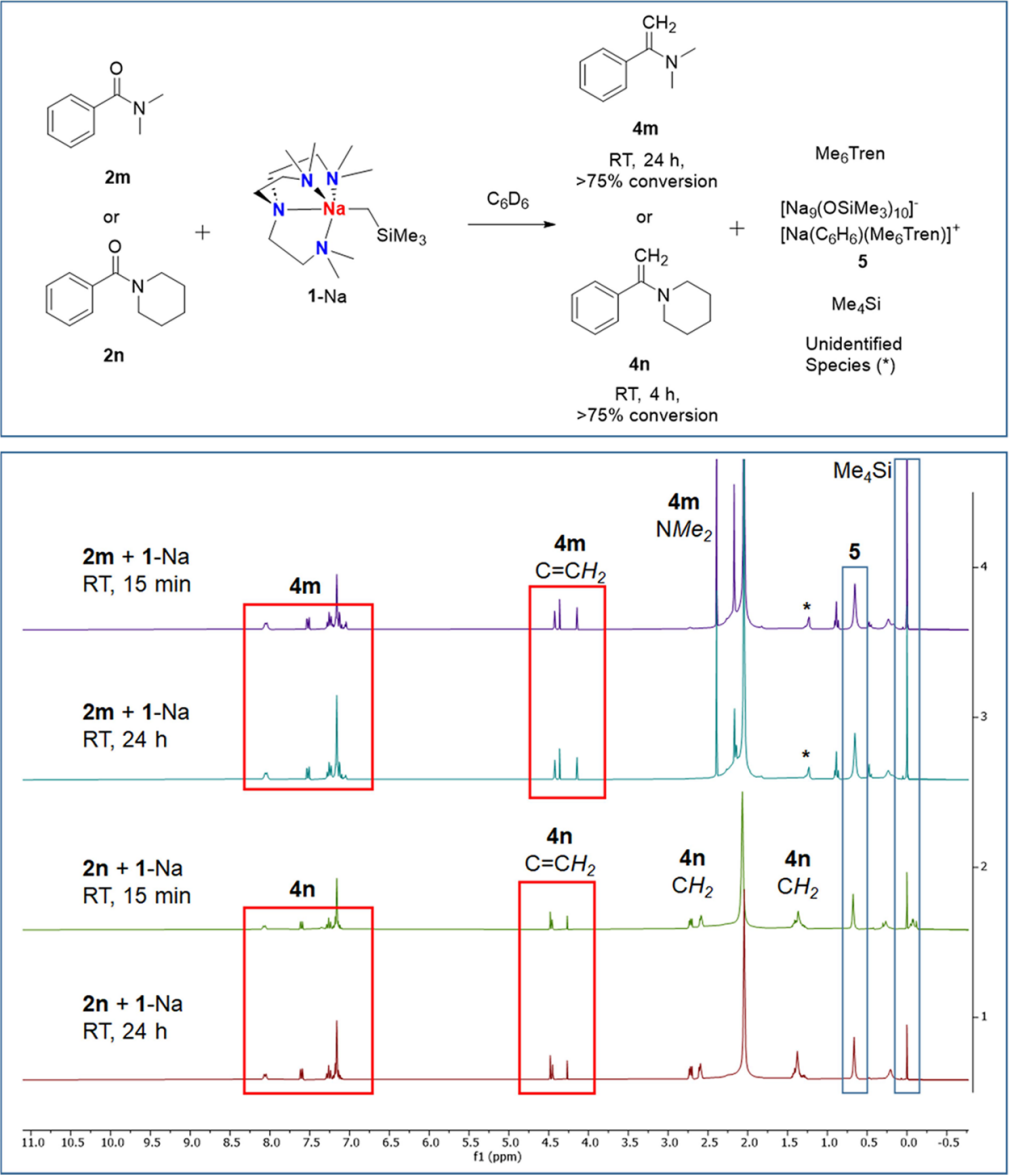
In situ ^1^H NMR spectra of the reactions between **1**-Na and *N*,*N*-dimethylbenzamide (**2m**) or 1-benzoylpiperidine (**2n**) (in C_6_D_6_, 298 K).

*N*,*N*-Dimethylbenzamide (**2m**), 1-benzoylpiperidine (**2n**), and phenyl benzoate (**2o**) were employed as readily available and representative model substrates. Reactions between the amides **2m/2n** and one equivalent of **1**-Na in C_6_D_6_ proceeded predominantly toward methylenation (over 75% conversion calculated from ^1^H NMR) at room temperature within 24 h (for **4m**) or 4 h (for **4n**), respectively (Figure 3). A small amount of Me_4_Si was observed in both **4m** and **4n** reactions (circa 15–20%), indicating deprotonation side-reactions, which are not surprising considering the Brønsted acidity of the N(C*H*_3_)_2_ (**2m**) and NC*H*_2_ (**2n**) groups.

**Figure 4:**
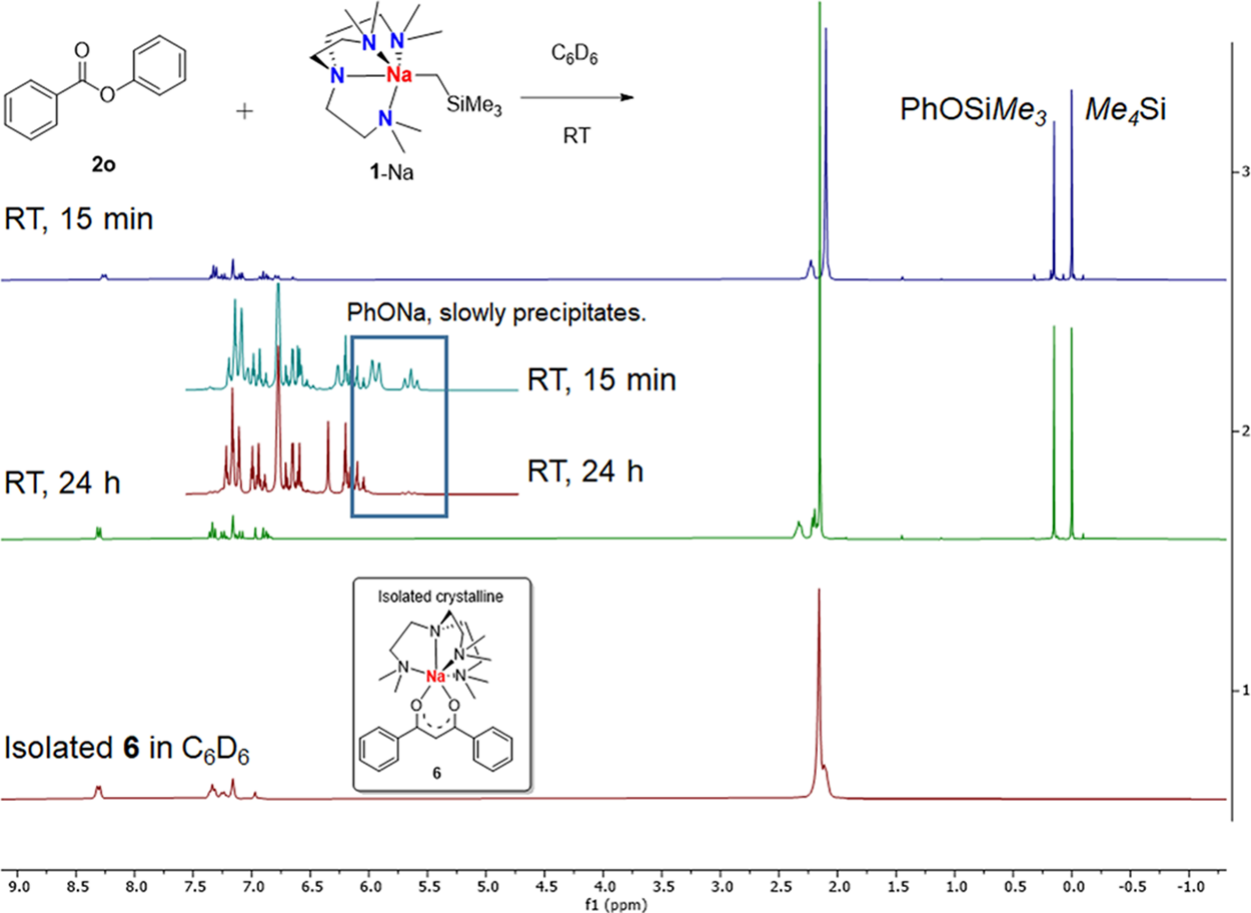
In situ ^1^H NMR spectra of the reaction between **1**-Na and phenyl benzoate **2o** and ^1^H NMR spectrum of isolated crystalline **6** (all in C_6_D_6_, 298 K).

**Figure 5:**
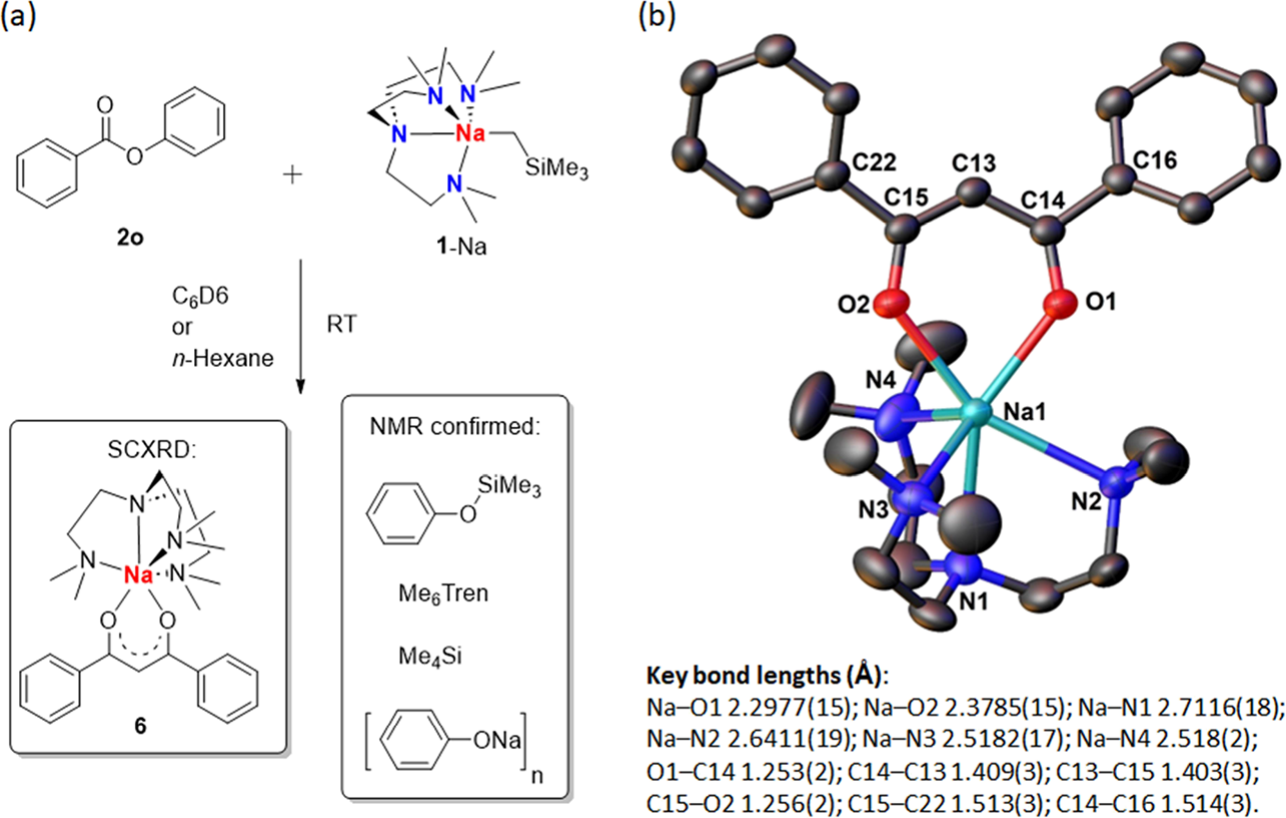
(a) Reaction between **1**-Na and ester **2o**; (b) SCXRD structure of **6**; H atoms are omitted for clarity.

However, reaction between phenyl benzoate (**2o**) and one equivalent of **1**-Na in C_6_D_6_ did not produce the methylenation product, judging from the lack of the diagnostic olefin C^sp2^–*H* signals in its ^1^H NMR spectrum (Figure 4). Instead, Me_4_Si, PhOSiMe_3_^87^ and sodium phenoxide [PhONa]_*n*_ were identified (Figure 4). Scaling up the reaction and crystallization in *n*-hexane at −35 °C afforded yellow needle-shaped crystals at low but reproducible yield (approximately 17% based on Na), the structure of which was characterized as an Me_6_Tren-coordinated sodium phenyl-acetylacetonate complex **6** (Figure 5). Coordination-free Me_6_Tren was recovered from the mother liquor of crystallization.

**Scheme 6:**
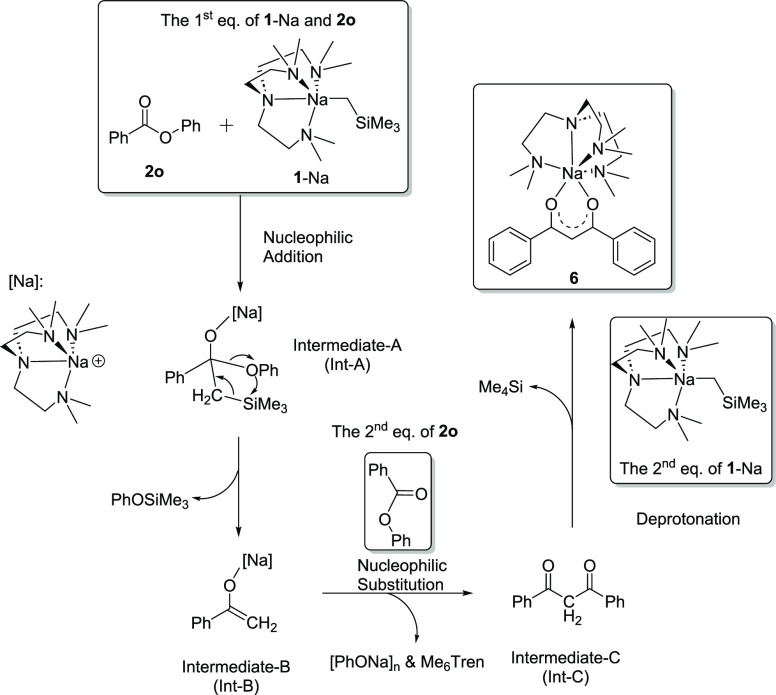
Postulated Mechanism of the Reaction between **1**-Na and **2n** The postulated reaction intermediates are identified as Int-A, Int-B, and Int-C.

The outcome of the reaction between **1**-Na and **2o** is surprising, involving cleavage of C–O bonds and formation of O–Si and C–C/C=C bonds. As a net result, two [PhC(O)] units (originated from the ester **2o**) are linked by a [CH] unit. We hypothesized that the [CH] unit is from [NaCH_2_SiMe_3_]. To the best of our knowledge, this reaction pattern is hitherto unknown for esters. The formations of PhOSiMe_3_ and Me_4_Si provide crucial clues, allowing us to postulate a sensible reaction mechanism to explain the outcome (Scheme 6): The reaction is initiated by a nucleophilic addition of the Na–C bond toward the C=O bond, followed by an intramolecular PhOSiMe_3_ elimination, driven by the formation of a strong O–Si bond. The resultant enolate intermediate underwent a nucleophilic substitution with the second equivalent of **2o** and eliminated [PhONa]_*n*_ and free Me_6_Tren ligand. Subsequently, the resultant 1,3-diphenyl-1,3-propanedione (Int-C in Scheme 6) is deprotonated by the second equivalent of **1**-Na to produce **6** and Me_4_Si.

We rationalize the dramatically different reaction patterns between the amides **2m**/**2n** and ester **2o** by the key step of intramolecular PhESiMe_3_ (E: O, N) elimination (Int-A in Scheme 6): the formation of a relatively weak N–Si bond (bond dissociation energy (BDE) 493 kJ mol^–1^) is less favorable than a strong O–Si bond (BDE 798 kJ mol^–1^).^88^ Though we believe that the postulated reaction mechanism in Scheme 6 is sensible, it should not be treated as a calculated reaction pathway analysis, which is currently underway in our groups, along with further effort to expand the ester/amide substrate scopes for exploiting the new reactivity. These results will be published in due course as follow-ups of the initial discovery presented herein.

### Li vs Na, Nucleophilic Addition vs Methylenation: Probing the Origin of the Diversified Reaction Patterns Using Density Functional Theory (DFT) Calculations—A Case Study of Benzophenone (**2a**)

To gain insight into the different reactivity observed in this reaction system, DFT calculations first looked at the **1**-M (M = Li, Na) species, and in particular the coordination mode of the Me_6_Tren ligand. Using a dispersion and solvation corrected methodology (see the SI for full details), the κ^4^ coordination mode, where all three sidearms of the Me_6_Tren ligand are coordinated to the metal center, is preferred for both Na and Li, by 6.6 and 3.3 kcal mol^–1^ respectively, compared to the κ^3^/two sidearms coordinated conformer. Natural charges show little differentiation between the metal centers (*q*_M_ = 0.81–0.83) or the directly coordinated carbon atom (*q*_C_ = −1.53 – −1.58) of the CH_2_SiMe_3_ fragment for both conformers (see the SI), suggesting that the electronics of the starting complex does not influence reactivity. The stability of the tetrameric alkoxide product **3**-M (M = Li, Na) was also explored, with exergonic cluster formation being preferred for M = Li by 19.5 kcal mol^–1^ in comparison to M = Na, with an overall free energy of formation of −142.5 kcal mol^–1^ for **3**-Li.

**Figure 6:**
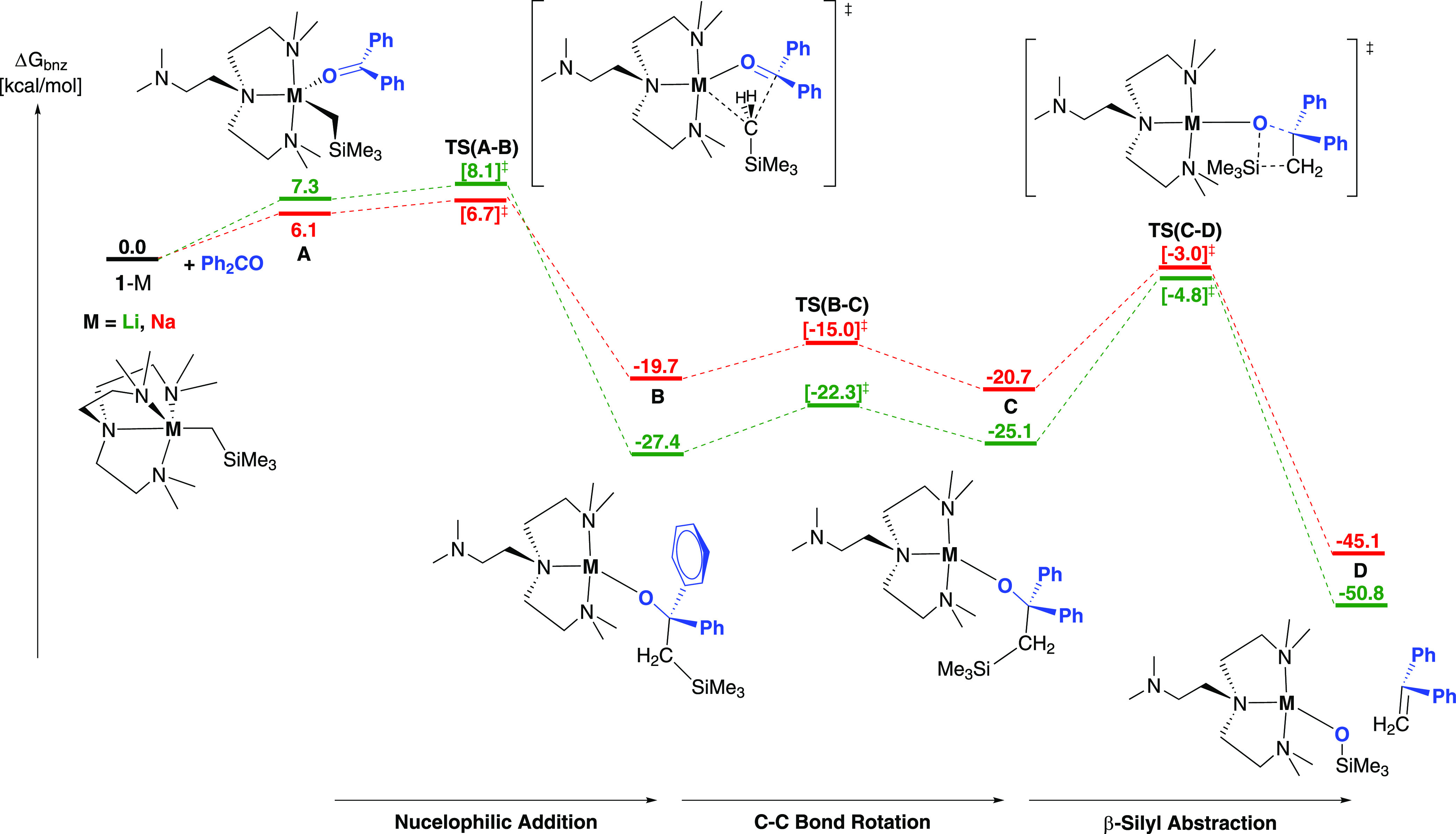
DFT-calculated free energy profile (BP86-D3BJ(C_6_H_6_)/6-311++G**//BP86/6-31G**&SDDALL, in kcal mol^–1^) for the reaction of **1**-M (M = Li, Na) with benzophenone (in blue). Calculated intermediates are named as **A**, **B**, and **C**, while the calculated Me_6_Tren-chelated silyloxide product is **D**. Color codes for the reaction pathways: Li = green pathway, Na = red pathway.

Reaction profiles were modeled for both **1**-Na and **1′**-Li (the hypothetic κ^4^ version of **1**-Li), with and without Me_6_Tren coordination, and for no Group-1 metal involvement, with the relative zero species being either **1**-Na or **1′**-Li, both the κ^4^ species (Figure 6). The first step of the reaction profiles requires endergonic coordination of **2a** (benzophenone) to the metal after dissociation of one of the N sidearms (to the κ^3^ conformer). This is easier for the sodium system, with intermediate **A**-Na 6.1 kcal mol^–1^ higher in free energy than **1**-Na. Similarly, the more sterically crowded (due to the smaller ionic radius of Li^+^) **A**-Li was eventually optimized and found to be 7.3 kcal mol^–1^ higher in free energy than **1′**-Li, see Figure 6. Nucleophilic addition (**TS(A-B)**) of the silyl alkyl group to the carbonyl carbon proceeds irreversibly by a barrier of 0.6 (**A**-Na) and 0.8 (**A**-Li) kcal mol^–1^, relieving the steric strain of the five coordinate metal center, to give the exergonic intermediate **B**, where the alkoxide is bound to the metal through the oxygen of **2a** and the SiMe_3_ group in an *anti*-conformation (O–C–C–Si dihedral close to 180°).

Subsequent low barriers (4.7 (**B**-Na) and 5.1 (**B**-Li) kcal mol^–1^) to C–C bond rotation (**TS(B-C)**) of the newly formed bond ensure that the SiMe_3_ group moves to a *syn* conformation (**C**), with a O–C–C–Si dihedral value close to 0°. Here, the intermediate is now primed for the following β-silyl abstraction, where the oxophilic trimethylsilyl is abstracted by the negatively charged oxo, involving simultaneous O–C and C–Si cleavage as further overlap occurs between the carbon centers to form the olefinic C=C π bond. This concerted β-silyl abstraction process was located via a four-membered transition structure **TS(C-D)**, which resembles a 1,2-oxasiletanide reported by Okazaki and co-workers in 1992,^89^ and is rate limiting for both the Na and Li ligated systems, with barriers of 17.2 and 22.5 kcal mol^–1^ found at −3.0 and −4.8 kcal mol^–1^ for **C**-Na and **C**-Li respectively. The higher barrier of **C**-Li could determine that the reaction does not proceed for the Li system, as experimentally observed. This then affords the olefin product adduct, **D**, which is irreversibly and exergonically formed at −45.1 kcal mol^–1^ for **D**-Na (−50.8 kcal mol^–1^ for **D**-Li).

In the absence of the Me_6_Tren ligand, reaction profiles were also computed for **2a** reacting with [CH_2_SiMe_3_]^−^ in the presence of one atom of Na or Li (see the Supporting InformationFigure S67 for Na and Figure S68 for Li, respectively). Coordination of **2a** to M(CH_2_SiMe_3_) raised the free energy of **^M^A** to over 16 kcal mol^–1^ followed by nucleophilic addition at 23.5 and 21.4 kcal mol^–1^, for M = Na and Li, respectively. Without the ligand to support the electron density on the metal, the nucleophilic addition barriers are larger by 8.2 and 5.3 kcal mol^–1^. Subsequent rotation about the new C–C bond, **TS(B-C)**, has a free energy barrier of 6.9 kcal mol^–1^ for Na (Li = 6.7 kcal mol^–1^), now larger due to a π-interaction between the metal and a phenyl ring of **2a**. The 1,2-oxasiletanide transition state, **TS(C-D)**, occurs at 15.9 kcal mol^–1^ (Li = 19.5 kcal mol^–1^). Again, β-silyl abstraction and olefin formation were notably easier for the Na system by 4.5 kcal mol^–1^, compared to 5.3 kcal mol^–1^ with the ligated systems. Hence, we conclude that ligation is the key to a lower barrier for nucleophilic addition. This explains the necessity of the Me_6_Tren ligand to facilitate the methylenation.

In the absence of a metal (Figure S69), the adduct **a** is substantially higher in free energy at 32.5 kcal mol^–1^ with the system balanced by the separated ion pair complex [(κ^4^-Me_6_Tren)Na]. The equivalent nucleophilic addition transition structure, **TS(a-b)**, has a barrier of 1.9 kcal mol^–1^, to form the *anti-*alkoxide conformer, **b**, at 15.9 kcal mol^–1^. Rotation about the C–C bond has two distinct barriers, **TS(b-c)1** and **TS(b-c)2** both under 2 kcal mol^–1^, and an intermediate **INT(b-c)**, before the *syn* conformer is isolated as **c** at 9.1 kcal mol^–1^. The final β-silyl abstraction step, **TS(c-d)**, was optimized at 21.5 kcal mol^–1^.

The calculated reaction pathways in Figure 6 clearly demonstrate that **1**-Na and **1′**-Li could both react with **2a** (benzophenone) and follow a similar reaction pathway. However, while the methylenation with **1**-Na proceeds at room temperature rapidly, the corresponding methylenation with **1**-Li was not experimentally observed, even at elevated temperature (60 °C). We note that, in Figure 6, the energetic barriers are consistently higher for **1′**-Li than those for **1**-Na. This could be one contributor for not observing methylenation with the Li system. However, we caution using the higher barriers of **1′**-Li as the sole explanation: the energetic differences between the barriers for **1′**-Li and **1**-Na are small (maximum 5.3 kcal mol^–1^, for **TS(C-D)**). Another potential contributor is steric, i.e., the larger Na^+^ ionic radius may favor methylenation. To summarize, we hypothesize that the observed difference between **1**-Na and **1**-Li methylenation could be a result of interplay among several factors, including a series of slightly higher energetic barriers for the Li system, and the more favorable steric factor endowed by the larger Na^+^ cation. Though a definite answer is absent for now, these calculations clearly demonstrate that organo-alkali metal reactions involve delicate balances: changing metal identity could shift the finely-balanced pathway, and result in a dramatic change of reaction outcomes, though the energetic changes of each single step may appear trivial.

Our argument can be supported by a study on aminometalations by Strohmann and co-workers,^90^ where they calculated the reaction profiles between lithium-, sodium- and potassium dimethylamide dimers [M(μ-NMe_2_)(OMe_2_)_*n*_]_2_ and 4-methyloxy styrene. The scenario is similar to our case in Figure 6 but with larger energetic differences. Nevertheless, unlike we observed here, Strohmann and co-workers did not experimentally observe different reaction patterns between the Li-, Na-, and K-amides per se. They alternated the balances by adding the second Group-1 metal ingredient to form the Lochmann–Schlosser-type heterometallic systems, which operates in a totally different mechanism. In comparison, in our system here, the difference between the metal identity itself (Li vs Na) is pronounced enough to result in divergent reaction patterns.

### Ligand-Catalyzed Organosodium-Mediated Ketone/Aldehyde Methylenation

Inspired by the presence of the coordination-free Me_6_Tren in the olefination of benzophenone, we wondered if a catalytic amount of Me_6_Tren would promote the methylenation, employing [NaCH_2_SiMe_3_]_∞_ as the stoichiometric CH_2_ transfer reagent. Compared with other popular CH_2_ transfer reagents, such as phosphorus ylides (Wittig^91^), sulfones (Julia^92^ and Kocieński^93^), titanium (Tebbe^64^), zirconium,^94^ rare-earth,^95^ molybdenum (Kauffmann^96^), or chromium (Takai^97^) reagents, the organosodium reagent [NaCH_2_SiMe_3_]_∞_ features significant sustainability advantages and, potentially, a much lower cost (for comparison, Tebbe reagent solution (0.5 M in toluene) is priced at £229 for 25 mL at Merck). The concept of ligand-catalysis (and the relevant solvent-catalysis^98^) is based on reversible coordination of a ligand (e.g., Me_6_Tren in this case) to the metal center, which has been seldom reported in Group-1 metal chemistry as early as in the 1960s^99−101^ until today,^102−104^ mostly for deprotonation reactions.

**Figure 7:**
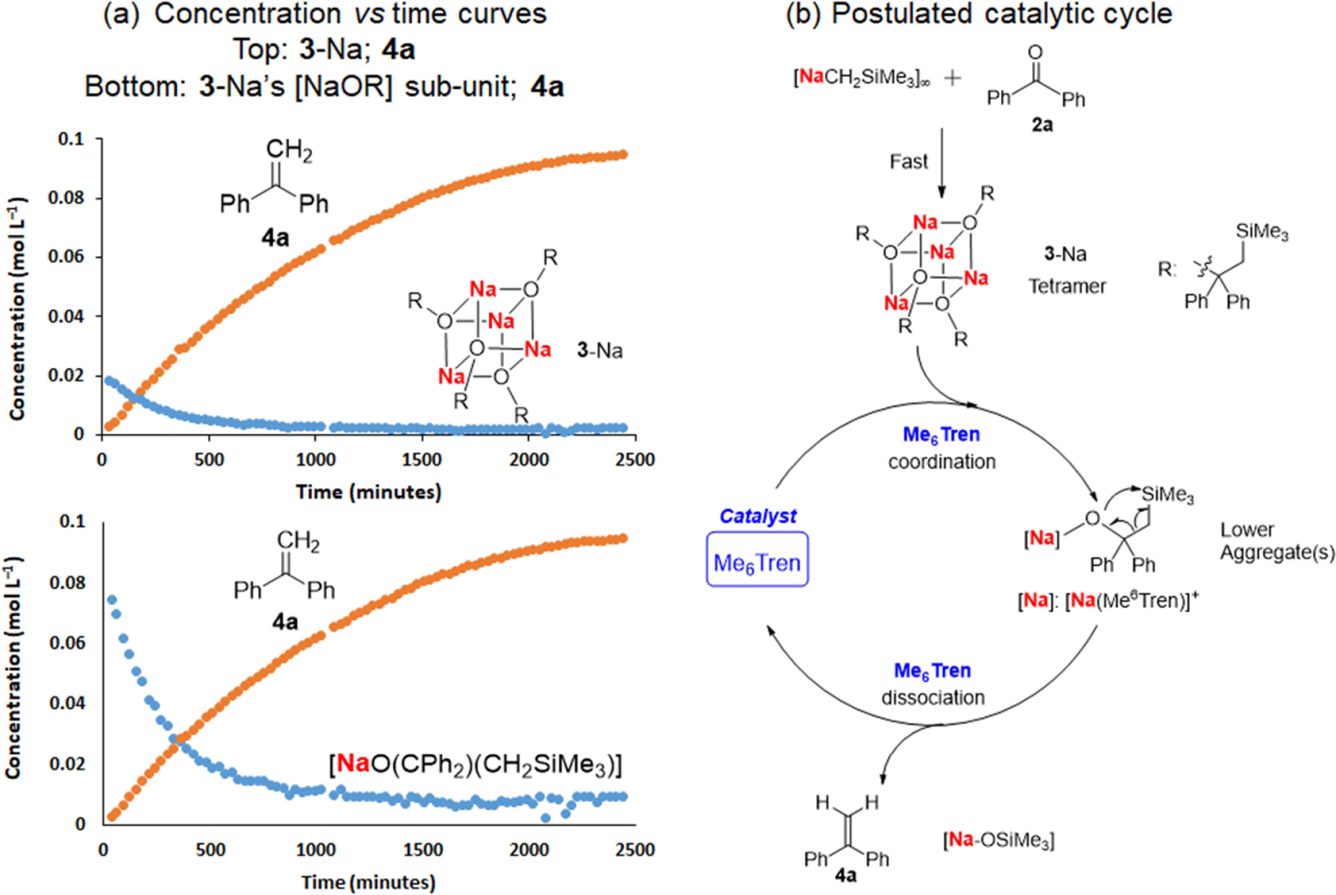
In situ ^1^H NMR monitoring of catalyzed benzophenone methylenation and its postulated mechanism. (a) Concentration *vs* time curves. Catalytic conditions: room temperature, C_6_D_6_, 5 mol % of Me_6_Tren, initial substrate concentration [**2a**]_0_ = 0.08 M; catalyst concentration [Me_6_Tren] = 0.004 M; internal standard: cyclohexane. For full details, see Supporting Information, Section 1.6, page S41. (b) Postulated catalytic cycle.

By using 20 mol % of Me_6_Tren as a catalyst, the 1:1 reaction between [NaCH_2_SiMe_3_]_∞_ and benzophenone yielded the olefin **4a** in >95% conversion within 3 h at room temperature (Table 2, entry 1). Lowering the catalyst loading to 5 mol % worked well, too; despite the longer reaction time (16 h, room temperature) needed to reach >95% conversion (Table 2, entry 2). In situ ^1^H NMR monitoring of the 5% Me_6_Tren-catalyzed benzophenone methylenation reveals that it is a stepwise reaction. First, benzophenone was fully and rapidly converted into the alkoxide tetramer **3**-Na within 10 min at room temperature, this then slowly converted into the olefin **4a** (Figure 7a). The concentration vs time curves of **3**-Na’s consumption and **4a**’s formation are presented in Figure 7a. It should be noted that **3**-Na is a tetramer (Figure 7a top). For clarity, the following discussion will use the concentration of its monomeric subunit [NaOR] (Figure 7a bottom), which is higher than the concentration of **3**-Na itself and hence, elucidates the trend more clearly. It can be observed that the consumption of [NaOR] and the formation of **4a** follow different trends (Figure 7a bottom). At the initial stage of reaction (0 to ∼300 min), [NaOR] is rapidly consumed, but **4a** only rises slowly. This indicates the presence of intermediate(s), which we identified on the ^1^H NMR (see the Supporting InformationFigure S56, highlighted in pink band) and postulate to be lower aggregate(s) of **3**-Na. Hence, we postulate a catalyzed reaction mechanism as depicted in Figure 7b: [NaCH_2_SiMe_3_]_∞_ rapidly reacts with benzophenone in a nucleophilic addition manner to form **3**-Na (a tetramer), which is subsequently slowly dissembled by the catalytic amount of Me_6_Tren into lower aggregate(s) intermediate(s), which then undergoes fast methylenation and regenerates the ligand catalyst Me_6_Tren. The lower aggregate(s) play an important role in our hypothesis but due to their low concentration and fast conversion into the methylenation product, we could not experimentally identify them unambiguously.

**Table 2:**
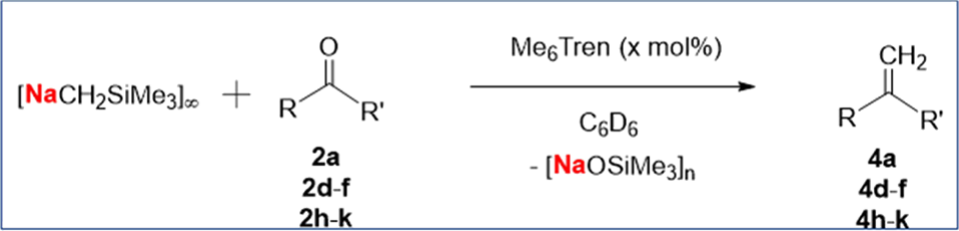

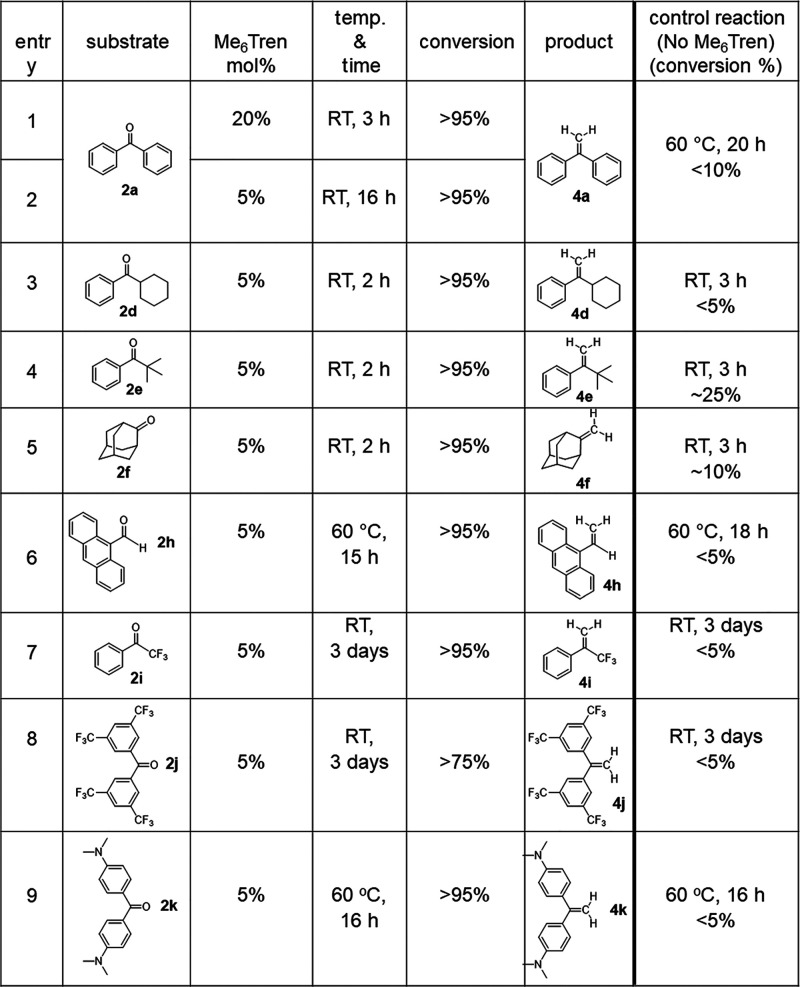
Me_6_Tren-Catalyzed Ketone/Aldehyde Methylenation Mediated by [NaCH_2_SiMe_3_]_∞_ and Corresponding Control Reactions with the Conversions Measured by ^1^H NMR (See the Supporting Information for Details)

**Figure 8:**
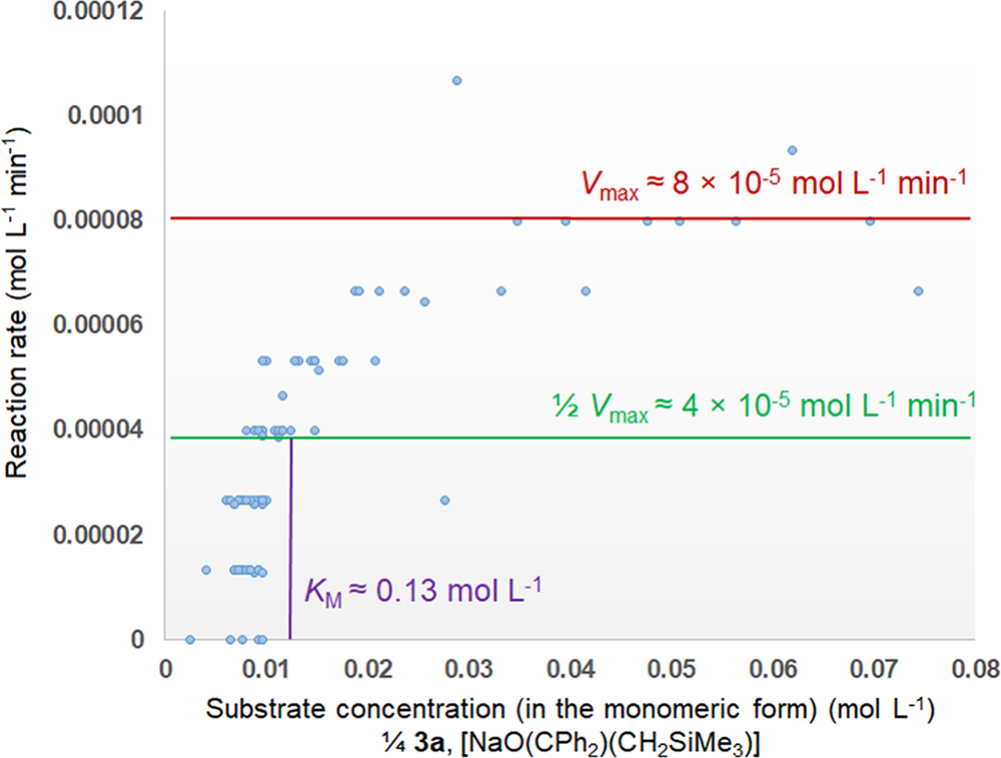
Michaelis–Menten saturation plotting (reaction rate-substrate concentration) for 5 mol % Me_6_Tren-catalyzed methylenation of benzophenone (**2a**) with NaCH_2_SiMe_3_. The solid lines for *V*_max_, 1/2 *V*_max,_ and *K*_M_ are for visual guide only. The analyses represented herein should be treated as qualitative, rather than quantitative.

To gain further insight into the catalytic process, we adopted the Michaelis–Menten (M–M) method^105^ to analyze the kinetic data of 5 mol % Me_6_Tren-catalyzed benzophenone methylenation. By adopting the M–M method, we hypothesize that the Me_6_Tren in our system plays the role of enzyme in the classic M–M model. The average reaction rate at each time point (*t_n_*) was calculated by dividing the increase of product concentration (in mol L^–1^; Δ[**4a**] = [**4a**]_*n*_ – [**4a**]_*n*−1_; *n* = 1, 2, 3...; [**4a**]_0_ = 0 mmol L^–1^) by time length (in minute; Δ*t* = *t_n_* – *t*_*n*–1_), i.e., d[**4a**]/d*t*, in the unit of mol L^–1^ min^–1^. A resultant reaction rate-substrate concentration plotting (Michaelis–Menten saturation plotting) is displayed in Figure 8. The key parameter of our interest is the Michaelis constant, *K*_M_, which is defined as the substrate concentration when the reaction rate is half of the maximum rate *V*_max_. In the classic M–M model, *K*_M_ is a probe for the enzyme’s affinity to substrate: a small *K*_M_ indicates high enzyme-substrate affinity, i.e., the reaction rate will approach *V*_max_ at lower substrate concentration.^106^ Examining Figure 8 indicates that, though a quantitative understanding of the system is out of our reach at the moment (largely due to lack of accurate reaction rate data), qualitatively speaking, our system features a relatively small *K*_M_ (circa 0.13 mol L^–1^), which indicates a strong affinity between Me_6_Tren and the [NaO(CPh_2_)(CH_2_SiMe_3_)]_*n*_ species.

Encouraged by the success of benzophenone, we subsequently expanded the catalytic substrate scope to **2d–f** and **2h–k**, all of which exhibited good to excellent catalytic activities (Table 2, Entries 3–9). For **2d**–**f**, 5 mol % of Me_6_Tren catalyzed their methylenations within 2 h at room temperature with >95% conversion. The catalytic methylenation of 9-anthracenealdehyde (**2h**) is slower (15 h) and requires an elevated temperature (60 °C) to achieve >95% conversion. The fluorinated (**2i**, **2j**) and electron-rich (**2k**) ketones all need longer reaction times or higher temperature to achieve high conversions, too.

## Conclusions and Outlook

**Figure 9:**
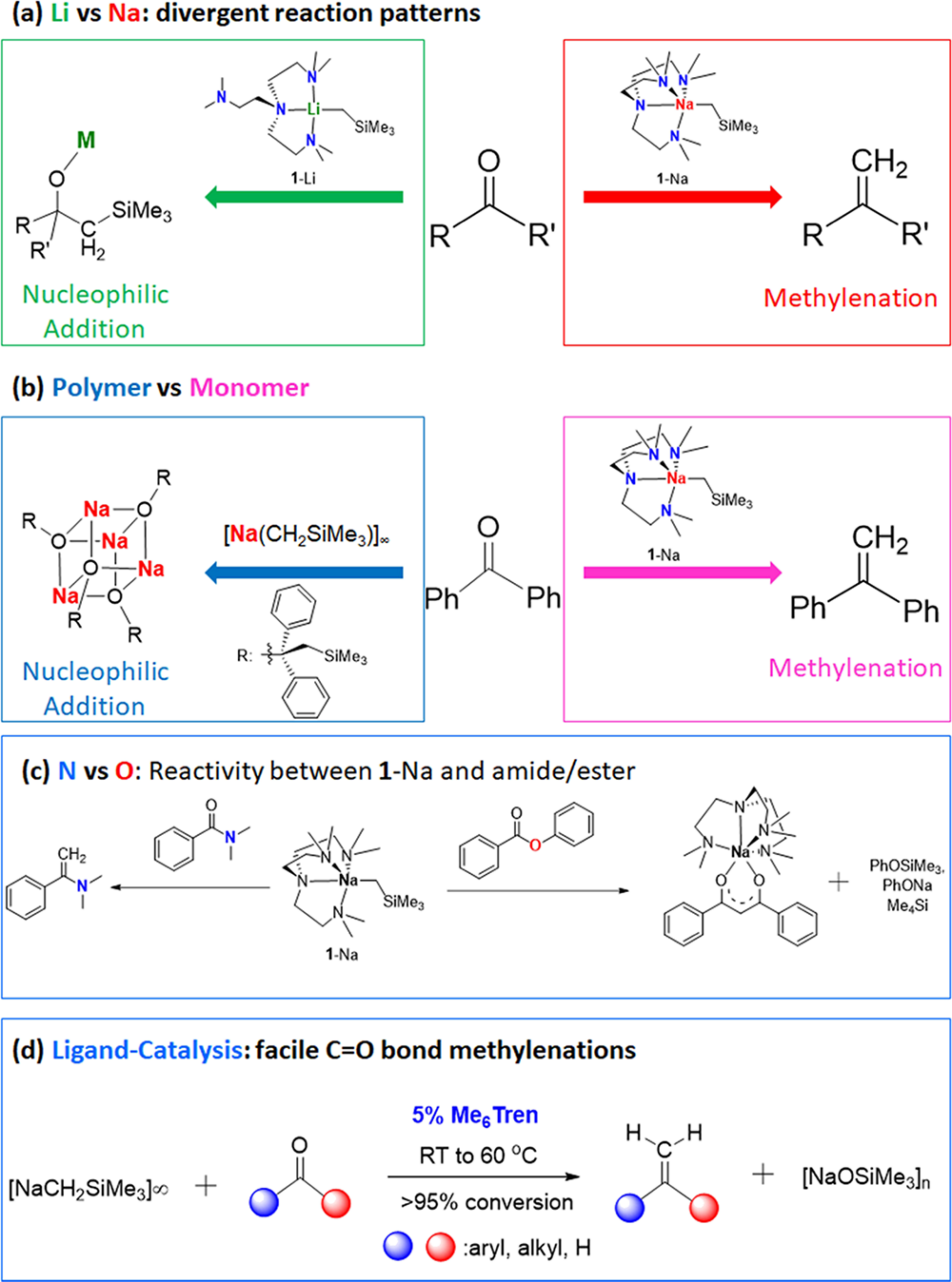
Schematic summary of the work herein. (a) Divergent reaction patterns between organosodium and organolithium complexes toward ketone/aldehyde; (b) diversified reactivity of organosodium complexes: polymer vs monomer; (c) diversified reactivity of **1**-Na toward amide/ester; (d) ligand-catalyzed C=O bond methylenation using organosodium reagent as a sustainable and low-cost CH_2_ feedstock.

In conclusion, in this work we reported several new discoveries that push forward the frontiers of organosodium, and more generally, organo-alkali metal chemistry. First, we reported the first unequivocal observation of divergent reaction patterns between organolithium and organosodium reagents, with a variety of ketone/aldehyde substrates (Figure 9a). Second, we clearly demonstrated different reaction patterns of the NaCH_2_SiMe_3_ monomer (**1**-Na) and polymer ([NaCH_2_SiMe_3_]_∞_) toward model ketone substrate: benzophenone (Figure 9b). Beyond ketones and aldehydes, **1**-Na was found to react with amide and ester via different pathways, which further demonstrated the hitherto unexplored versatile reactivity of organosodium complexes. Based on the stoichiometric reactivity studies, we pushed the organosodium-mediated ketone/aldehyde methylenations into the catalysis regime and developed a ligand-catalysis strategy, utilizing as low as 5 mol % of neutral amine ligand Me_6_Tren as the catalyst (Figure 9d).

This work unlocks new chemical space by proving the concept that, by tuning the chemical environments of organo-alkali metal complexes, it is possible to lead to diversified and divergent reaction patterns depending on the metal identity and aggregate size. Further work is underway in our groups in four directions: (1) Comprehensively explore the similarities and differences in reaction patterns between **1**-Li and **1**-Na and thoroughly understand their mechanisms; (2) push the strategy into heavier Group-1 metal congeners, i.e., organopotassium, organorubidium, and organocesium; (3) exploit the ligand-catalysis strategy by employing more catalysts, such as bidentate tetramethylethylenediamine (TMEDA)^107^ and *hexa*-dentate *N*,*N*′,*N*″-tris-(2-*N*-diethylaminoethyl)-1,4,7-triaza-cyclononane (DETAN),^108−110^ and establish catalyst structure-reactivity-selectivity relationships; and (4) expand the methodology from methylenation (C=CH_2_ bond formation) into general olefination (C=CRR′ bond formation, where R and R′ are H, aryls, or alkyls) and systematically study the *E*/*Z* selectivity.

## Supporting Information Available

Synthesis details, NMR spectra, and crystallographic and computational details (PDF)
