# Supplementary material for: Li vs Na: Divergent Reaction Patterns between Organolithium and Organosodium Complexes and Ligand-Catalyzed Ketone/Aldehyde Methylenation
Source: J Am Chem Soc. 2023 Mar 8;145(11):6562–76. doi: 10.1021/jacs.3c01033 (PMC10037334; doi:10.1021/jacs.3c01033)
Supplement: Supplementary file 1 — ja3c01033_si_001.pdf [file ja3c01033_si_001.pdf]

Supplementary Materials for

Li *vs* Na: Divergent Reaction Patterns between Organolithium and Organosodium Complexes, and Ligand-catalyzed Ketone/Aldehyde Methylenation

Nathan Davison[1], Claire L. McMullin[2]*, Lu Zhang[3§], Shu–Xian Hu[3§], Paul G. Waddell[1], Corinne Wills[1], Casey Dixon[1], Erli Lu [1, 4*§]

Affiliations:

[1] Chemistry–School of Natural and Environmental Sciences, Newcastle University, Newcastle upon Tyne, NE1 7RU (UK).

[2] Department of Chemistry, University of Bath, Claverton Down, Bath, BA2 7AY (UK).

[3] School of Mathematics and Physics, University of Science and Technology Beijing, Beijing 100083, P. R. China.

[4] Lead author

*Corresponding authors: Erli.Lu@newcastle.ac.uk (E. L.); cm2025@bath.ac.uk (C. L. M.)

§ The authors' names spelt in simplified Chinese characters: L. Z. (张 璐); S. –X. H. (胡 淑贤), E. L. (陆 而立)

This PDF file includes:

Materials and Methods
Supplementary Text
Figures S1 to S70
Tables S1 to S9
Data S1

Section 1. Experimental methods and data--S2
Section 2. Computational details and data--S66
References---S104

Materials and Methods

Section 1. Experimental methods and data

1.1 General procedures

All manipulations were carried out using Schlenk techniques, or in a Vigor™ glovebox equipped with a −35 ˚C freezer and a cold well, under an atmosphere of dry argon. Benzene, toluene and *n*-hexane was dried with sodium press, sodium-potassium alloy and distilled under reduced pressure, and kept in the glovebox. Chemicals were purchased from Merck, Fluorochem or Alfa Aesar, and dried under dynamic vacuum for several hours (for solids), or over activated 4Å molecular sieves, prior to use.

All glassware, including pipettes, vials and ampoules, must be silylated prior to use by treating with trimethylsilyl chloride (Me_3SiCl), rinsing with water, and dried in a 150 °C oven for 12 hours. Failing to silylate glassware will lead to significantly reduced yields, and in some cases, irreproducible results.

Organosodium complexes are highly reactive towards silicone grease. We would suggest excluding grease as much as possible for all chemicals used herein, including in the solvent distillation apparatus; otherwise irreproducible byproducts or side products may appear. For this purpose, we used J. Young tap solvent flasks and vacuum transfer apparatus to dry our solvents.

1-Li was prepared as previously described [1]. $[NaCH_2SiMe_3]_\infty$ was prepared by a modified procedure of that described in the literature [2,3,4].

^1H and $^{13}C\{^1H\}$ spectra were recorded on a Bruker 300 Avance III spectrometer operating at 300.13 and 75.48 respectively. ^{23}Na spectra were recorded on a Bruker 500 Avance III HD spectrometer operating at 132 MHz. Kinetic ^1H NMR experiments were carried out on a Bruker 700 Avance III HD NMR spectrometer using a TCI cryoprobe operating at 700 MHz. Chemical shifts are quoted in ppm and are relative to $SiMe_4$ (^1H and ^{13}C) or external 0.1 M NaCl in D_2O (^{23}Na) or external 0.1 M LiCl in D_2O (^7Li).

1.2 Synthesis and characterization of $[NaCH_2SiMe_3]_\infty$

$[LiCH_2SiMe_3]_\infty$ (0.9416 g, 10 mmol) was dissolved in n-hexane (10 ml). At room temperature, the $LiCH_2SiMe_3$ - *n*-hexane solution was added to a suspension of NaO^tBu (0.9610 g, 10 mmol) in *n*-hexane (50 ml) with stirring in 2 ml portions. The resulting mixture was stirred at room temperature for 18 hours, during the course a white precipitate ($[NaCH_2SiMe_3]_\infty$) formed. The solid was isolated via centrifuging, which was then washed by 8 mL of *n*-hexane at room temperature. The solid was collected and all volatiles were removed *in vacuo*, to afford a whilt solid as the final product (0.8438 g, 77% yield).

Note: Since the isolation of $[NaCH_2SiMe_3]_\infty$ from the by-product LiO^tBu relies on their different solubilities in *n*-hexane ($[NaCH_2SiMe_3]_\infty$: insoluble/poor-soluble; LiO^tBu: soluble), the volume of *n*-hexane is essential for obtaining pure $[NaCH_2SiMe_3]_\infty$ free from potential LiO^tBu contamination. In the scenario of deviating from the reaction scale described herein, we suggest readers to adjust the hexane volume proportionally to ensure the $[NaCH_2SiMe_3]_\infty$ purity.

$[NaCH_2SiMe_3]_\infty$ is sparingly soluble in d_6-benzene (C_6D_6) at room temperature, and readily soluble at elevated temperature. A mixture of approximately 10 mg of $[NaCH_2SiMe_3]_\infty$ in 0.5 mL of C_6D_6 was heated to 80 ˚C until fully dissolved within 30 minutes. The colorless solution was slowly cooled (in a course of 5-8 hours, stop heating but without removing from the oil bath) to afford colorless needle-shaped crystals, which are suitable for single-crystal X-ray diffraction study.

^{1}H NMR (300 MHz, d_6-benzene, 25 °C): δ (ppm) 0.15 (s, 9H, Si(CH_3)$_3$), -2.42 (s, 2H, NaCH_2Si).

^{23}Na NMR (132 MHz, d6-benzene, 25 °C): δ (ppm) 22.66

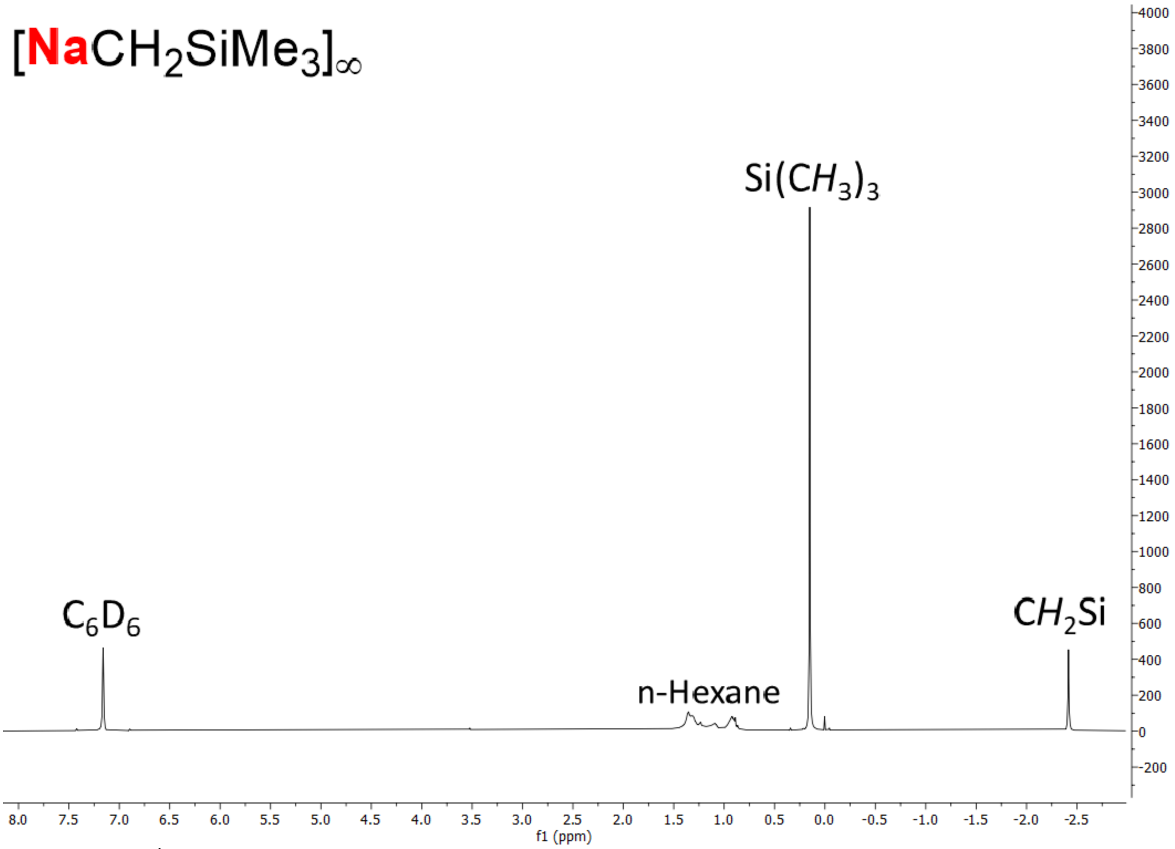

Figure S1: ^{1}H NMR (d_6-benzene, 25 ˚C, 300 MHz) of $[NaCH_2SiMe_3]_\infty$.

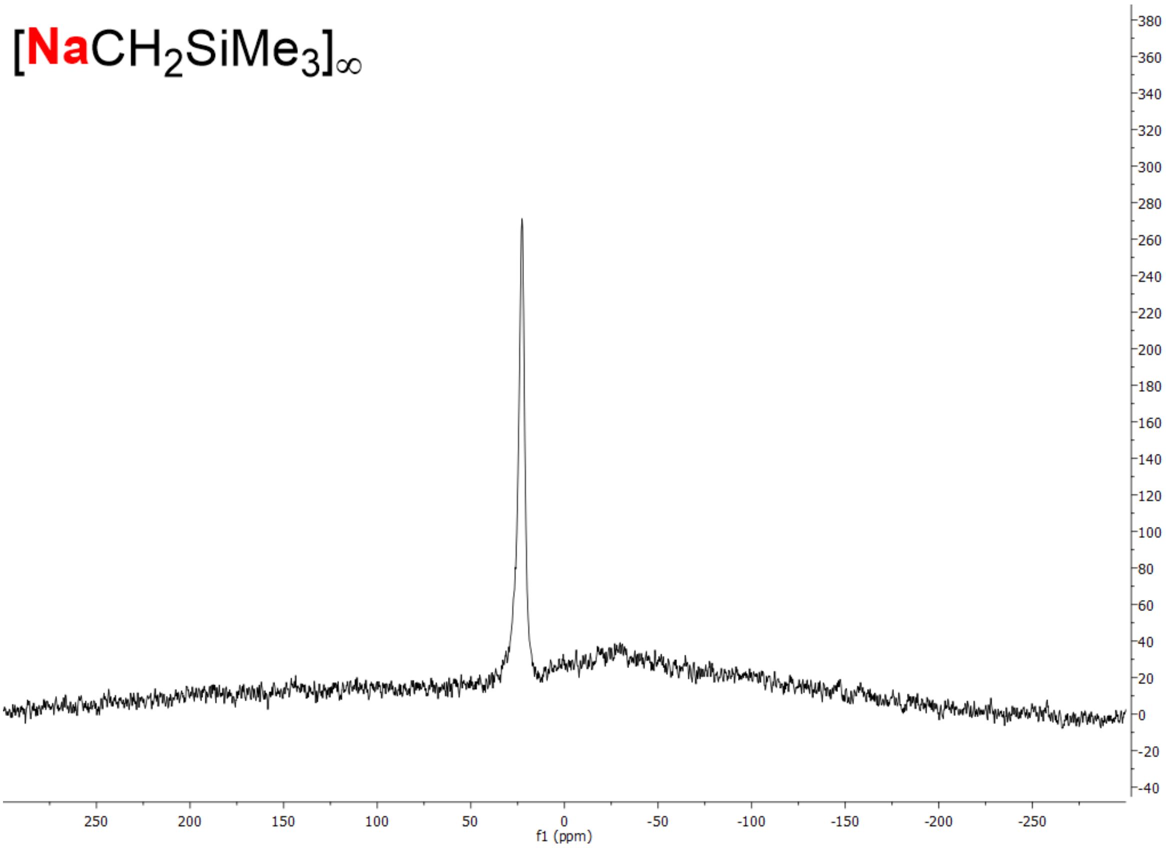

Figure S2: ^{23}Na NMR (d_6-benzene, 25 ˚C, 132 MHz) of $[NaCH_2SiMe_3]_\infty$.

1.3 Synthesis and characterization of [Na(CH_2SiMe_3)(Me_6Tren)] (**1**-Na)

Method A

[Li(CH_2SiMe_3)(Me_6Tren)] (**1**-Li) (0.3246 g, 1 mmol) was dissolved in *n*-hexane (1.5 ml). The resulting solution was added to a suspension of NaO^{t}Bu (0.0961, 1 mmol) in *n*-hexane (1 ml) at room temperature. After mixing, an extra portion of *n*-Hexane (6 ml) was added and the resulting solution was filtered through a pipette equipped with glass-wool plug and celite, the yellow clear solution was placed in a -35 ˚C freezer. Colourless crystals resulted overnight. The mother liquor was removed and the crystals dried *in vacuo*. Crystals suitable for SCXRD resulted from the mother liquor. NMR spectra taken of both matched. Due to their similar solubilities, the LiO^{t}Bu and [Na(CH_2SiMe_3)(Me_6Tren)] were unable to be completely separated and a signal in the ^{7}Li NMR was always present.

Method B

Me_6Tren (0.4608 g, 2 mmol) was dissolved in *n*-hexane (1 ml). The resulting solution was added in one-portion to a suspension of $[NaCH_2SiMe_3]_\infty$ (0.2204 g, 2 mmol) in *n*-hexane (4 ml) at room temperature. An extra portion of *n*-Hexane (15 ml) was added to the mixture and the resulting solution was filtered through a pipette equipped with glass-wool plug and celite and placed in a -35 ˚C freezer. Colourless crystals resulted overnight. The mother liquor was removed and the crystals were dried *in vacuo* (0.5145 g, 76 % yield).

NOTE: **Method B** is similar to the procedure reported by Hevia and co-workers very recently[4] but with higher thermal stability of **1**-Na herein. We also notice that Hevia and co-workers used two equivalents of Me_6Tren instead of one.

Isolated crystalline **1**-Na is soluble in C_6D_6 and d_{12}-cyclohexane (C_6D_{12}) at room temperature and is stable for 1-2 hours (C_6D_6) or 6-8 hours (C_6D_{12}).

^{1}H NMR (300 MHz, d_6-benzene, 25 °C): δ (ppm) 2.01 (s, 18H, N(CH_3)$_2$), 1.82 – 1.64 (m, 12H, NCH_2CH_2N), 0.64 (s, 9H, Si(CH_3)$_3$), -1.42 (s, 2H, NaCH_2Si).

^{1}H NMR (300 MHz, 25 °C, d_{12}-cyclohexane): δ (ppm) 2.44 – 2.36 (m, 6H, NCH_2CH_2N), 2.36 – 2.29 (m, 6H, NCH_2CH_2N), 2.24 (s, 18H, N(CH_3)$_2$), -0.15 (s, 9H, Si(CH_3)$_3$), -2.11 (s, 2H, NaCH_2Si).

^{13}C{^{1}H} NMR (75 MHz, d_6-benzene, 25 °C): δ (ppm) 56.7 (NCH_2CH$_2$N), 51.0 (NCH_2CH$_2$N), 45.1 (N(CH_3)$_2$), 7.6 (Si(CH_3)$_3$), −8.4 (NaCH$_2$Si).

^{23}Na NMR (132 MHz, d_6-benzene, 25 °C): δ (ppm) 25.15.

^{7}Li NMR (117 MHz, d_6-benzene, 25 °C): No signals, confirming no LiO^{t}Bu was remaining.

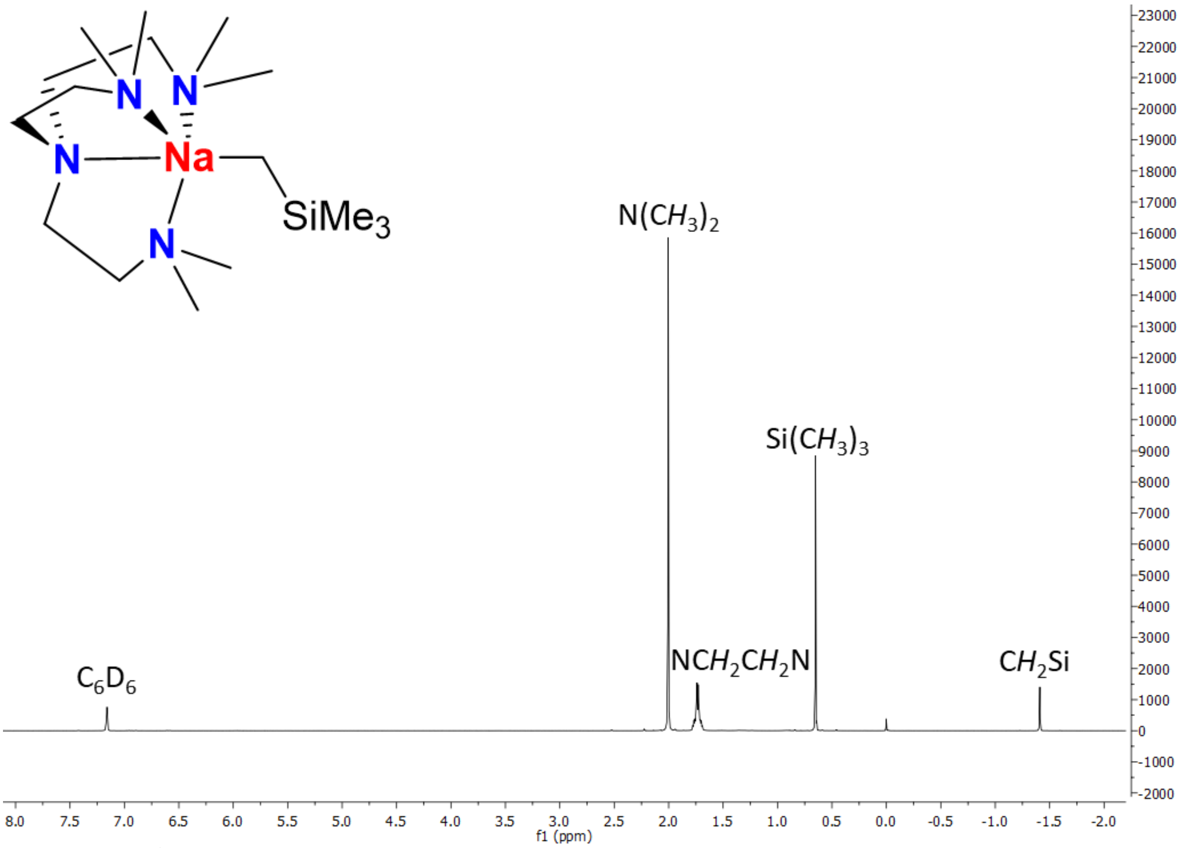

Figure S3: ^{1}H NMR (d_6-benzene, 25 ˚C, 300 MHz) of **1**-Na.

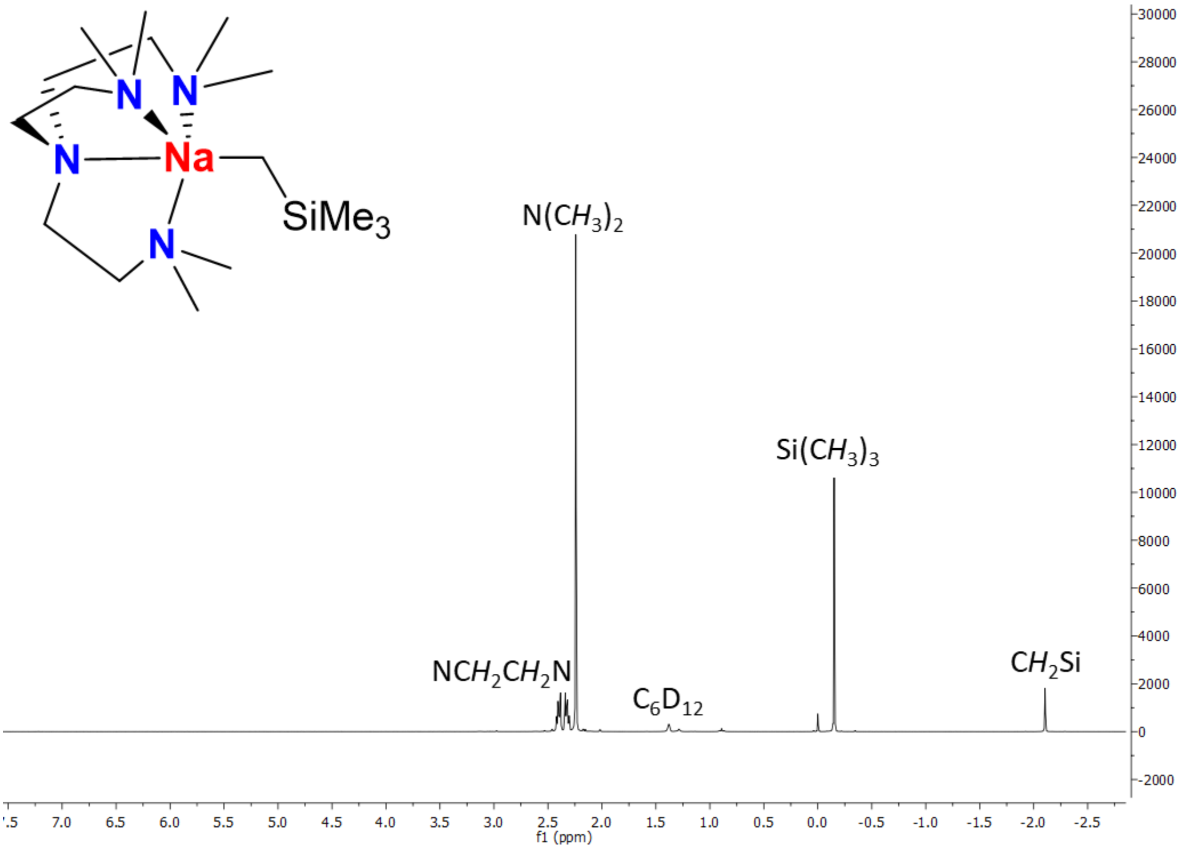

Figure S4: ^{1}H NMR (d_{12}-cyclohexane, 25 ˚C, 300 MHz) of **1**-Na.

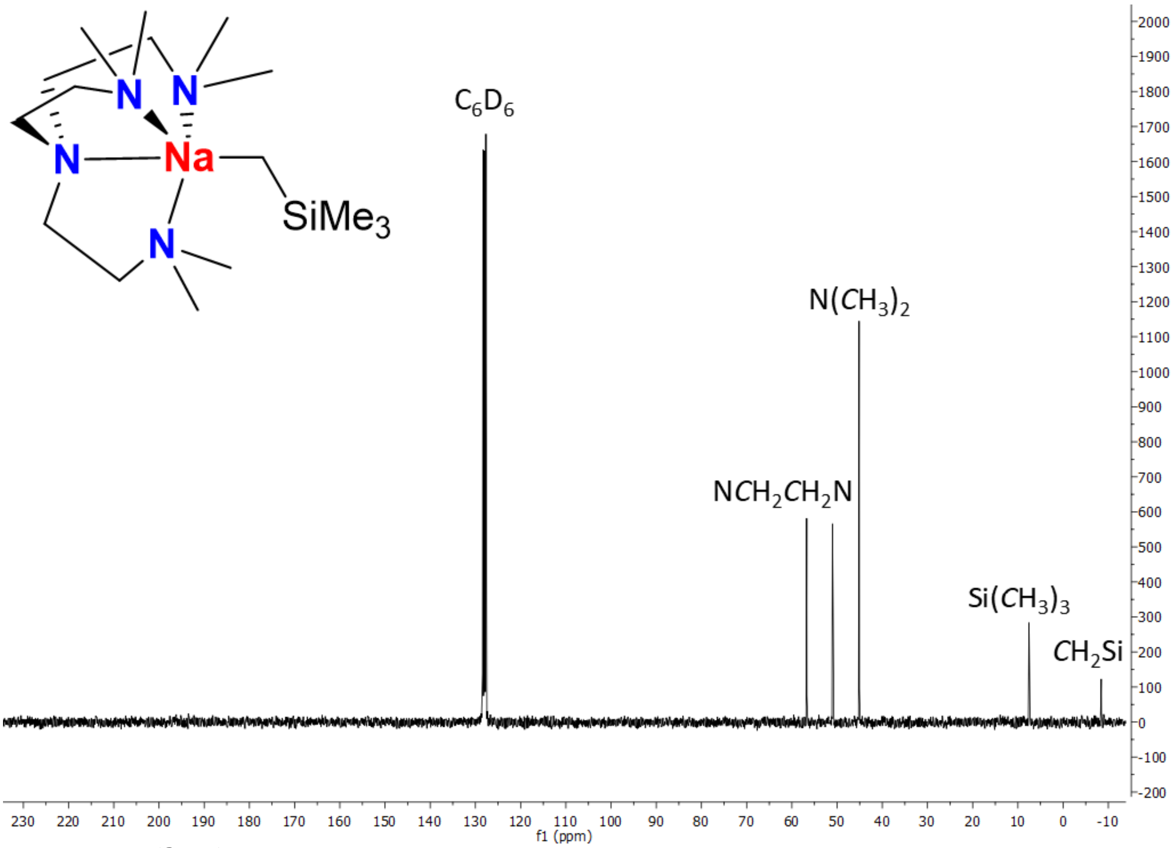

Figure S5: ^{13}C{^{1}H} NMR (d_6-benzene, 25 ˚C, 75 MHz) of **1**-Na.

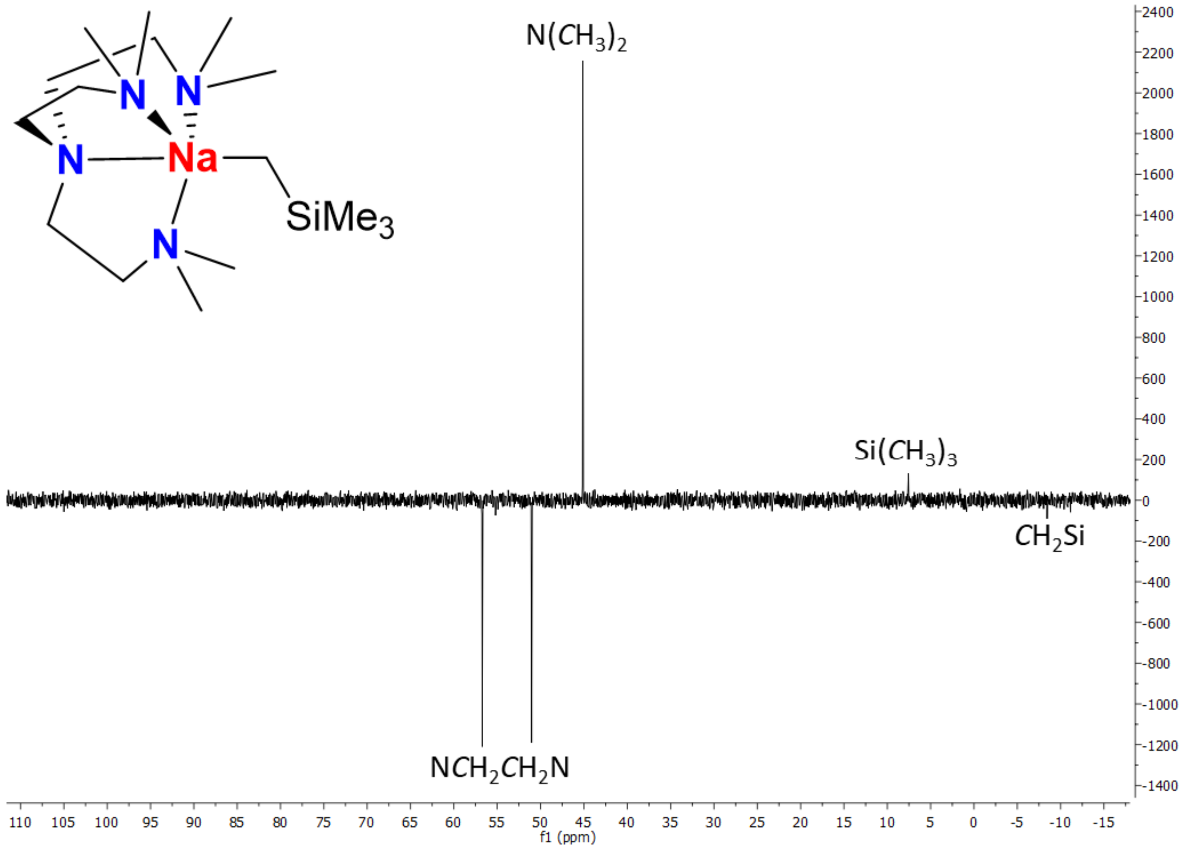

Figure S6: ^{13}C DEPT 135 NMR (d_6-benzene, 25 ˚C, 75 MHz) of **1**-Na.

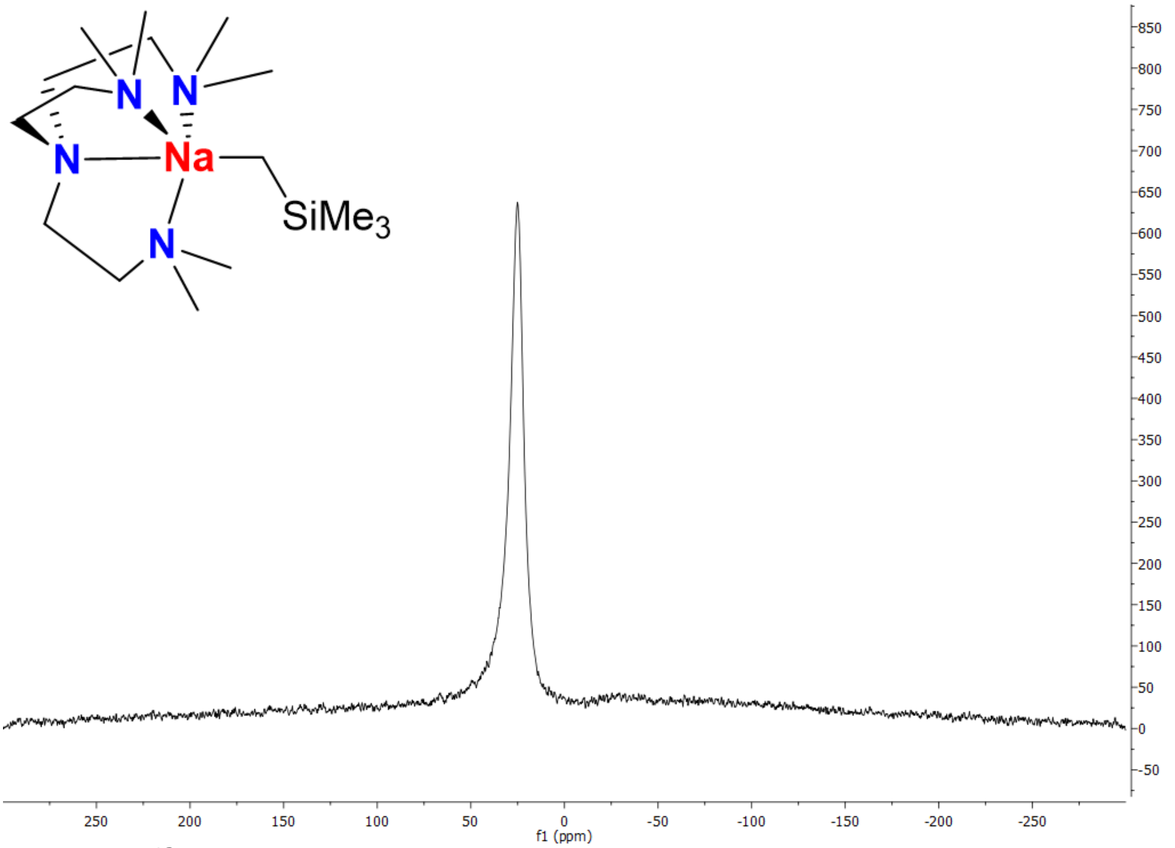

Figure S7: ^{23}Na NMR (d_6-benzene, 25 ˚C, 132 MHz) of **1**-Na.

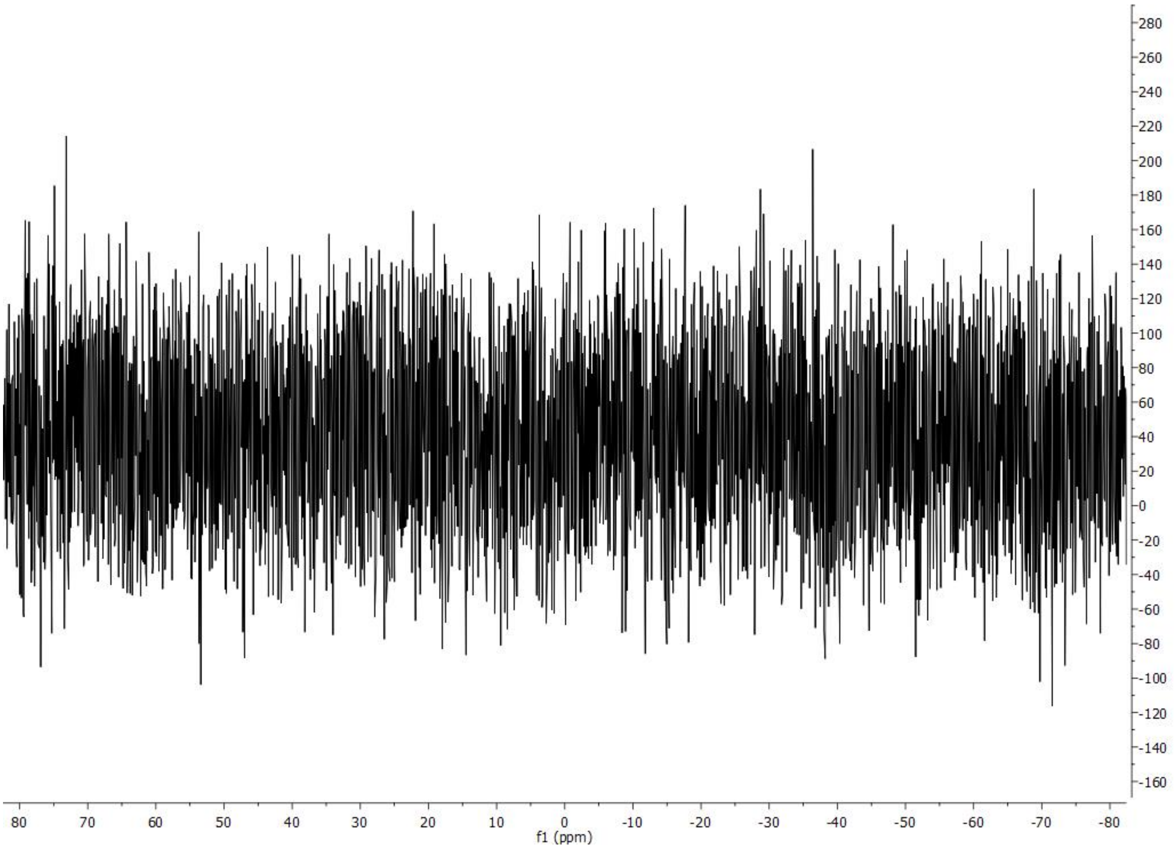

Figure S8: ^{7}Li NMR (d_6-benzene, 25 ˚C, 117 MHz) of **1**-Na showing no Li is present.

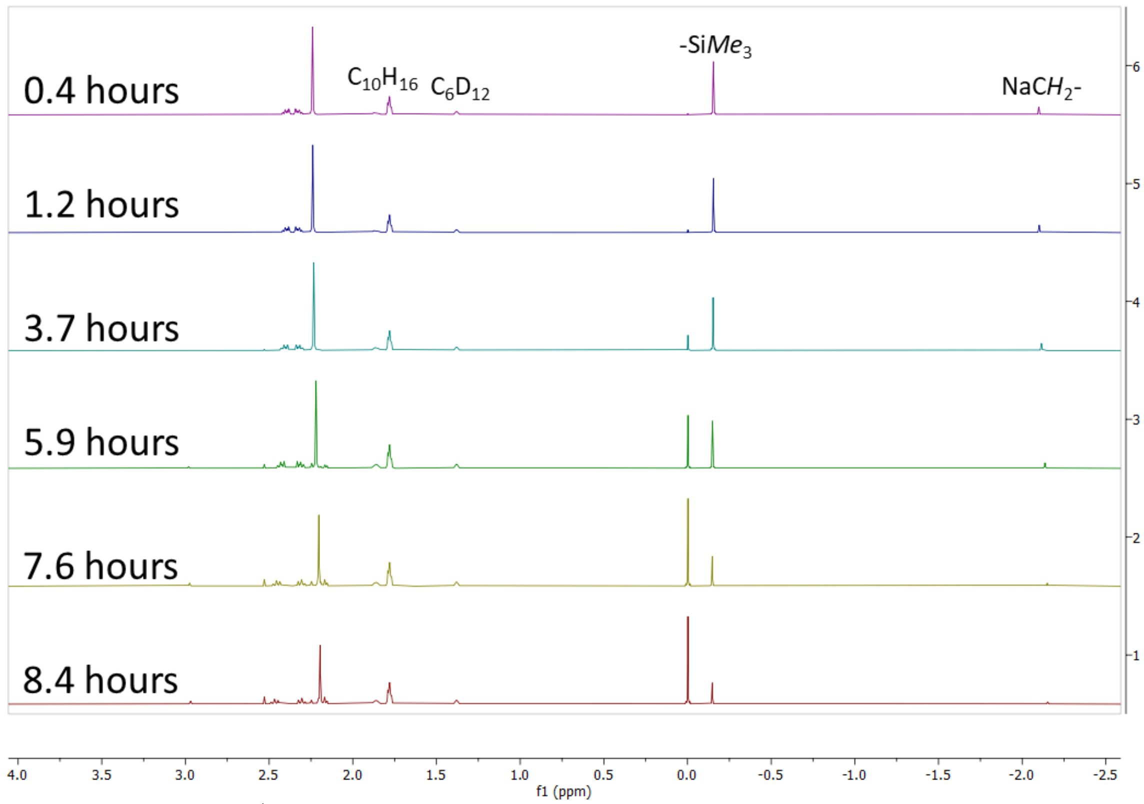

Figure S9: Stacked ^{1}H NMR (d_{12}-cyclohexane, 25 ˚C, 300 MHz) showing a stability test of **1**-Na (0.03 mmol) in C_6D_{12} (0.5 ml) over multiple hours at room temperature using adamantane ($C_{10}H_{16}$, 0.03 mmol) as an internal standard.

1.4 Stoichiometric reactions between **1**-Na and organic carbonyl substrates

Olefination of benzophenone (1a) to 1,1-diphenylethylene (4a)

NMR Scale Reaction:

1-Na (0.0102 g, 0.03 mmol) was dissolved in C_6D_6 (0.5 ml). The solution was added to benzophenone (0.0055g, 0.03 mmol) and the resulting solution was transferred to a J. Young NMR tube. The reaction was monitored by ^{1}H NMR after 15 minutes and 3 hours.

Scale up:

1-Na (0.3406 g, 1 mmol) was dissolved in benzene (1.5 ml). The solution was added to a solution of benzophenone (0.1822 g, 1 mmol) in benzene (0.5 ml) at room temperature. After 30 minutes, colorless block-shaped crystals were obtained and were proved to be **5** by SCXRD study. The mother liquor was removed and the volatiles removed *in vacuo*. An oil resulted, which ^{1}H and ^{13}C NMR revealed to be a mixture of 1,1-diphenylethylene and Me_6Tren. The crystals of **5** were washed with benzene (1 ml) and dried in vacuo (0.0769 g).

Data for 1,1-diphenylethylene (**4a**):

^{1}H NMR (300 MHz, d_6-benzene, 25 °C): δ (ppm) 7.34 – 7.27 (m, 4H, Ar*H*), 7.14 – 7.07 (m, 6H, Ar*H*), 5.36 (s, 2H, C=CH_2).

$^{13}C\{^1H\}$ NMR (75 MHz, d_6-benzene, 25 °C): δ (ppm) 150.7 (quaternary carbon), 142.0 (quaternary carbon), 128.6 (Ar*C*H), 128.5 (Ar*C*H), 127.9 (Ar*C*H), 114.2 (=CH_2).

NMR data are consistent with the literature[5] and a commercial sample of 1,1-diphenylethylene.

Data for $[Na_9(OSiMe_3)_{10}]^-[Na(C_6H_6)(Me_6Tren)]^+$ (**5**):
^1H NMR (300 MHz, d_6-benzene, 25 °C): δ (ppm) 7.16 (m, 6H, C_6H_6), 2.66 (m, 6H, NCH_2CH_2N), 2.40 (m, 6H, NCH_2CH_2N), 2.13 (s, 18H, $N(CH_3)_2$), 0.17 (s, 90H, $Si(CH_3)_3$).

$^{13}C\{^1H\}$ NMR (75 MHz, d_6-benzene, 25 °C): δ (ppm) 128.0 (C_6H_6), 58.7 (NCH_2CH_2N), 53.9 (NCH_2CH_2N), 46.1 ($N(CH_3)_2$), 4.7 ($Si(CH_3)_3$).

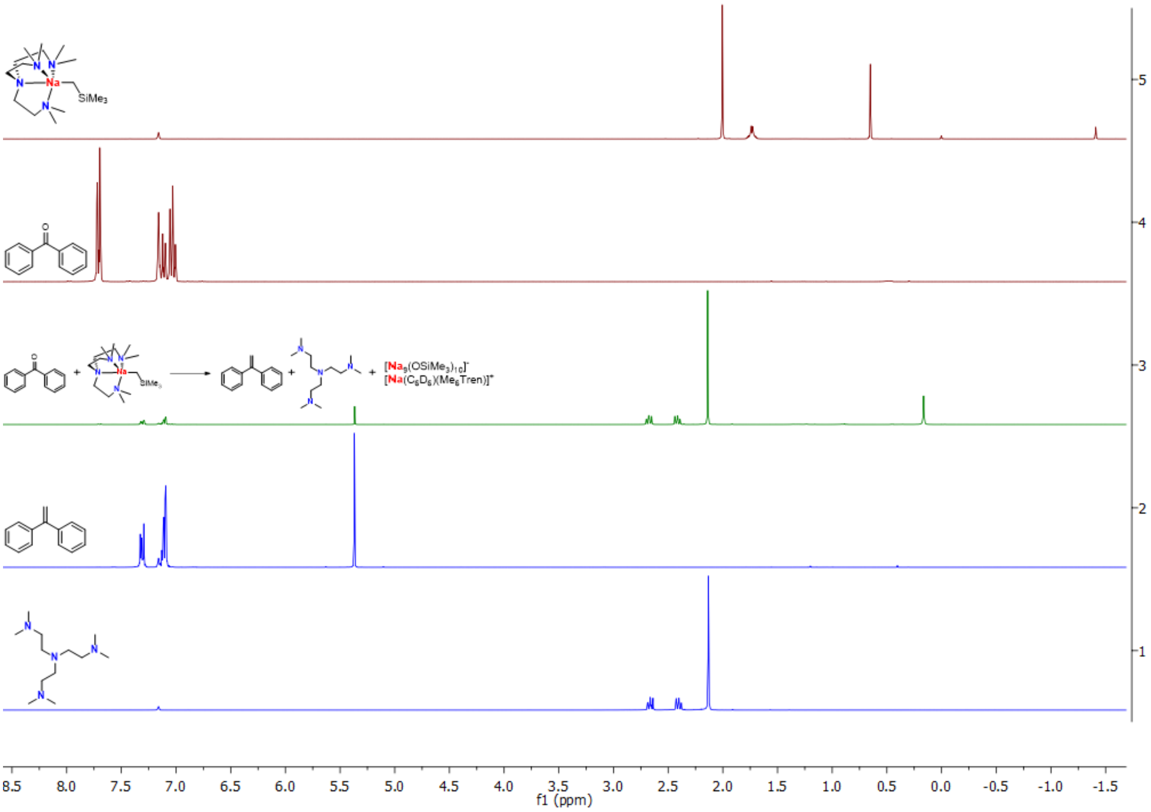

Figure S10: Stacked ^1H NMR (d_6-benzene, 25 ˚C, 300 MHz) of an NMR scale reaction between **1**-Na and benzophenone (middle: green); the starting materials (top: red); and authenticated samples of 1,1-diphenylethylene and Me_6Tren (bottom: blue).

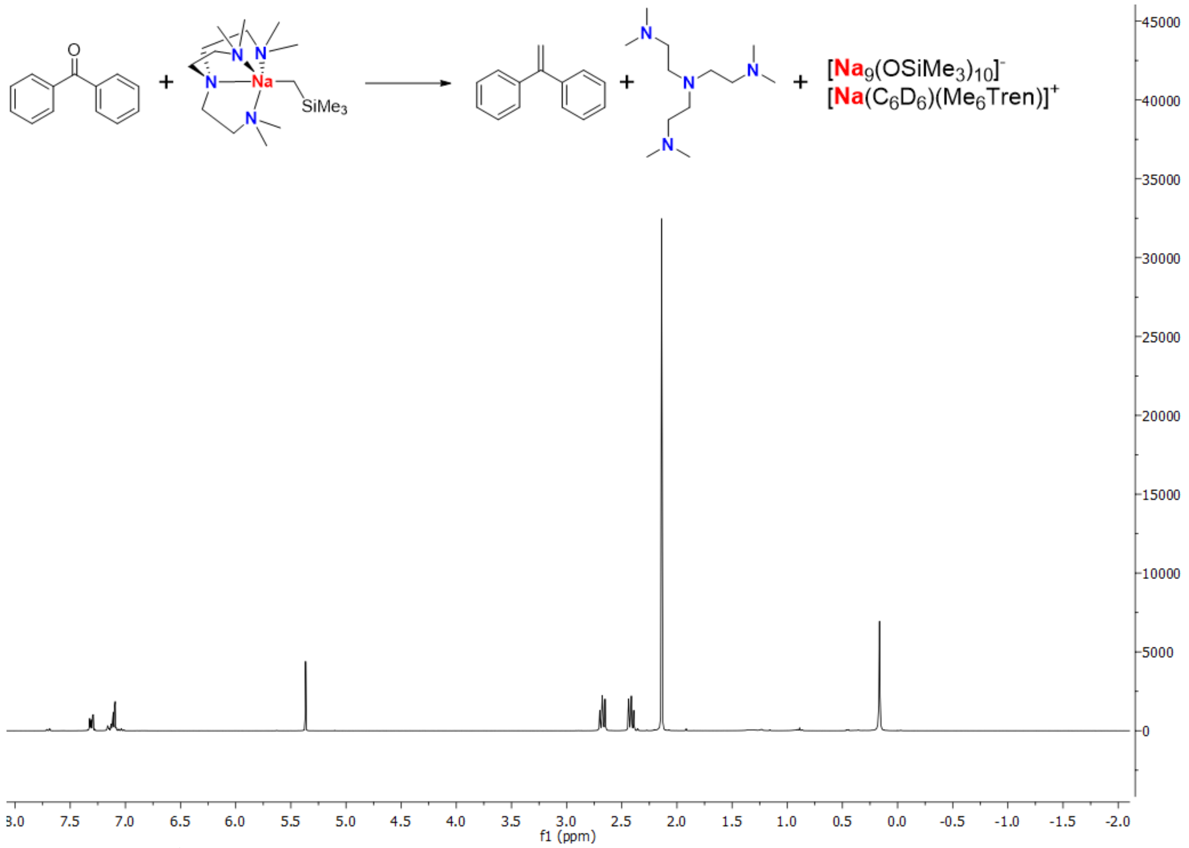

Figure S11: ^{1}H NMR (d_6-benzene, 25 ˚C, 300 MHz) of a NMR scale reaction between **1**-Na and benzophenone.

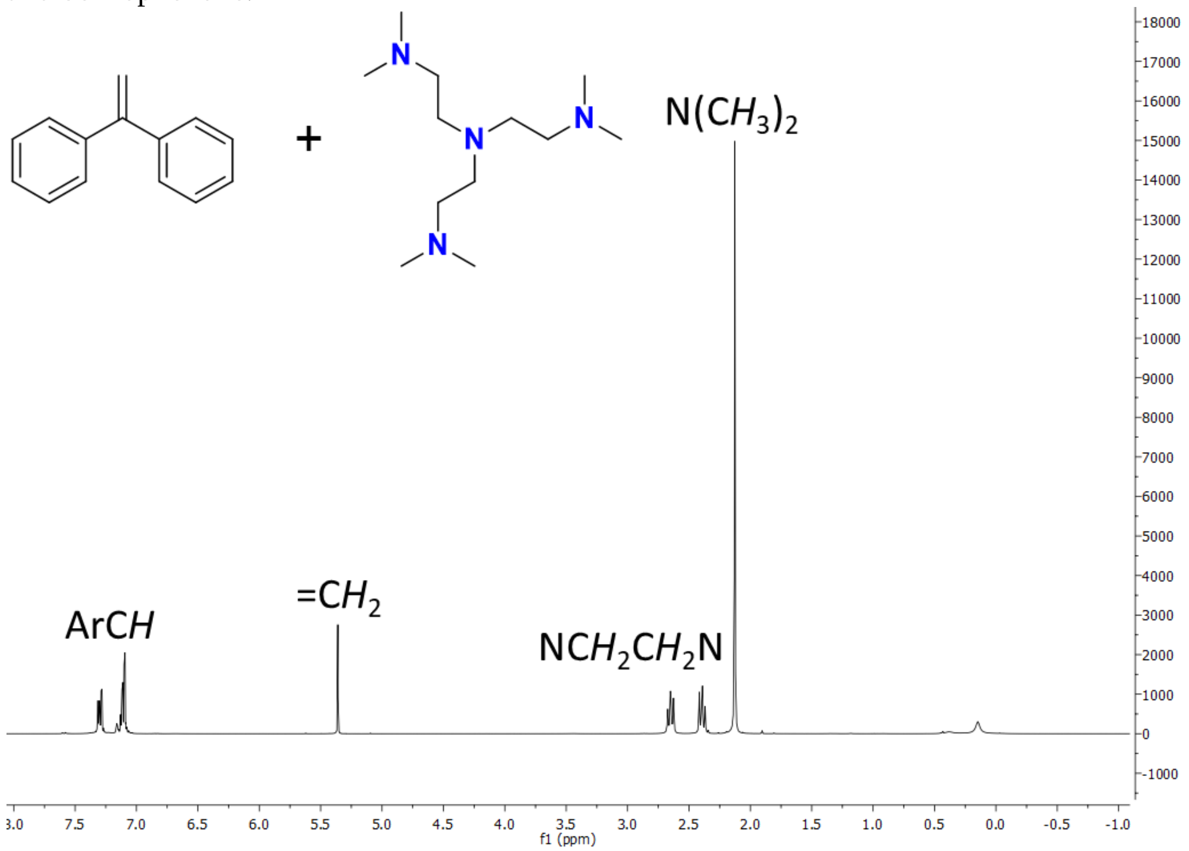

Figure S12: ^{1}H NMR (d_6-benzene, 25 ˚C, 300 MHz) of a mixture of 1,1-diphenylethylene and Me_6Tren from a scale-up reaction between **1**-Na and benzophenone.

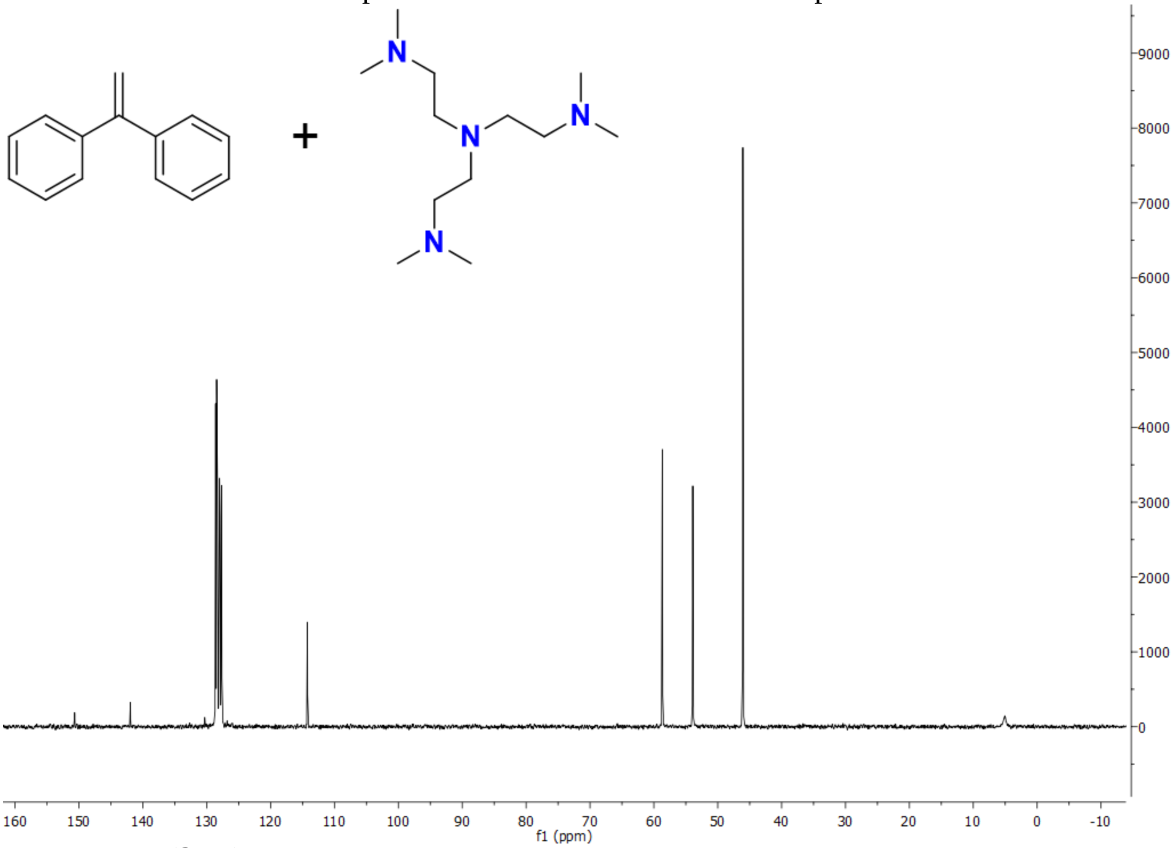

Figure S13: $^{13}C\{^{1}H\}$ NMR (d_6-benzene, 25 ˚C, 75 MHz) of a mixture of 1,1-diphenylethylene and Me_6Tren from a scale-up reaction between **1**-Na and benzophenone.

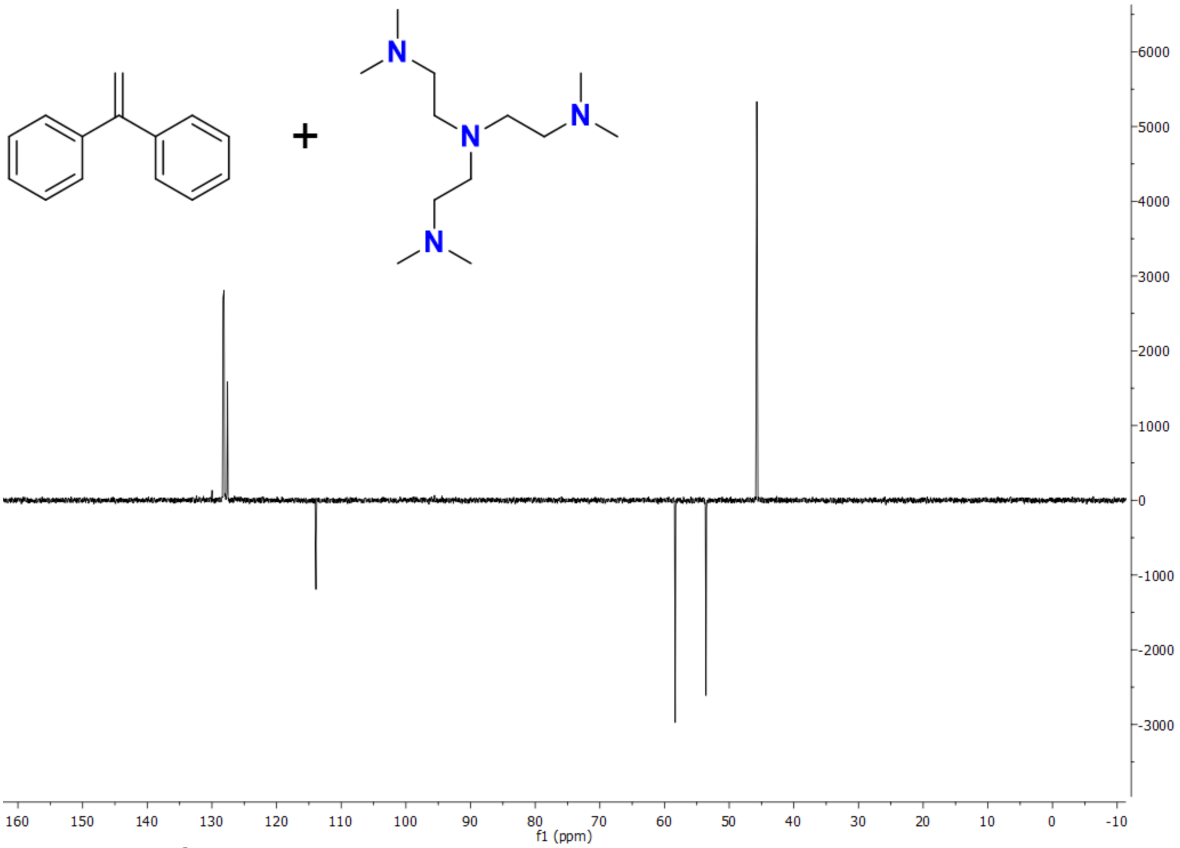

Figure S14: ^{13}C DEPT 135 NMR (d_6-benzene, 25 °C, 75 MHz) of a mixture of 1,1-diphenylethylene and Me_6Tren from a scale-up reaction between **1**-Na and benzophenone.

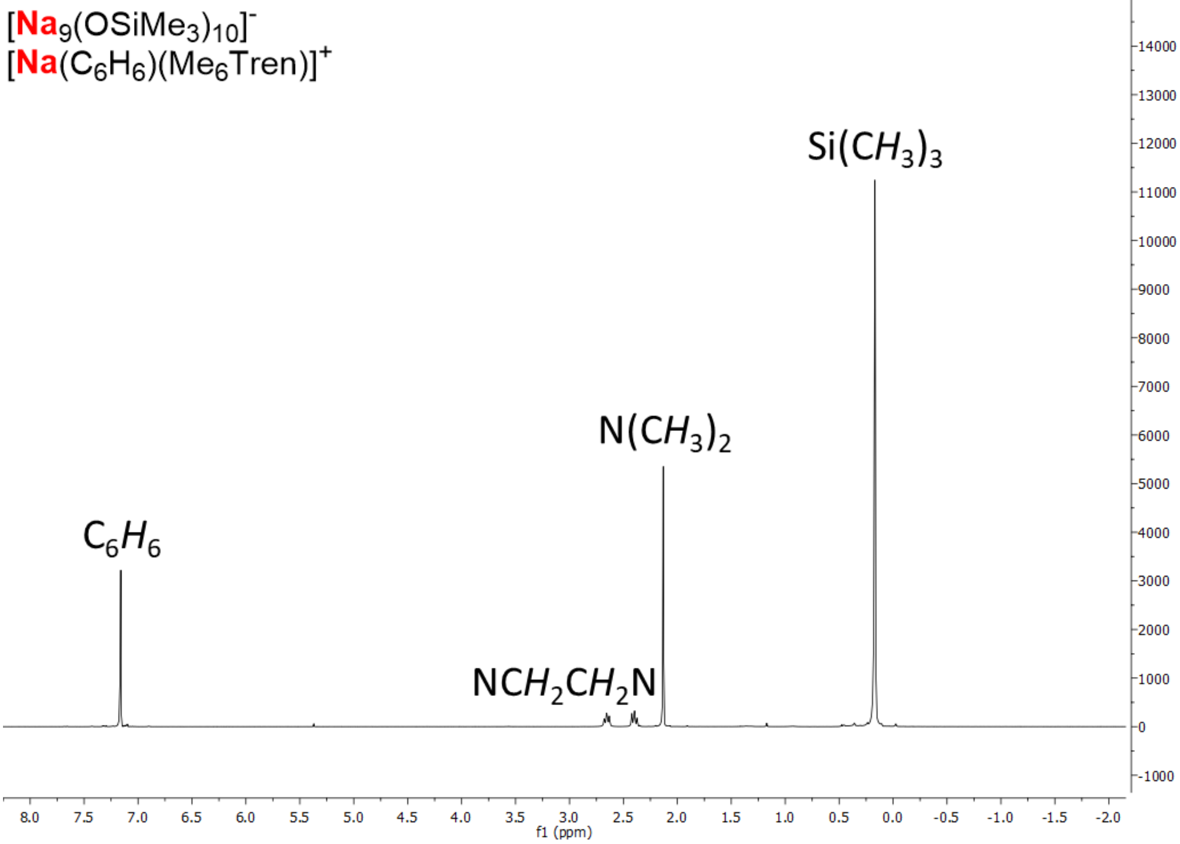

Figure S15: ^{1}H NMR (d_6-benzene, 25 ˚C, 300 MHz) of a **5**.

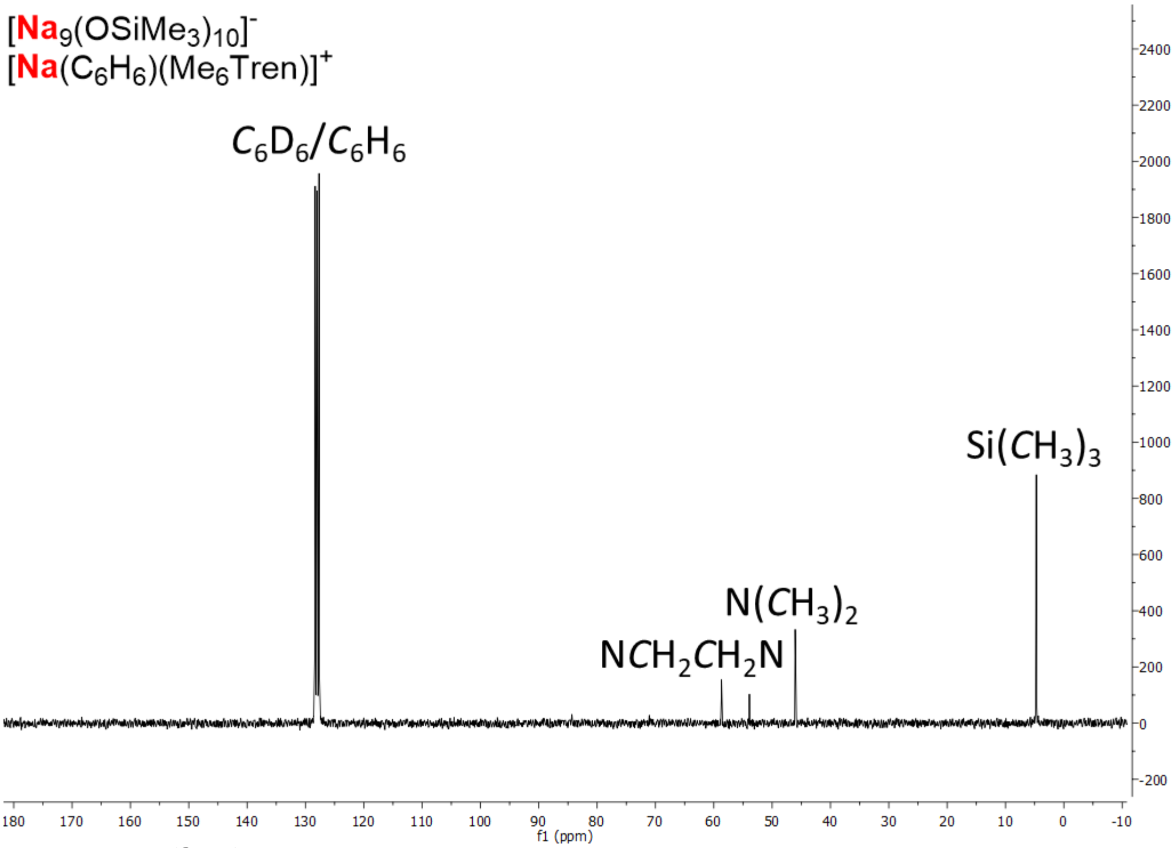

Figure S16: ^{13}C{^{1}H} NMR (d_6-benzene, 25 ˚C, 75 MHz) of a **5**.

Enolization of acetophenone (2b)

1-Na (0.0102 g, 0.03 mmol) was dissolved in C_6D_6 (0.5 ml). The solution was added to acetophenone (**2b**) (0.0036g, 0.03 mmol) and the resulting cloudy solution was transferred to a J Young NMR tube. The reaction was monitored by ^{1}H NMR after 15 minutes. Insolubles were present in the NMR tube.

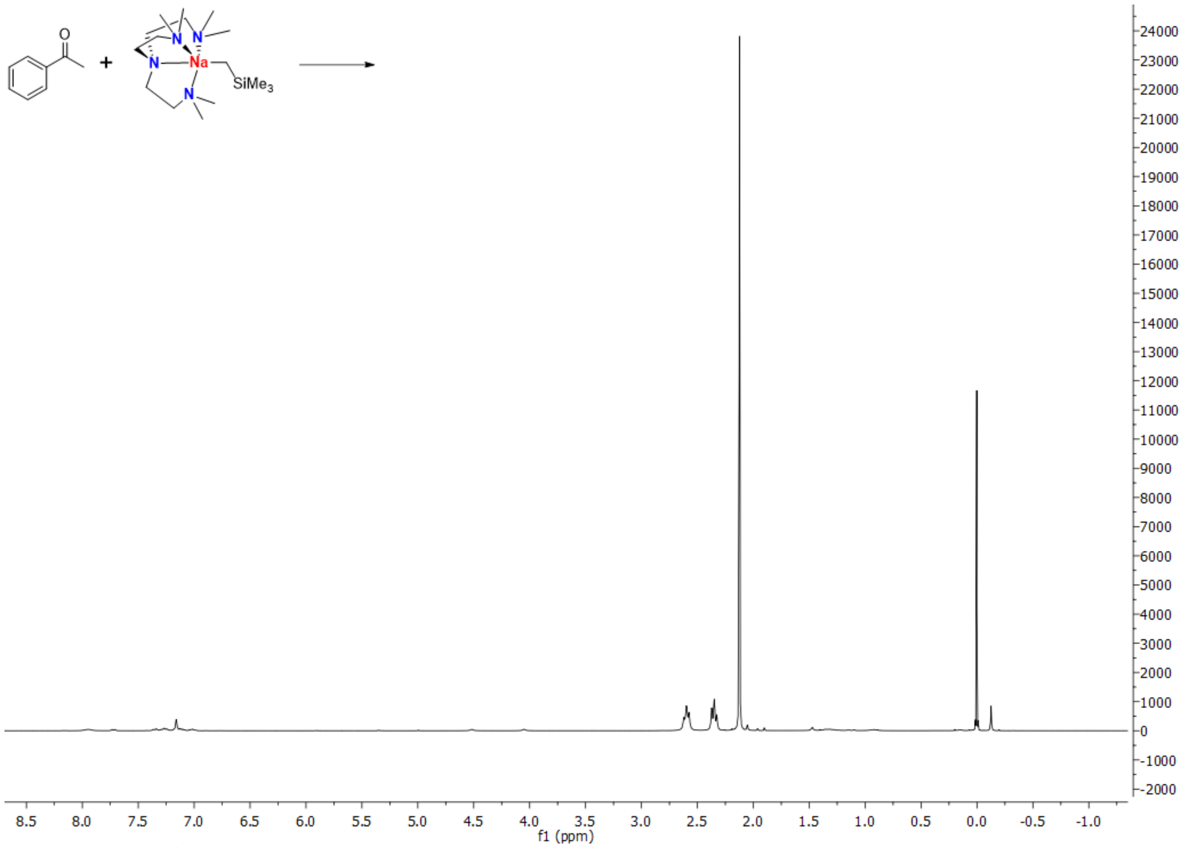

Figure S17: ^{1}H NMR (d_6-benzene, 25 ˚C, 300 MHz) of a NMR scale reaction between **1**-Na and acetophenone.

Olefination of dicyclohexyl ketone (2c) to 1,1-dicyclohexylethene
1-Na (0.0102 g, 0.03 mmol) was dissolved in C_6D_6 (0.5 ml). The solution was added to dicyclohexyl ketone (**2c**) (0.0058 g, 0.03 mmol) and the resulting solution was transferred to a J Young NMR tube. The reaction was monitored by ^{1}H NMR after 30 minutes.

Data for 1,1-dicyclohexylethene (**4c**):
^{1}H NMR (300 MHz, *d*$_6$-benzene, 25 °C): δ (ppm) 4.86 (s, 2H), 1.97 – 1.05 (m, 22H).

NMR data are consistent with the literature[6]

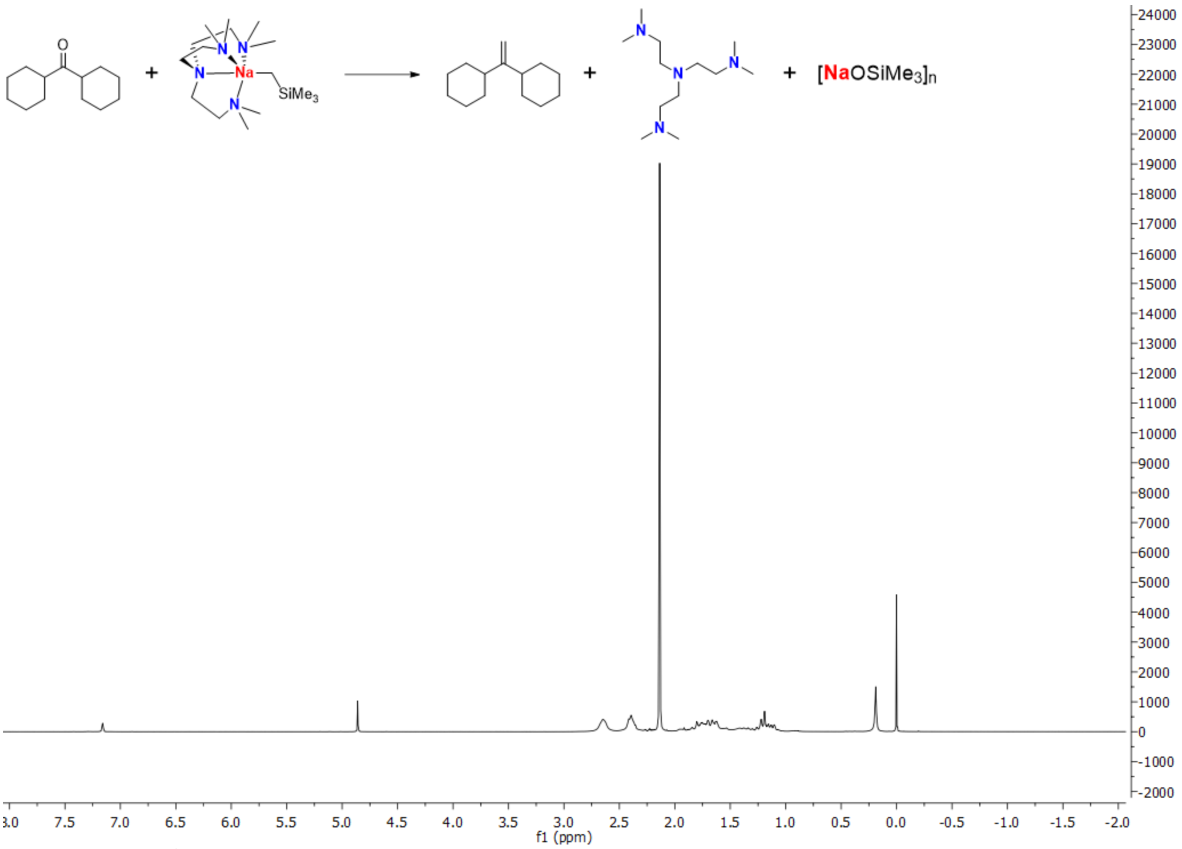

Figure S18: ^{1}H NMR (*d*$_6$-benzene, 25 ˚C, 300 MHz) of a NMR scale reaction between **1**-Na and dicyclohexyl ketone (**2c**).

Olefination of phenyl cyclohexyl ketone (2d) to 1-phenyl-1-cyclohexylethylene
1-Na (0.0102 g, 0.03 mmol) was dissolved in C_6D_6 (0.5 ml). The solution was added to phenyl cyclohexyl ketone (**2d**) (0.0056 g, 0.03 mmol) and the resulting solution was transferred to a J Young NMR tube. The reaction was monitored by ^{1}H NMR after 30 minutes.

Data for 1-phenyl-1-cyclohexylethylene (**4d**):
^{1}H NMR (300 MHz, d_6-benzene, 25 °C): δ (ppm) 7.34 – 7.27 (m, 2H, Ar*H*), 7.20 – 7.09 (m, 3H, Ar*H*) 5.20, (dd, *J* = 1.4, 0.6 Hz, 1H, C=C*H*$_2$), 5.01 (t, *J* = 1.4 Hz, 1H, C=C*H*$_2$), 2.47 – 2.33 (m, 1H, C*H*), 1.90 – 1.77 (m, 2H, C*H*$_2$), 1.76 – 1.46 (m, 3H, C*H*$_2$), 1.34 – 0.98 (m, 5H, C*H*$_2$).

^{13}C{^{1}H} NMR (75 MHz, d_6-benzene, 25 °C): *δ* (ppm) 155.3 (quaternary carbon), 143.3 (quaternary carbon), 128.4 (Ar*C*H), 127.3 (Ar*C*H), 127.0 (Ar*C*H), 110.5 (=*C*H$_2$), 42.9 (*C*H), 33.0 (*C*H$_2$), 27.1 (*C*H$_2$), 26.7 (*C*H$_2$).

NMR data are consistent with the literature[7].

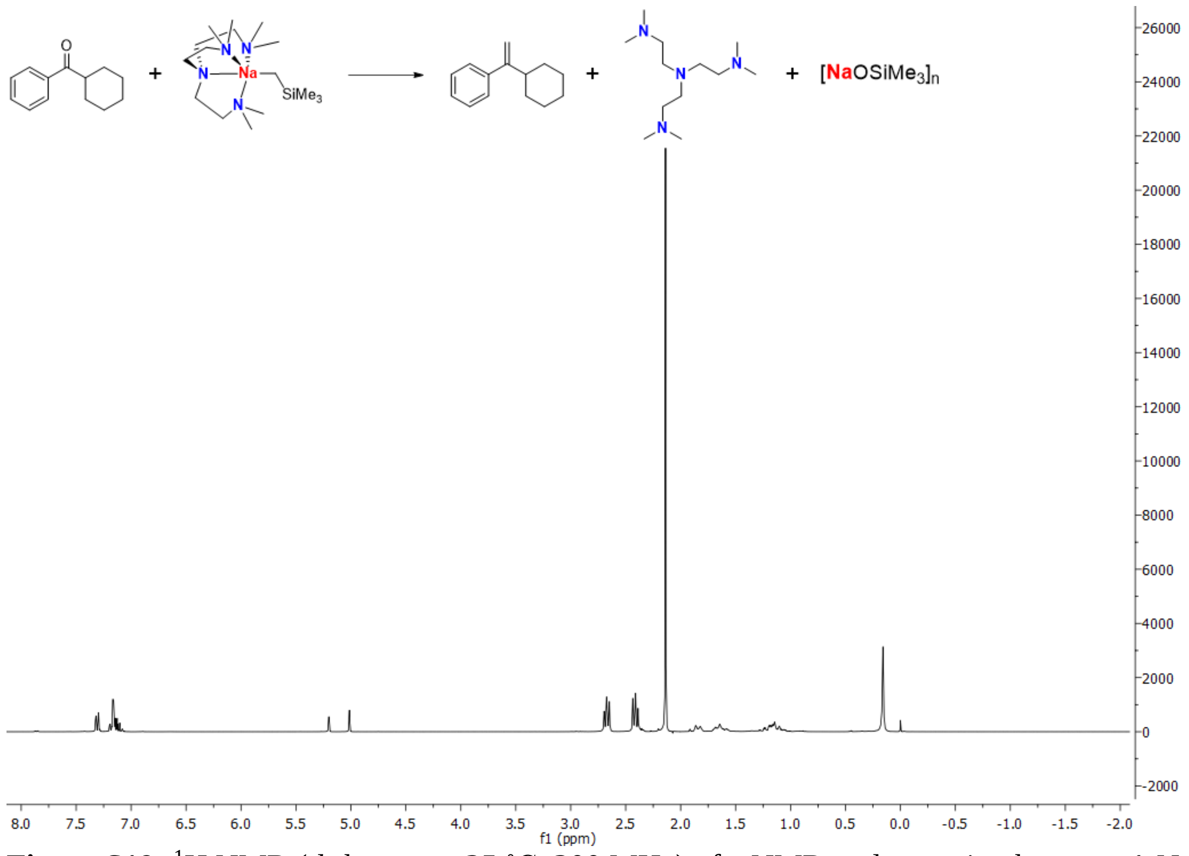

Figure S19: ^{1}H NMR (d_6-benzene, 25 ˚C, 300 MHz) of a NMR scale reaction between **1**-Na and phenyl cyclohexyl ketone.

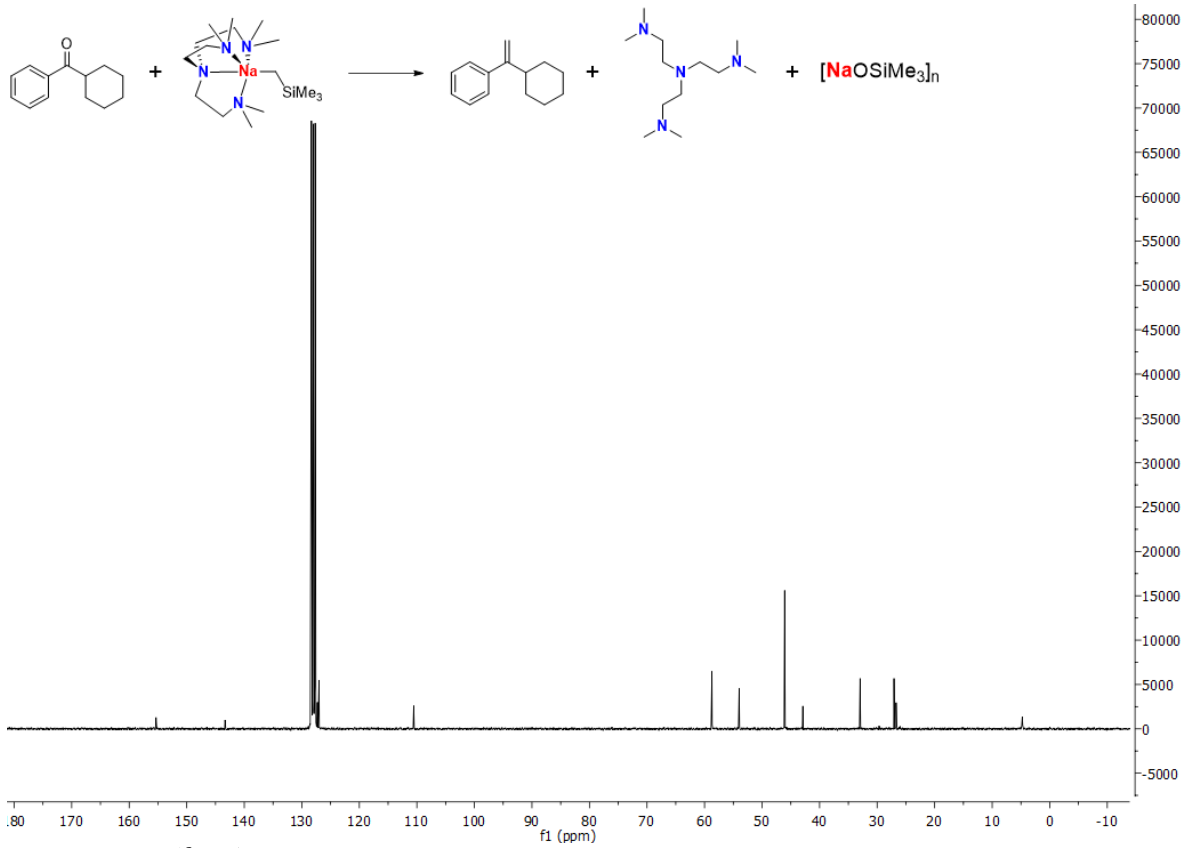

Figure S20: $^{13}C\{^{1}H\}$ NMR (d_6-benzene, 25 ˚C, 75 MHz) of a NMR scale reaction between **1**-Na and phenyl cyclohexyl ketone.

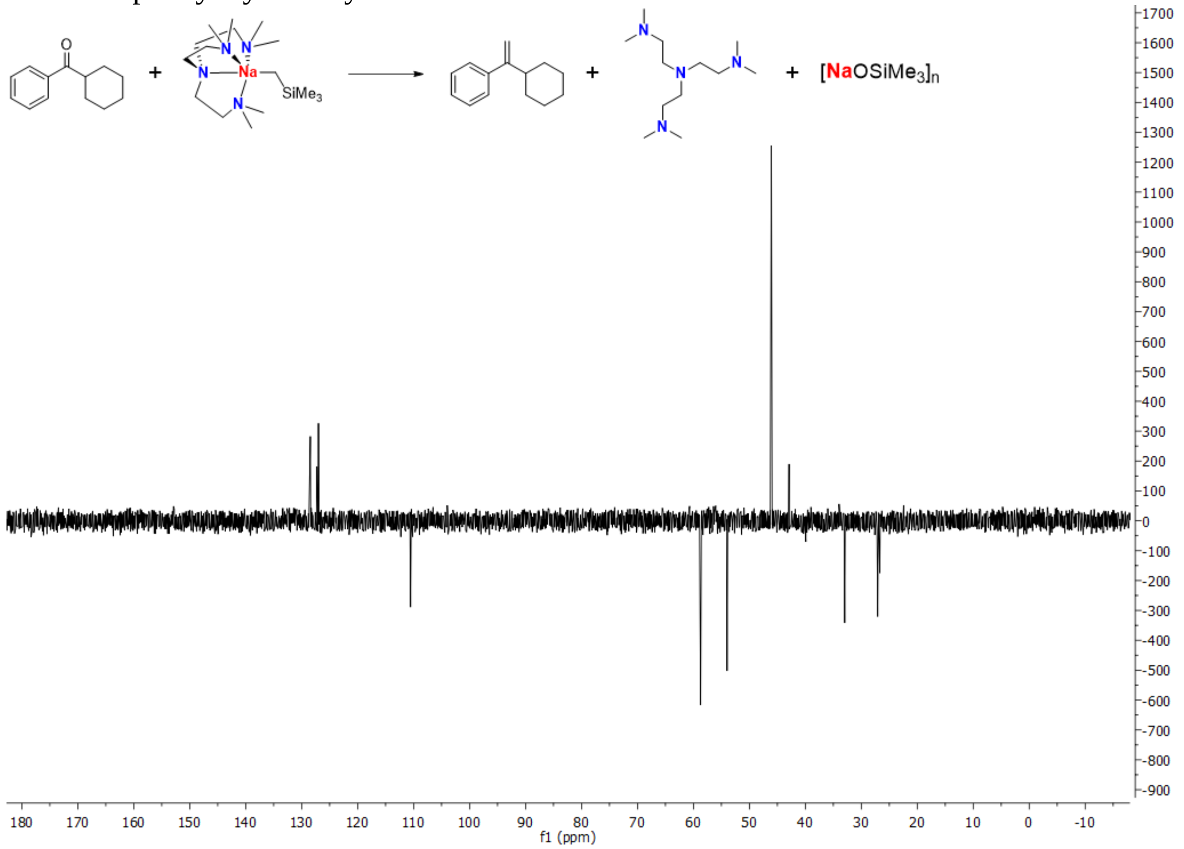

Figure S21: ^{13}C DEPT 135 NMR (d_6-benzene, 25 ˚C, 75 MHz) of a NMR scale reaction between **1**-Na and phenyl cyclohexyl ketone.

Olefination of 2,2,2-trimethylacetophenone (2e) to (3,3-dimethylbut-1-en-2-yl)benzene (4e)

1-Na (0.0102 g, 0.03 mmol) was dissolved in C_6D_6 (0.5 ml). The solution was added to 2,2,2-trimethylacetophenone (**2e**) (0.0049 g, 0.03 mmol) and the resulting solution was transferred to a J Young NMR tube. The reaction was monitored by ^{1}H NMR after 30 minutes.

Data for (3,3-dimethylbut-1-en-2-yl)benzene (**4e**):
^{1}H NMR (300 MHz, d_6-benzene, 25 °C): δ (ppm) 7.18 – 7.06 (m, 5H, Ar*H*), 5.16 (d, *J* = 1.7 Hz, 1H, C=CH_2), 4.85 (d, *J* = 1.7 Hz, 1H, C=CH_2), 1.08 (s, 9H, CH_3).

^{13}C{^{1}H} NMR (75 MHz, d_6-benzene, 25 °C): *δ* (ppm) 160.0 (quaternary carbon), 143.7 (quaternary carbon), 129.3 (Ar*C*H), 127.7 (Ar*C*H), 126.6 (Ar*C*H), 111.8 (=*C*H_2), 36.2 (quaternary carbon), 29.7 (*C*H_3).

NMR data are consistent with the literature[8].

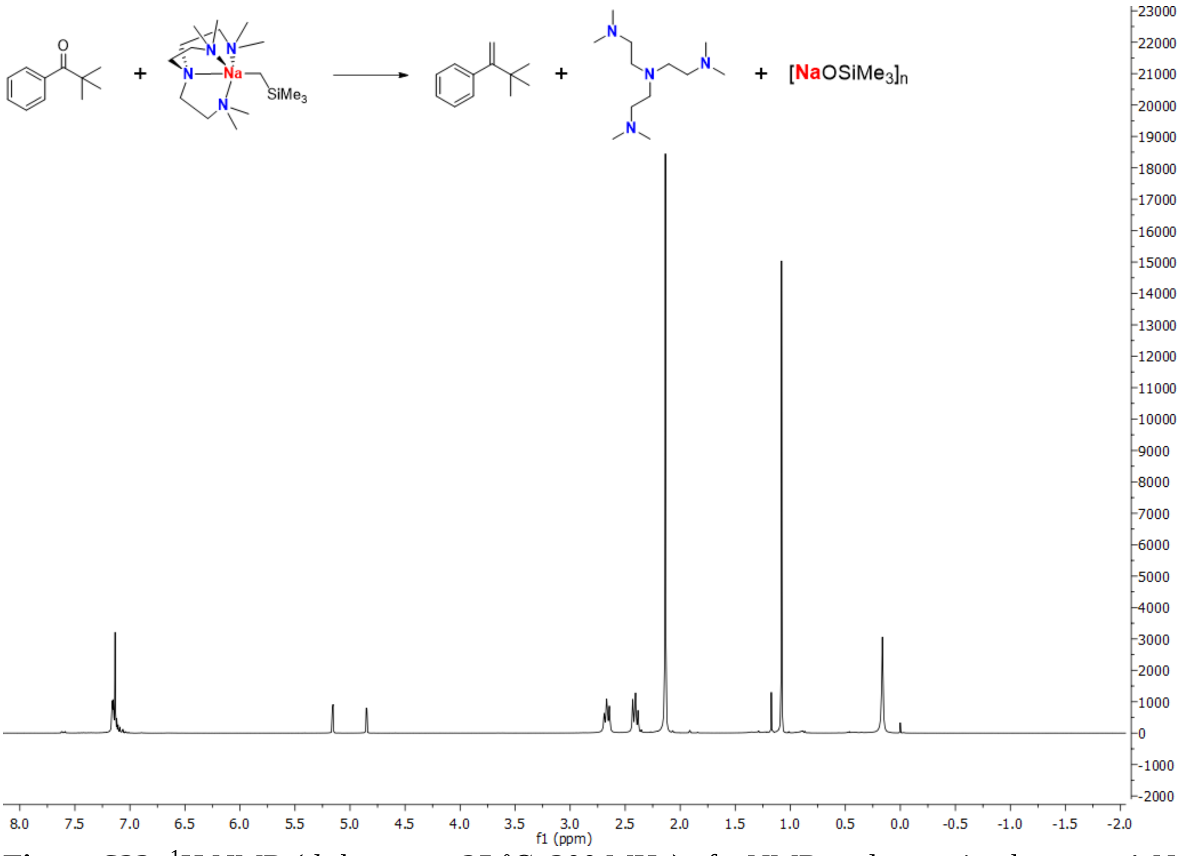

Figure S22: ^{1}H NMR (d_6-benzene, 25 ˚C, 300 MHz) of a NMR scale reaction between **1**-Na and 2,2,2-trimethylacetophenone.

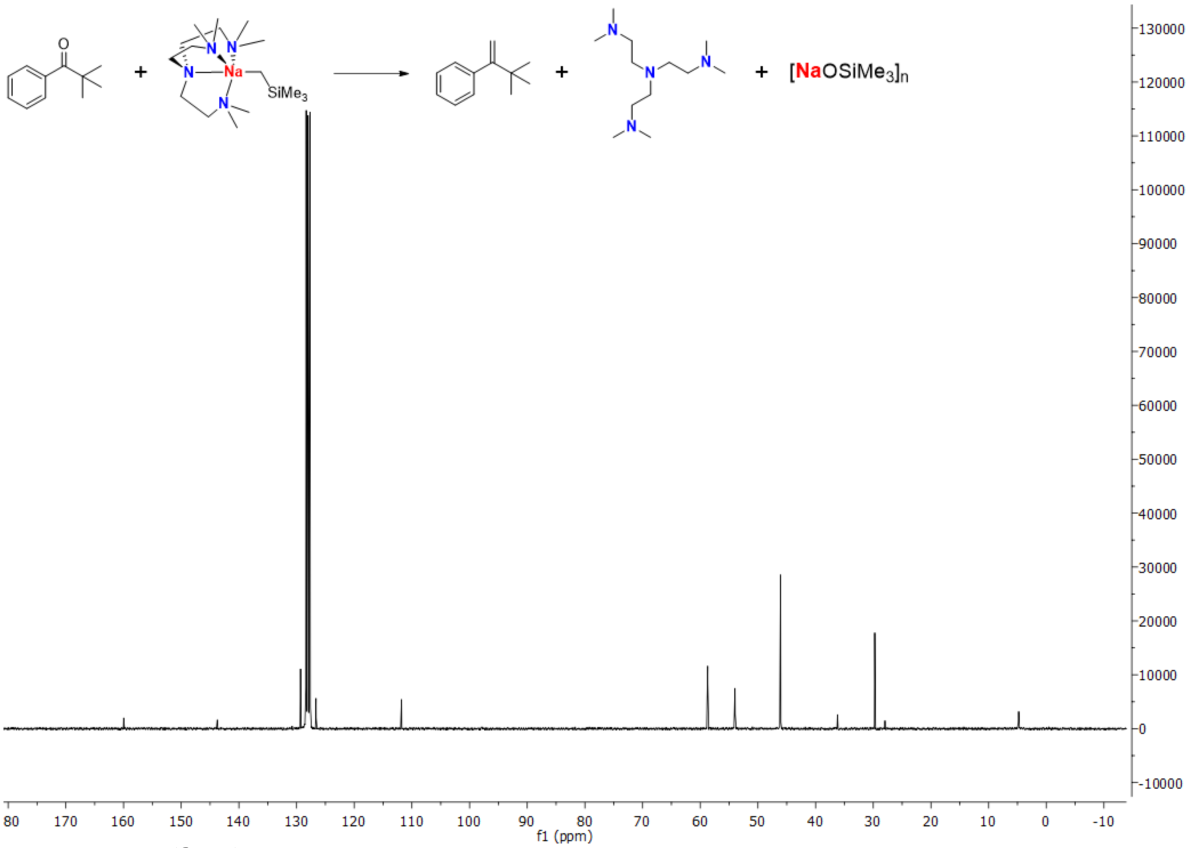

Figure S23: $^{13}C\{^{1}H\}$ NMR (d_6-benzene, 25 ˚C, 75 MHz) of a NMR scale reaction between **1**-Na and 2,2,2-trimethylacetophenone.

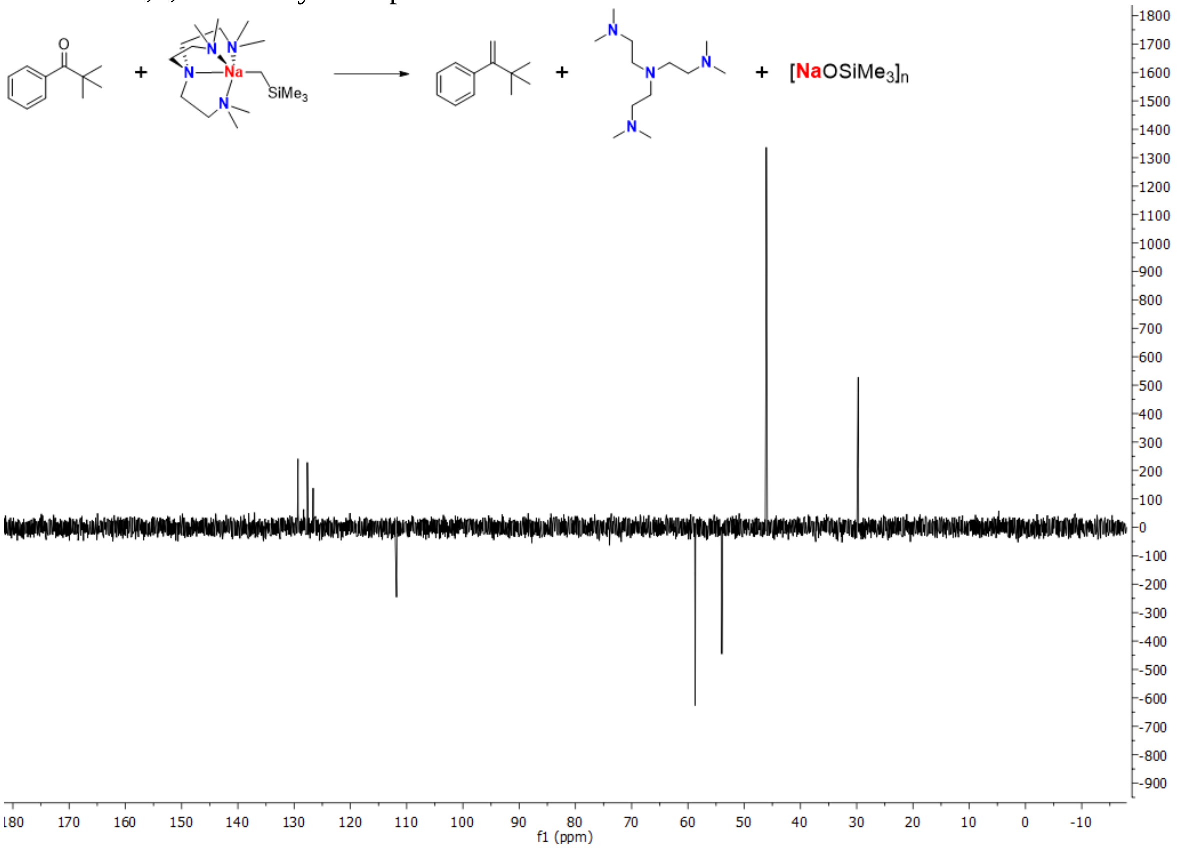

Figure S24: ^{13}C DEPT 135 NMR (d_6-benzene, 25 ˚C, 75 MHz) of a NMR scale reaction between **1**-Na and 2,2,2-trimethylacetophenone.

Olefination of 2-adamantanone (2f) to 2-methyleneadamantane (4f)

1-Na (0.0102 g, 0.03 mmol) was dissolved in C_6D_6 (0.5 ml). The solution was added to 2-adamantanone (**2f**) (0.0045 g, 0.03 mmol) and the resulting solution was transferred to a J Young NMR tube. The reaction was monitored by ^{1}H NMR after 30 minutes.

Data for 2-methyleneadamantane (**4f**):
^{1}H NMR (300 MHz, d_6-benzene, 25 °C): δ (ppm) 4.65 (s, 2H, C=C*H*$_2$), 2.46 (s, 2H, Ad*H*), 1.87 – 1.67 (m, 12H, Ad*H*)
.
^{13}C{^{1}H} NMR (75 MHz, d_6-benzene, 25 °C): *δ* (ppm) 157.8 (quaternary carbon), 101.4 (=*C*H$_2$), 39.8 (*C*H$_2$), 39.4 (*C*H), 37.5 (*C*H$_2$), 28.6 (*C*H).

NMR data are consistent with the literature[9].

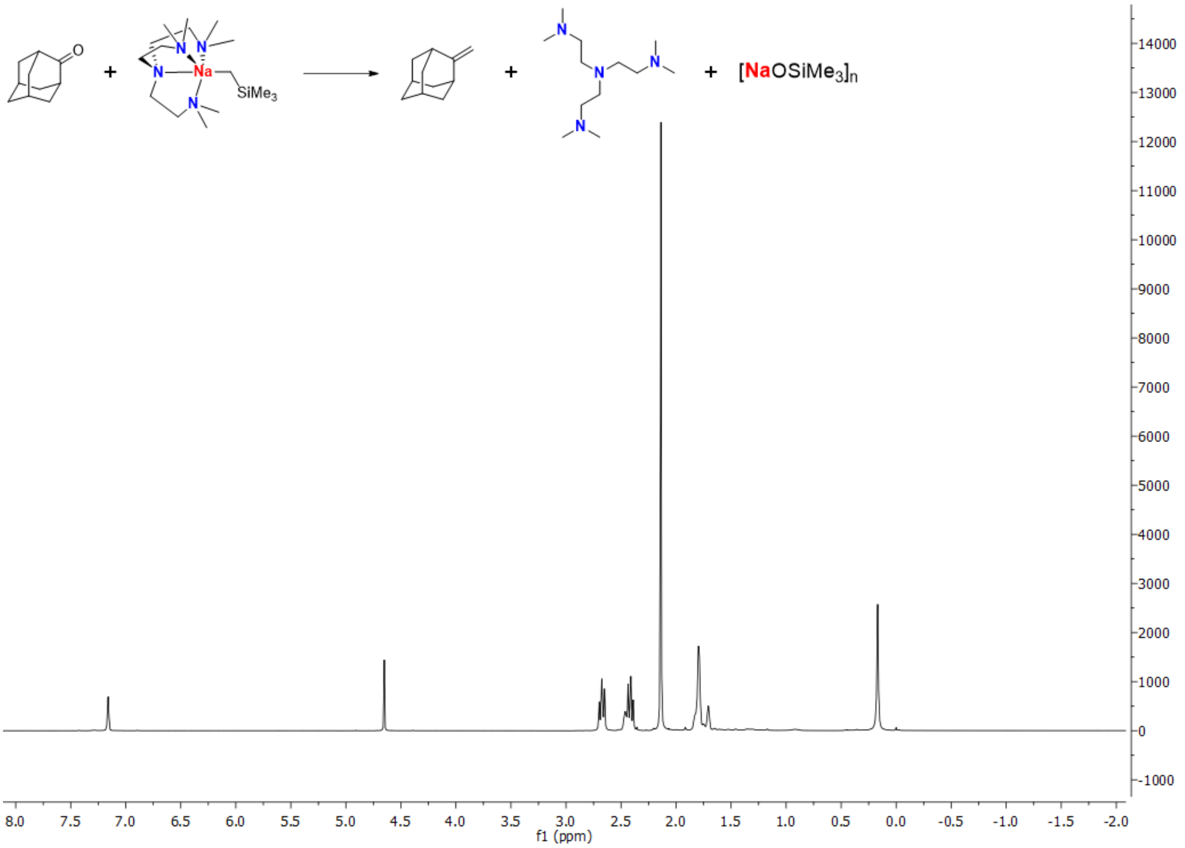

Figure S25: ^{1}H NMR (d_6-benzene, 25 ˚C, 300 MHz) of a NMR scale reaction between **1**-Na and 2-adamantanone.

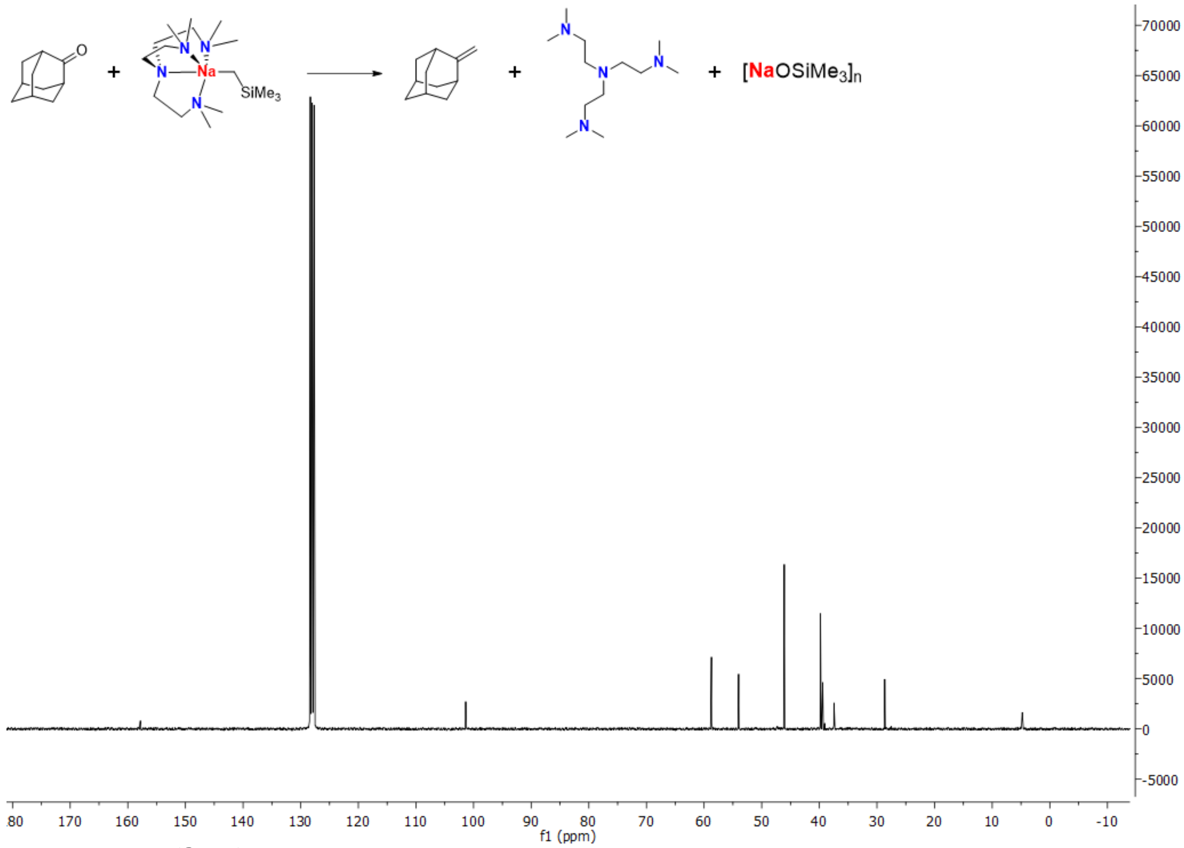

Figure S26: $^{13}C\{^1H\}$ NMR (d_6-benzene, 25 ˚C, 75 MHz) of a NMR scale reaction between **1**-Na and 2-adamantanone.

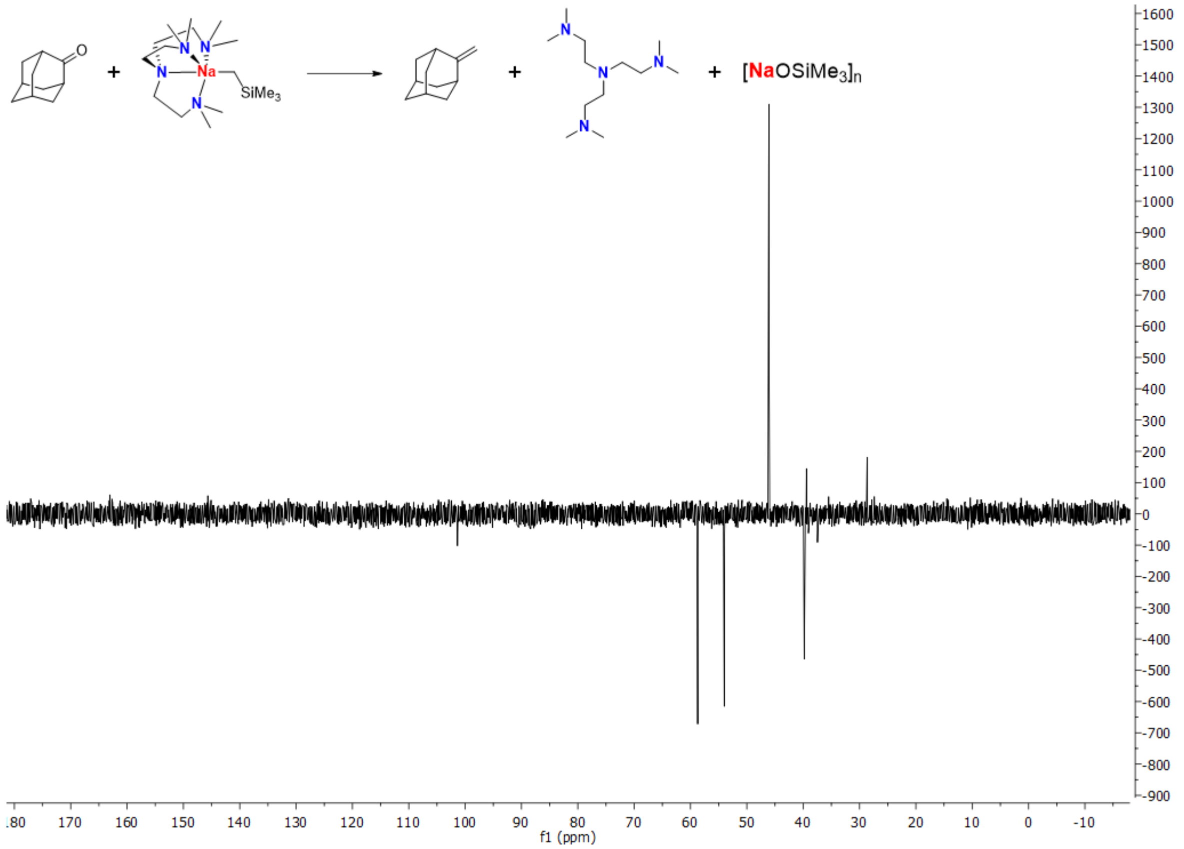

Figure S27: ^{13}C DEPT 135 NMR (d_6-benzene, 25 ˚C, 75 MHz) of a NMR scale reaction between **1**-Na and 2-adamantanone.

Olefination of benzaldehyde (2g) to styrene (4g)

1-Na (0.0112 g, 0.033 mmol) was dissolved in C_6D_6 (0.5 ml). The solution was added to benzaldehyde (**4g**) (0.0035 g, 0.033 mmol) and the resulting solution was transferred to a J Young NMR tube. Complete conversion to styrene was observed after 3 days at room temperature.

Data for styrene (**4g**):

^1H NMR (300 MHz, d_6-benzene, 25 °C): δ (ppm) 7.26 – 7.20 (m, 2H, Ar*H*), 7.14 – 7.00 (m, 3H, Ar*H*), 6.58 (dd, *J* = 17.6, 10.9 Hz, 1H, *H*C=), 5.60 (dd, *J* = 17.6, 1.0 Hz, 1H, =CH_2), 5.07 (dd, *J* = 10.9, 1.0 Hz, 1H, =CH_2).

NMR data are consistent with the literature [10] and authenticated commercial sample.

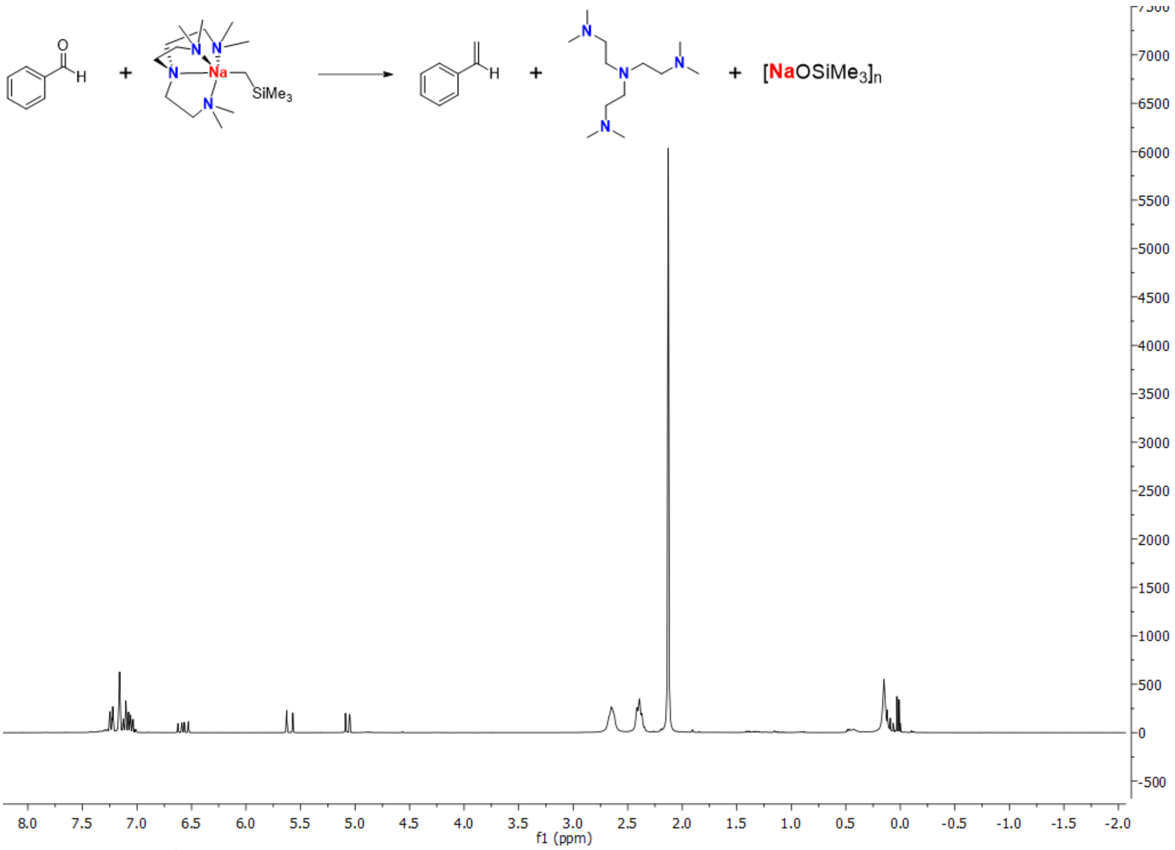

Figure S28: ^1H NMR (d_6-benzene, 25 ˚C, 300 MHz) of a NMR scale reaction between **1**-Na and benzaldehyde.

Olefination of 9-anthracenecarboxaldehyde (2h) to 9-vinylanthracene (4h)
1-Na (0.0102 g, 0.03 mmol) was dissolved in C_6D_6 (0.5 ml). The solution was added to 9-anthracenecarboxaldehyde (**2h**) (0.0062 g, 0.03 mmol) and the resulting solution was transferred to a J Young NMR tube. The reaction was monitored by ^{1}H NMR after 30 minutes.

Data for 9-vinylanthracene (**4h**):
^{1}H NMR (300 MHz, d_6-benzene, 25 °C): δ (ppm) 8.35 – 8.22 (m, 2H, Ar*H*), 8.15 (s, 1H, Ar*H*), 7.88 – 7.75 (m, 2H, Ar*H*), 7.32 – 7.18 (m, 5H, Ar*H*, *H*C=), 5.67 (dd, *J* = 11.4, 2.2 Hz, 1H, =CH_2), 5.40 (dd, *J* = 17.9, 2.2 Hz, 1H, =CH_2).

NMR data are consistent with the literature[11].

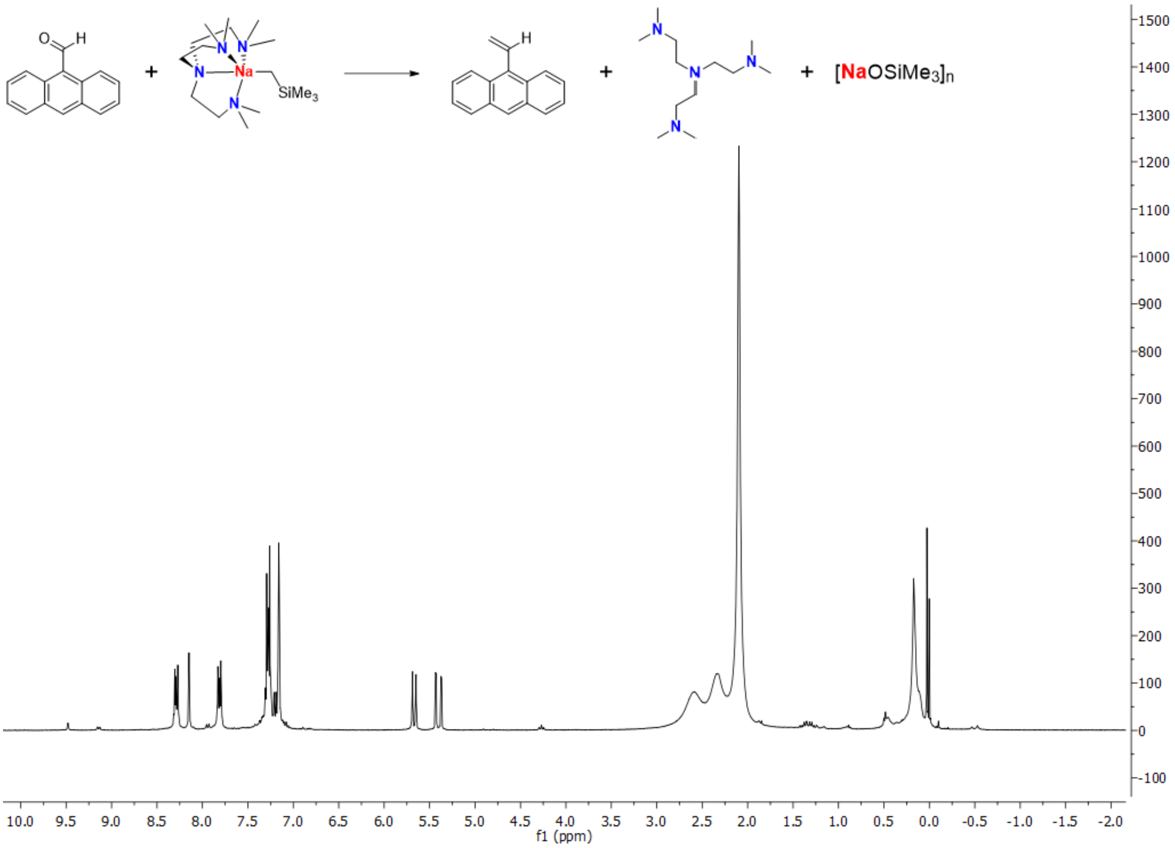

Figure S29: ^{1}H NMR (d_6-benzene, 25 ˚C, 300 MHz) of a NMR scale reaction between **1**-Na and 9-anthracenecarboxaldehyde.

Olefination of 2,2,2-trifluoroacetophenone (2i) to [1-(trifluoromethyl)vinyl]benzene (4i)
1-Na (0.0102 g, 0.03 mmol) was dissolved in C_6D_6 (0.5 ml). The solution was added to 2,2,2-trifluoroacetophenone (**2i**) (0.0052 g, 0.03 mmol) and the resulting solution was transferred to a J Young NMR tube. The reaction was monitored by ^{1}H NMR, which showed >90% conversion to the olefin within 2 hours.

Data for [1-(trifluoromethyl)vinyl]benzene (**4i**):
^{1}H NMR (300 MHz, d$_6$-benzene, 25 °C): δ (ppm) 7.29 – 7.22 (m, 2H, Ar*H*), 7.06 – 6.98 (m, 3H, Ar*H*), 5.55 (q, *J* = 1.4 Hz, 1H, =C*H*$_2$), 5.18 (q, *J* = 1.7 Hz, 1H, =C*H*$_2$).

^{19}F NMR (282 MHz, d$_6$-benzene, 25 °C) δ (ppm) -64.5

NMR data are consistent with the literature[12].

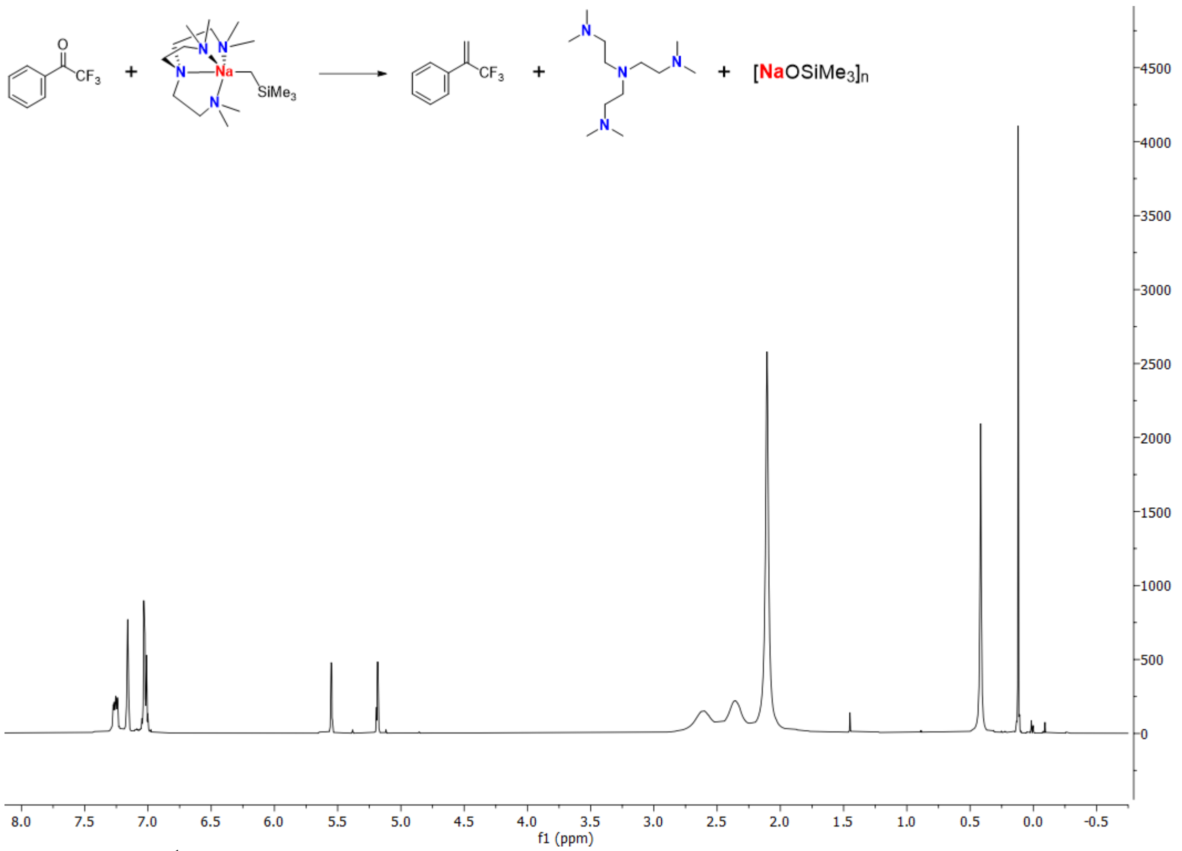

Figure S30: ^{1}H NMR (*d*$_6$-benzene, 25 ˚C, 300 MHz) of a NMR scale reaction between **1**-Na and 2,2,2-trifluoroacetophenone.

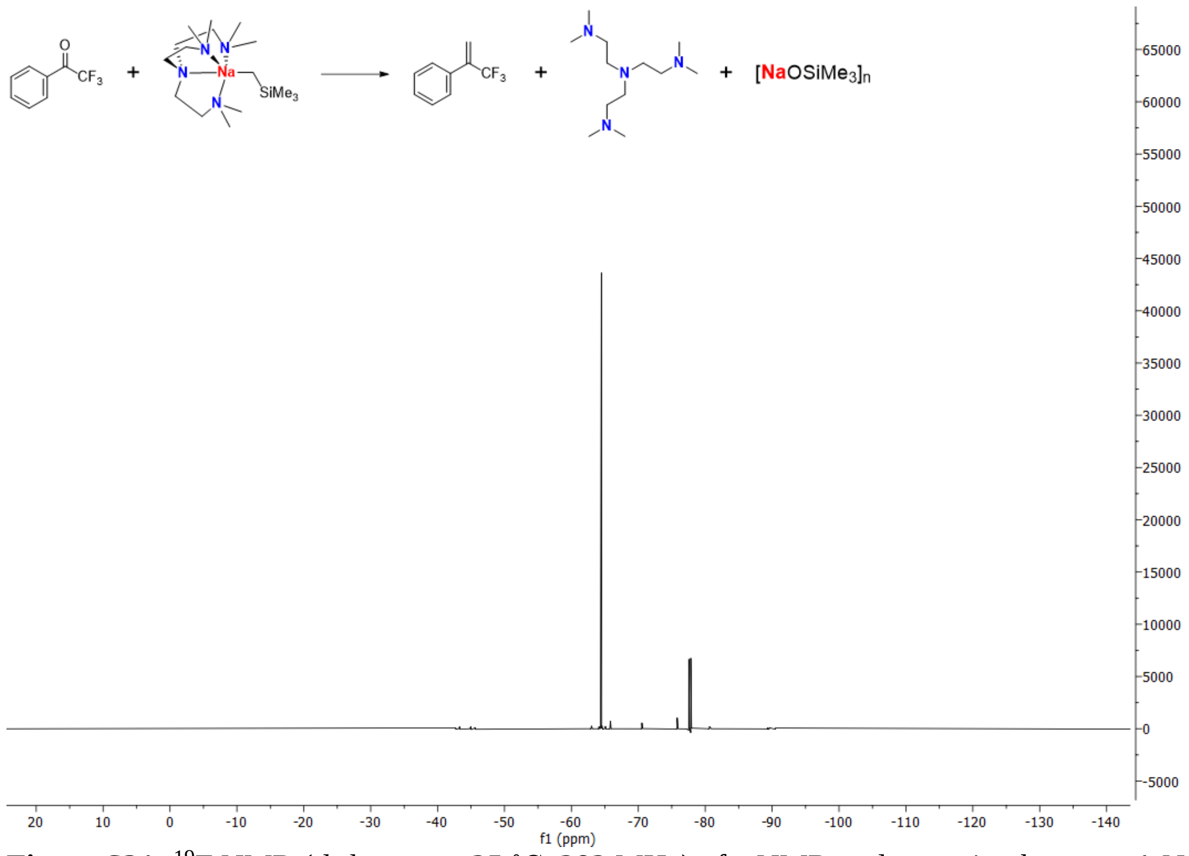

Figure S31: ^{19}F NMR (d_6-benzene, 25 ˚C, 282 MHz) of a NMR scale reaction between **1**-Na and 2,2,2-trifluoroacetophenone.

Olefination of 3,3',5,5'-tetrakis(trifluoromethyl)benzophenone (2j) to 1,1-bis{3,5-bis(trifluromethyl)phenyl}ethylene (4j)

1-Na (0.0102 g, 0.03 mmol) was dissolved in C_6D_6 (0.5 ml). The solution was added to 3,3',5,5'-tetrakis(trifluoromethyl)benzophenone (**2j**) (0.0136 g, 0.03 mmol) and the resulting solution was transferred to a J Young NMR tube. The reaction was monitored by ^{1}H NMR, which showed >90% conversion to the olefin within 30 minutes.

Data for 1,1-bis{3,5-bis(trifluromethyl)phenyl}ethylene (**4j**):
^{1}H NMR (300 MHz, d$_6$-benzene, 25 °C): δ (ppm) 7.68 – 7.61 (m, 2H, Ar*H*), 7.42 – 7.36 (m, 4H, Ar*H*), 4.90 (s, 2H, =C*H*$_2$)

^{19}F NMR (282 MHz, d$_6$-benzene, 25 °C) δ (ppm) -62.8

NMR data are consistent with the literature[13].

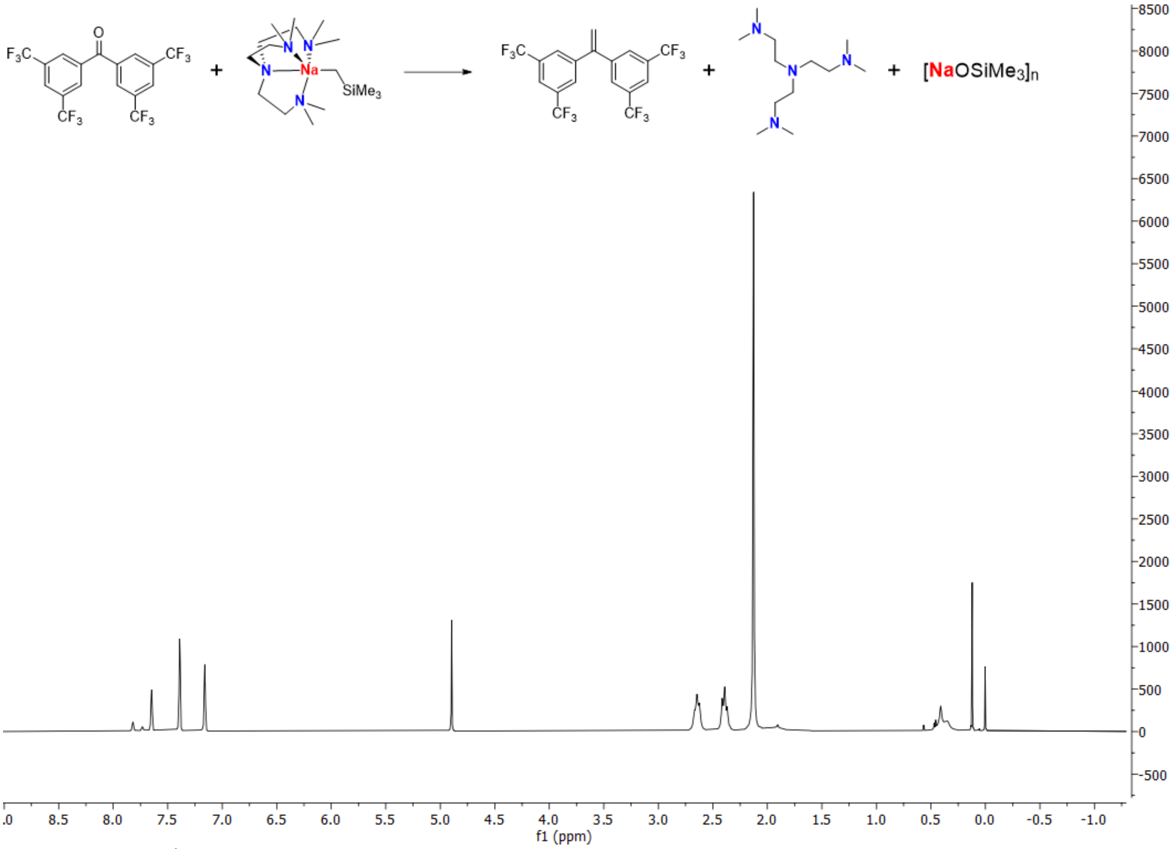

Figure S32: ^{1}H NMR (*d*$_6$-benzene, 25 ˚C, 300 MHz) of a NMR scale reaction between **1**-Na and 3,3',5,5'-tetrakis(trifluoromethyl)benzophenone.

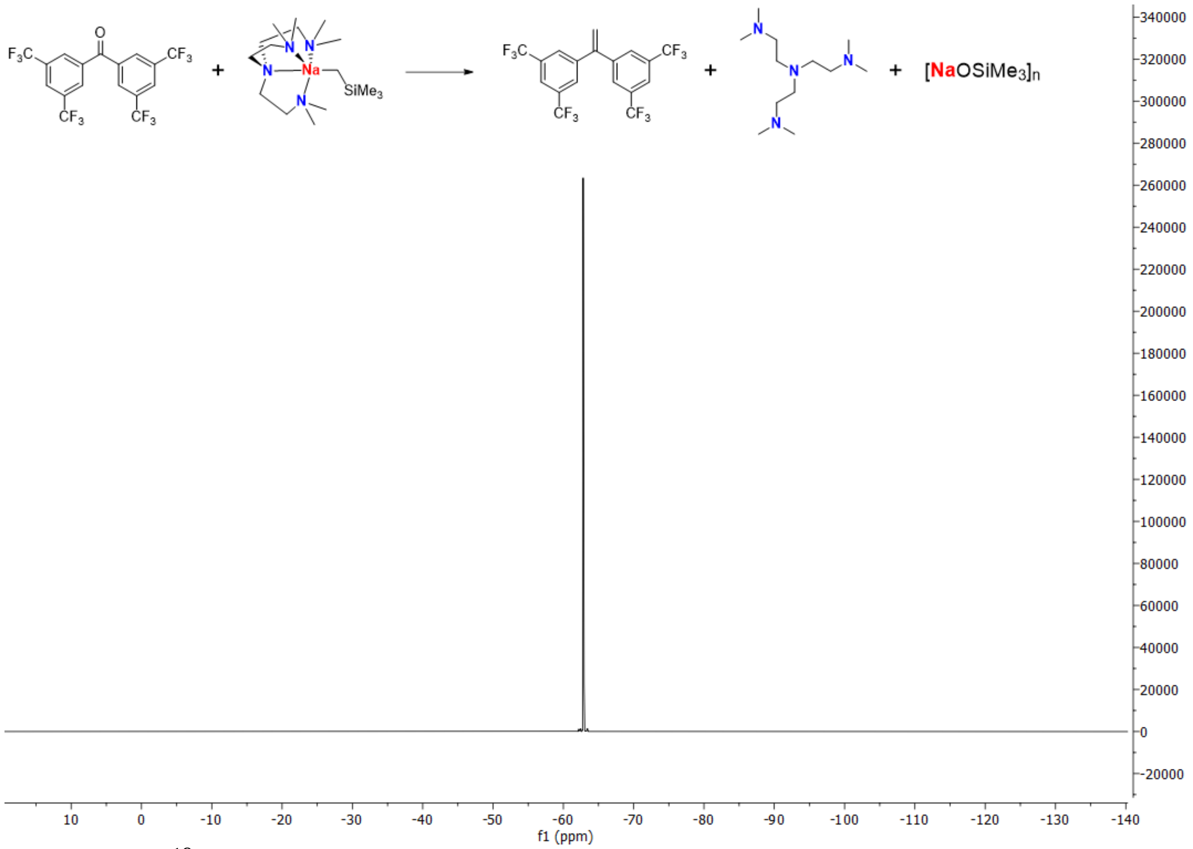

Figure S33: ^{19}F NMR (d_6-benzene, 25 ˚C, 282 MHz) of a NMR scale reaction between **1**-Na and 3,3',5,5'-tetrakis(trifluoromethyl)benzophenone.

Olefination of 4,4′-bis(dimethylamino)benzophenone (2k, Michler′s ketone) to 1,1-bis(4-dimethylaminophenyl)ethylene (4k)

1-Na (0.0102 g, 0.03 mmol) was dissolved in C_6D_6 (0.5 ml). The solution was added to 4,4′-bis(dimethylamino)benzophenone (**2k**) (0.0081 g, 0.03 mmol) and the resulting solution was transferred to a J Young NMR tube. The reaction was monitored by ^{1}H NMR, which showed compete conversion to the olefin within 18 hours at room temperature.

Data for 1,1-bis(4-dimethylaminophenyl)ethylene (**4k**):
^{1}H NMR (300 MHz, d_6-benzene, 25 °C): δ (ppm) 7.59 – 7.51 (m, 4H, Ar*H*), 6.65 – 6.56 (m, 4H, Ar*H*), 5.46 (s, 2H, =CH_2), 2.51 (s, 12H, NMe_2)

NMR data are consistent with the literature[14].

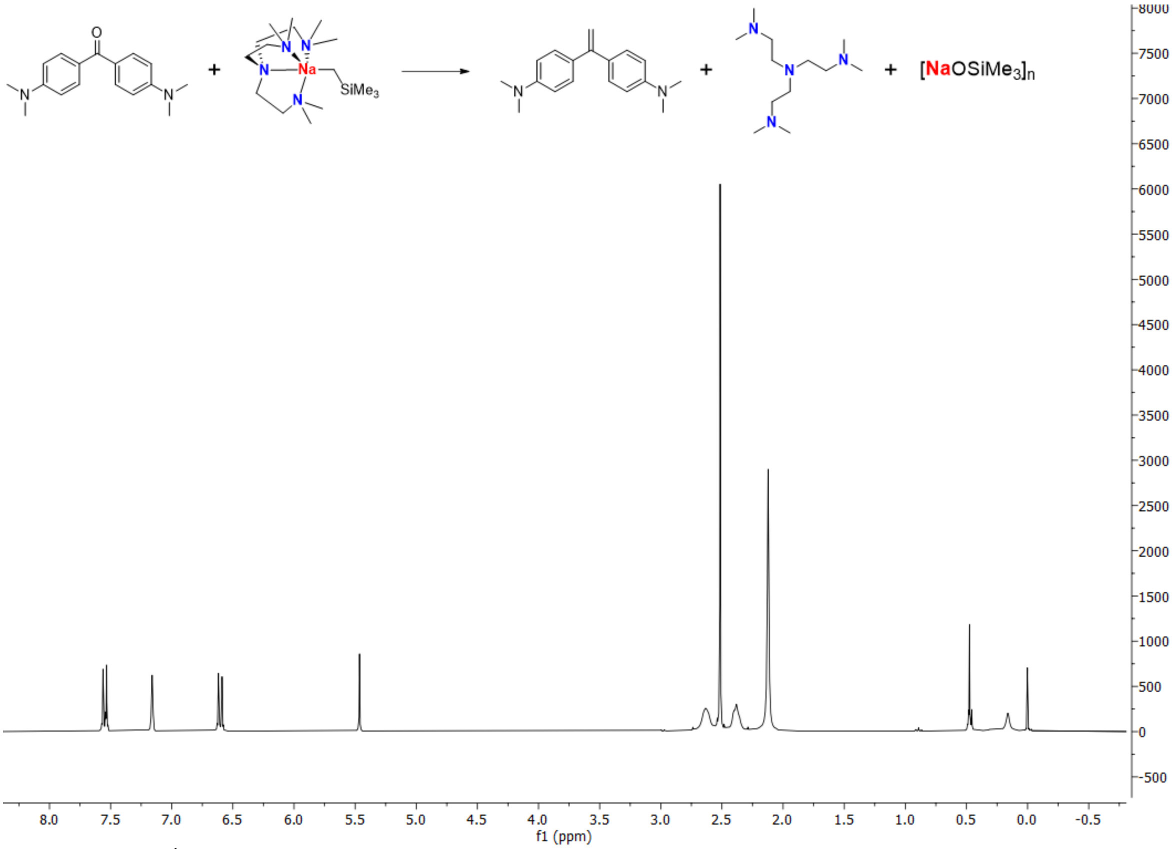

Figure S34: ^{1}H NMR (d_6-benzene, 25 ˚C, 300 MHz) of a NMR scale reaction between **1**-Na and 4,4′-bis(dimethylamino)benzophenone.

Attempted reaction between 1-Na and decafluorobenzophenone (2l)

1-Na (0.0102 g, 0.03 mmol) was dissolved in C_6D_6 (0.5 ml). The solution was added to decafluorobenzophenone (**2l**) (0.0108 g, 0.03 mmol) and the resulting solution was transferred to a J Young NMR tube. The reaction was monitored by ^1H NMR over 24 hours at RT, which showed an intractable mixture and coordination-free Me_6Tren.

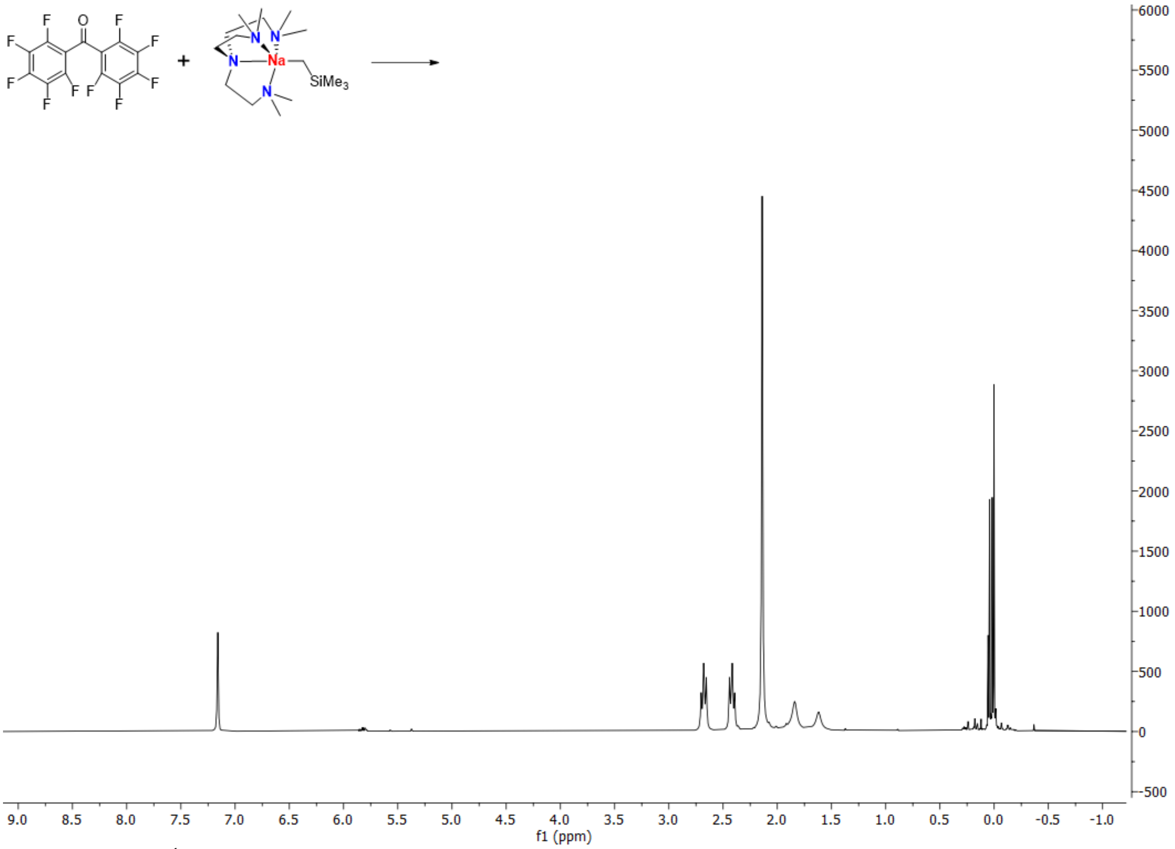

Figure S35: ^1H NMR (d_6-benzene, 25 ˚C, 300 MHz) of a NMR scale reaction between **1**-Na and decafluorobenzophenone.

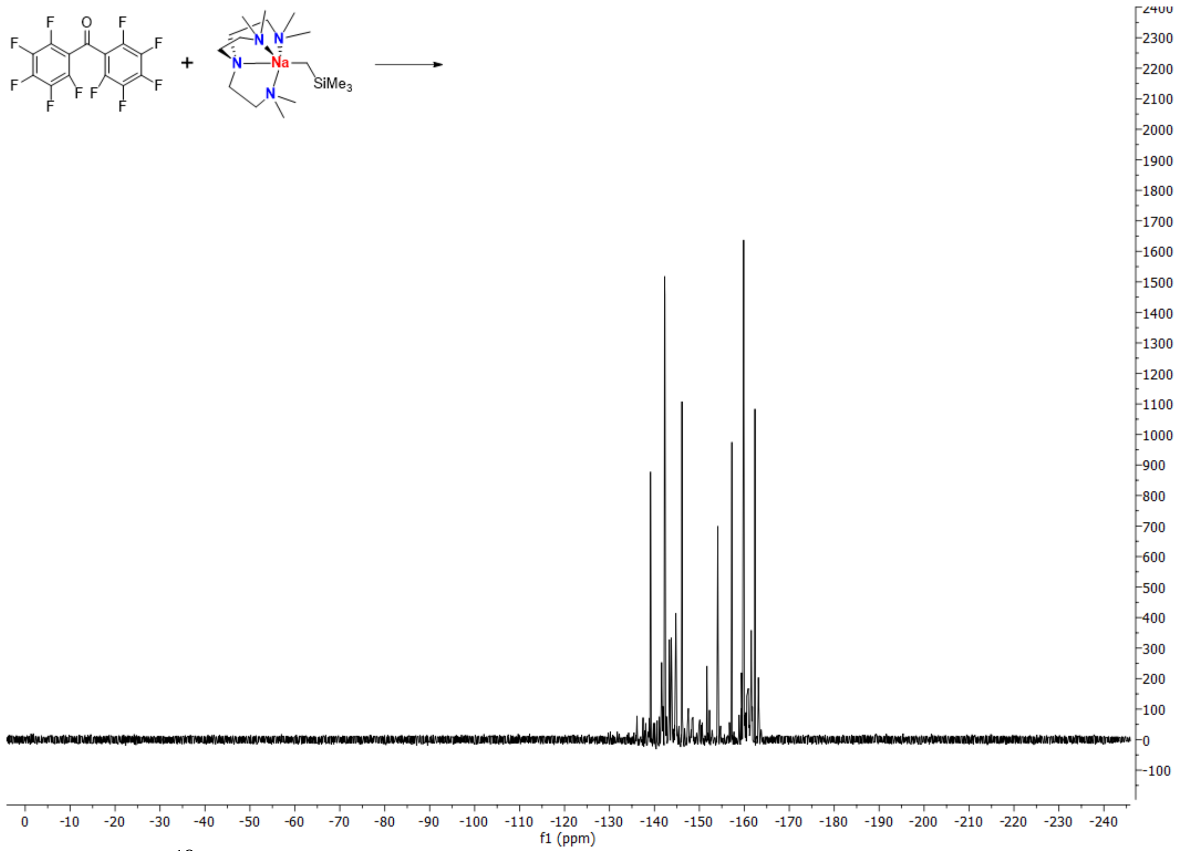

Figure S36: ^{19}F NMR (d_6-benzene, 25 ˚C, 282 MHz) of a NMR scale reaction between **1**-Na and decafluorobenzophenone.

Olefination of *N,N*-dimethylbenzamide (2m) to *N,N*-dimethyl-1-phenylethenamine (4m)
1-Na (0.0136 g, 0.04 mmol) was dissolved in C_6D_6 (0.5 ml). The solution was added to *N,N*-dimethylbenzamide (**2m**) (0.0060 g, 0.04 mmol) and the resulting solution was transferred to a J Young NMR tube. The reaction was monitored by ^{1}H NMR, which showed >75% conversion to the olefin within 24 hours.

Data for *N,N*-dimethyl-1-phenylethenamine (**4m**):
^{1}H NMR (300 MHz, d$_6$-benzene, 25 °C): δ (ppm) 8.12 – 7.94 (m, 2H, Ar*H*), 7.56 – 7.49 (m, 1H, Ar*H*), 7.30 – 7.20 (m, 2H, Ar*H*), 4.42 (s, 1H, cis-*H* to Ph in the C=CH_2(Ph)(N) unit), 4.36 (s, 0.5H, trans-*H* to Ph in the C=CH_2(Ph)(N) unit), 4.14 (s, 0.5H, trans-*H* to Ph in the C=CH_2(Ph)(N) unit), 2.39 (s, 6H, C*H*$_3$). The trans-*H* to Ph in the C=CH_2(Ph)(N) unit split into two singlets (0.5 H each) due to the interconversion between two tetrahedral geometries at the adjacent N center.

NMR data are consistent with the literature[15].

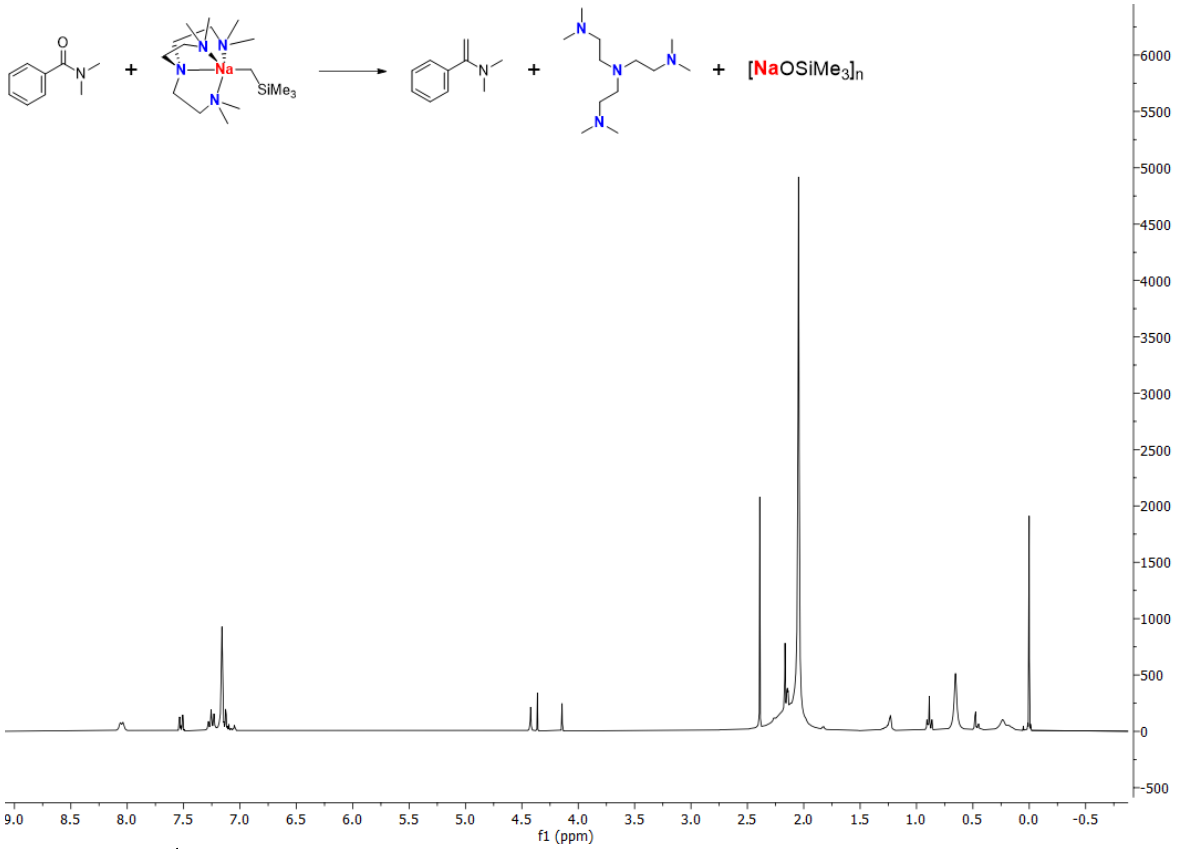

Figure S37: ^{1}H NMR (d_6-benzene, 25 ˚C, 300 MHz) of a NMR scale reaction between **1**-Na and *N,N*-dimethylbenzamide.

Olefination of 1-benzoylpiperidine (2n) to 1-(1-phenylvinyl)piperidine (4n)
1-Na (0.0102 g, 0.03 mmol) was dissolved in C_6D_6 (0.5 ml). The solution was added to 1-benzoylpiperidine (**2n**) (0.0057 g, 0.03 mmol) and the resulting solution was transferred to a J. Young NMR tube. The reaction was monitored by ^{1}H NMR, which showed >75% conversion to the olefin within 4 hours.

Data for 1-(1-phenylvinyl)piperidine (**4n**):
^{1}H NMR (300 MHz, d_6-benzene, 25 °C): δ (ppm) 8.06 (d, *J* = 7.5 Hz, 2H, Ar*H*), 7.68 – 7.55 (m, 1H, Ar*H*), 7.31 – 7.22 (m, 2H, Ar*H*), 4.48 (s, 0.5H, trans-*H* to Ph in the C=CH_2(Ph)(N) unit), 4.45 (s, 1H, cis-*H* to Ph in the C=CH_2(Ph)(N) unit), 4.27 (s, 0.5H, trans-*H* to Ph in the C=CH_2(Ph)(N) unit), 2.76 – 2.53 (m, 4H, CH_2, overlapping signals), 1.46 – 1.31 (m, 6H, CH_2, overlapping signals). The trans-*H* to Ph in the C=CH_2(Ph)(N) unit split into two singlets (0.5 H each) due to the existence of the interconvertible tetrahedral N center at its adjacent position.

^{13}C{^{1}H} NMR (75 MHz, d_6-benzene, 25 °C): *δ* (ppm) 91.1 (=*C*H_2)

NMR data are consistent with the literature[16].

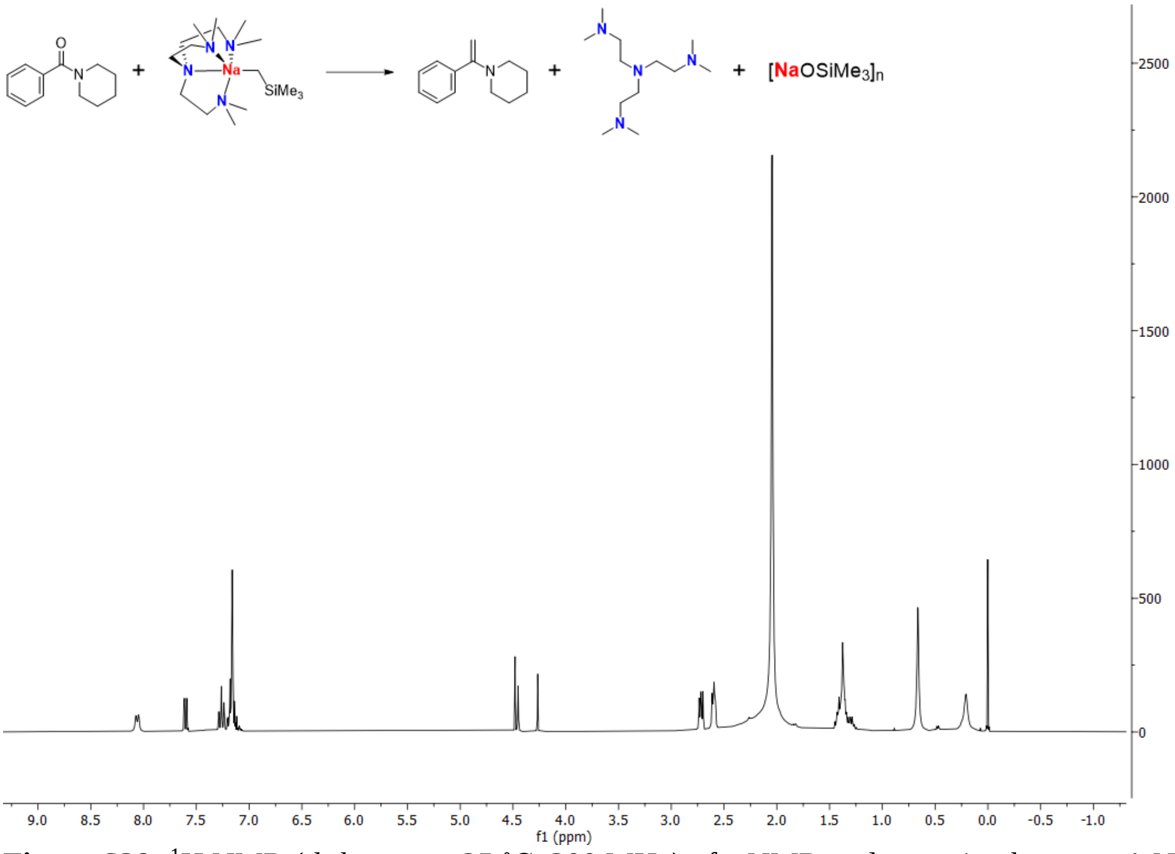

Figure S38: ^{1}H NMR (d_6-benzene, 25 ˚C, 300 MHz) of a NMR scale reaction between **1**-Na and 1-benzoylpiperidine.

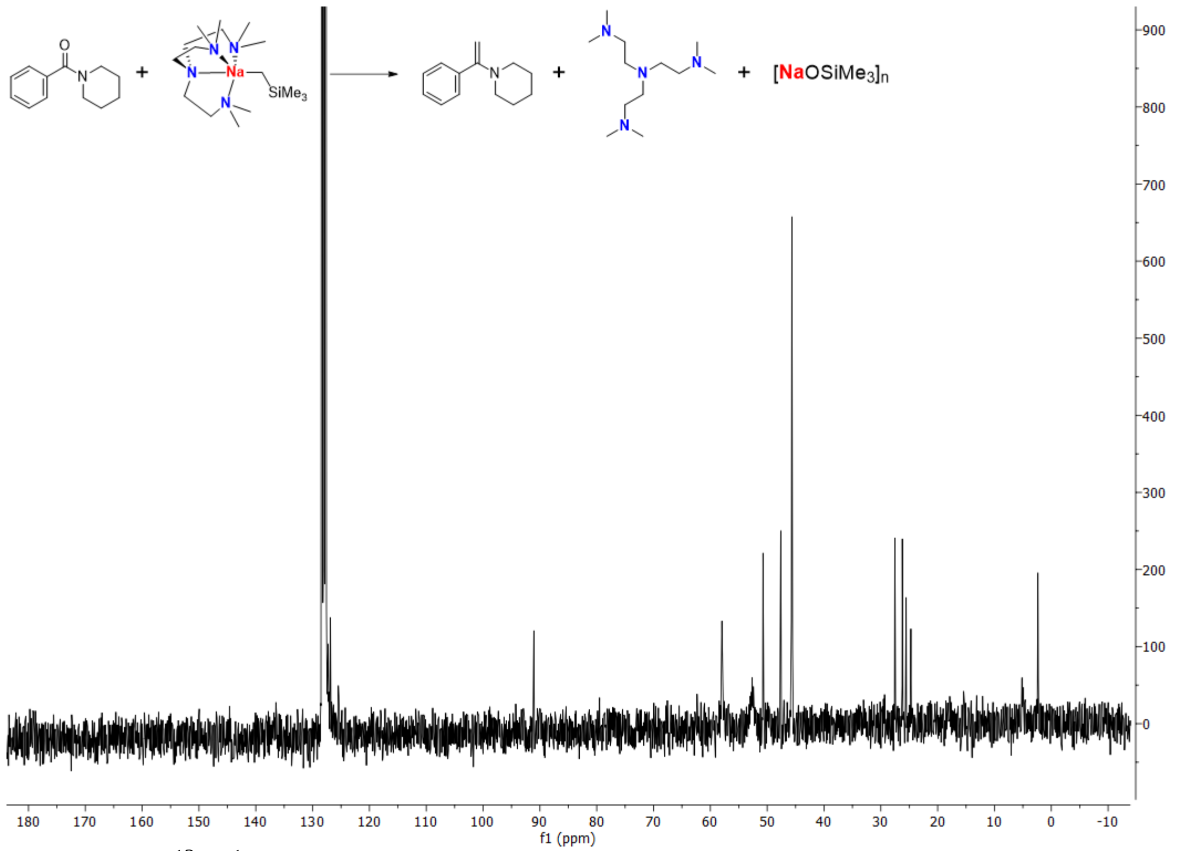

Figure S39: $^{13}C\{^{1}H\}$ NMR (d_6-benzene, 25 ˚C, 75 MHz) of a NMR scale reaction between **1**-Na and 1-benzoylpiperidine.

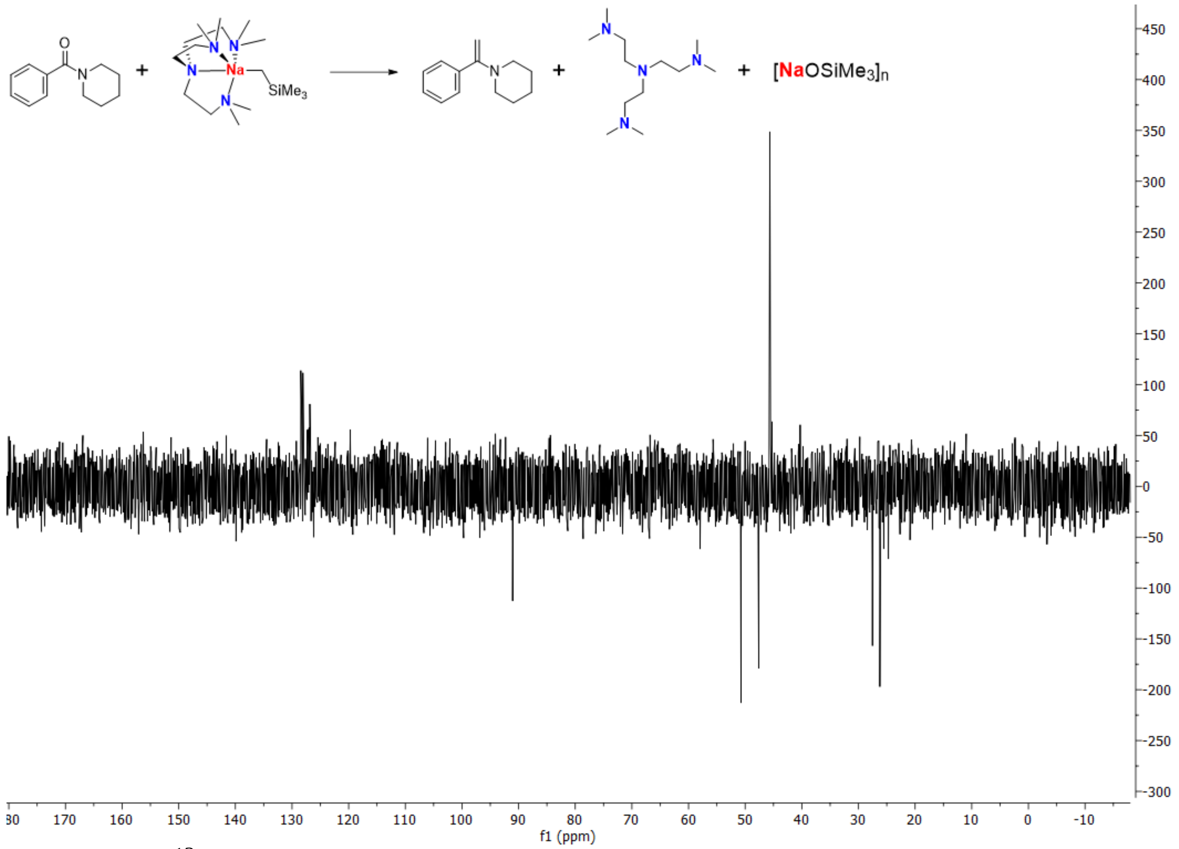

Figure S40: ^{13}C DEPT 135 NMR (d_6-benzene, 25 ˚C, 75 MHz) of a NMR scale reaction between **1**-Na and 1-benzoylpiperidine.

Reaction between 1-Na and phenyl benzoate (2o) and synthesis of complex 6

NMR Scale Reaction:

1-Na (0.0102 g, 0.03 mmol) was dissolved in C_6D_6 (0.5 ml). The solution was added to phenyl benzoate (**2o**) (0. g, 0.03 mmol) and the resulting solution was transferred to a J Young NMR tube. The reaction was monitored by ^{1}H NMR after 15 minutes at which point there was no insolubles in the NMR stube and after 1 day, when insolubles were observed in the NMR tube, with **6** and tetramethylsilane and Ph-OSiMe, observed in ^{1}H NMR.

Scale up:

1-Na (0.1703 g, 0.5 mmol) was dissolved in benzene (1 ml). The solution was added to a solution of phenyl benzoate (**2o**) (0.0991 g, 0.5 mmol) in benzene (1 ml) at room temperature. The resulting yellow/orange solution was left at room temperature for 10 minutes, before the volatiles were removed *in vacuo*. A pale-yellow solid resulted. Hexane (14 ml) was added, some of the solid dissolved and the solution was filtered and placed in a –35 ˚C freezer. Overnight yellow crystals of **6** suitable for SCXRD resulted. The mother liquor was removed and the solid dried *in vacuo*. Pure **6** resulted as a crystalline solid resulted (0.0200 g, 17% yield based on Na). The volatiles were removed *in vacuo* from the mother liquor. A yellow oil resulted, which ^{1}H NMR revealed to be free Me_6Tren.

Data for **6**:

^{1}H NMR (300 MHz, d_6-benzene, 25 °C): δ (ppm) 8.36 – 8.26 (m, 4H, Ar*H*), 7.39 – 7.29 (m, 4H, Ar*H*), 7.28 – 7.19 (m, 2H, Ar*H*), 6.97 (s, 1H, C*H*), 2.22 – 2.06 (m, 30H, Me_6Tren).

The poor solubility of the crystals of **6** in C_6D_6 prevented ^{13}C NMR from being collected.

Data for Ph-$OSiMe_3$:
^1H NMR (300 MHz, d_6-benzene, 25 °C): δ (ppm) 7.14 – 7.06 (m, 2H), 6.92 – 6.82 (m, 3H), 0.15 (s, 9H).

NMR data for Ph-$OSiMe_3$ are consistent with the literature[17].

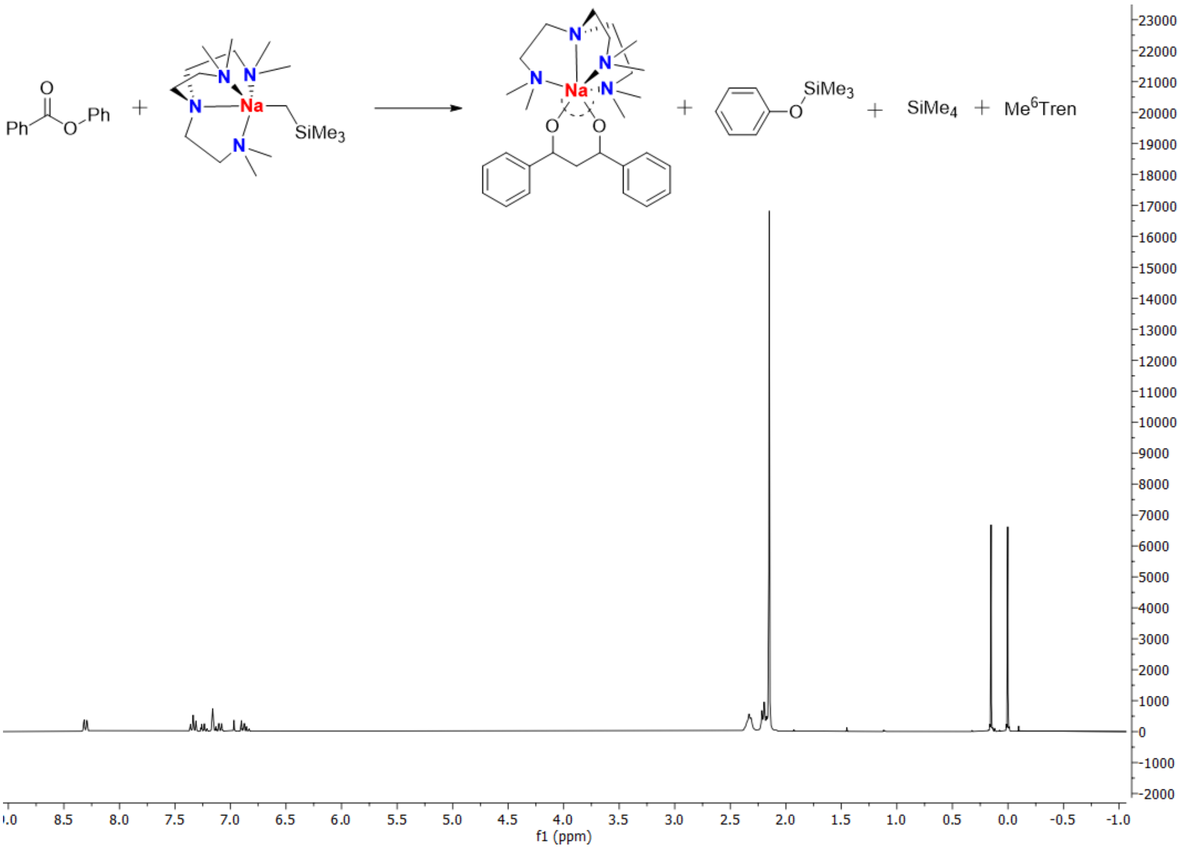

Figure S41: ^{1}H NMR (d_6-benzene, 25 ˚C, 300 MHz) of a NMR scale reaction between **1**-Na and phenyl benzoate after 1 day.

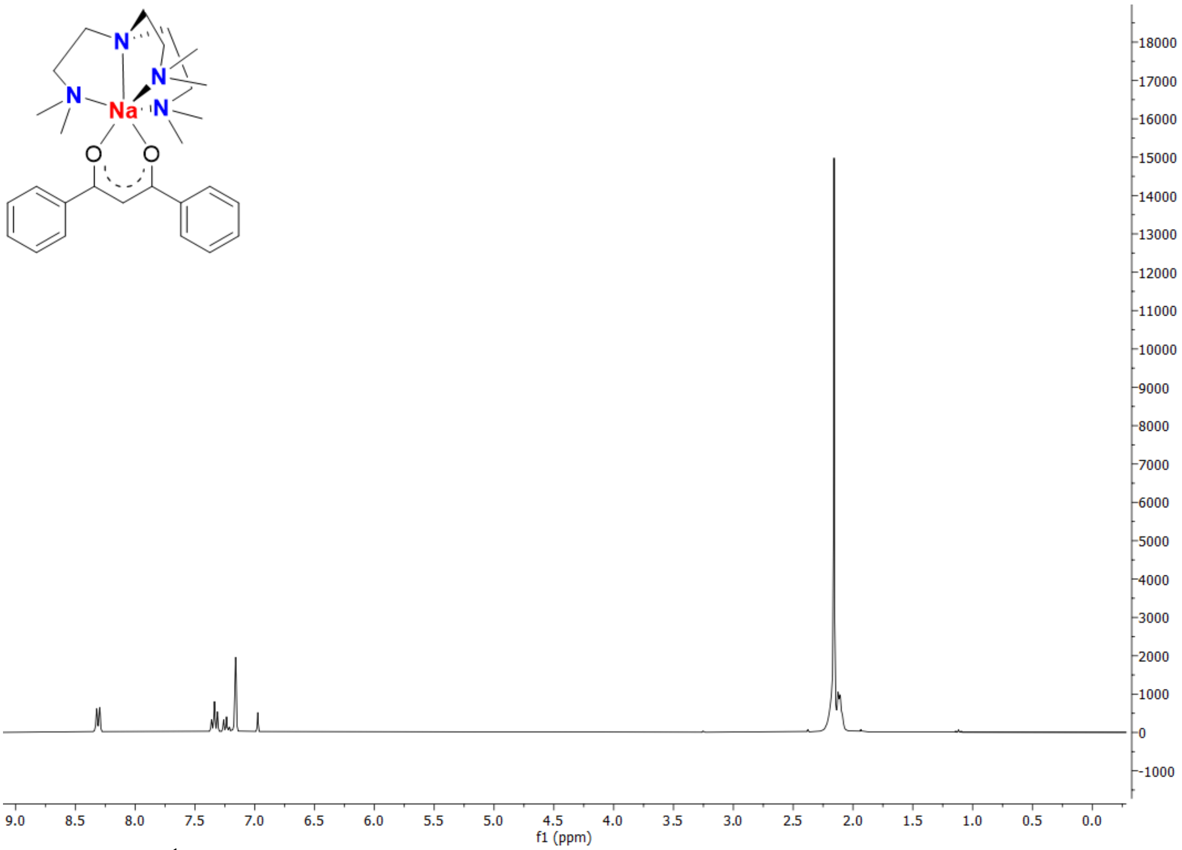

Figure S42: ^{1}H NMR (d_6-benzene, 25 ˚C, 300 MHz) of **6**.

1.5 Control reactions

Reaction between [Li(CH$_2$SiMe$_3$)(Me$_6$Tren)] (1-Li) and benzophenone (2a)

[Li(CH$_2$SiMe$_3$)(Me$_6$Tren)] (**1**-Li) (0.0097 g, 0.03 mmol) was dissolved in C_6D_6 (0.5 ml). The solution was added to benzophenone (0.0055 g, 0.03 mmol) and the resulting solution was transferred to a J Young NMR tube. The NMR tube was heated to 60 ˚C for 20 hours. ^{1}H NMR was ran, which showed free Me$_6$Tren and **3-Li** with no observable conversion to the olefin. ^{1}H for **3**-Li matched that previously reported.[1]

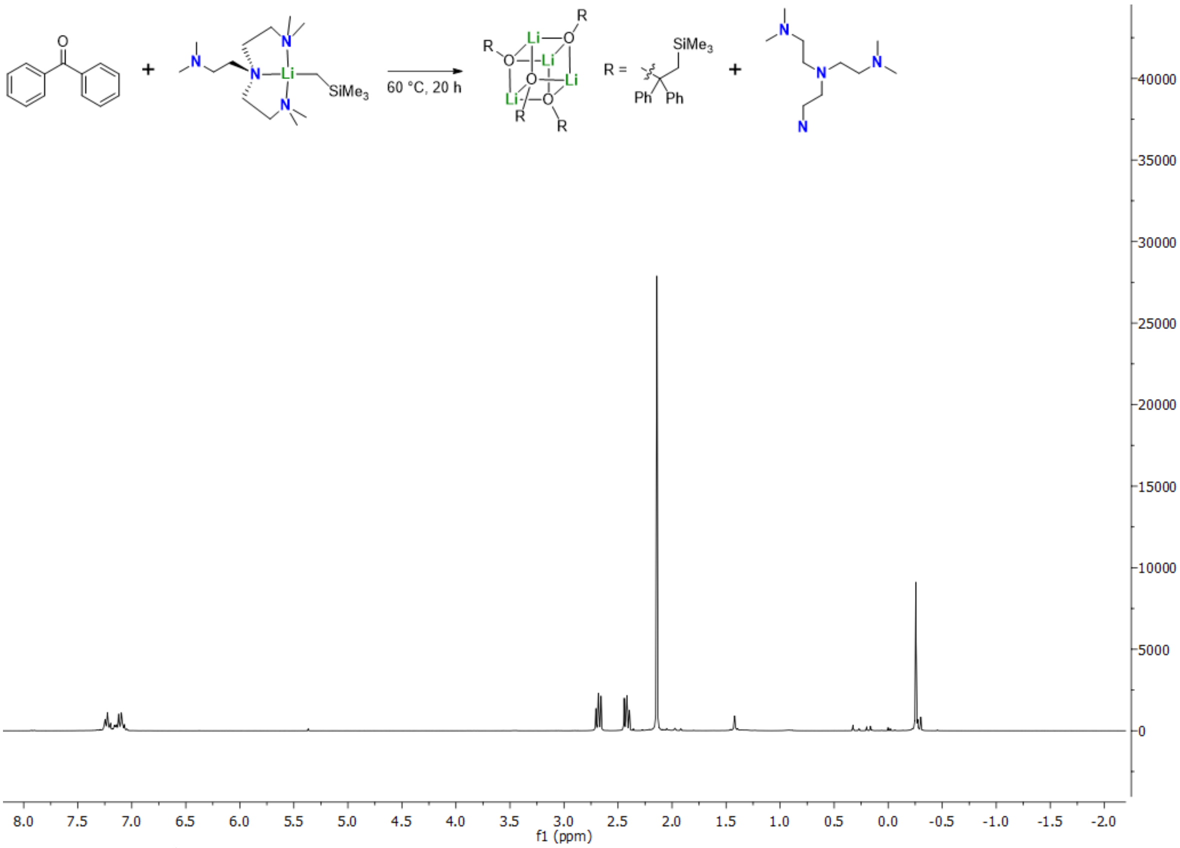

Figure S43: ^{1}H NMR (d_6-benzene, 25 ˚C, 300 MHz) of a NMR scale reaction between **1**-Li and benzophenone.

Reaction between [$NaCH_2SiMe_3$]$_\infty$ and benzophenone (synthesis of 3-Na)

NMR scale-reaction:

Benzophenone (0.0055 g, 0.03 mmol) was dissolved in C_6D_6 (0.5 ml). The solution was added to [$NaCH_2SiMe_3$]$_\infty$ (0.0033 g, 0.03 mmol) and the resulting solution was transferred to a J Young NMR tube. The NMR tube was heated to 60 ˚C for 20 hours. ^{1}H NMR was ran, which showed **3-Na** and <10% conversion to the olefin.

Scale-up:

Benzophenone (0.0911 g, 0.5 mmol) was dissolved in benzene (1 ml). The solution was added to a suspension of [$NaCH_2SiMe_3$]$_\infty$ (0.0551 g, 0.5 mmol). A pale green solution resulted. The volatiles were removed *in vacuo*. A colourless crystalline solid resulted. The solid was dissolved in hexane (8 ml). The solution was filtered then concentrated to ~5 ml. The solution was placed in a –35 ˚C freezer and colourless crystals of **3-Na** suitable for single-crystal x-ray diffraction resulted after 24 hours. The mother liquor was removed and the crystals dried *in vacuo* (0.0398 g). ^{1}H NMR showed no change at room temperature after 5 days.

Data for **3-Na**

^{1}H NMR (300 MHz, d_6-benzene, 25 °C): δ (ppm) 7.36 – 7.28 (m, 16H, Ar*H*), 7.21 – 7.11 (m, 16H, Ar*H*), 7.11 – 7.03 (m, 8H, Ar*H*), 1.44 (s, 8H, CH_2Si), -0.24 (s, 36H, Si(CH_3)$_3$).

$^{13}C\{^1H\}$ NMR (75 MHz, d_6-benzene, 25 °C): δ (ppm) 156.8 (quaternary carbon), 128.5 (ArCH), 126.6 (ArCH), 125.7 (ArCH), 79.2 (quaternary carbon), 38.5 (CH$_2$Si), 0.1 (Si(CH$_3$)$_3$).

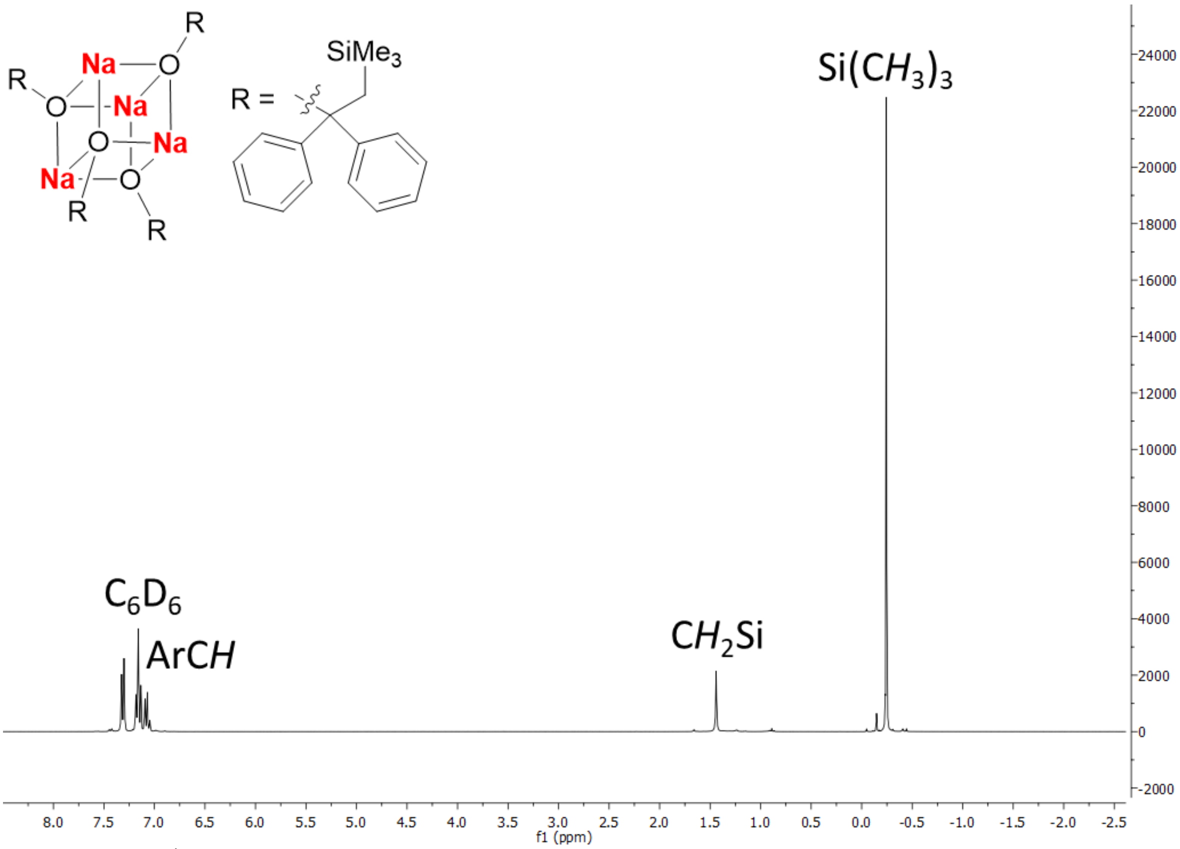

Figure S44: ^{1}H NMR (d_6-benzene, 25 ˚C, 300 MHz) of **3**-Na

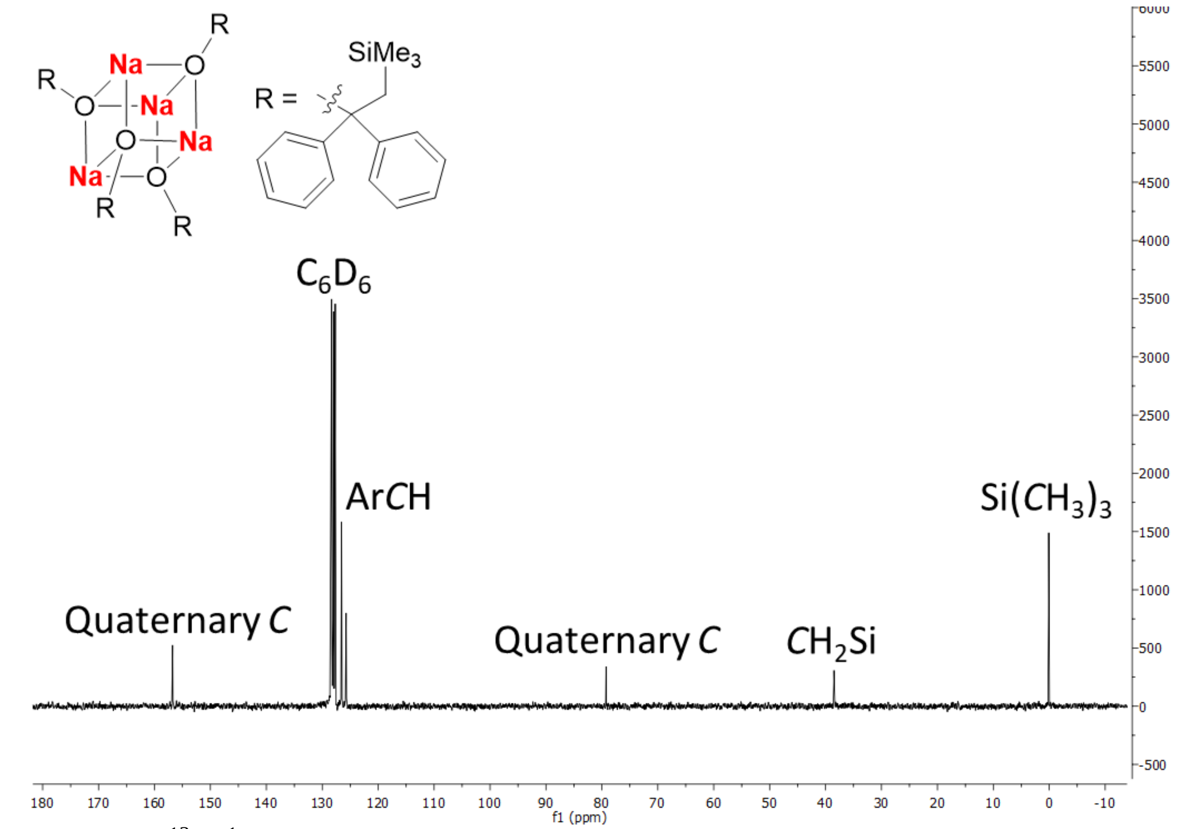

Figure S45: ${}^{13}C\{{}^{1}H\}$ NMR (d_6-benzene, 25 ˚C, 75 MHz) of **3**-Na

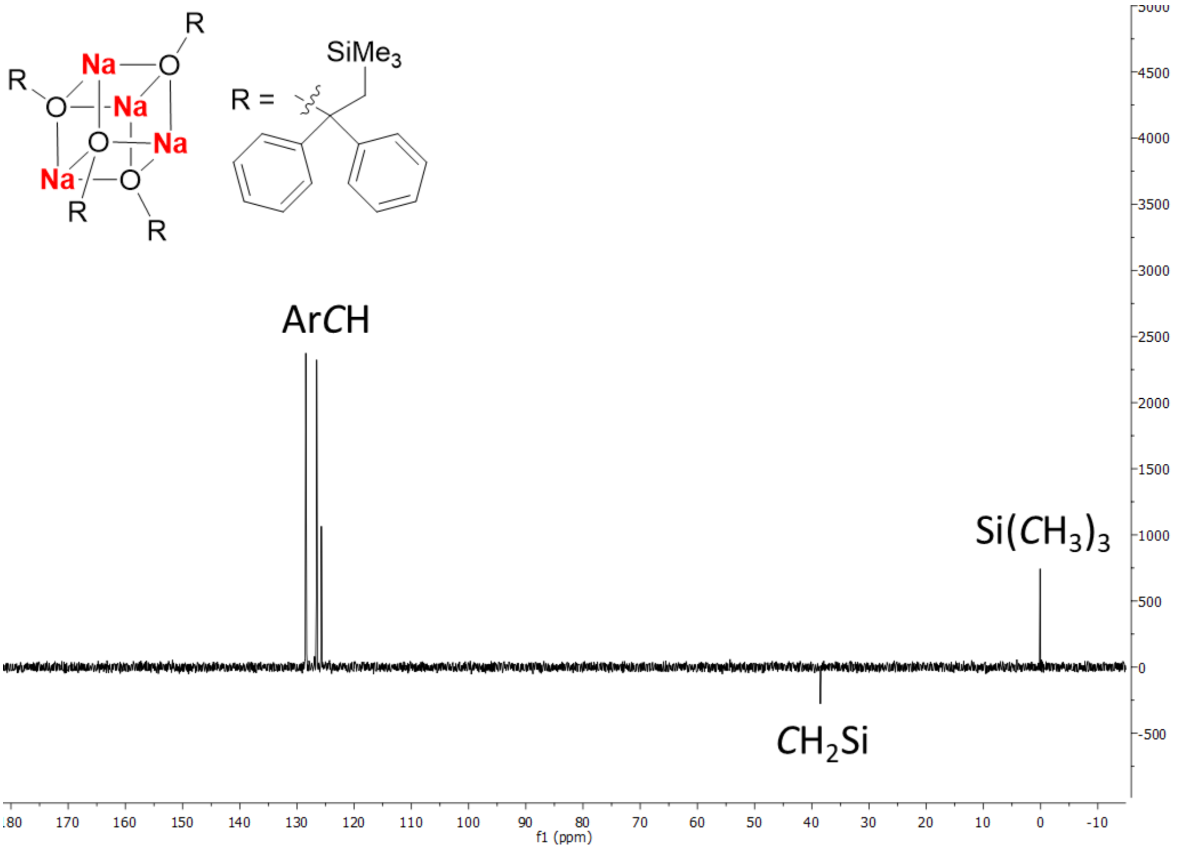

Figure S46: ^{13}C DEPT 135 NMR (d_6-benzene, 25 ˚C, 75 MHz) of **3**-Na

Reaction between [$NaCH_2SiMe_3$]$_\infty$ and phenyl cyclohexyl ketone (2d)
Phenyl cyclohexyl ketone (**2d**) (0.0075 g, 0.04 mmol) was dissolved in C_6D_6 (0.5 ml). The solution was added to [$NaCH_2SiMe_3$]$_\infty$ (0.0044 g, 0.04 mmol) and the resulting solution was transferred to a J Young NMR tube. ^{1}H NMR was ran after 3 hours at room temperature, which showed no observable conversion to the olefin.

Reaction between [$NaCH_2SiMe_3$]$_\infty$ and 2,2,2-trimethylacetophenone (2e)
2,2,2-Trimethylacetophenone (**2e**) (0.0065 g, 0.04 mmol) was dissolved in C_6D_6 (0.5 ml). The solution was added to [$NaCH_2SiMe_3$]$_\infty$ (0.0044 g, 0.04 mmol) and the resulting solution was transferred to a J Young NMR tube. ^{1}H NMR was ran after 3 hours at room temperature, which showed ~25 % conversion to the olefin.

Reaction between [$NaCH_2SiMe_3$]$_\infty$ and 2-adamantanone (2f)
2-Adamantanone (**2f**) (0.0060 g, 0.04 mmol) was dissolved in C_6D_6 (0.5 ml). The solution was added to [$NaCH_2SiMe_3$]$_\infty$ (0.0044 g, 0.04 mmol) and the resulting solution was transferred to a J Young NMR tube. ^{1}H NMR was ran after 3 hours at room temperature, which showed ~10 % conversion to the olefin.

Reaction between [$NaCH_2SiMe_3$]$_\infty$ and 9-anthracenecarboxaldehyde (2h)
9-Anthracenecarboxaldehyde (**2h**) (0.0082 g, 0.04 mmol) was dissolved in C_6D_6 (0.5 ml). The solution was added to [$NaCH_2SiMe_3$]$_\infty$ (0.0044 g, 0.04 mmol) and the resulting solution was transferred to a J Young NMR tube. ^{1}H NMR was ran after heating to 60 ˚C for 20 hours, which showed no observable conversion to the olefin.

Reaction between [$NaCH_2SiMe_3$]$_\infty$ and 2,2,2-trifluoroacetophenone (2i)
2,2,2-Trifluoroacetophenone (**2i**) (0.0070 g, 0.04 mmol) was dissolved in C_6D_6 (0.5 ml). The solution was added to [$NaCH_2SiMe_3$]$_\infty$ (0.0044 g, 0.04 mmol) and the resulting solution was transferred to a J Young NMR tube. ^{1}H NMR was ran after 3 days at room temperature, which showed no observable conversion to the olefin.

Reaction between [$NaCH_2SiMe_3$]$_\infty$ and 3,3',5,5'-tetrakis(trifluoromethyl)benzophenone (2j)
3,3',5,5'-Tetrakis(trifluoromethyl)benzophenone (**2j**) (0.0182g, 0.04 mmol) was dissolved in C_6D_6 (0.5 ml). The solution was added to [$NaCH_2SiMe_3$]$_\infty$ (0.0044 g, 0.04 mmol) and the resulting solution was transferred to a J Young NMR tube. ^{1}H NMR was ran after 3 day at room temperature, which showed no observable conversion to the olefin.

Reaction between [$NaCH_2SiMe_3$]$_\infty$ and 4,4′-bis(dimethylamino)benzophenone (2k, Michler′s ketone)
4,4′-Bis(dimethylamino)benzophenone (**2k**) (0.0107 g, 0.04 mmol) was dissolved in C_6D_6 (0.5 ml). The solution was added to [$NaCH_2SiMe_3$]$_\infty$ (0.0044 g, 0.04 mmol) and the resulting solution was transferred to a J Young NMR tube. ^{1}H NMR was ran after heating to 60 ˚C for 18 hours, which showed no observable conversion to the olefin.

1.6 Ligand-catalyzed reactions

$[NaCH_2SiMe_3]_\infty$ and benzophenone (2a), 20 mol% Me_6Tren catalytic reaction: Benzophenone (**2a**) (0.0063 g, 0.0347 mmol) was dissolved in C_6D_6 (0.25 ml). The solution was added to Me_6Tren (0.0016 g, 0.0069 mmol, 0.2 equiv.) The mixed solution was added at room temperature to a suspension of $[NaCH_2SiMe_3]_\infty$ (0.0038 g, 0.0347 mmol) in C_6D_6 (0.25 ml). The resulting solution was transferred to a J Young NMR tube. ^{1}H showed complete conversion to the olefin after 3 hours.

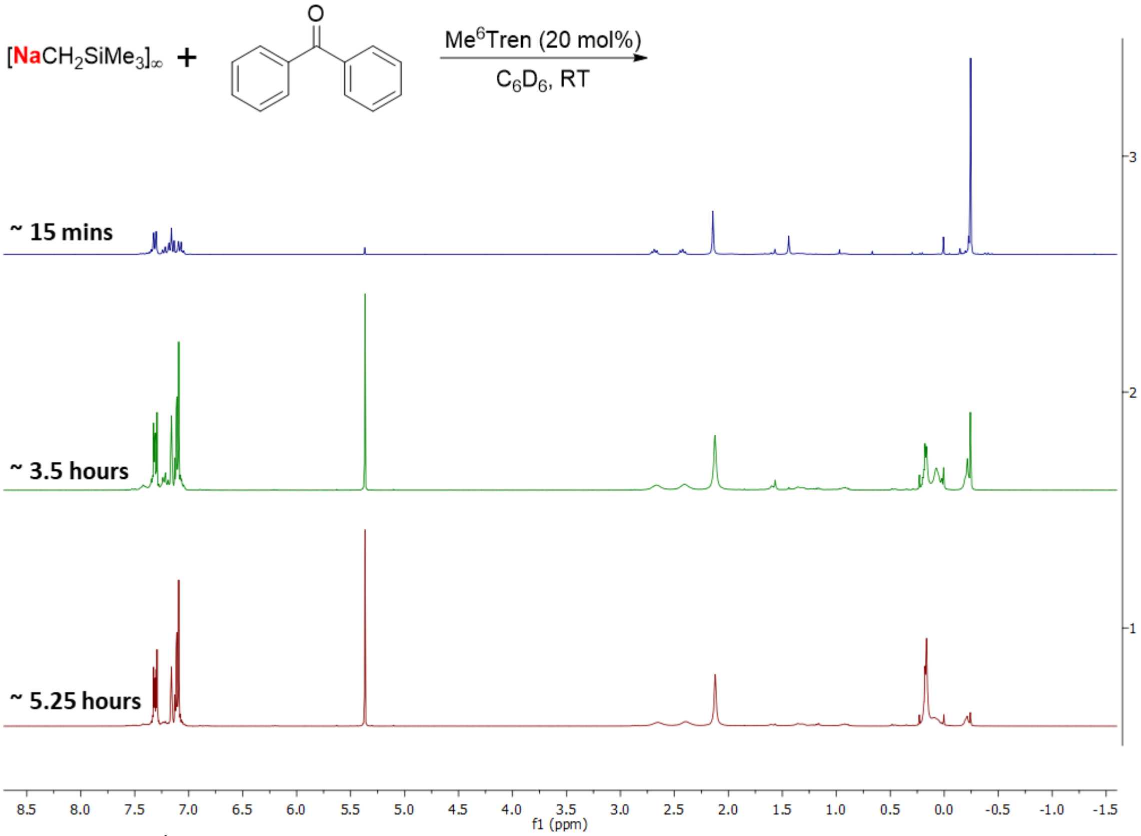

Figure S47: ^{1}H NMR (d_6-benzene, 25 ˚C, 300 MHz) of a NMR scale reaction between $[NaCH_2SiMe_3]_\infty$, benzophenone and 20 mol% Me_6Tren.

[$NaCH_2SiMe_3$]∞ and benzophenone (2a), 5 mol% Me_6Tren catalytic reaction:
A solution of Me_6Tren in C_6D_6 (0.4755 g, 0.002 mmol, 0.5 ml, $[Cat]_0$ = 4 mM) was added to benzophenone (**2a**) (0.0073 g, 0.04 mmol, $[Sub]_0$ = 80 mM). The solution was added at room temperature to [$NaCH_2SiMe_3$]∞ (0.0044 g, 0.04 mmol). The resulting solution was transferred to a J Young NMR tube. ^{1}H showed complete conversion to the olefin within 20 hours.

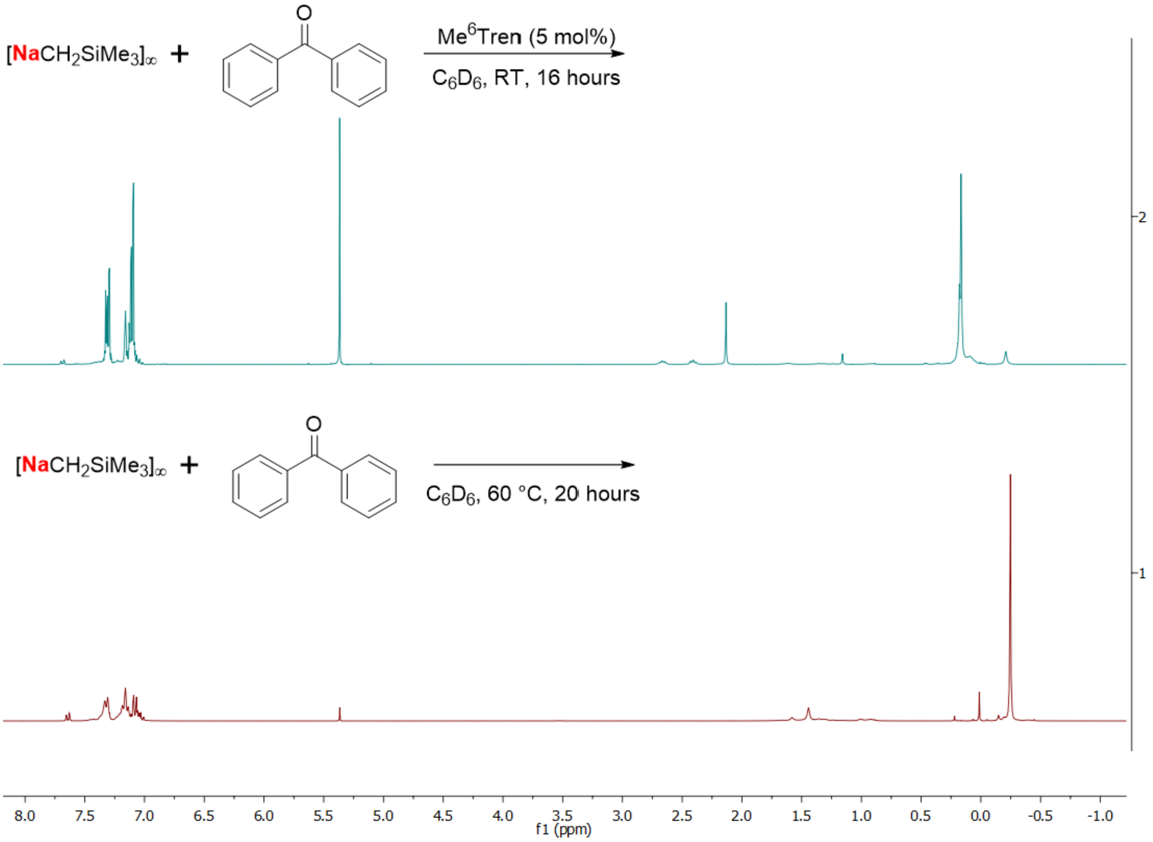

Figure S48: Stacked ^{1}H NMR (d_6-benzene, 25 ˚C, 300 MHz) of a NMR scale reaction between [$NaCH_2SiMe_3$]∞, benzophenone and 5 mol% Me_6Tren (top: cyan) and control reaction between [$NaCH_2SiMe_3$]∞ and benzophenone (bottom: red).

$[NaCH_2SiMe_3]_\infty$ and phenyl cyclohexyl ketone (2d), 5 mol% Me_6Tren catalytic reaction: A solution of Me_6Tren in C_6D_6 (0.4755 g, 0.002 mmol, 0.5 ml, $[Cat]_0$ = 4 mM) was added to phenyl cyclohexyl ketone (**2d**) (0.0075 g, 0.04 mmol, $[Sub]_0$ = 80 mM). The solution was added at room temperature to $[NaCH_2SiMe_3]_\infty$ (0.0044 g, 0.04 mmol). The resulting solution was transferred to a J Young NMR tube. ^{1}H showed complete conversion to the olefin within 2 hours.

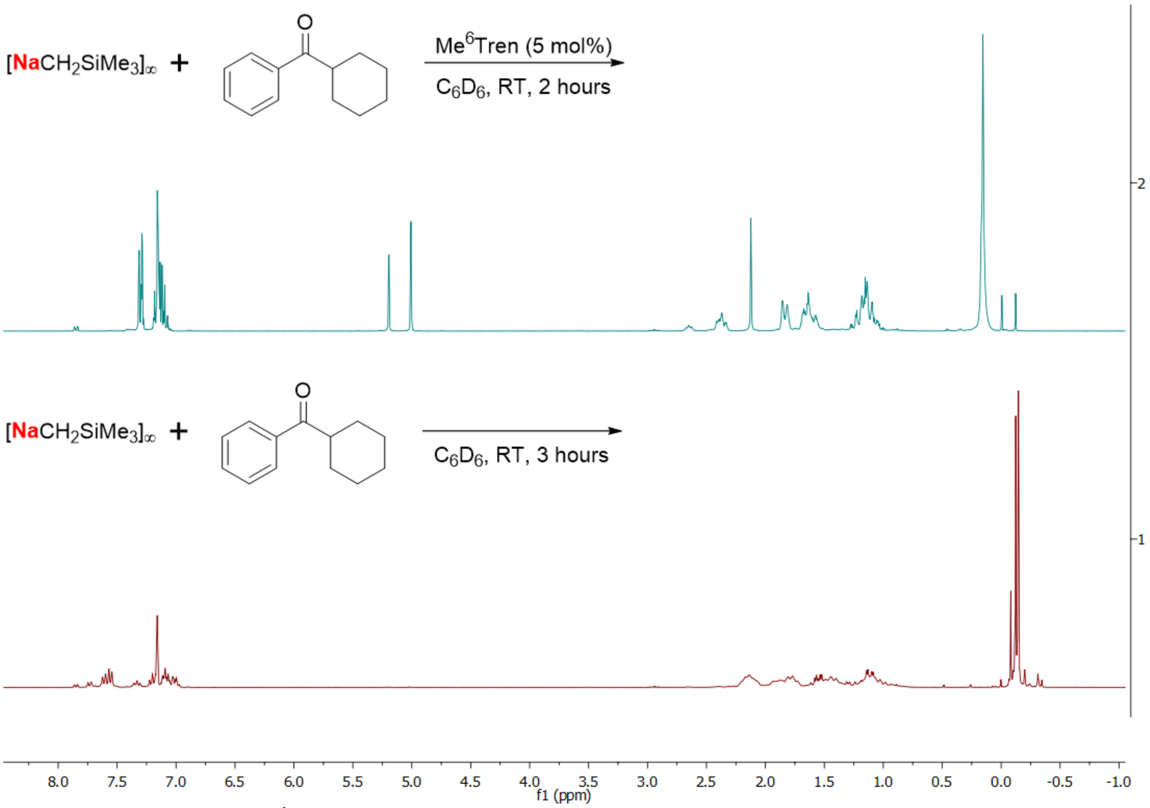

Figure S49: Stacked ^{1}H NMR (d_6-benzene, 25 ˚C, 300 MHz) of a NMR scale reaction between $[NaCH_2SiMe_3]_\infty$, phenyl cyclohexyl ketone and 5 mol% Me_6Tren (top: cyan) and control reaction between $[NaCH_2SiMe_3]_\infty$ and phenyl cyclohexyl ketone (bottom: red).

[$NaCH_2SiMe_3$]$_\infty$ and 2,2,2-trimethylacetophenone (2e), 5 mol% Me_6Tren catalytic reaction:

A solution of Me_6Tren in C_6D_6 (0.4755 g, 0.002 mmol, 0.5 ml, [Cat]$_0$ = 4 mM) was added to 2,2,2-trimethylacetophenone (**2e**) (0.0065 g, 0.04 mmol, [Sub]$_0$ = 80 mM). The solution was added at room temperature to [$NaCH_2SiMe_3$]$_\infty$ (0.0044 g, 0.04 mmol). The resulting solution was transferred to a J Young NMR tube. ^{1}H showed complete conversion to the olefin within 2 hours.

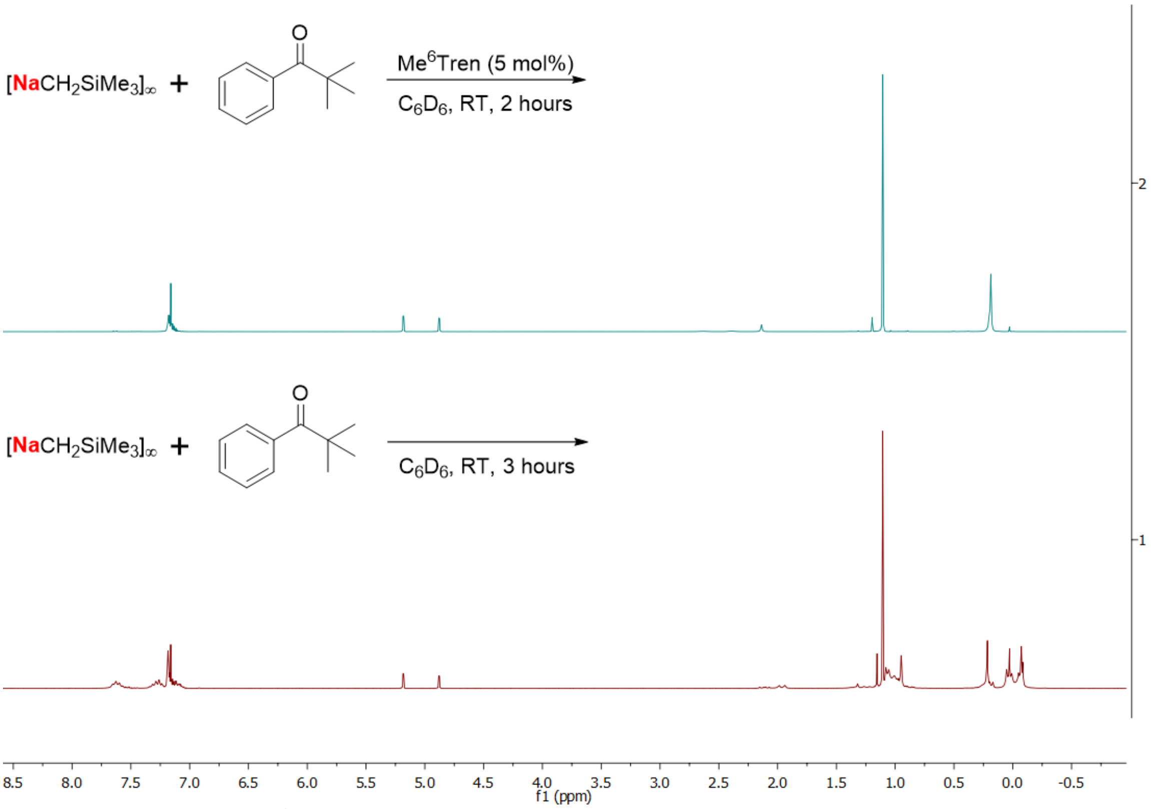

Figure S50: Stacked ^{1}H NMR (d_6-benzene, 25 ˚C, 300 MHz) of a NMR scale reaction between [$NaCH_2SiMe_3$]$_\infty$, 2,2,2-trimethylacetophenone and 5 mol% Me_6Tren (top: cyan) and control reaction between [$NaCH_2SiMe_3$]$_\infty$ and 2,2,2-trimethylacetophenone (bottom: red).

$[NaCH_2SiMe_3]_\infty$ and 2-adamantanone (2f), 5 mol% Me_6Tren catalytic reaction:
A solution of Me_6Tren in C_6D_6 (0.4755 g, 0.002 mmol, 0.5 ml, $[Cat]_0$ = 4 mM) was added to 2-adamantanone (**2f**) (0.0060 g, 0.04 mmol, $[Sub]_0$ = 80 mM). The solution was added at room temperature to $[NaCH_2SiMe_3]_\infty$ (0.0044 g, 0.04 mmol). The resulting solution was transferred to a J Young NMR tube. ^{1}H showed complete conversion to the olefin within 2 hours.

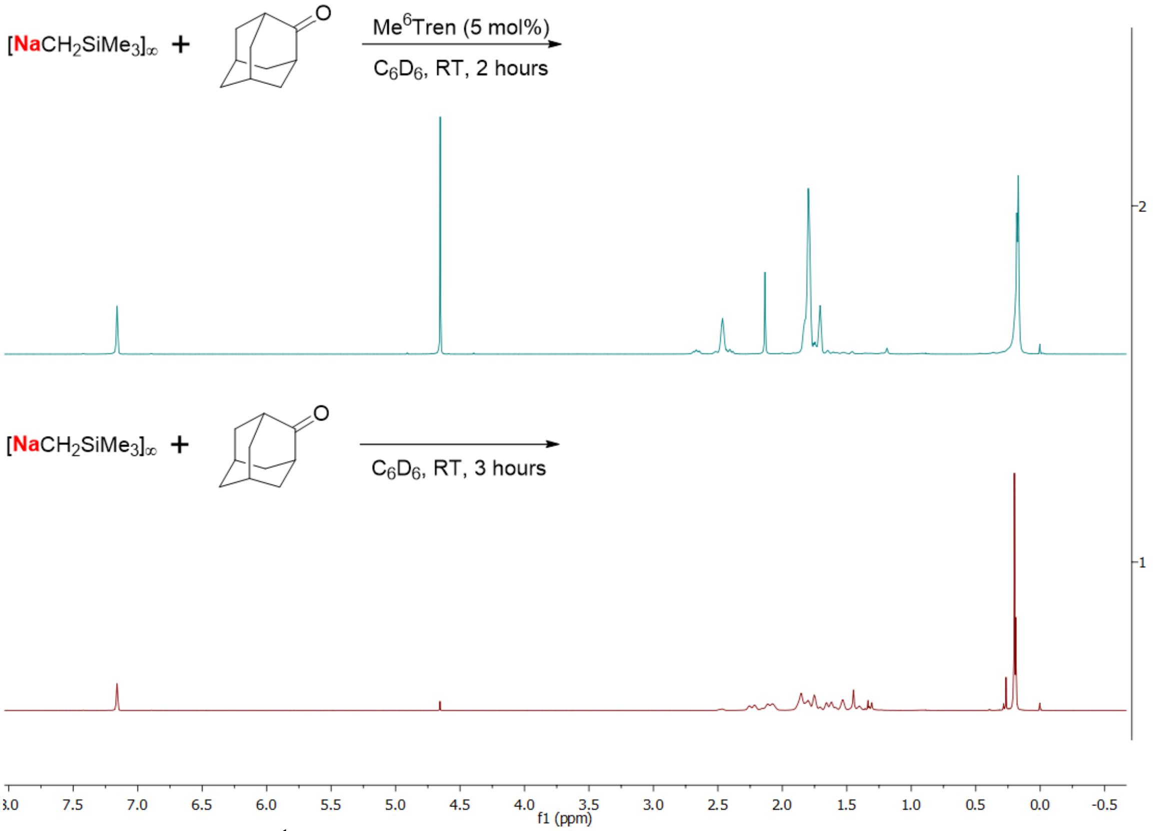

Figure S51: Stacked ^{1}H NMR (d_6-benzene, 25 ˚C, 300 MHz) of a NMR scale reaction between $[NaCH_2SiMe_3]_\infty$, 2-adamantanone and 5 mol% Me_6Tren (top: cyan) and control reaction between $[NaCH_2SiMe_3]_\infty$ and 2-adamantanone (bottom: red).

$[NaCH_2SiMe_3]_\infty$ and 9-anthracenecarboxaldehyde (2h), 5 mol% Me_6Tren catalytic reaction:

A solution of Me_6Tren in C_6D_6 (0.4755 g, 0.002 mmol, 0.5 ml, $[Cat]_0$ = 4 mM) was added to 9-anthracenecarboxaldehyde (**2h**) (0.0082 g, 0.04 mmol, $[Sub]_0$ = 80 mM). The solution was added at room temperature to $[NaCH_2SiMe_3]_\infty$ (0.0044 g, 0.04 mmol). The resulting solution was transferred to a J Young NMR tube. ^{1}H NMR showed little conversion after 2 hours at room temperature but showed complete conversion to the olefin within 18 hours after heating to 60 ˚C.

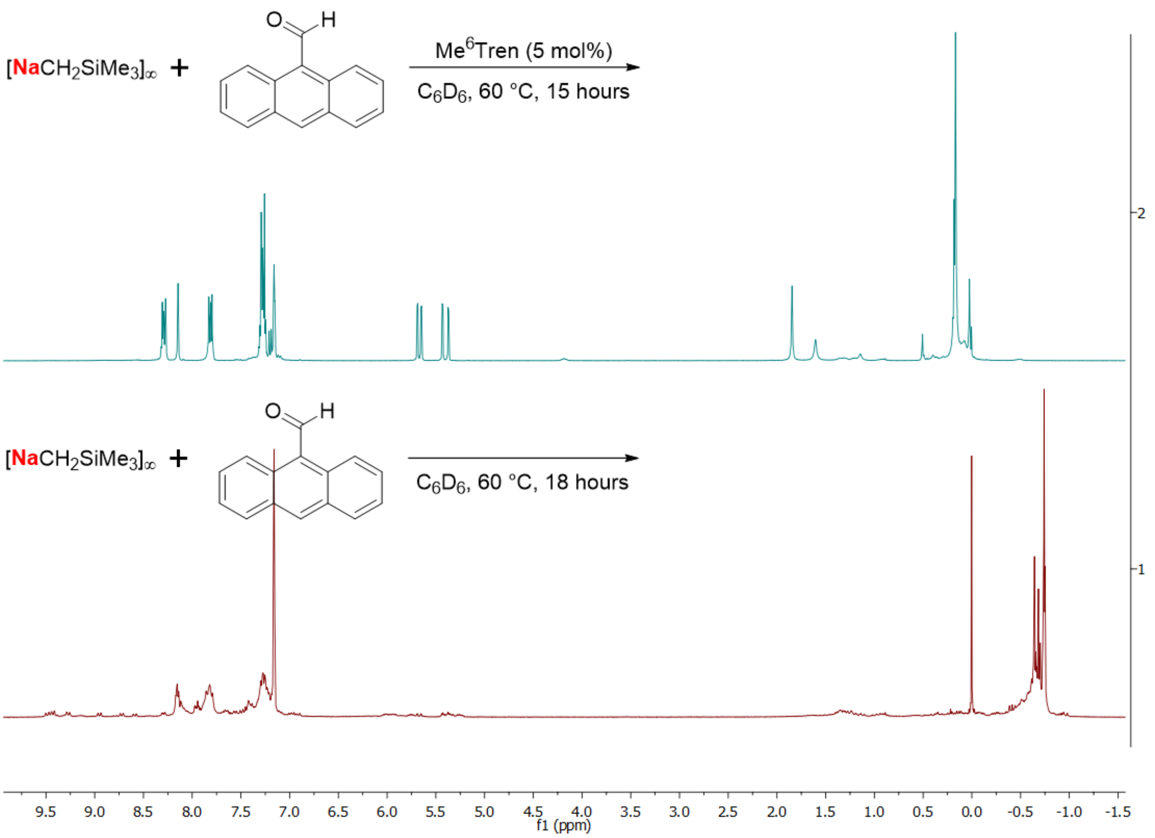

Figure S52: Stacked ^{1}H NMR (d_6-benzene, 25 ˚C, 300 MHz) of a NMR scale reaction between $[NaCH_2SiMe_3]_\infty$, 9-anthracenecarboxaldehyde and 5 mol% Me_6Tren (top: cyan) and control reaction between $[NaCH_2SiMe_3]_\infty$ and 9-anthracenecarboxaldehyde (bottom: red).

[$NaCH_2SiMe_3$]$_\infty$ and 2,2,2-trifluoroacetophenone (2i), 5 mol% Me_6Tren catalytic reaction:

A solution of Me_6Tren in C_6D_6 (0.4755 g, 0.002 mmol, 0.5 ml, $[Cat]_0$ = 4 mM) was added to 2,2,2-trifluoroacetophenone (**2i**) (0.0070 g, 0.04 mmol, $[Sub]_0$ = 80 mM). The solution was added at room temperature to [$NaCH_2SiMe_3$]$_\infty$ (0.0044 g, 0.04 mmol). The resulting solution was transferred to a J Young NMR tube. ^{1}H NMR showed >95% conversion after 3 days at room temperature.

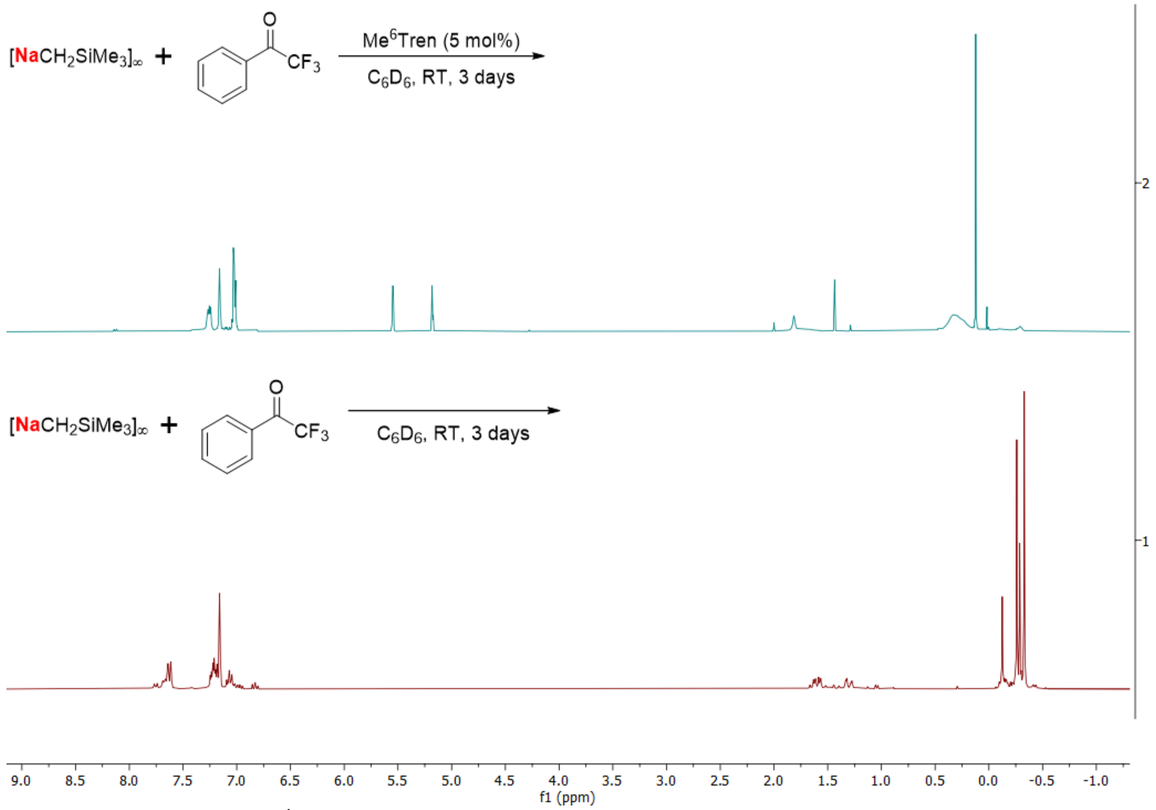

Figure S53: Stacked ^{1}H NMR (d_6-benzene, 25 ˚C, 300 MHz) of a NMR scale reaction between [$NaCH_2SiMe_3$]$_\infty$, 2,2,2-trifluoroacetophenone and 5 mol% Me_6Tren (top: cyan) and control reaction between [$NaCH_2SiMe_3$]$_\infty$ and 2,2,2-trifluoroacetophenone (bottom: red).

[$NaCH_2SiMe_3]_\infty$ and 3,3',5,5'-tetrakis(trifluoromethyl)benzophenone (2j), 5 mol% Me_6Tren catalytic reaction:

A solution of Me_6Tren in C_6D_6 (0.4755 g, 0.002 mmol, 0.5 ml, $[Cat]_0$ = 4 mM) was added to 3,3',5,5'-tetrakis(trifluoromethyl)benzophenone (**2j**) (0.0182g, 0.04 mmol, $[Sub]_0$ = 80 mM). The solution was added at room temperature to $[NaCH_2SiMe_3]_\infty$ (0.0044 g, 0.04 mmol). The resulting solution was transferred to a J Young NMR tube. ^{1}H NMR showed >75% conversion after 3 days at room temperature.

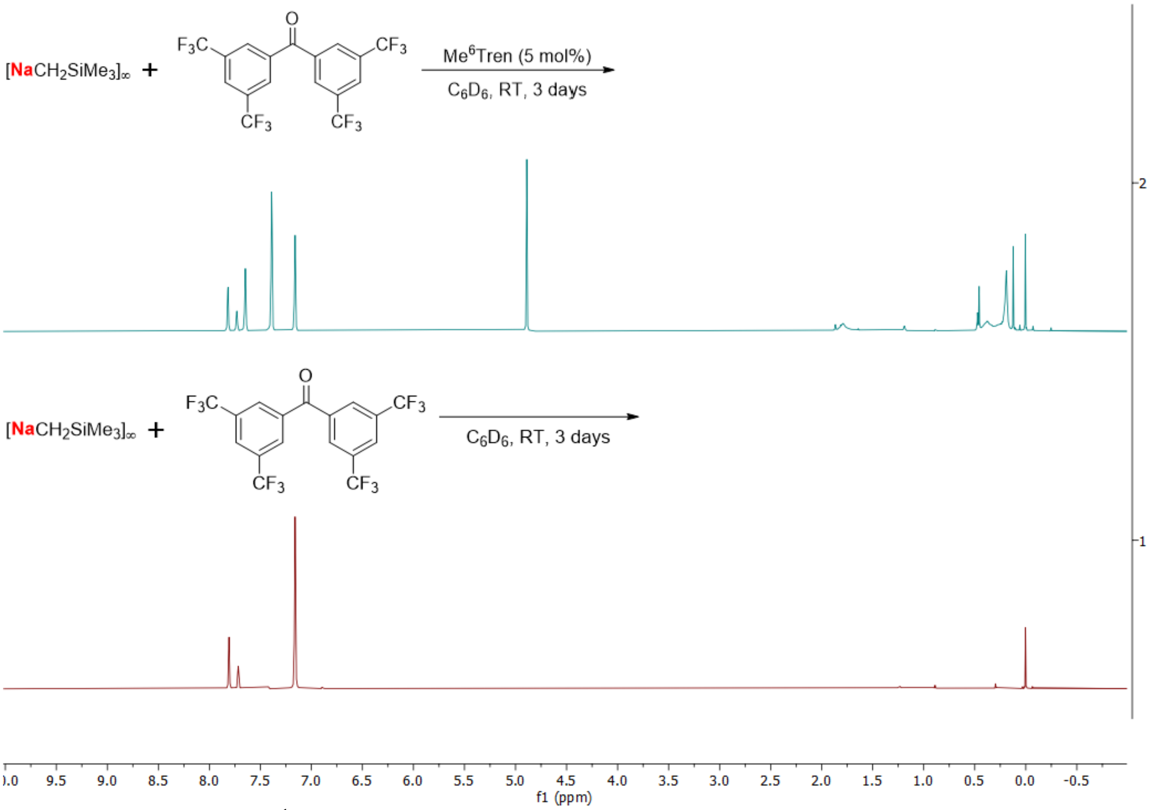

Figure S54: Stacked ^{1}H NMR (d_6-benzene, 25 ˚C, 300 MHz) of a NMR scale reaction between $[NaCH_2SiMe_3]_\infty$, 3,3',5,5'-tetrakis(trifluoromethyl)benzophenone and 5 mol% Me_6Tren (top: cyan) and control reaction between $[NaCH_2SiMe_3]_\infty$ and 3,3',5,5'-tetrakis(trifluoromethyl)benzophenone (bottom: red).

$[NaCH_2SiMe_3]_\infty$ and 4,4′-bis(dimethylamino)benzophenone (2k, Michler′s ketone), 5 mol% Me_6Tren catalytic reaction:

A solution of Me_6Tren in C_6D_6 (0.4755 g, 0.002 mmol, 0.5 ml, $[Cat]_0$ = 4 mM) was added to 4,4′-bis(dimethylamino)benzophenone (**2k**) (0.0107 g, 0.04 mmol, $[Sub]_0$ = 80 mM). The solution was added at room temperature to $[NaCH_2SiMe_3]_\infty$ (0.0044 g, 0.04 mmol). The resulting solution was transferred to a J Young NMR tube. ^{1}H NMR showed little conversion after 2 hours at room temperature but showed complete conversion to the olefin within 18 hours after heating to 60 ˚C.

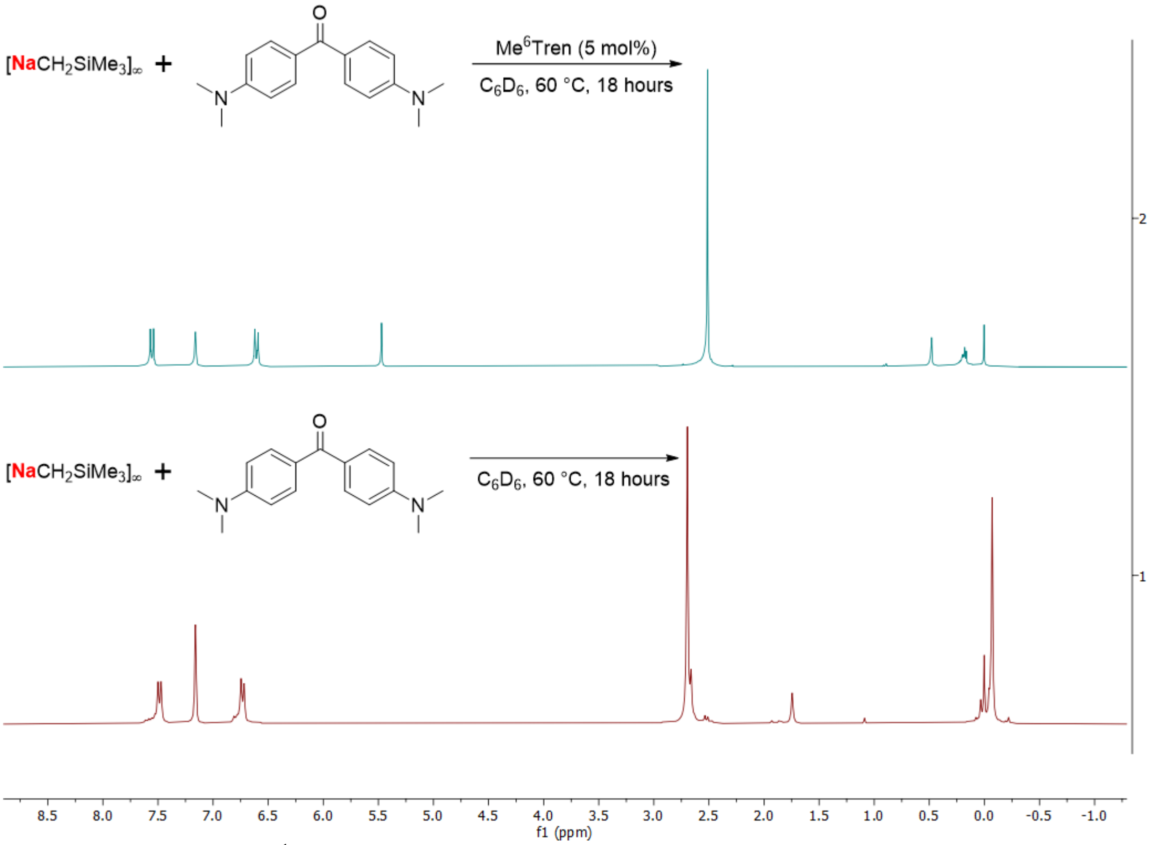

Figure S55: Stacked ^{1}H NMR (d_6-benzene, 25 ˚C, 300 MHz) of a NMR scale reaction between $[NaCH_2SiMe_3]_\infty$, 4,4′-bis(dimethylamino)benzophenone and 5 mol% Me_6Tren (top: cyan) and control reaction between $[NaCH_2SiMe_3]_\infty$ and 4,4′-bis(dimethylamino)benzophenone (bottom: red).

1.7 Kinetic NMR

$[NaCH_2SiMe_3]_\infty$ (0.0088g, 0.08 mmol), benzophenone (0.0146 g, 0.08 mmol), 0.04 M Me_6Tren solution in C_6D_6 (0.1 ml, 0.0959 g, 0.004 mmol, 0.05 equivalents) and 0.089 M cyclohexane solution in C_6D_6 (0.9 ml, 0.8618 g, 0.08 mmol) were placed into separate vials.

The solution of cyclohexane (^{1}H NMR integration internal standard) in C_6D_6 was added to the solution of Me_6Tren in C_6D_6, which was then added to the benzophenone. The resulting solution was added to the $[NaCH_2SiMe_3]_\infty$ at room temperature and the time of mixing was recorded. The resulting solution was placed in a J Young NMR tube in glove box.

The concentration of **3**-Na was obtained from the area of the signal at 1.44 ppm (2H, CH_2Si), and the concentration of 1,1-diphenylethylene from the area of the signal at 5.36 ppm (2H, =CH_2), using cyclohexane (12H, 1.40 ppm) as the internal standard.

The MestReNova™ NMR software was used to process the spectrum and obtain accurate integrations.

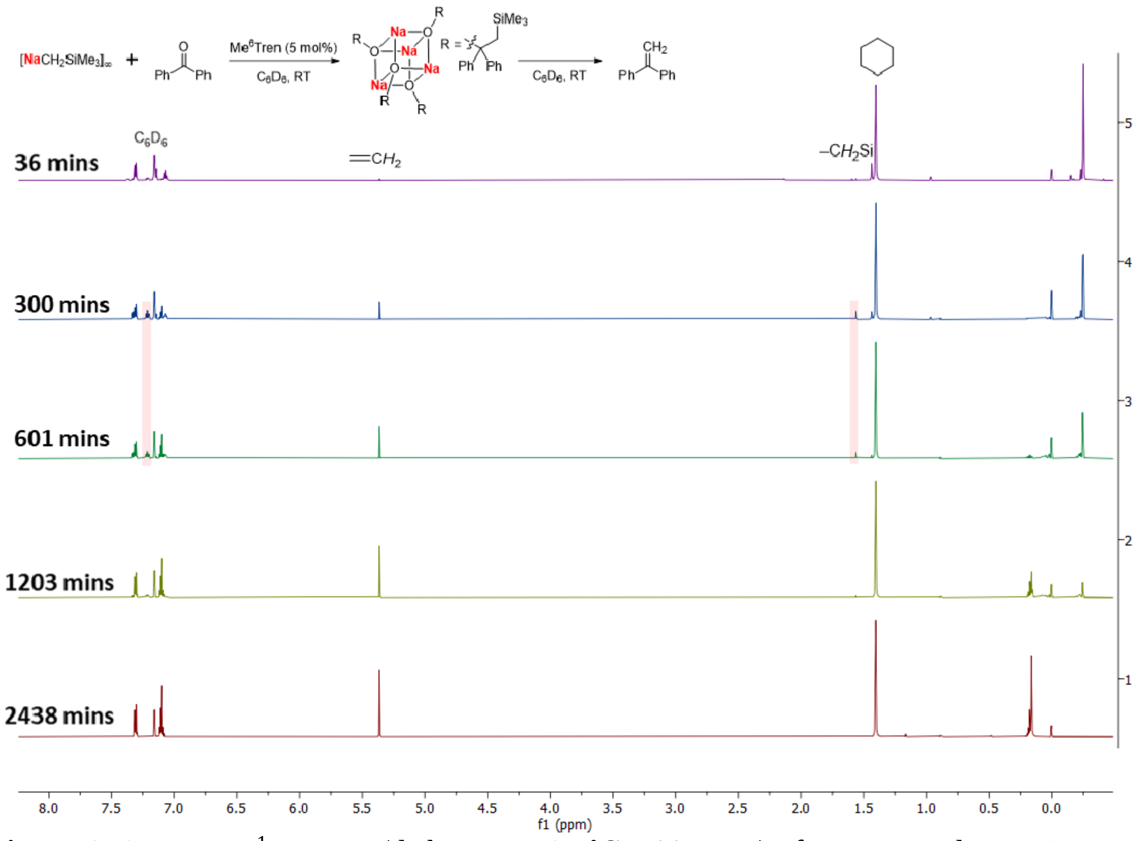

Figure S56: Kinetic ^{1}H NMR (d_6-benzene, 25 ˚C, 700 MHz) of a NMR scale reaction between $[NaCH_2SiMe_3]_\infty$, benzophenone and 5 mol% Me_6Tren. Internal standard (I) = cyclohexane. $[C]_0$ = 0.08 M, [Cat] = 0.004 M, [I] = 0.08 M. Signals that possibly represent an intermediate are highlighted in pink.

1.8 Diffusion Ordered Spectroscopy (DOSY NMR) Details

^{1}H DOSY of [$NaCH_2SiMe_3$]$_\infty$ in C_6D_6

^{1}H DOSY NMR experiment was performed on a Bruker 700 Avance III HD NMR spectrometer using a TCI cryoprobe with a maximum gradient strength of 60 G cm^{-1}. Sample was prepared using 7.5 mM of $NaCH_2SiMe_3$ and an equimolar ratio of adamantane in C_6D_6.

The method of using DOSY to estimate MW as outlined in Stalke papers was followed[18,19]. The Bruker dstebpgp3s pulse sequence was used with a diffusion time Δ of 0.1 s and p30 (δ/2) pf 600 μs. Diffusion coefficients were calculated and the DOSY plot was generated using Bruker's dynamic centre.

We employed a relaxation delay time (d_1) of 5 seconds and acquired 16 spectra for each DOSY experiment leading to a total acquisition time of 32 minutes. The average value of D = 7.44 E-10 m^2s^{-1}.

MW calculation using the external calibration curves (ECCs) method:
Assuming Dissipated Sphere and Ellipsoid = MW = 416.87.
This would correspond to approximately a tetramer (4 x 110.2 = 440.8).

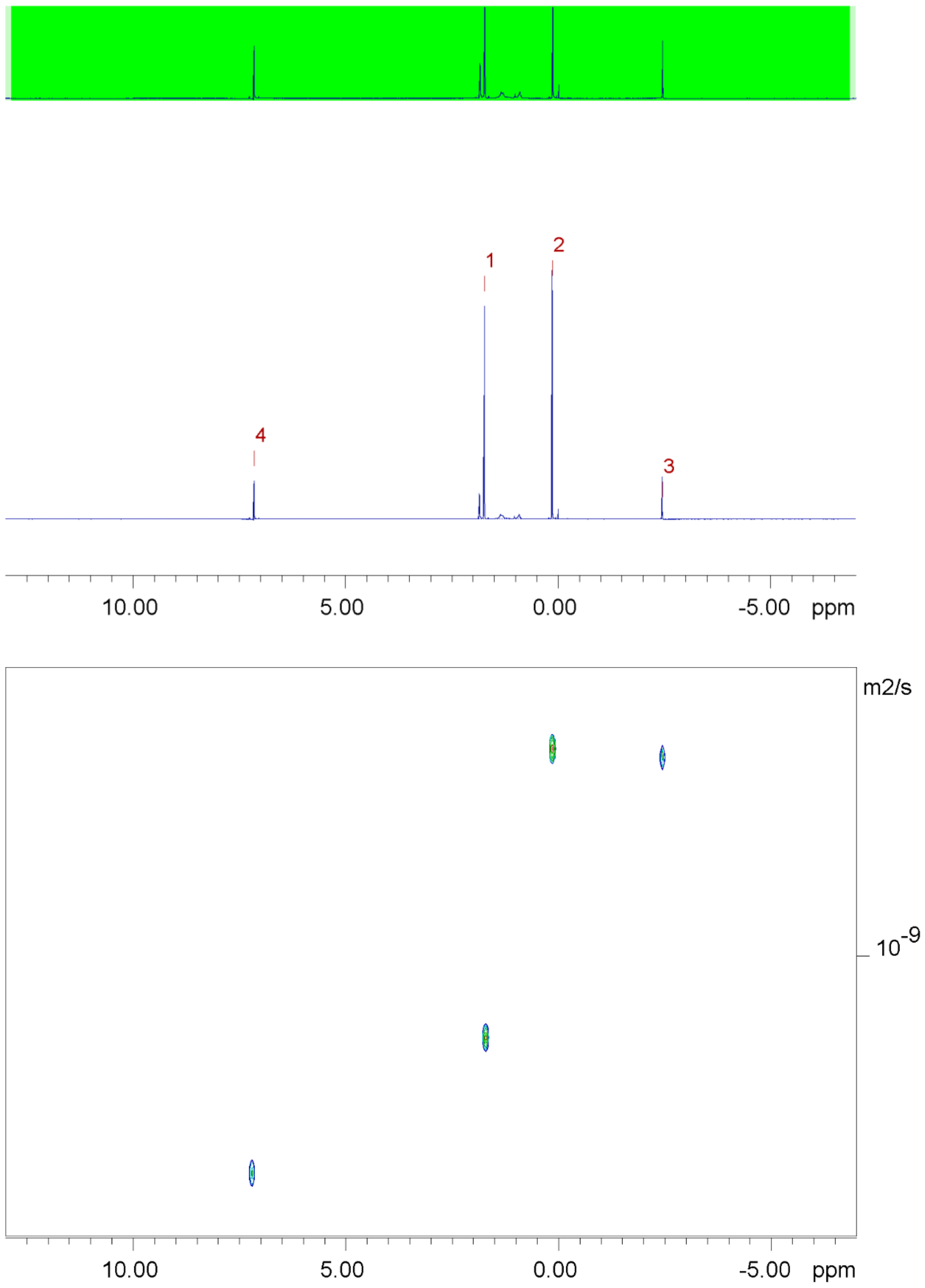

Figure S57: ^{1}H DOSY NMR (d_6-benzene, 25 ˚C, 700 MHz) of $[NaCH_2SiMe_3]_\infty$ and the internal standard adamantane.

^{1}H DOSY of [Na(CH_2SiMe_3)(Me_6Tren)] (1-Na) in C_6D_{12}

^{1}H DOSY NMR experiment was performed on a Bruker 700 Avance III HD NMR spectrometer using a TCI cryoprobe with a maximum gradient strength of 60 G cm^{-1}. Sample was prepared using 15 mM of [Na(CH_2SiMe_3)(Me_6Tren)] (**1**-Na) and an equimolar ratio of adamantane in C_6D_{12}.

The method of using DOSY to estimate MW as outlined in Stalke papers was followed[18,19]. The Bruker dstebpgp3s pulse sequence was used with a diffusion time Δ of 0.1 s and p30 (δ/2) pf 600 μs. Diffusion coefficients were calculated with the T1/T2 software in Topspin and the DOSY plot was generated using Bruker's dynamic centre.

We employed a relaxation delay time (d_1) of 5 seconds and acquired 16 spectra for each DOSY experiment leading to a total acquisition time of 32 minutes. The average value of D = 5.9 E-10 m^2s^{-1}

MW calculation using the external calibration curves (ECCs) method:
Assuming Expanded Disc = MW = 333.12.
Assuming Dissipated Sphere and Ellipsoid = MW = 355.57
The actual value is 340.61. The complex remains a monomer in solution.

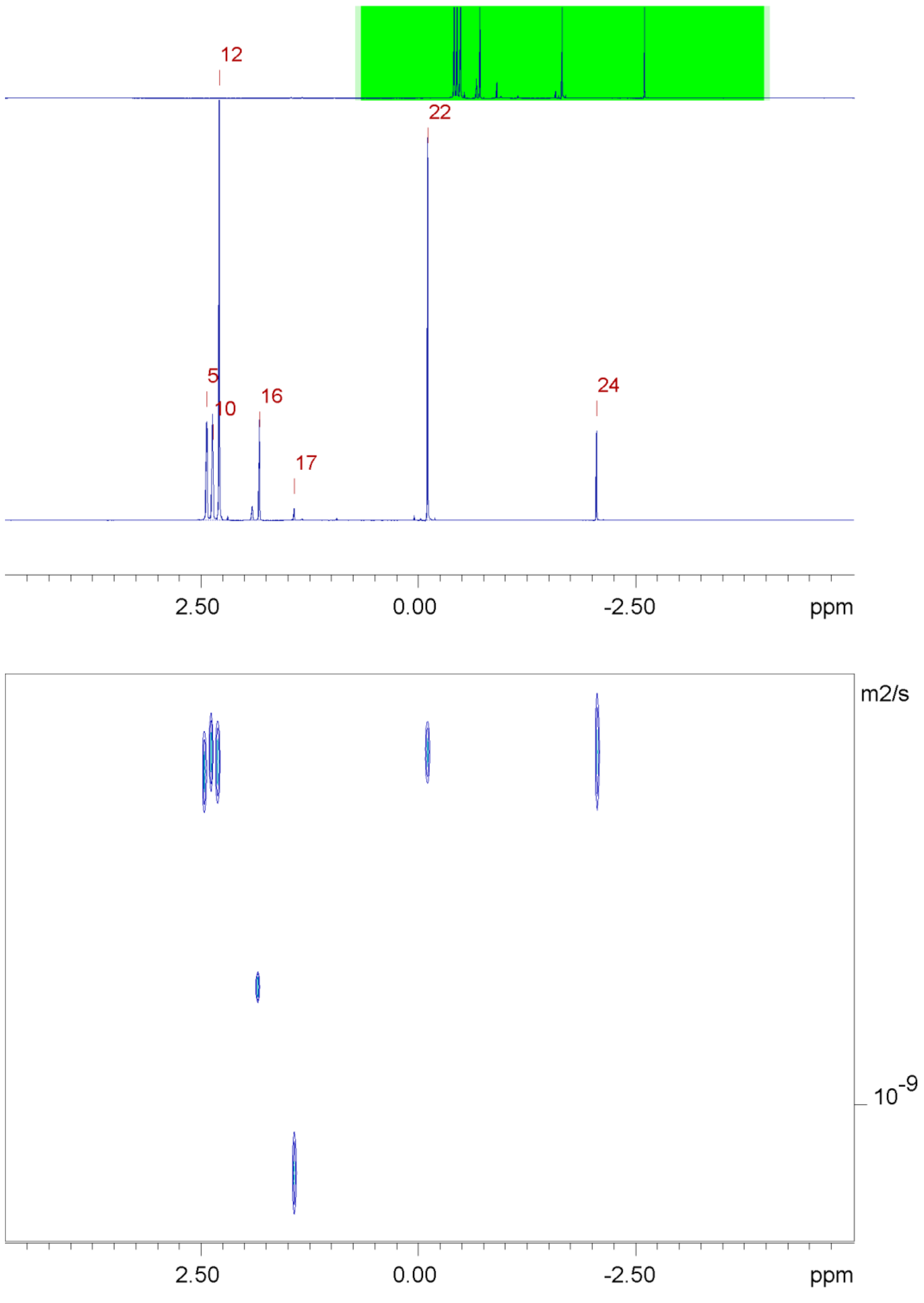

Figure S58: ^{1}H DOSY NMR (d_{12}-cyclohexane, 25 ˚C, 700 MHz) of [Na(CH_2SiMe_3)(Me_6Tren)] (**1**-Na) and the internal standard adamantane.

1.9 Single-crystal X-ray diffraction (SCXRD) Details

Crystal structures data for **1-Na** and **5** were collected on a XtaLAB Synergy, Dualflex, HyPix-Arc 100 diffractometer equipped with an micro-focus sealed X-ray tube ($\lambda_{Cu K\alpha}$ = 1.54184 Å) and an Oxford Cryosystems CryostreamPlus open-flow N_2 cooling device. Cell refinement, data collection and data reduction were undertaken via software CrysAlisPro 1.171.42.63a (Rigaku OD, 2022). Intensities were corrected for absorption using CrysAlisPro 1.171.42.63a (Rigaku Oxford Diffraction, 2022) Analytical numeric absorption correction using a multifaceted crystal model based on expressions derived by R.C. Clark & J.S. Reid. (Clark, R. C. & Reid, J. S. (1995). Acta Cryst. A51, 887-897) Empirical absorption correction using spherical harmonics, implemented in SCALE3 ABSPACK scaling algorithm. Using Olex2 (Dolomanov, 2009), the structure was solved using SHELXT 2014/5 (Sheldrick, 2014) and refined by XL (Sheldrick, 2008).

Crystal structure data for $[NaCH_2SiMe_3]_\infty$, **3-Na** and **6** were collected on a XtaLAB Synergy, Single source at home/near, HyPix-Arc 100 diffractometer equipped with an fine-focus sealed X-ray tube ($\lambda_{Cu K\alpha}$ = 1.54184 Å) and an Oxford Cryosystems CryostreamPlus open-flow N_2 cooling device. Cell refinement, data collection and data reduction were undertaken via software CrysAlisPro 1.171.42.60a (Rigaku OD, 2022). Intensities were corrected for absorption using CrysAlisPro 1.171.42.60a (Rigaku Oxford Diffraction, 2022) Analytical numeric absorption correction using a multifaceted crystal model based on expressions derived by R.C. Clark & J.S. Reid. (Clark, R. C. & Reid, J. S. (1995). Acta Cryst. A51, 887-897) Empirical absorption correction using spherical harmonics, implemented in SCALE3 ABSPACK scaling algorithm. Using Olex2 (Dolomanov, 2009), the structure was solved using SHELXT 2014/5 (Sheldrick, 2014) and refined by XL (Sheldrick, 2008).

Compound	**1-Na**	$[NaCH_2SiMe_3]_\infty$	**5**
Empirical formula	$C_{16}H_{41}N_4NaSi$	$C_4H_{11}NaSi$	$C_{39}H_{108}N_2Na_{9.5}O_{10}Si_{10}$
Formula weight	340.61	110.21	1264.57
Temperature/K	150.0(2)	150.0(2)	150.0(2)
Crystal system	monoclinic	monoclinic	trigonal
Space group	$P2_1/c$	I2/a	R3m
a/Å	10.0622(2)	12.7227(3)	18.5450(2)
b/Å	14.7010(2)	12.4636(3)	18.5450(2)
c/Å	16.4066(3)	17.9667(4)	53.7108(9)
α/°	90	90	90
β/°	106.019(2)	92.521(2)	90
γ/°	90	90	120
Volume/$Å^3$	2332.70(7)	2846.23(12)	15997.3(4)
Z	4	16	6
$\rho_{calc}g/cm^3$	0.97	1.029	0.788
μ/mm^{-1}	1.075	2.517	1.781
F(000)	760	960	4083
Crystal size/mm^3	0.2 × 0.19 × 0.15	0.12 × 0.09 × 0.02	0.56 × 0.44 × 0.26
Radiation	Cu Kα (λ = 1.54184)	Cu Kα (λ = 1.54184)	Cu Kα (λ = 1.54178)
2Θ range for data collection/°	8.222 to 156.62	8.638 to 154.126	5.744 to 157.792
Index ranges	-12 ≤ h ≤ 12, -18 ≤ k ≤ 7, -19 ≤ l ≤ 20	-11 ≤ h ≤ 15, -15 ≤ k ≤ 14, -22 ≤ l ≤ 22	-23 ≤ h ≤ 6, -16 ≤ k ≤ 22, -65 ≤ l ≤ 63
Reflections collected	17232	11850	19069
Independent reflections	4683 [R_{int} = 0.0278, R_{sigma} = 0.0260]	2821 [R_{int} = 0.0486, R_{sigma} = 0.0405]	6720 [R_{int} = 0.0248, R_{sigma} = 0.0370]
Data/restraints/parameters	4683/0/209	2821/0/127	6720/562/333
Goodness-of-fit on F^2	1.059	1.038	1.056
Final R indexes [I>=2σ (I)]	R_1 = 0.0350, wR_2 = 0.0948	R_1 = 0.0353, wR_2 = 0.0807	R_1 = 0.0335, wR_2 = 0.0896
Final R indexes [all data]	R_1 = 0.0425, wR_2 = 0.0994	R_1 = 0.0493, wR_2 = 0.0862	R_1 = 0.0344, wR_2 = 0.0906
Largest diff. peak/hole / e $Å^{-3}$	0.27/-0.28	0.29/-0.27	0.24/-0.20

Table S1. Crystal Structure Refinement Details for Complexes **1**-Na, $[NaCH_2SiMe_3]_\infty$, and **5.**

Compound	**3-Na**	**6**
Empirical formula	$C_{17}H_{21}NaOSi$	$C_{27}H_{41}N_4NaO_2$
Formula weight	292.42	476.63
Temperature/K	150.0(2)	150.0(2)
Crystal system	monoclinic	monoclinic
Space group	$P2_1/c$	$P2_1/n$
a/Å	13.01390(10)	9.44329(14)
b/Å	22.4368(2)	15.8167(3)
c/Å	23.2912(2)	18.7038(3)
α/°	90	90
β/°	94.4530(10)	98.7199(15)
γ/°	90	90
Volume/Å^3	6780.27(10)	2761.35(8)
Z	16	4
ρ_{calc}g/cm^3	1.146	1.146
μ/mm^{-1}	1.406	0.709
F(000)	2496	1032
Crystal size/mm^3	0.11 × 0.04 × 0.02	0.18 × 0.12 × 0.04
Radiation	Cu Kα (λ = 1.54184)	Cu Kα (λ = 1.54184)
2Θ range for data collection/°	7.614 to 154.75	7.356 to 155.498
Index ranges	$-16 \le h \le 16, -28 \le k \le 16, -27 \le l \le 29$	$-11 \le h \le 11, -6 \le k \le 19, -23 \le l \le 23$
Reflections collected	53601	17573
Independent reflections	13468 [R_{int} = 0.0311, R_{sigma} = 0.0286]	17573 [R_{int} = 0.031, R_{sigma} = 0.0214]
Data/restraints/parameters	13468/0/733	17573/423/314
Goodness-of-fit on F^2	1.039	1.066
Final R indexes [I>=2σ (I)]	R_1 = 0.0335, wR_2 = 0.0825	R_1 = 0.0546, wR_2 = 0.1470
Final R indexes [all data]	R_1 = 0.0437, wR_2 = 0.0875	R_1 = 0.0630, wR_2 = 0.1542
Largest diff. peak/hole / e Å^{-3}	0.28/-0.33	0.51/-0.26

Table S2. Crystal Structure Refinement Details for Complexes **3**-Na and **6**.

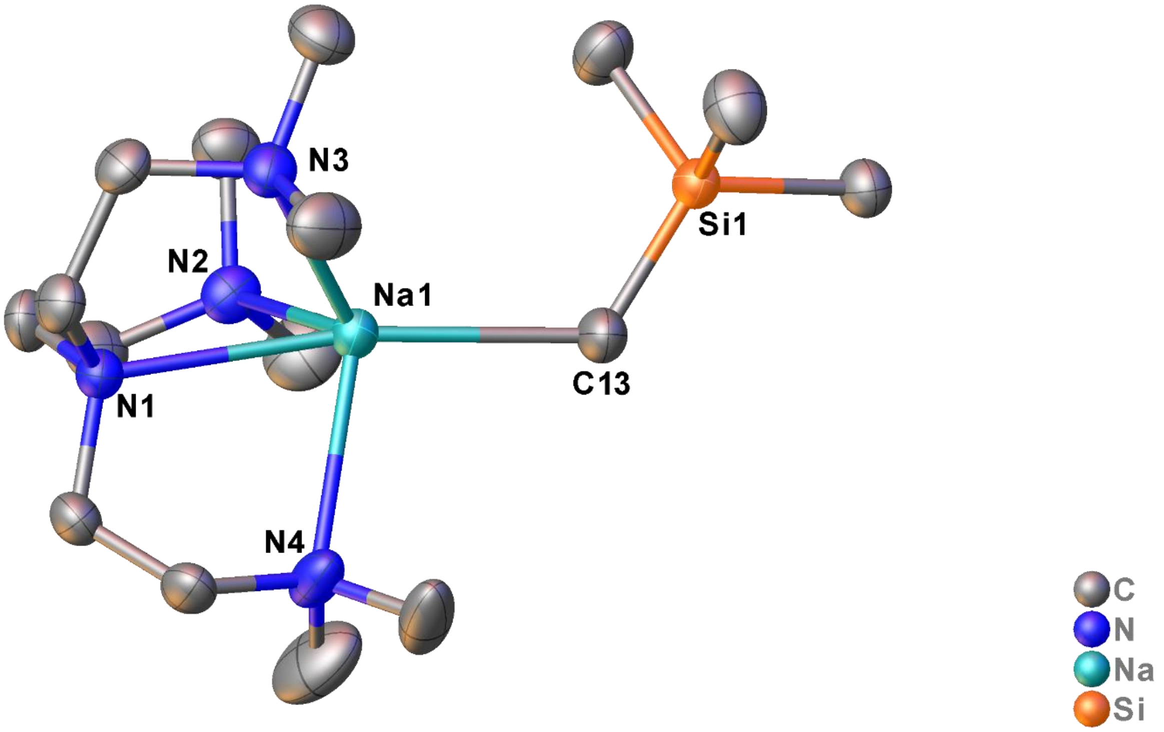

Figure S59: Molecular structure of **1**-Na. Key bond lengths (Å): Na1–C13 2.5054(14), Si1–C13 1.7938(14), Na1–N1 2.6137(11), Na1–N2 2.5232(12), Na1–N3 2.5588(12), Na1–N4 2.5484(13). Hydrogen atoms are omitted for clarity.

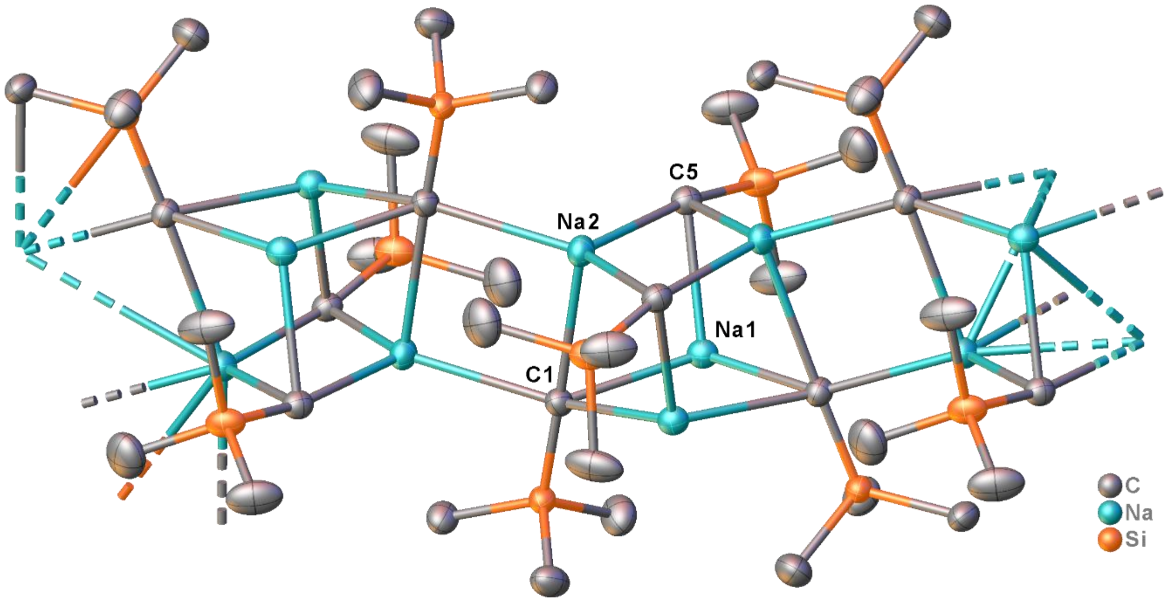

Figure S60: Molecular structure of. $[NaCH_2SiMe_3]_\infty$. Key bond lengths (Å): Na1–C1 2.642(2), Na1–C5 2.577(2), Na2–C1 2.825(2), Na2–C5 2.601(2). Hydrogen atoms are omitted for clarity. This is a polymorph of the previously reported structure.[3]

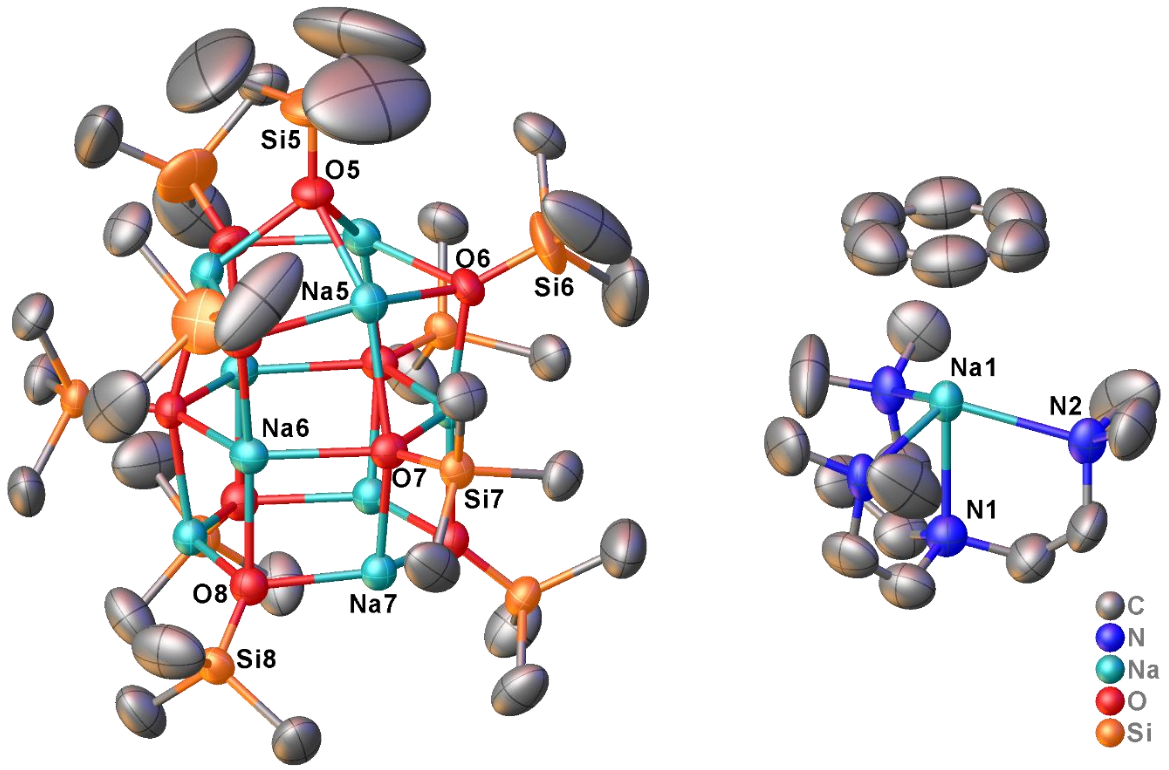

Figure S61: Molecular structure of **5**. Key bond lengths (Å): Na1–N1 2.426(7), Na1–N2 2.479(5), Na5–O5 2.279(3), Na5–O6 2.343(2), Na5–O7 2.556(3), Si5–O5 1.550(5), Na6–O6 2.241(3), Si6–O6 1.564(3), Na6–O7 2.3648(14), Na6–O8 2.356(3), O7–Si7 1.641(3), Na7–O7 2.317(3), Na7–O8 2.2166(16), Si8–O8 1.599(3). Hydrogen atoms are omitted and only heteroatoms are labelled for clarity. Only the crystallographically independent atoms are labelled. Various parts of both anion and cation in the structure of **5** are disordered across a symmetry element. The occupancies of the parts were constrained to sum to unity and, where appropriate, restraints were applied to imbue a more correct geometry. One of the spherical cations appears to be 'tumbling' and is hence likely to manifest as being disordered over multiple orientations. This could not be modelled in a satisfactory way hence the electron density associated with this cation was treated using the Olex2 solvent mask. This masking of the cation is the reason for the perceived discrepancy in the formula shown in Table S1.

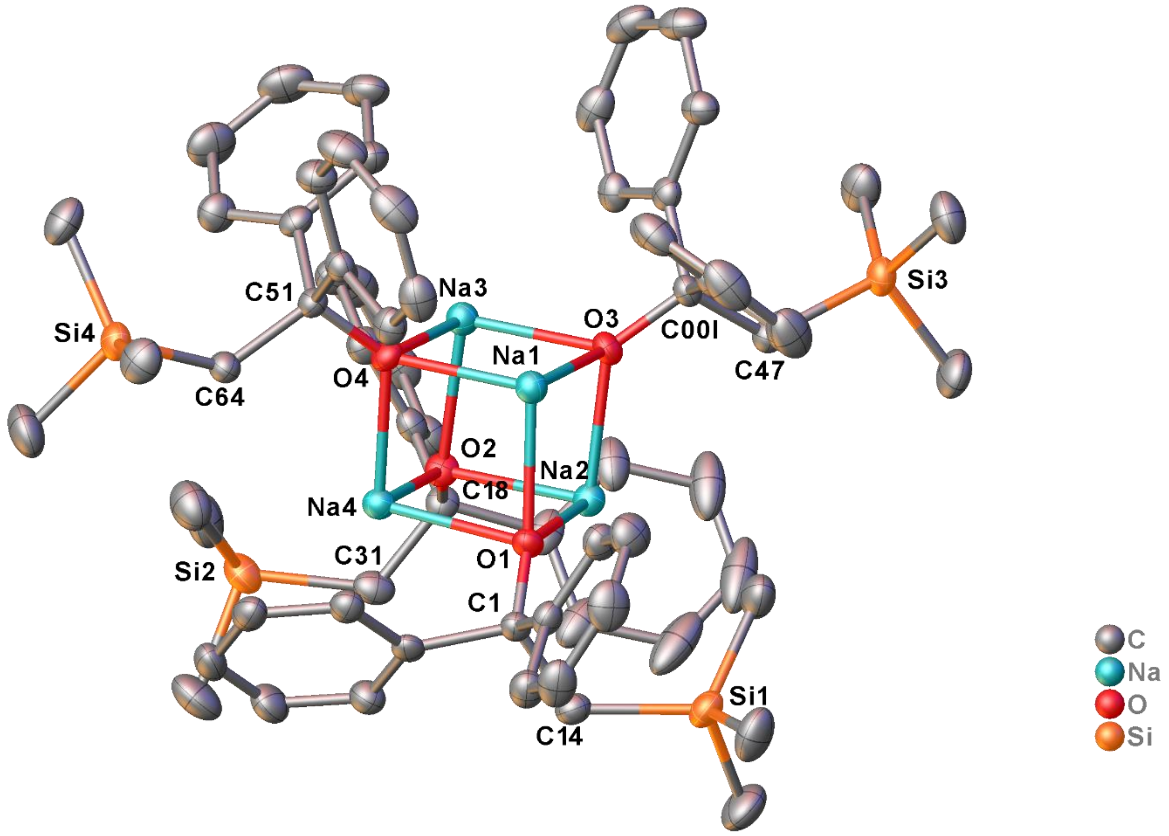

Figure S62: Molecular structure of **3**-Na. Key bond lengths (Å): Na1–O1 2.2657(11), Na1–O3 2.2798(10), Na1–O4 2.2452(11), Na2–O1 2.2483(10), Na2–O2 2.3647(11), Na2–O3 2.2328(11), Na3–O2 2.2986(11), Na3–O3 2.2445(11), Na3–O4 2.2571(10), Na4–O1 2.4219(10), Na4–O2 2.2124(10), Na4–O4 2.2262(10). O1–C1 1.3919(16), C1–C14 1.5577(19), Si1–C14 1.8834(15), O2–C18 1.3907(16), C18–C31 1.556(2), Si2–C31 1.8747(16), O3–C00I 1.3984(16), C00I–C47 1.5532(19), Si3–C47 1.8865(14), O4–C51 1.3963(16), C51–C64 1.5551(18), Si4–C64 1.8847(14). Hydrogen atoms are omitted for clarity.

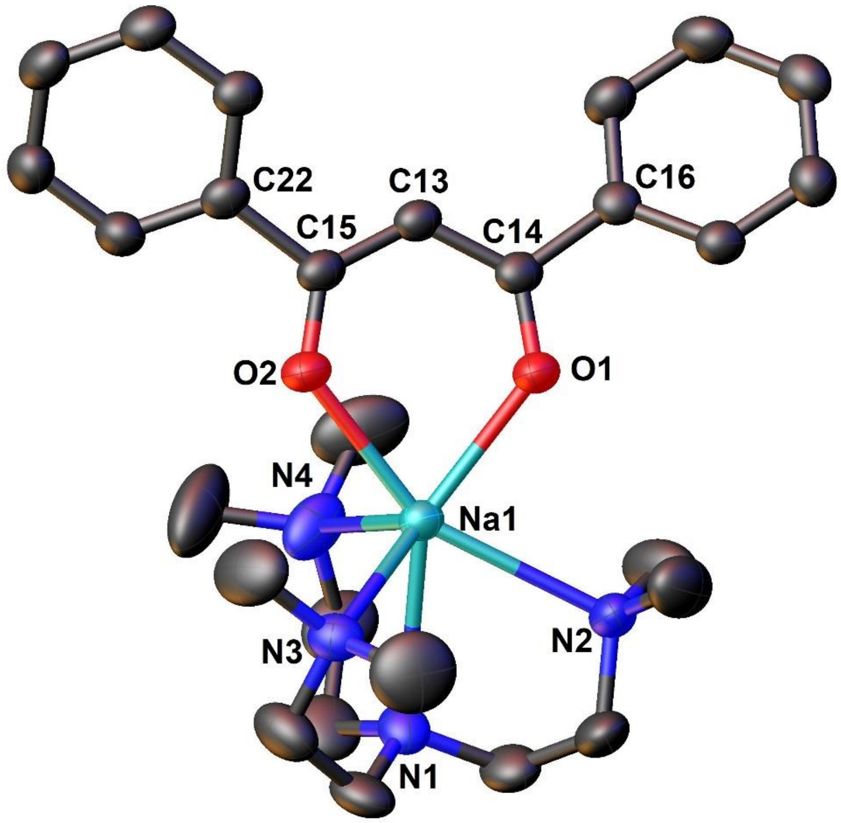

Figure S63: Molecular structure of **6**. Key bond lengths (Å): Na1–O1 2.2977(15), Na1–O2 2.3785(15), Na1–N1 2.7116(18), Na1–N2 2.6411(19), Na1–N3 2.5182(17), Na1–N4 2.518(2), O1–C14 1.253(2), O2–C15 1.256(2), C13–C14 1.409(3), C13–C15 1.403(3), C15–C22 1.513(3), C14–C16 1.514(3). Hydrogen atoms are omitted for clarity.

Section 2. Computational Details

2.1 Reaction Pathway Analyses

2.1.1 Computational Methodology

DFT calculations were run with Gaussian 16 (C.01)[20]. The Na and Si centres were described with the Stuttgart RECPs and associated basis sets[21], and 6-31G** basis sets were used for all other atoms (BS1)[22,23]. A polarization function was also added to Si (ζ_d = 0.284). Initial BP86[24,25] optimizations were performed using the 'grid = ultrafine' option, with all stationary points being fully characterized via analytical frequency calculations as minima (all positive eigenvalues) or a transition state (one negative eigenvalue). All energies were recomputed with a larger basis set featuring 6-311++G** on all atoms. Corrections for the effect of benzene (ε = 2.2706) solvent were run using the polarizable continuum model and BS1[26]. Single-point dispersion corrections to the BP86 results employed Grimme's D3 parameter set with Becke-Johnson damping as implemented in Gaussian[27]. Natural Bonding Orbital (NBO7)[28] analyses were performed on the BP86-optmised geometries using wavefunction files obtained at the BP86/6-311++G** level within Gaussian 16 (C.01).

2.1.2

Table S3. Cluster Size of **3**-M

Computed Parameter	Li	Na
M-O edge / Å	2.025	2.352
M-O diagonal / Å	3.380	4.053
Collision Diameter / Å	14.93	15.26
Molar Volume / bohr^3 mol^{-1}	10452.4	10476.4
/ cm^3 mol^{-1}	932.8	934.9

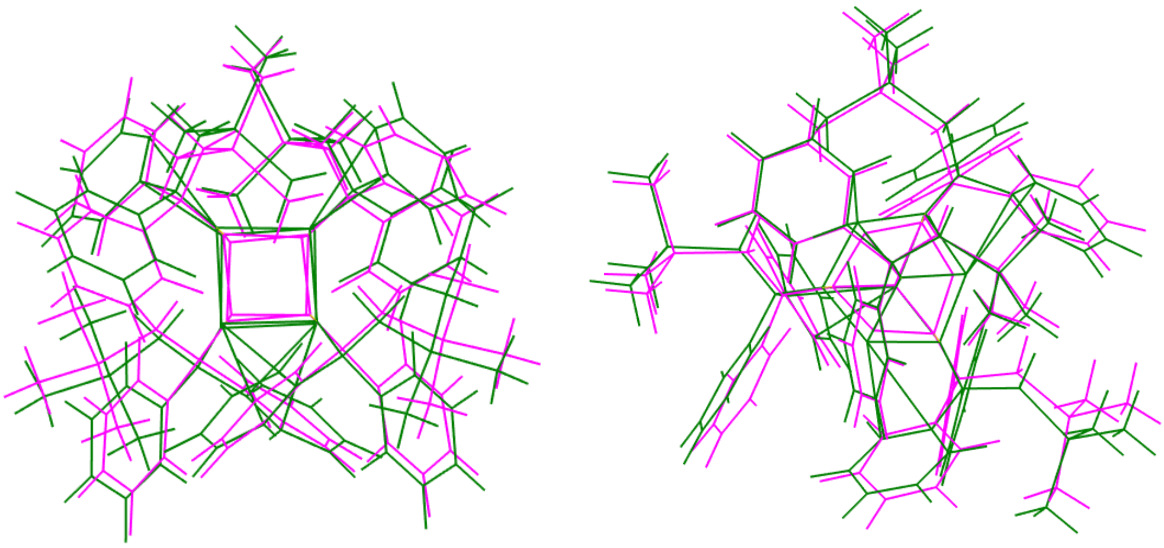

Figure S64: Overlaid structures of the computed structures ((BP86-D3BJ(C6H6)/6-311++G**//BP86/6-31G**&SDDALL) of **3**-Li (Green) and **4**-Na (Magenta) by the four oxygen atoms of the cluster, which gives an RMS of 0.284 Å.

2.1.3 Coordination of Ligand and Overall Reaction Free Energy Dynamics

Table S4. Free Energy preference is for κ^4 ligation of Me_6Tren at both Li and Na, as both values exergonic. Loss of CH_2SiMe_3 from κ^4 **1**-M is endergonic. Overall free energy reaction shows $OSiMe_3$ formation is exergonic, as is the formation of the **3**-M clusters.

	**Li**	**Na**
[κ^{3}N]**M**(CH_2SiMe_3) → [κ^{4}N]**M**(CH_2SiMe_3)	−3.3	−6.6
[κ^{3}N]**Na** → [κ^{4}N]**Na**		−10.2
[κ^{4}N]**M**(CH_2SiMe_3) → [κ^{4}N]**M**	+14.0	+15.6
[κ^{4}N]**M**(CH_2SiMe_3) → [κ^{4}N]**M**($OSiMe_3$)	−58.0	−49.1
[κ^{4}N]**M**(CH_2SiMe_3) → **3**-M	−142.5	−123.0

Table S5. Separated Ion Pair (SIP) Energies

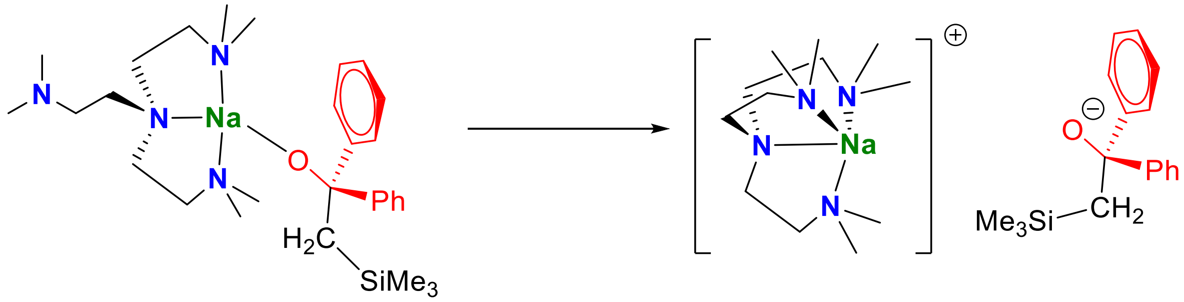

	**Li**	**Na**
[κ^{3}N]**M**(Ph2C(O)CH_2SiMe_3) → [κ^{4}N]**M**$^+$ + (Ph2C(O)CH_2SiMe_3)$^-$	+41.4	+35.3

2.1.4 Potential Energy Surfaces

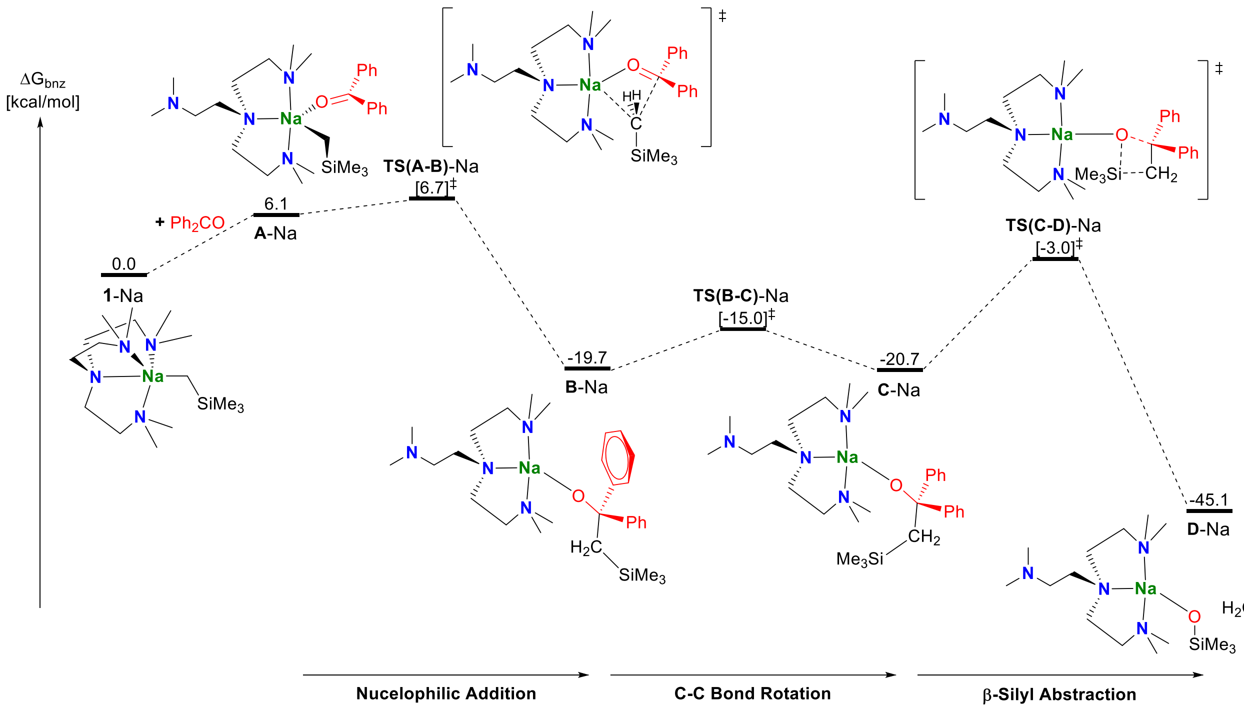

Figure S65. DFT calculated free energy profile (BP86-D3BJ(C_6H_6)/6-311++G**//BP86/6-31G**&SDDALL, in kcal mol^{-1}) for the reaction of **1**-Na with **2a**.

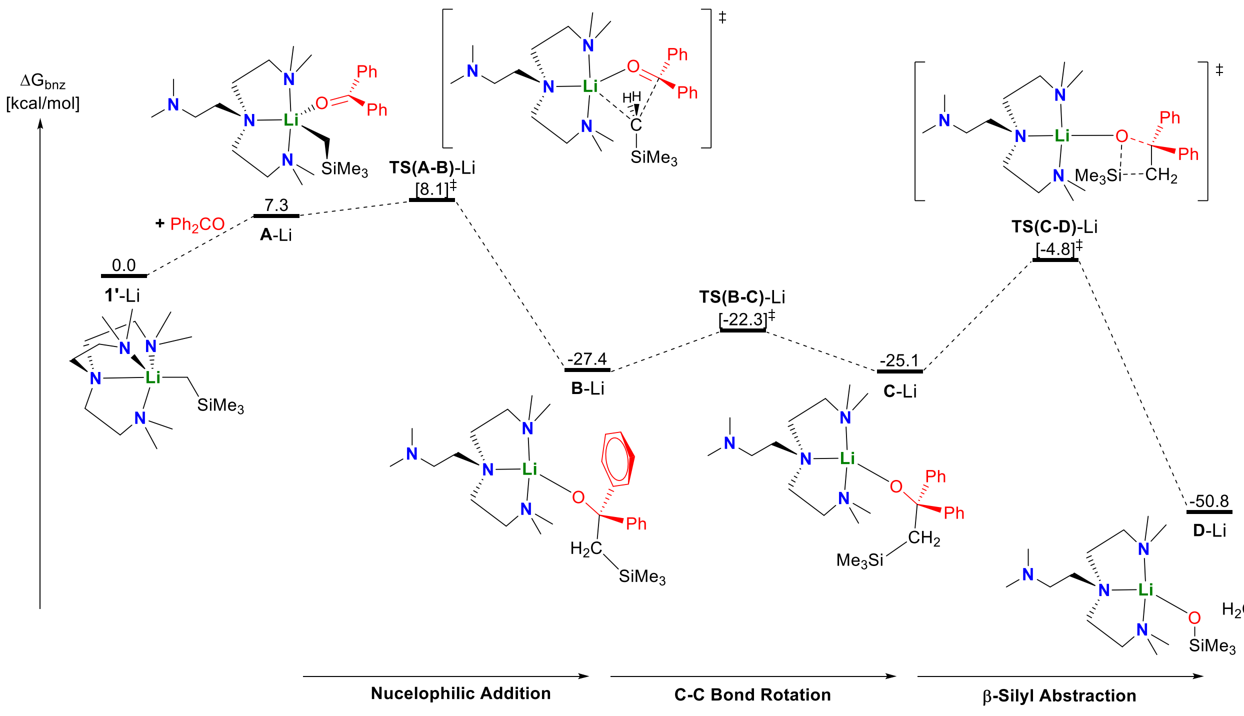

Figure S66. DFT calculated free energy profile (BP86-D3BJ(C_6H_6)/6-311++G**//BP86/6-31G**, in kcal mol^{-1}) for the reaction of **1**-Li with benzophenone.

For both the **1**-Na and **1′**-Li systems a second C–C rotation barrier was found that is counter-clockwise to **TS(B-C)**-M that has been labelled **TS(B-C′)**-M. This is the opposite direction to move between the alkoxide conformers *anti* (**B**-M) and *syn* (**C**-M), which also gives an alternative **C′**-M *gauche* conformer. These **TS(B-C′)**-M barriers are at −12.7 and −21.8 kcal mol^{-1} for **1**-Na and **1′**-Li respectively.

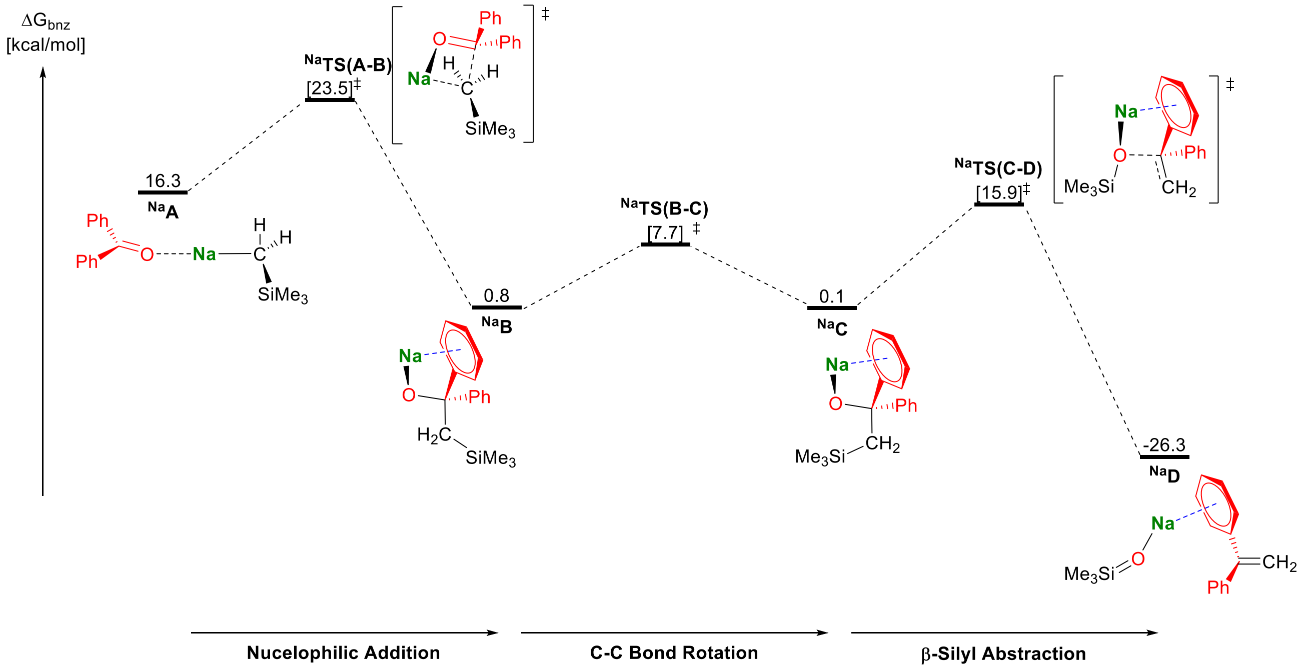

Figure S67. - DFT calculated free energy profile (BP86-D3BJ(C_6H_6)/6-311++G**//BP86/6-31G**&SDDALL, in kcal mol^{-1}) for the reaction of **1**-Na with **2a**

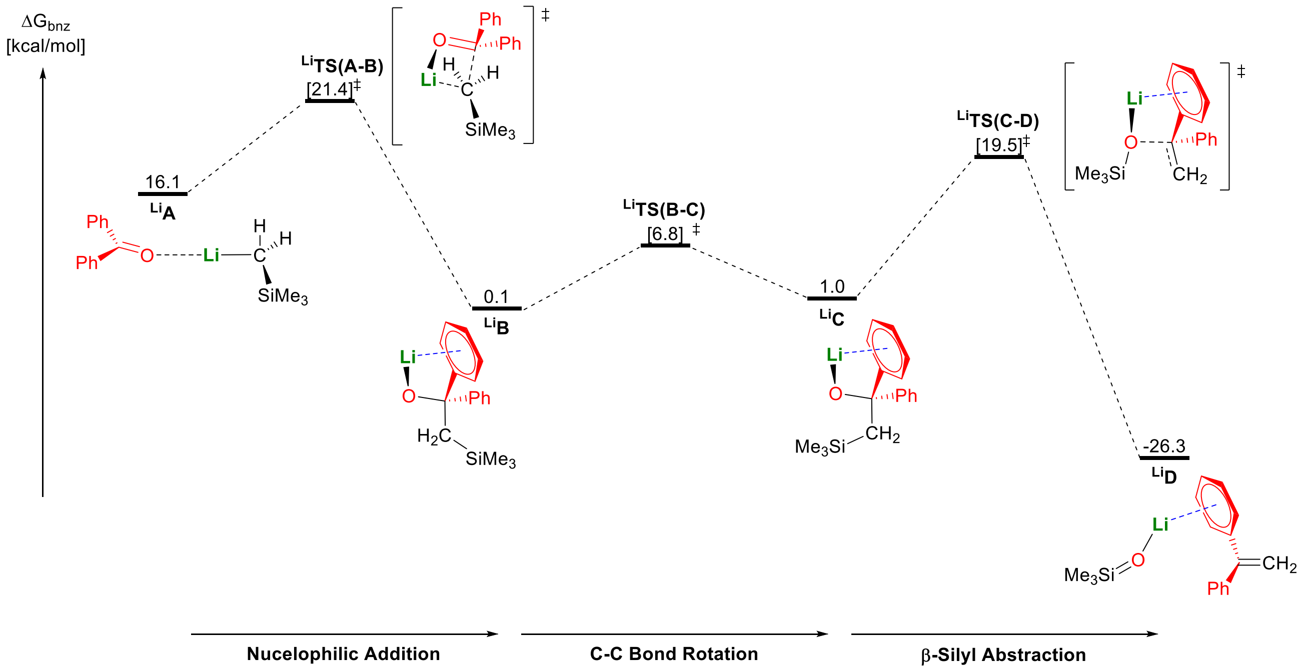

Figure S68. - DFT calculated free energy profile (BP86-D3BJ(C_6H_6)/6-311++G**//BP86/6-31G**, in kcal mol^{-1}) for the reaction of **1**-Li with **2a**.

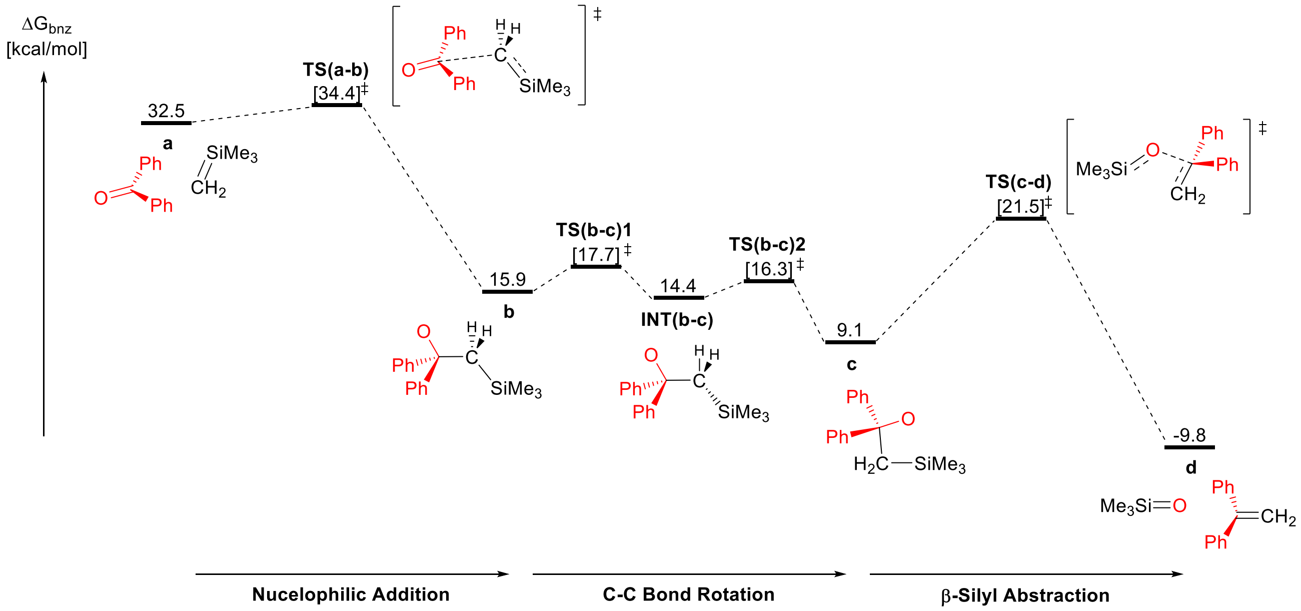

Figure S69. DFT calculated free energy profile (BP86-D3BJ(C_6H_6)/6-311++G**//BP86/6-31G**, in kcal mol^{-1}) for the reaction of **1**-Na with **2a**.

2.1.5 NBO7 Charges

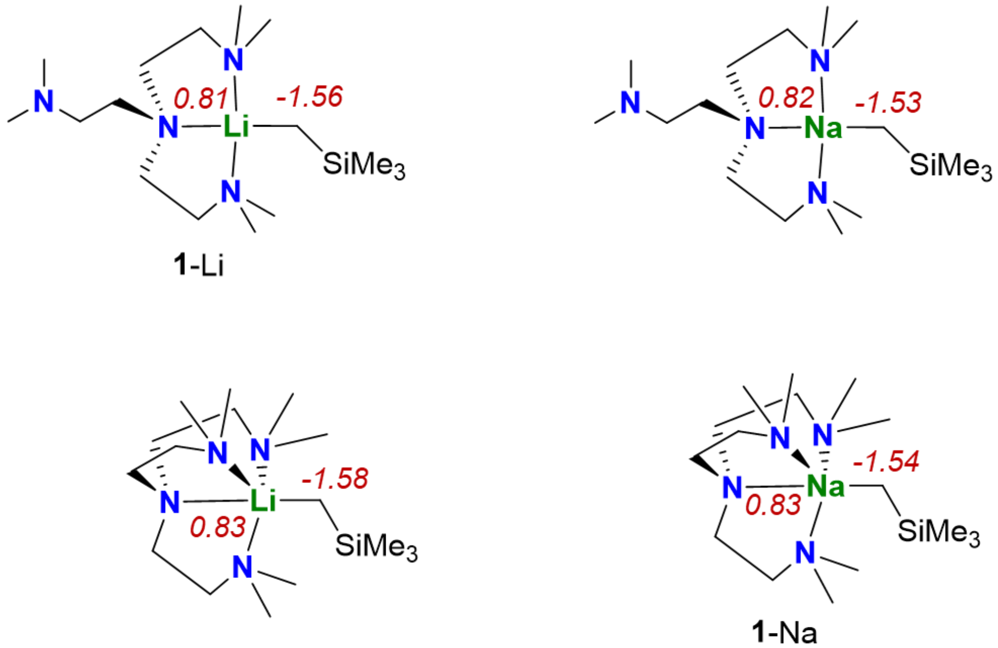

Figure S70. The calculated NBO7 charge distributions of **1-**Li and **1-**Na.

2.1.6 Breakdown of Energy Contributions

The following tables detail the evolution of the relative energies as the successive corrections to the initial SCF energy are included. Terms used are:

$\mathbf{\Delta E_{BSI}}$ SCF energy computed with the BP86 functional with BS1

$\mathbf{\Delta H_{BSI}}$ Enthalpy at 0 K with BS1

$\mathbf{\Delta G_{BSI}}$ Free energy at 298.15 K and 1 atm with BS1

$\mathbf{\Delta G_{BSI/\mathit{bnz}}}$ Free energy corrected for benzene solvent with BS1

$\mathbf{\Delta G_{BSI/\mathit{bnz}+D3BJ}}$ Free energy corrected for benzene and dispersion effects with BS1

$\mathbf{\Delta E_{BS2}}$ SCF energy computed with the BP86 functional with BS2

$\mathbf{\Delta G_{bnz}}$ Free energy corrected for basis set (BS2), dispersion effects and benzene solvent

In each case the final data used in the main article are highlighted in bold.

Table S6. Relative energies for computed structures. Data in bold are those used in the main text. Free energies are quoted relative to complex **1**-Na **or 1′**-Li and **2a** at 0.0 kcal mol^{-1}.

	ΔE_{BSI}	ΔH_{BSI}	ΔG_{BSI}	$\Delta G_{BSI/bnz}$	$\Delta G_{BSI/bnz+D3BJ}$	ΔE_{BS2}	ΔG_{bnz}
**1**-Na (κ^4)	0.0	0.0	0.0	0.0	0.0	0.0	**0.0**
**1**-Na (κ^3)	5.3	5.3	3.4	2.2	7.8	4.1	**6.7**
**A**-Na	−2.5	−2.4	7.3	9.0	4.4	−0.8	**6.1**
**TS(A-B)**-Na	1.4	1.9	13.3	14.7	5.3	2.7	**6.7**
**B**-Na	−27.4	−24.4	−10.7	−8.4	−22.8	−24.4	**−19.7**
**TS(B-C)**-Na	−22.5	−19.8	−5.6	−3.5	−17.6	−19.9	**−15.0**
**TS(B-C′)**-Na	−20.0	−17.6	−4.1	−2.2	−15.0	−17.8	**−12.7**
**C**-Na	−28.1	−25.0	−10.8	−8.7	−24.3	−24.5	**−20.7**
**C′**-Na	−26.4	−23.7	−10.2	−8.4	−23.0	−23.7	**−20.2**
**TS(C-D)**-Na	−4.8	−3.1	10.9	11.7	−4.5	−3.4	**−3.0**
**D**-Na	−45.0	−42.8	−31.6	−30.2	−44.9	−45.2	**−45.1**
**3**-Na	−141.7	−128.2	−76.3	−60.9	−137.8	−126.9	**−123.0**

	ΔE_{BSI}	ΔH_{BSI}	ΔG_{BSI}	$\Delta G_{BSI/bnz}$	$\Delta G_{BSI/bnz+D3BJ}$	ΔE_{BS2}	ΔG_{bnz}
**1′**-Li (κ^4)	0.0	0.0	0.0	0.0	0.0	0.0	**0.0**
**1**-Li (κ^3)	0.7	0.7	−0.6	−1.5	4.1	−0.1	**3.3**
**A**-Li	0.0	0.2	10.8	12.2	5.3	2.0	**7.3**
**TS(A-B)**-Li	2.2	2.6	14.8	16.1	6.2	4.2	**8.1**
**B**-Li	−34.1	−31.3	−19.2	−17.8	−30.0	−31.5	**−27.4**
**TS(B-C)**-Li	−30.0	−27.3	−13.3	−12.2	−25.0	−27.3	**−22.3**
**TS(B-C′)**-Li	−29.1	−26.5	−13.0	−12.0	−24.2	−26.6	**−21.8**
**C**-Li	−30.8	−27.9	−14.6	−13.0	−28.1	−27.8	**−25.1**
**C′**-Li	−33.6	−30.8	−18.8	−17.5	−31.3	−30.8	**−28.4**
**TS(C-D)**-Li	−5.5	−3.8	10.1	11.5	−6.2	−4.1	**−4.8**
**D**-Li	−55.6	−53.6	−45.7	−45.0	−49.4	−56.9	**−50.8**
**3**-Li	−154.4	−141.8	−94.4	−82.5	−158.9	−138.0	**−142.5**

	ΔE_{BSI}	ΔH_{BSI}	ΔG_{BSI}	$\Delta G_{BSI/bnz}$	$\Delta G_{BSI/bnz+D3BJ}$	ΔE_{BS2}	ΔG_{bnz}
**1**-Na	0.0	0.0	0.0	0.0	0.0	0.0	**0.0**
^{Na}**A**	10.9	10.9	7.2	6.4	17.4	9.7	**16.3**
^{Na}**TS(A-B)**	21.0	21.7	23.4	21.0	24.7	19.8	**23.5**
^{Na}**B**	3.4	5.7	7.5	1.7	3.5	0.7	**0.8**
^{Na}**TS(B-C)**	9.2	11.2	13.6	7.7	10.7	6.3	**7.7**
^{Na}**C**	0.3	2.6	4.5	0.2	1.6	−1.2	**0.1**
^{Na}**TS(C-D)**	20.4	21.2	23.3	18.0	17.8	18.5	**15.9**
^{Na}**D**	−26.1	−24.4	−26.2	−29.9	−21.6	−30.8	**−26.3**

	ΔE_{BSI}	ΔH_{BSI}	ΔG_{BSI}	$\Delta G_{BSI/bnz}$	$\Delta G_{BSI/bnz+D3BJ}$	ΔE_{BS2}	ΔG_{bnz}
**1′**-Li	0.0	0.0	0.0	0.0	0.0	0.0	**0.0**
^{Li}**A**	9.5	9.0	4.6	3.5	16.4	9.2	**16.1**
^{Li}**TS(A-B)**	14.9	15.2	15.6	15.2	21.4	14.8	**21.4**
^{Li}**B**	−0.1	2.0	2.8	−3.2	0.7	−0.7	**0.1**
^{Li}**TS(B-C)**	5.5	7.4	8.9	2.8	7.8	4.5	**6.8**
^{Li}**C**	-2.5	−0.4	0.7	−2.8	1.2	−2.7	**1.0**
^{Li}**TS(C-D)**	22.1	22.6	23.9	18.2	20.9	20.7	**19.5**
^{Li}**D**	−33.4	−31.9	−34.0	−35.3	−23.7	−36.0	**−26.3**

	ΔE_{BSI}	ΔH_{BSI}	ΔG_{BSI}	$\Delta G_{BSI/bnz}$	$\Delta G_{BSI/bnz+D3BJ}$	ΔE_{BS2}	ΔG_{bnz}
**1**-Na	0.0	0.0	0.0	0.0	0.0	0.0	**0.0**
**a**	77.0	76.8	77.7	37.6	36.0	73.4	**32.5**
**TS(a-b)**	81.1	80.4	81.7	40.5	39.2	76.4	**34.4**
**b**	64.9	66.6	70.6	27.9	20.1	60.7	**15.9**
**TS(b-c)1**	65.7	67.4	72.6	29.9	22.2	61.2	**17.7**
**INT(b-c)**	64.0	65.7	70.3	27.5	18.9	59.6	**14.4**
**TS(b-c)2**	64.6	66.1	71.7	29.0	20.7	60.2	**16.3**
**c**	50.9	52.8	56.9	16.2	9.5	50.5	**9.1**
**TS(c-d)**	66.4	67.2	71.6	31.0	24.0	63.9	**21.5**
**d**	36.0	37.5	38.6	−3.7	−4.5	40.7	**−9.8**

Data S1. Computed Structures and Energy Data [in Hartrees] (S48-S76).

1-Na system

[(κ^3-Me^{6}Tren)Na]
SCF (BP86) Energy = -694.381170012
Enthalpy 0K = -693.963485
Enthalpy 298K = -693.940103
Free Energy 298K = -694.015651
Lowest Frequency = 15.9143 cm^{-1}
Second Frequency = 31.7048 cm^{-1}
SCF (BP86-D3BJ) Energy = -694.465045009
SCF (Bnz) Energy = -694.425606939
SCF (BS2) Energy = -856.633651314

```
N  -0.19197 -0.18838 -0.26178
N   1.59896  2.35668  0.11639
N   2.28794 -1.97810  0.26949
N  -3.97934 -0.38387  0.40390
C   0.00395  0.91033 -1.24835
H  -0.87604  1.02657 -1.91367
H   0.84268  0.62814 -1.90845
C   0.04439 -1.52617 -0.86924
H  -0.46656 -2.27237 -0.23675
C  -1.51567 -0.12482  0.42405
H  -1.60266  0.87066  0.89398
H  -1.50731 -0.85799  1.25148
C  -2.77393 -0.40914 -0.43092
H  -2.84279  0.29921 -1.29336
H  -2.68737 -1.42182 -0.86345
C  -4.41416  0.98298  0.71296
H  -3.62478  1.53642  1.24870
H  -5.29277  0.94542  1.37713
H  -4.69306  1.56862 -0.19583
C  -5.07712 -1.13704 -0.21642
H  -5.41463 -0.70842 -1.18909
H  -5.94236 -1.14882  0.46661
H  -4.76597 -2.18128 -0.38726
Na  1.69623  0.18198  1.24220
C   0.29615  2.27792 -0.60486
H   0.24198  3.05705 -1.39708
H  -0.49983  2.52111  0.12082
C   1.60492  3.55218  0.99511
H   2.56801  3.61807  1.52791
H   0.79539  3.47806  1.74042
H   1.46482  4.49460  0.42453
C   2.71869  2.47320 -0.84901
H   2.75268  1.59611 -1.51573
H   3.67588  2.52811 -0.30457
H   2.62923  3.38188 -1.48204
C   1.53064 -1.90276 -1.01096
H   1.58525 -2.87234 -1.55567
H   2.04609 -1.16272 -1.65145
C   3.72967 -2.15127 -0.03275
H   3.93121 -3.08236 -0.60385
H   4.30483 -2.19672  0.90672
H   4.09648 -1.29855 -0.62874
C   1.83251 -3.12752  1.08844
H   2.43450 -3.18380  2.01010
H   1.93897 -4.09253  0.54810
H   0.77607 -3.00971  1.37786
H  -0.41590 -1.61407 -1.87603
```

[(κ^4-Me^{6}Tren)Na]
SCF (BP86) Energy = -694.401785611
Enthalpy 0K = -693.983382
Enthalpy 298K = -693.960577
Free Energy 298K = -694.032359
Lowest Frequency = 46.4240 cm^{-1}
Second Frequency = 46.6552 cm^{-1}
SCF (BP86-D3BJ) Energy = -694.492963014
SCF (Bnz) Energy = -694.440929570
SCF (BS2) Energy = -856.651782890

```
Na -0.00012 -0.00008 -1.07974
N   0.00057  0.00033  1.34994
N  -0.56416 -2.39102 -0.52316
N  -1.78917  1.68367 -0.52325
N   2.35307  0.70678 -0.52358
C  -0.54858 -1.30840  1.78297
H  -1.64912 -1.23607  1.78155
H  -0.26332 -1.54322  2.83222
C  -0.11188 -2.48468  0.89123
H   0.99160 -2.54839  0.87861
H  -0.46989 -3.42952  1.35952
C  -0.85825  1.13038  1.78270
H  -0.24535  2.04739  1.78047
H  -1.20378  1.00137  2.83219
C  -2.09565  1.33969  0.89144
H  -2.70246  0.41584  0.87977
H  -2.73478  2.12234  1.35955
C   1.40850  0.17925  1.78300
H   1.89636 -0.80993  1.78222
H   1.46906  0.54427  2.83205
C   2.20857  1.14526  0.89079
H   1.71193  2.13270  0.87825
H   3.20605  1.30773  1.35860
C   0.15916 -3.39785 -1.33497
H  -0.16968 -3.33438 -2.38550
H  -0.02115 -4.43621 -0.98236
H   1.24443 -3.20559 -1.29560
C  -2.01729 -2.65547 -0.62654
H  -2.59200 -1.90587 -0.05913
H  -2.28885 -3.66228 -0.24092
H  -2.32997 -2.59762 -1.68212
C  -3.02306  1.55890 -1.33436
H  -3.39811  0.52255 -1.29403
H  -2.80447  1.81127 -2.38522
H  -3.83258  2.23374 -0.98184
C  -1.29315  3.07478 -0.62816
H  -0.35624  3.19971 -0.06180
H  -2.02989  3.81282 -0.24261
H  -1.08803  3.31592 -1.68416
C   2.86247  1.83708 -1.33530
H   2.97152  1.52105 -2.38603
H   3.85190  2.20030 -0.98301
H   2.15301  2.68050 -1.29540
C   3.30940 -0.41867 -0.62785
H   2.94899 -1.29206 -0.06083
H   4.31720 -0.14977 -0.24288
H   3.41528 -0.71760 -1.68369
```

1-Na (κ^3)
SCF (BP86) Energy = -857.486779716
Enthalpy 0K = -856.939202
Enthalpy 298K = -856.904379
Free Energy 298K = -857.007229

Lowest Frequency = 14.0158 cm^{-1}
Second Frequency = 23.2389 cm^{-1}
SCF (BP86-D3BJ) Energy = -857.604705737
SCF (Bnz) Energy = -857.495605604
SCF (BS2) Energy = -1305.41488268

```
Si -3.52182  1.13230 -0.54516
N   1.68794 -0.38184  0.11585
N  -0.20971 -1.58736  2.25310
N   0.81708 -2.70204 -1.67191
N   2.66879  3.29317 -0.41374
C   2.06928 -0.78442  1.48634
H   2.93097 -0.19698  1.87373
H   2.41745 -1.83097  1.44654
C   2.54359 -1.00470 -0.91493
H   2.43085 -0.41397 -1.84052
C   1.55343  1.08414 -0.06261
H   0.78349  1.44042  0.64319
H   1.14250  1.26425 -1.07253
C   2.84587  1.92273  0.08202
H   3.20499  1.91353  1.14309
H   3.64476  1.45637 -0.52432
C   1.73888  4.07012  0.41221
H   0.73166  3.62359  0.39851
H   1.65224  5.08906 -0.00047
H   2.06963  4.15806  1.47704
C   3.95918  3.97787 -0.50929
H   4.47323  4.09850  0.47617
H   3.81241  4.98360 -0.93859
H   4.63333  3.41594 -1.17866
C  -2.57617 -0.15092 -1.48080
H  -2.09182  0.31939 -2.36562
H  -3.28455 -0.90718 -1.88208
C  -4.62730  0.33718  0.81695
H  -5.36782 -0.34767  0.36603
H  -5.18503  1.09726  1.39277
H  -4.02711 -0.25427  1.53135
C  -4.70861  2.29032 -1.54188
H  -5.22192  3.03105 -0.90049
H  -5.48462  1.69761 -2.05858
H  -4.15342  2.84668 -2.31863
C  -2.32870  2.35325  0.36616
H  -1.72509  1.82313  1.12817
H  -2.86785  3.17074  0.87789
H  -1.62730  2.81519 -0.35347
Na -0.74689 -1.19960 -0.26377
C   0.92378 -0.67210  2.50764
H   1.34886 -0.83352  3.52758
H   0.52495  0.35785  2.49862
C  -1.38156 -1.17272  3.04783
H  -2.22725 -1.84817  2.83896
H  -1.68632 -0.15281  2.76216
H  -1.18178 -1.19210  4.14401
C   0.13868 -2.97864  2.58430
H   0.98340 -3.32754  1.96796
H  -0.72376 -3.63302  2.37497
H   0.41824 -3.10401  3.65654
C   2.19714 -2.47818 -1.20284
H   2.94680 -2.86672 -1.93518
H   2.33201 -3.07586 -0.28177
C   0.50087 -4.13940 -1.65822
H   1.15154 -4.73177 -2.34307
H  -0.54693 -4.28808 -1.96806
H   0.62083 -4.54155 -0.63759
C   0.60157 -2.16369 -3.02770
H  -0.44719 -2.32812 -3.32127
H   1.26927 -2.64229 -3.78178
H   0.77243 -1.07613 -3.04609
H   3.62673 -0.96448 -0.65697
```

1-Na (κ[4])

SCF (BP86) Energy = -857.495290800
Enthalpy 0K = -856.947715
Enthalpy 298K = -856.913319
Free Energy 298K = -857.012691
Lowest Frequency = 23.6776 cm^{-1}
Second Frequency = 30.3470 cm^{-1}
SCF (BP86-D3BJ) Energy = -857.622227464
SCF (Bnz) Energy = -857.502120206
SCF (BS2) Energy = -1305.42135644

```
Si -3.70857 -0.17661  0.23453
Na -0.02776 -0.13320  0.19374
N   2.65579  0.21358 -0.18215
N   0.67647  2.33156  0.81736
N   0.58138 -0.42504 -2.36328
N   1.34381 -1.92662  1.49531
C   2.94735  1.65700 -0.04995
H   2.73242  2.14114 -1.01728
H   4.03101  1.83883  0.14491
C   2.13054  2.35660  1.05092
H   2.31796  1.85953  2.02029
H   2.51594  3.40003  1.16016
C   2.97267 -0.28050 -1.53739
H   3.04780 -1.37969 -1.49092
H   3.97289  0.07663 -1.88196
C   1.93535  0.10663 -2.60596
H   1.85043  1.20748 -2.65677
H   2.33002 -0.21712 -3.60017
C   3.36221 -0.56652  0.85634
H   3.33155  0.01625  1.79254
H   4.44454 -0.69055  0.61125
C   2.76260 -1.96118  1.11734
H   2.84640 -2.57915  0.20430
H   3.39716 -2.46681  1.88641
C  -0.06097  2.64871  2.05281
H  -1.14157  2.54383  1.86819
H   0.14495  3.68080  2.42159
H   0.21192  1.93192  2.84468
C   0.27481  3.26618 -0.24479
H   0.78297  3.02196 -1.19253
H   0.51152  4.32679  0.00879
H  -0.81138  3.18108 -0.41270
C  -0.39709  0.25724 -3.22828
H  -0.37913  1.34196 -3.03001
H  -1.41008 -0.11327 -3.00255
H  -0.19663  0.09621 -4.31326
C   0.51484 -1.87627 -2.60158
H   1.20620 -2.41196 -1.93093
H   0.77050 -2.14694 -3.65317
H  -0.50529 -2.23415 -2.38658
C   0.70519 -3.24292  1.34795
H  -0.37579 -3.13349  1.53412
H   1.11862 -4.00563  2.04905
H   0.84058 -3.61267  0.31706
C   1.13119 -1.43265  2.86433
H   1.59929 -0.44276  2.99646
H   1.55480 -2.11926  3.63523
H   0.04712 -1.31614  3.03075
```

C -2.22782 -0.54728 1.27620
H -2.32441 -1.58801 1.66075
H -2.24551 0.10659 2.17815
C -5.45568 -0.17943 1.06940
H -5.67292 -1.16700 1.51471
H -6.27070 0.05607 0.35930
H -5.49956 0.56224 1.88755
C -3.57245 1.57450 -0.57308
H -3.44127 2.35361 0.20129
H -4.47196 1.84156 -1.15683
H -2.70290 1.62990 -1.25423
C -3.86526 -1.43142 -1.22187
H -2.95301 -1.43619 -1.84697
H -4.72288 -1.20287 -1.87957
H -4.00345 -2.45941 -0.84066

3-Na
SCF (BP86) Energy = -2959.55971342
Enthalpy 0K = -2958.266610
Enthalpy 298K = -2958.170751
Free Energy 298K = -2958.404074
Lowest Frequency = 11.6161 cm^{-1}
Second Frequency = 15.4359 cm^{-1}
SCF (BP86-D3BJ) Energy = -2960.06998991
SCF (Bnz) Energy = -2959.57015978
SCF (BS2) Energy = -4751.13639412

Si -5.72686 -1.38846 -1.86811
Si -3.14269 2.49509 3.75778
Si 3.14190 -2.47689 3.77226
Si 5.72569 1.38042 -1.87649
O -1.65362 -0.44670 -0.91408
O -0.51568 1.65343 1.45116
O 0.51638 -1.64524 1.46020
O 1.65336 0.43997 -0.91741
C -2.72526 -0.54904 -1.81786
C -2.48224 -1.72501 -2.80943
C -2.79903 0.82104 -2.54838
C -0.41340 2.71045 2.35707
C -0.93744 4.05630 1.78053
C 1.11052 2.86824 2.68410
C 0.41397 -2.69892 2.36994
C 0.93950 -4.04642 1.79873
C -1.11016 -2.85616 2.69626
C 2.72470 0.53884 -1.82193
C 2.79904 -0.83442 -2.54641
C 2.48086 1.71019 -2.81876
C -3.83689 4.26606 3.67128
H -3.35406 4.91655 4.42012
H -3.68469 4.72368 2.68105
H -4.92044 4.25456 3.88528
C -3.59213 1.78051 5.47429
H -3.15270 2.38593 6.28612
H -4.68605 1.76671 5.62260
H -3.22285 0.74657 5.58993
C -4.00170 1.41612 2.43548
H -3.66477 1.67658 1.41859
H -3.82808 0.33921 2.61547
H -5.09502 1.56706 2.47611
C -1.22155 2.42987 3.67850
H -0.89630 3.13100 4.47531
H -0.93676 1.41908 4.03482
C 1.22082 -2.41292 3.69105
H 0.89499 -3.11098 4.49032
H 0.93548 -1.40077 4.04300
C 3.83734 -4.24770 3.69293
H 3.35405 -4.89582 4.44353
H 3.68665 -4.70898 2.70418
H 4.92062 -4.23470 3.90819
C 3.58958 -1.75573 5.48649
H 3.14961 -2.35816 6.30024
H 4.68338 -1.74115 5.63565
H 3.22002 -0.72142 5.59790
C 4.00111 -1.40185 2.44689
H 3.66518 -1.66589 1.43059
H 3.82623 -0.32459 2.62347
H 5.09454 -1.55159 2.48876
C 7.05897 1.13623 -0.52673
H 6.81416 1.71383 0.38164
H 7.14661 0.07507 -0.23506
H 8.05005 1.46991 -0.88015
C 5.70500 3.22351 -2.36615
H 6.69500 3.53340 -2.74434
H 4.95954 3.42563 -3.15245
H 5.46279 3.86299 -1.49965
C 4.05095 0.87848 -1.05082
H 4.25476 0.05767 -0.33510
H 3.79600 1.75108 -0.41692
C 6.23069 0.35326 -3.40315
H 7.21562 0.69604 -3.76671
H 6.30906 -0.72268 -3.17785
H 5.50957 0.47180 -4.22806
C -5.70788 -3.23338 -2.35092
H -4.96282 -3.43920 -3.13662
H -5.46605 -3.86971 -1.48201
H -6.69827 -3.54383 -2.72766
C -7.05957 -1.13836 -0.51886
H -6.81510 -1.71287 0.39154
H -7.14637 -0.07608 -0.23103
H -8.05098 -1.47254 -0.87093
C -6.23161 -0.36609 -3.39805
H -7.21688 -0.70934 -3.76025
H -6.30920 0.71059 -3.17604
H -5.51082 -0.48760 -4.22282
C -4.05148 -0.88461 -1.04496
H -4.25455 -0.06072 -0.33258
H -3.79690 -1.75471 -0.40751
C 3.94346 2.92752 3.04915
H 5.02821 2.95657 3.19404
C 3.11979 2.25813 3.96863
H 3.56090 1.76389 4.84140
C 1.72333 2.23021 3.78679
H 1.10448 1.71740 4.52980
C 3.35251 3.57098 1.94683
H 3.97710 4.11043 1.22639
C 1.96054 3.53626 1.76958
H 1.51358 4.05878 0.91710
C -0.63969 5.29262 2.39501
H 0.00590 5.31172 3.28068
C -1.14241 6.49741 1.88134
H -0.89327 7.44399 2.37387
C -1.96039 6.49371 0.73764
H -2.35120 7.43415 0.33450
C -2.27349 5.27196 0.12218
H -2.91265 5.24626 -0.76703
C -1.76447 4.06879 0.64274
H -2.04369 3.11317 0.18662
C -3.68711 3.09892 -2.81170
H -4.45958 3.83688 -2.56661

C -2.64145 3.43883 -3.68272
H -2.58332 4.44208 -4.11751
C -1.66871 2.47046 -3.99306
H -0.83912 2.71473 -4.66497
C -3.75971 1.81042 -2.24944
H -4.58401 1.58168 -1.56875
C -1.75328 1.18600 -3.43362
H -0.99713 0.43767 -3.69433
C   2.00148 2.93264 -2.29569
H   1.82952 3.00586 -1.21512
C   1.78839 4.05100 -3.11526
H   1.42656 4.98754 -2.67607
C   2.04535 3.97146 -4.49570
H   1.87902 4.84017 -5.14167
C   2.52875 2.76837 -5.03285
H   2.74112 2.69262 -6.10527
C   2.74908 1.65379 -4.20214
H   3.13121 0.72708 -4.64145
C   1.75249 -1.20428 -3.42868
H   0.99519 -0.45787 -3.69154
C   1.66864 -2.49116 -3.98260
H   0.83845 -2.73917 -4.65241
C   2.64291 -3.45711 -3.66952
H   2.58534 -4.46225 -4.10000
C   3.68938 -3.11233 -2.80140
H   4.46304 -3.84837 -2.55431
C   3.76126 -1.82139 -2.24465
H   4.58626 -1.58884 -1.56612
C -2.00265 -2.94504 -2.28096
H -1.82999 -3.01316 -1.20016
C -1.79023 -4.06724 -3.09544
H -1.42817 -5.00176 -2.65214
C -2.04810 -3.99413 -4.47607
H -1.88227 -4.86585 -5.11808
C -2.53179 -2.79352 -5.01851
H -2.74488 -2.72278 -6.09112
C -2.75146 -1.67506 -4.19287
H -3.13382 -0.75037 -4.63624
C   1.76796 -4.06208 0.66198
H   2.04734 -3.10766 0.20336
C   2.27838 -5.26661 0.14597
H   2.91858 -5.24340 -0.74257
C   1.96532 -6.48654 0.76505
H   2.35719 -7.42805 0.36545
C   1.14606 -6.48702 1.90784
H   0.89704 -7.43215 2.40323
C   0.64195 -5.28089 2.41699
H -0.00458 -5.29750 3.30201
C -3.35115 -3.56480 1.96161
H -3.97480 -4.10916 1.24405
C -1.95903 -3.53045 1.78533
H -1.51115 -4.05812 0.93651
C -3.94344 -2.91462 3.05921
H -5.02829 -2.94337 3.20336
C -3.12093 -2.23883 3.97509
H -3.56307 -1.73927 4.84429
C -1.72435 -2.21135 3.79430
H -1.10642 -1.69349 4.53458
Na -1.54514 -0.38557 1.36272
Na -0.38115 1.53051 -0.89344
Na 0.38076 -1.53669 -0.88550
Na 1.54550 0.39297 1.35962

A-Na
SCF (BP86) Energy = -1434.12262854
Enthalpy 0K = -1433.388836
Enthalpy 298K = -1433.340761
Free Energy 298K = -1433.476373
Lowest Frequency = 8.5418 cm^{-1}
Second Frequency = 13.4318 cm^{-1}
SCF (BP86-D3BJ) Energy = -1434.30389342
SCF (Bnz) Energy = -1434.12955508
SCF (BS2) Energy = -1882.19202392

Si 1.00809 2.27294 -2.53566
N   2.84259 -1.28386 0.68179
N   0.65467 -0.47279 2.65617
N   0.79079 -2.83356 -1.00011
N   6.05348 0.72636 0.09444
C   2.80286 -1.66365 2.10914
H   3.82008 -1.83271 2.53010
H   2.28463 -2.63516 2.18423
C   3.16320 -2.43554 -0.18544
H   3.47467 -2.03527 -1.16578
C   3.71415 -0.11722 0.40655
H   3.28836 0.75485 0.93255
H   3.63960 0.10991 -0.67161
C   5.21370 -0.27747 0.75953
H   5.35825 -0.25821 1.87055
H   5.55862 -1.27047 0.41534
C   5.77058 2.08631 0.56114
H   4.73528 2.37510 0.31937
H   6.43830 2.79458 0.04281
H   5.92419 2.21231 1.66226
C   7.47130 0.41001 0.27225
H   7.80074 0.42714 1.34071
H   8.08523 1.14007 -0.28238
H   7.68474 -0.59403 -0.13347
C -0.25916 1.13142 -1.82322
H -0.62466 0.38596 -2.55795
H -1.14127 1.66785 -1.42558
C   1.84406 3.33748 -1.16652
H   1.09421 3.93897 -0.62145
H   2.59190 4.03585 -1.58278
H   2.35207 2.69369 -0.42591
C   0.36787 3.55008 -3.83726
H   1.17495 4.20929 -4.20766
H -0.41958 4.19175 -3.40356
H -0.07294 3.03227 -4.70736
C   2.40089 1.29703 -3.43532
H   2.93094 0.61161 -2.74944
H   3.15590 1.97267 -3.87470
H   1.98295 0.68801 -4.25702
Na 0.28806 -0.47565 0.05690
C   2.07784 -0.64225 3.00039
H   2.20948 -0.95169 4.06546
H   2.56752 0.34297 2.90481
C   0.11975 0.78258 3.20946
H -0.92224 0.90420 2.87367
H   0.70701 1.63602 2.83060
H   0.14393 0.80735 4.32410
C -0.15714 -1.60203 3.13781
H   0.20983 -2.55149 2.71333
H -1.19704 -1.46062 2.80434
H -0.13982 -1.69273 4.24982
C   1.99496 -3.41811 -0.38226
H   2.37961 -4.28038 -0.98136
H   1.69259 -3.83792 0.59510
C -0.32675 -3.78672 -0.91196

H -0.12683 -4.73568 -1.46349
H -1.23823 -3.32903 -1.32938
H -0.52079 -4.03914 0.14481
C   1.02188 -2.47942 -2.41256
H   0.09562 -2.06354 -2.83864
H   1.33245 -3.36171 -3.02058
H   1.79283 -1.69862 -2.49972
H   4.02782 -3.03023 0.19227
O -2.01348 -0.33354 0.70756
C -3.20662 0.00182 0.48167
C -4.11348 -0.93421 -0.24584
C -5.17575 -0.48788 -1.06859
C -3.85714 -2.32446 -0.16521
C -5.95918 -1.40694 -1.78058
H -5.36206 0.58500 -1.17449
C -4.65180 -3.24085 -0.86287
H -3.02980 -2.66149 0.46629
C -5.70653 -2.78539 -1.67525
H -6.76589 -1.04530 -2.42688
H -4.45315 -4.31464 -0.77517
H -6.32519 -3.50149 -2.22622
C -3.69948 1.33242 0.94106
C -5.04639 1.56324 1.31343
C -2.76395 2.38726 1.07819
C -5.44344 2.81517 1.80373
H -5.77447 0.74908 1.24440
C -3.17013 3.64181 1.54539
H -1.72808 2.20271 0.77660
C -4.51042 3.86011 1.91494
H -6.48468 2.97510 2.10348
H -2.44169 4.45626 1.62106
H -4.82482 4.83953 2.29067

TS(A-B)-Na
SCF (BP86) Energy = -1434.11645476
Enthalpy 0K = -1433.382068
Enthalpy 298K = -1433.335054
Free Energy 298K = -1433.466662
Lowest Frequency = -188.5766 cm^{-1}
Second Frequency = 11.8375 cm^{-1}
SCF (BP86-D3BJ) Energy = -
1434.30521430
SCF (Bnz) Energy = -1434.12412773
SCF (BS2) Energy = -1882.18636691

Si -0.57659 2.64112 -1.55067
N   3.04713 -0.95615 0.18779
N   1.61019 -1.53430 2.80897
N   1.05640 -2.86702 -1.19472
N   4.78511 2.32859 -0.79721
C   3.69964 -1.51321 1.39111
H   4.77531 -1.23534 1.45906
H   3.67979 -2.61389 1.30721
C   3.32806 -1.74268 -1.03136
H   3.09417 -1.10111 -1.89859
C   3.28183 0.49546 -0.00749
H   2.97485 1.00875 0.92004
H   2.58166 0.84854 -0.78688
C   4.71734 0.91618 -0.40051
H   5.44006 0.68528 0.42388
H   5.03682 0.31823 -1.27363
C   4.57914 3.23841 0.33245
H   3.57670 3.09983 0.76732
H   4.64046 4.28021 -0.02335
H   5.33676 3.10638 1.14494
C   6.05561 2.62249 -1.46248
H   6.94721 2.47448 -0.80443
H   6.05867 3.67126 -1.80490
H   6.17327 1.97452 -2.34789
C -0.67595 1.05174 -0.61882
H -0.80691 0.08295 -1.11162
H -0.66928 1.06871 0.47221
C -0.30719 4.07513 -0.31007
H -1.10842 4.08739 0.44871
H -0.30558 5.05536 -0.81786
H   0.65627 3.96968 0.21872
C -2.16791 3.04630 -2.55426
H -2.07036 4.00146 -3.10148
H -3.04141 3.12604 -1.88425
H -2.38294 2.25924 -3.29895
C   0.85249 2.69243 -2.83957
H   1.84045 2.63789 -2.34888
H   0.82341 3.62072 -3.43939
H   0.77853 1.84324 -3.54320
Na 0.49427 -1.09412 0.55900
C   3.01650 -1.09648 2.70730
H   3.62972 -1.48036 3.55750
H   3.02836 0.00446 2.79180
C   0.87885 -0.76228 3.83033
H -0.18821 -1.03484 3.79481
H   0.96000 0.31474 3.60790
H   1.26556 -0.93742 4.86063
C   1.50893 -2.97083 3.11166
H   2.00696 -3.56934 2.33077
H   0.44710 -3.26537 3.14073
H   1.97153 -3.23183 4.09239
C   2.52016 -3.04971 -1.13154
H   2.89416 -3.61491 -2.01986
H   2.73517 -3.68558 -0.25232
C   0.37880 -4.16218 -1.00336
H   0.64635 -4.90509 -1.79015
H -0.71241 -4.00939 -1.02693
H   0.65152 -4.58696 -0.02200
C   0.63584 -2.28191 -2.48351
H -0.46267 -2.19928 -2.50257
H   0.95907 -2.90333 -3.35082
H   1.04530 -1.26627 -2.60268
H   4.40487 -2.01167 -1.12821
O -1.64796 -1.00167 1.35173
C -2.57163 -0.41271 0.69895
C -3.14696 -1.12215 -0.50032
C -3.62477 -0.47064 -1.66112
C -3.19921 -2.53572 -0.45643
C -4.14613 -1.20785 -2.73288
H -3.53017 0.61678 -1.73250
C -3.72403 -3.27376 -1.52995
H -2.83453 -3.03818 0.44537
C -4.20127 -2.61343 -2.67365
H -4.49846 -0.68371 -3.62811
H -3.76962 -4.36730 -1.46840
H -4.60790 -3.18603 -3.51415
C -3.29242 0.74297 1.31766
C -4.56937 1.18872 0.89458
C -2.72439 1.35239 2.46513
C -5.21922 2.23936 1.55755
H -5.06410 0.69900 0.05149
C -3.37208 2.40242 3.12172
H -1.75882 0.97869 2.81812
C -4.62364 2.86034 2.66771
H -6.20502 2.56766 1.20963

```
H  -2.90524  2.86628  3.99824
H  -5.13300  3.68182  3.18250
```

B-Na
SCF (BP86) Energy = -1434.16233047
Enthalpy 0K = -1433.423932
Enthalpy 298K = -1433.377978
Free Energy 298K = -1433.504919
Lowest Frequency = 9.2186 cm^{-1}
Second Frequency = 18.9903 cm^{-1}
SCF (BP86-D3BJ) Energy = -1434.35913048
SCF (Bnz) Energy = -1434.16854504
SCF (BS2) Energy = -1882.22953791

```
Si -3.70760  1.56796 -1.29707
N   3.07192  0.73047  0.34414
N   2.24089 -1.62129  2.30978
N   3.44815 -1.52803 -1.63842
N   1.98504  4.38211 -0.08238
C   3.46908  0.52267  1.75562
H   3.73669  1.48003  2.25221
H   4.39266 -0.08311  1.76238
C   4.20465  0.65734 -0.59416
H   3.93792  1.23210 -1.49809
C   2.18303  1.89910  0.13140
H   1.23592  1.69453  0.66413
H   1.91518  1.91758 -0.94154
C   2.78194  3.28183  0.47811
H   2.90465  3.39495  1.58539
H   3.79621  3.35539  0.04007
C   0.65955  4.47340  0.53893
H   0.08389  3.55024  0.36762
H   0.10071  5.30767  0.08218
H   0.70741  4.65444  1.64149
C   2.69643  5.65386  0.05135
H   2.88533  5.94849  1.11349
H   2.10775  6.45872 -0.42160
H   3.67117  5.59517 -0.46355
C  -2.00096  1.14957 -0.50716
H  -1.21005  1.46716 -1.21556
H  -1.90863  1.85556  0.34194
C  -5.02718  1.89126  0.04166
H  -5.22121  0.98904  0.64371
H  -5.97896  2.21285 -0.41675
H  -4.69947  2.68972  0.72972
C  -4.37568  0.27182 -2.53187
H  -5.31197  0.63987 -2.98773
H  -4.59833 -0.68813 -2.03859
H  -3.66072  0.06986 -3.34670
C  -3.44621  3.19904 -2.26810
H  -3.06374  3.99842 -1.60992
H  -4.39199  3.55605 -2.71226
H  -2.71958  3.06870 -3.08924
Na  1.44994 -1.15290 -0.07709
C   2.38892 -0.17527  2.60300
H   2.62404 -0.01528  3.68316
H   1.40697  0.29115  2.40256
C   0.97422 -2.10854  2.89820
H   0.85608 -3.18318  2.67399
H   0.14252 -1.54406  2.43791
H   0.95060 -1.98523  4.00570
C   3.36842 -2.40138  2.83810
H   4.32138 -2.07784  2.38672
H   3.22553 -3.46759  2.59398
H   3.46511 -2.31087  3.94588
C   4.55431 -0.79217 -0.98619
H   5.46424 -0.77623 -1.63403
H   4.82658 -1.35601 -0.07459
C   3.77955 -2.96084 -1.73407
H   4.68276 -3.15289 -2.35858
H   2.92999 -3.50481 -2.17988
H   3.96472 -3.36987 -0.72640
C   3.16238 -1.00242 -2.98584
H   2.32111 -1.56419 -3.42321
H   4.04085 -1.08531 -3.66733
H   2.86371  0.05638 -2.93324
H   5.13252  1.13024 -0.19893
O  -0.31491 -0.08492  0.72260
C  -1.55104 -0.23089  0.10971
C  -1.32463 -1.33177 -0.96584
C  -1.05818 -1.04761 -2.32460
C  -1.14162 -2.67495 -0.54159
C  -0.62246 -2.04750 -3.21553
H  -1.19450 -0.02903 -2.69984
C  -0.70938 -3.67585 -1.42446
H  -1.35656 -2.92736  0.50248
C  -0.43887 -3.36743 -2.77293
H  -0.44238 -1.79199 -4.26668
H  -0.59737 -4.70559 -1.06458
H  -0.11567 -4.14921 -3.46879
C  -2.64307 -0.65471  1.13369
C  -3.75631 -1.46156  0.82101
C  -2.52227 -0.17184  2.45187
C  -4.71997 -1.77538  1.79641
H  -3.86590 -1.86374 -0.19235
C  -3.48786 -0.46770  3.42523
H  -1.63664  0.42908  2.68407
C  -4.59262 -1.27516  3.10218
H  -5.57044 -2.41531  1.53401
H  -3.37710 -0.07382  4.44252
H  -5.34359 -1.51724  3.86232
```

TS(B-C)-Na
SCF (BP86) Energy = -1434.15438267
Enthalpy 0K = -1433.416594
Enthalpy 298K = -1433.371215
Free Energy 298K = -1433.496882
Lowest Frequency = -74.7007 cm^{-1}
Second Frequency = 9.4256 cm^{-1}
SCF (BP86-D3BJ) Energy = -1434.35077822
SCF (Bnz) Energy = -1434.16069117
SCF (BS2) Energy = -1882.22241024

```
Si -3.72937  1.57943 -1.20016
N   2.95214  0.98956  0.38646
N   2.18431 -1.36393  2.36859
N   3.70066 -1.30731 -1.44739
N   1.55242  4.51414 -0.21593
C   3.25712  0.86443  1.83009
H   3.39853  1.85711  2.30869
H   4.22733  0.34619  1.92958
C   4.15435  0.98683 -0.46407
H   3.90038  1.48797 -1.41444
C   1.98754  2.06929  0.06492
H   1.03138  1.81476  0.55659
H   1.77701  2.01409 -1.01909
C   2.45186  3.51169  0.37202
H   2.56639  3.66805  1.47477
```

H   3.45354 3.66807 -0.07246
C   0.24049 4.52785 0.43902
H -0.25531 3.54942 0.34073
H -0.40220 5.28090 -0.04741
H   0.30041 4.77862 1.52735
C   2.15685 5.84597 -0.16543
H   2.34774 6.20888 0.87497
H   1.48962 6.57380 -0.65840
H   3.11886 5.84296 -0.70675
C -2.03070 0.67795 -1.19225
H -1.90537 0.39406 -2.25475
H -1.28990 1.48163 -1.02300
C -4.27972 2.28175 0.48400
H -4.55497 1.48232 1.18949
H -5.15374 2.94356 0.34925
H -3.47573 2.87782 0.94888
C -5.14623 0.53917 -1.95038
H -6.01625 1.18500 -2.16426
H -5.47641 -0.25715 -1.26525
H -4.83895 0.06850 -2.90059
C -3.45380 3.06472 -2.38206
H -2.66160 3.73578 -2.00682
H -4.37577 3.66273 -2.49022
H -3.15460 2.72925 -3.39062
Na 1.54116 -1.06479 -0.08704
C   2.18043 0.09692 2.61904
H   2.31262 0.31067 3.70731
H   1.18070 0.46718 2.32754
C   0.92071 -1.94656 2.86903
H   0.91746 -3.03351 2.67767
H   0.08428 -1.47857 2.31887
H   0.78935 -1.78916 3.96456
C   3.32791 -2.02286 3.01434
H   4.28290 -1.62621 2.63070
H   3.30096 -3.10396 2.79736
H   3.32263 -1.89254 4.12230
C   4.67315 -0.43599 -0.75132
H   5.62745 -0.35753 -1.32660
H   4.92303 -0.92832 0.20676
C   4.16906 -2.70478 -1.43231
H   5.13315 -2.84135 -1.97498
H   3.41073 -3.35110 -1.90519
H   4.30967 -3.04001 -0.39088
C   3.48685 -0.87936 -2.84148
H   2.74822 -1.54595 -3.31524
H   4.42612 -0.90602 -3.44154
H   3.08309 0.14486 -2.87579
H   4.99916 1.57001 -0.03132
O -0.38795 -0.11662 0.45180
C -1.54022 -0.49083 -0.22033
C -1.11011 -1.77572 -1.01041
C -0.55460 -1.73364 -2.31226
C -1.01685 -3.01372 -0.32125
C   0.05668 -2.86037 -2.89516
H -0.59884 -0.80629 -2.89302
C -0.40709 -4.13928 -0.89496
H -1.44300 -3.08651 0.68443
C   0.14102 -4.07242 -2.19027
H   0.44898 -2.79189 -3.91729
H -0.37084 -5.08012 -0.33302
H   0.60072 -4.95477 -2.64841
C -2.68559 -0.84621 0.77072
C -3.80221 -1.63138 0.41458
C -2.60875 -0.35681 2.08723
C -4.81365 -1.91498 1.34806
H -3.87181 -2.04103 -0.59996
C -3.62127 -0.62665 3.02238
H -1.72546 0.23839 2.34074
C -4.72942 -1.40962 2.65695
H -5.66660 -2.53803 1.05476
H -3.54520 -0.22808 4.04111
H -5.51733 -1.63048 3.38554

C-Na
SCF (BP86) Energy = -1434.16329700
Enthalpy 0K = -1433.424954
Enthalpy 298K = -1433.379015
Free Energy 298K = -1433.505157
Lowest Frequency = 12.6620 cm^{-1}
Second Frequency = 21.2211 cm^{-1}
SCF (BP86-D3BJ) Energy = -1434.36203282
SCF (Bnz) Energy = -1434.16968084
SCF (BS2) Energy = -1882.22976978

Si -2.37791 2.60741 -0.76600
N   2.89996 0.40621 0.38681
N   1.65561 -1.47052 2.61632
N   3.09818 -2.27982 -1.06576
N   2.44659 4.11130 -0.52097
C   3.15030 0.42286 1.84757
H   3.46049 1.43163 2.19449
H   4.00579 -0.24133 2.06274
C   4.06010 -0.02827 -0.41262
H   3.91949 0.34726 -1.44162
C   2.27725 1.65692 -0.10712
H   1.38226 1.84480 0.50916
H   1.89221 1.47443 -1.12666
C   3.19605 2.90112 -0.15568
H   3.74349 3.03302 0.81124
H   3.97088 2.73976 -0.92751
C   1.70132 4.65615 0.61776
H   0.99898 3.90819 1.01723
H   1.10475 5.52198 0.28592
H   2.36889 4.99159 1.44974
C   3.32436 5.13626 -1.08943
H   4.10315 5.50101 -0.37545
H   2.72069 6.00552 -1.40127
H   3.83695 4.73961 -1.98247
C -1.91526 0.85070 -1.40351
H -2.63246 0.61954 -2.21825
H -0.92088 0.95143 -1.88162
C -1.89615 2.91152 1.05509
H -2.71547 2.61750 1.73112
H -1.65512 3.97253 1.24088
H -1.03210 2.27052 1.29533
C -4.24710 2.92561 -0.99526
H -4.51924 3.94033 -0.65499
H -4.84248 2.19935 -0.41727
H -4.54281 2.83672 -2.05533
C -1.42172 3.84757 -1.86824
H -0.33057 3.76157 -1.71672
H -1.71273 4.88827 -1.63924
H -1.62777 3.67382 -2.93938
Na 1.06153 -1.33135 0.12669
C   1.92966 -0.01629 2.67891
H   2.07796 0.31326 3.73590
H   1.02666 0.49764 2.30114
C   0.28828 -1.73571 3.11603
H   0.09504 -2.82210 3.08633

H -0.43166 -1.22239 2.44959
H   0.15042 -1.39206 4.16729
C   2.63263 -2.23797 3.40097
H   3.65713 -2.07917 3.02422
H   2.40725 -3.31452 3.31939
H   2.61858 -1.96284 4.48196
C   4.23085 -1.55881 -0.44595
H   5.19165 -1.79694 -0.96347
H   4.33019 -1.93303 0.58963
C   3.19925 -3.72445 -0.78883
H   4.12035 -4.18054 -1.22022
H   2.32307 -4.24020 -1.21587
H   3.20715 -3.89558 0.30085
C   3.04807 -2.06675 -2.52374
H   2.17723 -2.60039 -2.93712
H   3.96939 -2.43141 -3.03483
H   2.92390 -0.99719 -2.75604
H   5.02058 0.41009 -0.05795
O -0.76813 -0.14354 0.51118
C -1.81247 -0.33248 -0.37487
C -1.52145 -1.68725 -1.11047
C -1.06963 -1.78798 -2.44442
C -1.55603 -2.88231 -0.34556
C -0.66013 -3.02182 -2.99034
H -1.05298 -0.89451 -3.07671
C -1.15113 -4.11207 -0.88245
H -1.92759 -2.82956 0.68400
C -0.69156 -4.19012 -2.21365
H -0.33472 -3.06804 -4.03688
H -1.21189 -5.01990 -0.27038
H -0.39106 -5.15244 -2.64239
C -3.19653 -0.47329 0.32989
C -4.36726 -0.83227 -0.37208
C -3.29369 -0.23177 1.71093
C -5.60098 -0.93638 0.28889
H -4.31013 -1.04446 -1.44670
C -4.52672 -0.33156 2.37862
H -2.37109 0.04427 2.23128
C -5.68624 -0.68399 1.67001
H -6.49871 -1.21934 -0.27297
H -4.58244 -0.13556 3.45617
H -6.64874 -0.76602 2.18712

TS(B-C')-Na
SCF (BP86) Energy = -1434.15051444
Enthalpy 0K = -1433.413075
Enthalpy 298K = -1433.367515
Free Energy 298K = -1433.494519
Lowest Frequency = -58.8817 cm^{-1}
Second Frequency = 7.1188 cm^{-1}
SCF (BP86-D3BJ) Energy = -1434.34469997
SCF (Bnz) Energy = -1434.15723670
SCF (BS2) Energy = -1882.21905346

Si -2.55476 1.49907 -2.50916
N   3.00458 0.35998 0.32800
N   1.89020 -1.92471 2.22000
N   2.98613 -1.85977 -1.74229
N   2.40364 4.14825 0.16029
C   3.41986 0.02700 1.70956
H   3.85005 0.90714 2.23399
H   4.23810 -0.71252 1.65055
C   4.07537 0.16511 -0.66339
H   3.85165 0.80216 -1.53685
C   2.29146 1.65497 0.20328
H   1.34724 1.56933 0.77125
H   1.98077 1.75972 -0.85254
C   3.09852 2.92037 0.57247
H   3.33600 2.93792 1.66658
H   4.06942 2.89729 0.04089
C   1.18288 4.37862 0.94056
H   0.47359 3.54574 0.81378
H   0.68972 5.29798 0.58220
H   1.38295 4.49997 2.03417
C   3.29370 5.30542 0.26056
H   3.64135 5.51053 1.30353
H   2.77082 6.20686 -0.10282
H   4.18566 5.14967 -0.37092
C -2.13960 1.33326 -0.64062
H -1.30253 2.03309 -0.45415
H -3.02001 1.83962 -0.19787
C -3.25073 3.27634 -2.67467
H -4.16137 3.40724 -2.06499
H -3.51116 3.50853 -3.72234
H -2.51490 4.02734 -2.33817
C -3.90500 0.29344 -3.11348
H -4.19808 0.52813 -4.15188
H -4.80827 0.38040 -2.48492
H -3.57024 -0.75532 -3.07721
C -1.04703 1.44476 -3.69894
H -0.21825 2.05640 -3.30128
H -1.33691 1.87526 -4.67406
H -0.65931 0.43138 -3.89571
Na 1.10057 -1.25173 -0.09735
C   2.27617 -0.53757 2.57232
H   2.56940 -0.46985 3.64771
H   1.37543 0.08730 2.43258
C   0.57530 -2.23471 2.82184
H   0.27648 -3.25993 2.54395
H -0.16532 -1.52146 2.41815
H   0.59466 -2.16841 3.93408
C   2.89093 -2.90023 2.67347
H   3.87190 -2.70726 2.20754
H   2.57369 -3.91590 2.38299
H   3.02760 -2.88427 3.78052
C   4.20538 -1.30363 -1.11261
H   5.08442 -1.38996 -1.79644
H   4.43003 -1.93173 -0.23075
C   3.12080 -3.31980 -1.89400
H   3.96720 -3.60707 -2.55988
H   2.19163 -3.73423 -2.31974
H   3.28600 -3.78403 -0.90726
C   2.73223 -1.25204 -3.06075
H   1.82626 -1.70019 -3.50063
H   3.57795 -1.40782 -3.77018
H   2.55631 -0.16931 -2.96330
H   5.07617 0.49231 -0.29932
O -0.44702 0.11729 0.68502
C -1.77079 0.07445 0.29333
C -1.92073 -1.32380 -0.37987
C -1.32397 -1.55549 -1.64396
C -2.40577 -2.45892 0.31121
C -1.21893 -2.84389 -2.19438
H -0.93568 -0.69989 -2.20591
C -2.29781 -3.75237 -0.22937
H -2.87546 -2.32515 1.29026
C -1.70261 -3.95886 -1.48428
H -0.77834 -2.97578 -3.19055
H -2.68897 -4.60623 0.33640

H -1.63169 -4.96524 -1.91060
C -2.74764 0.20131 1.50436
C -4.13942 -0.00816 1.38813
C -2.23757 0.60423 2.74937
C -4.98970 0.16232 2.49155
H -4.56027 -0.31531 0.42250
C -3.08460 0.78884 3.85542
H -1.15753 0.77448 2.80697
C -4.46461 0.56302 3.73326
H -6.06589 -0.01652 2.38337
H -2.66617 1.11117 4.81658
H -5.12800 0.69996 4.59447

C'-Na
SCF (BP86) Energy = -1434.16066556
Enthalpy 0K = -1433.422899
Enthalpy 298K = -1433.376661
Free Energy 298K = -1433.504147
Lowest Frequency = 12.1455 cm^{-1}
Second Frequency = 19.2545 cm^{-1}
SCF (BP86-D3BJ) Energy = -1434.35776044
SCF (Bnz) Energy = -1434.16758849
SCF (BS2) Energy = -1882.22845863

Si -1.24888 2.97643 -1.76877
N   2.80266 -0.78747 0.25654
N   0.92436 -2.33986 2.21166
N   1.47626 -2.55962 -1.86116
N   4.35969 2.71675 0.34390
C   3.20918 -1.51686 1.47844
H   4.13367 -1.09495 1.92875
H   3.46567 -2.55084 1.19102
C   3.47885 -1.28561 -0.95913
H   3.37794 -0.50743 -1.73569
C   2.88562 0.68874 0.39326
H   2.21432 0.98758 1.21635
H   2.45720 1.13654 -0.52069
C   4.30414 1.27282 0.60090
H   4.68144 1.02853 1.62754
H   4.99841 0.78873 -0.11149
C   3.55503 3.48369 1.29992
H   2.48673 3.23589 1.19815
H   3.66227 4.55935 1.08242
H   3.86132 3.31615 2.36271
C   5.74689 3.18337 0.35192
H   6.25652 3.04731 1.33794
H   5.77695 4.25743 0.10138
H   6.33036 2.63814 -0.41021
C -2.64862 2.00901 -0.88832
H -3.05491 2.73838 -0.16117
H -3.49370 1.80023 -1.57130
C -0.10188 3.78175 -0.47732
H -0.65284 4.50304 0.15117
H   0.74570 4.31190 -0.94540
H   0.26969 2.96883 0.16689
C -2.10241 4.37978 -2.76318
H -1.35742 5.02993 -3.25533
H -2.71867 5.01817 -2.10609
H -2.76442 3.97725 -3.55017
C -0.20452 1.96747 -3.01107
H   0.23961 1.11506 -2.47441
H   0.60109 2.59070 -3.43860
H -0.80825 1.57923 -3.84963
Na 0.29578 -1.22225 -0.02682
C   2.12293 -1.54824 2.56854
H   2.58371 -1.92816 3.51240
H   1.78681 -0.51720 2.77847
C -0.16659 -2.05280 3.16486
H -1.04956 -2.65930 2.90461
H -0.45906 -0.99214 3.10286
H   0.11699 -2.28990 4.21611
C   1.21338 -3.78331 2.22677
H   1.99736 -4.03525 1.49421
H   0.30398 -4.34230 1.95162
H   1.55047 -4.13829 3.22885
C   2.90292 -2.61424 -1.48404
H   3.53604 -2.94893 -2.34147
H   3.00695 -3.39246 -0.70523
C   0.94689 -3.92306 -2.04667
H   1.45929 -4.47189 -2.87070
H -0.12976 -3.86865 -2.27766
H   1.07305 -4.50317 -1.11661
C   1.26985 -1.78639 -3.10084
H   0.19369 -1.76068 -3.33623
H   1.81648 -2.22786 -3.96654
H   1.60306 -0.74449 -2.97335
H   4.57375 -1.43432 -0.81532
O -0.78082 0.68458 0.01764
C -2.16009 0.72004 -0.10437
C -2.55709 -0.59375 -0.84545
C -2.63386 -0.67514 -2.25504
C -2.66570 -1.81114 -0.12526
C -2.80548 -1.90420 -2.91424
H -2.56300 0.24088 -2.84885
C -2.83534 -3.04391 -0.77755
H -2.65568 -1.77517 0.97059
C -2.90415 -3.10064 -2.18173
H -2.87963 -1.92539 -4.00816
H -2.94244 -3.96143 -0.18607
H -3.05987 -4.05647 -2.69392
C -2.87419 0.77492 1.28176
C -4.25456 0.53403 1.44806
C -2.12257 1.15162 2.40945
C -4.85965 0.65033 2.70984
H -4.86011 0.24562 0.58030
C -2.72455 1.28227 3.67268
H -1.05688 1.34685 2.24265
C -4.09659 1.02552 3.82977
H -5.93170 0.44903 2.82051
H -2.12334 1.58858 4.53768
H -4.56985 1.12042 4.81346

TS(C-D)-Na
SCF (BP86) Energy = -1434.12627948
Enthalpy 0K = -1433.389944
Enthalpy 298K = -1433.344198
Free Energy 298K = -1433.470580
Lowest Frequency = -393.7520 cm^{-1}
Second Frequency = 10.6932 cm^{-1}
SCF (BP86-D3BJ) Energy = -1434.32595759
SCF (Bnz) Energy = -1434.13477615
SCF (BS2) Energy = -1882.19606872

Si 0.98187 -1.28563 -2.33988
N -3.09708 0.46108 0.92717
N -1.17765 -1.66497 2.14824
N -0.83276 2.50152 1.08315
N -6.09443 -0.16445 -1.38191

```
C  -3.31127 -0.29905  2.18005
H  -4.39221 -0.42644  2.40955
H  -2.90023  0.29949  3.01095
C  -3.29748  1.91345  1.13171
H  -3.46960  2.36851  0.14089
C  -3.88797 -0.05700 -0.21691
H  -3.54862 -1.08622 -0.42343
H  -3.61552  0.53780 -1.10756
C  -5.42921 -0.01022 -0.08180
H  -5.78407 -0.77730  0.65297
H  -5.72628  0.97533  0.32261
C  -5.88436 -1.49597 -1.95904
H  -4.81446 -1.67365 -2.15244
H  -6.40964 -1.55961 -2.92649
H  -6.26474 -2.32075 -1.30658
C  -7.52607  0.12063 -1.26568
H  -8.06583 -0.58343 -0.58586
H  -7.99694  0.05676 -2.26136
H  -7.67408  1.14512 -0.88293
C   3.39089 -0.62206 -2.31894
H   3.94241 -1.56020 -2.42810
H   3.58282  0.12738 -3.09217
C  -0.95615 -1.54160 -2.04399
H  -1.19926 -2.07639 -1.10386
H  -1.36959 -2.15759 -2.86490
H  -1.52465 -0.58956 -2.06741
C   1.56273 -3.11194 -2.47010
H   0.71089 -3.80943 -2.57360
H   2.12690 -3.39387 -1.56482
H   2.23570 -3.25194 -3.33197
C   0.84879 -0.42177 -4.05828
H   0.73615  0.67120 -3.93772
H  -0.04241 -0.77855 -4.60860
H   1.74235 -0.59657 -4.67695
Na -0.54204  0.08401  0.33794
C  -2.65617 -1.68923  2.18853
H  -3.02226 -2.24001  3.08810
H  -3.00240 -2.26466  1.31188
C  -0.66519 -3.01143  1.82059
H   0.43412 -2.98662  1.75669
H  -1.05257 -3.32960  0.83923
H  -0.95975 -3.77026  2.58121
C  -0.61656 -1.23667  3.44273
H  -0.95492 -0.21928  3.69938
H   0.48361 -1.21857  3.37840
H  -0.90771 -1.91781  4.27587
C  -2.10820  2.60949  1.81646
H  -2.38953  3.67746  1.98588
H  -1.94939  2.17316  2.82037
C   0.27887  2.96733  1.93600
H   0.16316  4.03423  2.23575
H   1.23405  2.86027  1.39732
H   0.32408  2.35669  2.85441
C  -0.85495  3.29331 -0.16285
H   0.12395  3.21053 -0.66185
H  -1.06366  4.37114  0.03039
H  -1.62489  2.91264 -0.85413
H  -4.20720  2.13736  1.73409
O   1.26353 -0.42654 -0.86925
C   3.05300 -0.18016 -0.99820
C   3.12583  1.31301 -0.74738
C   2.60787  2.22613 -1.70096
C   3.76452  1.85681  0.39104
C   2.74998  3.61035 -1.53776
H   2.09953  1.83278 -2.58560
C   3.90384  3.24690  0.55792
H   4.17923  1.18266  1.14633
C   3.40062  4.13492 -0.40420
H   2.35339  4.28757 -2.30354
H   4.41808  3.63255  1.44600
H   3.51559  5.21698 -0.27951
C   3.60618 -1.03336  0.13771
C   4.97043 -1.40026  0.10585
C   2.84920 -1.43049  1.25917
C   5.55106 -2.14340  1.14623
H   5.57098 -1.09108 -0.75542
C   3.42340 -2.17660  2.30118
H   1.78917 -1.16694  1.27189
C   4.77963 -2.53799  2.25051
H   6.61068 -2.41687  1.09079
H   2.80912 -2.48536  3.15605
H   5.22828 -3.12294  3.06076
```

D-Na

```
SCF (BP86) Energy = -1434.19031160
Enthalpy 0K = -1433.453351
Enthalpy 298K = -1433.406108
Free Energy 298K = -1433.538222
```
Lowest Frequency = 15.4665 cm^{-1}
Second Frequency = 16.1916 cm^{-1}
```
SCF (BP86-D3BJ) Energy = -
1434.38758010
SCF (Bnz) Energy = -1434.19794598
SCF (BS2) Energy = -1882.26273882
```

```
Si  0.02388  0.74140  3.03741
N  -2.45624 -0.88949 -0.69480
N  -0.44188 -1.03133 -2.98215
N  -0.89027 -3.37482  0.34912
N  -4.36518  2.24657  0.43875
C  -2.83177 -1.07392 -2.10547
H  -3.82549 -0.63173 -2.34667
H  -2.93073 -2.15435 -2.30759
C  -2.98260 -1.92403  0.21681
H  -2.80891 -1.55914  1.24387
C  -2.71275  0.48324 -0.18550
H  -2.33476  1.18910 -0.94651
H  -2.06837  0.62008  0.70490
C  -4.18674  0.81777  0.14059
H  -4.87042  0.48233 -0.68212
H  -4.48590  0.25370  1.04149
C  -4.39684  3.06470 -0.77292
H  -3.47471  2.92586 -1.36084
H  -4.45730  4.13158 -0.49850
H  -5.26581  2.82938 -1.43805
C  -5.56625  2.48337  1.24093
H  -6.51327  2.19301  0.72173
H  -5.63643  3.55578  1.49231
H  -5.50173  1.91681  2.18475
C   4.97272  1.93298  0.65577
H   5.11807  3.01651  0.61780
H   5.72388  1.34446  1.19164
C   0.81963 -0.44959  4.32408
H   0.23717 -1.38347  4.42385
H   0.88174  0.01141  5.32681
H   1.84356 -0.72820  4.01713
C  -1.73219  1.15887  3.70685
H  -1.69306  1.64826  4.69722
H  -2.34865  0.24700  3.81312
H  -2.26058  1.83923  3.01482
```

C   1.03110 2.37473 3.14203
H   2.07606 2.22479 2.81705
H   1.05341 2.76780 4.17494
H   0.59449 3.15652 2.49582
Na 0.04164 -1.04868 -0.31971
C -1.80587 -0.46271 -3.07810
H -2.20420 -0.56226 -4.11725
H -1.71846 0.62042 -2.88078
C   0.48866 -0.19418 -3.76203
H   1.50295 -0.62438 -3.71225
H   0.52458 0.82350 -3.33966
H   0.20113 -0.12490 -4.83707
C -0.40722 -2.40963 -3.49740
H -1.05551 -3.06442 -2.89419
H   0.62122 -2.80223 -3.43137
H -0.73553 -2.47519 -4.56156
C -2.33636 -3.31160 0.05705
H -2.90253 -4.01755 0.71381
H -2.48259 -3.67654 -0.97730
C -0.37060 -4.69311 -0.04549
H -0.84701 -5.53051 0.51637
H   0.71544 -4.73042 0.14003
H -0.54383 -4.86214 -1.12260
C -0.62255 -3.13540 1.78526
H   0.44017 -3.34550 1.98779
H -1.24208 -3.79097 2.44028
H -0.78223 -2.07000 2.03113
H -4.08331 -2.06776 0.11267
O -0.03286 0.10251 1.52359
C   3.90226 1.34948 0.06167
C   3.68588 -0.12305 0.15321
C   3.97578 -0.83893 1.33663
C   3.22906 -0.84950 -0.97223
C   3.83600 -2.23309 1.38918
H   4.28794 -0.28612 2.22793
C   3.09517 -2.24666 -0.92309
H   3.01625 -0.30737 -1.89980
C   3.39979 -2.94538 0.25938
H   4.05981 -2.76471 2.31991
H   2.77897 -2.79565 -1.81774
H   3.30553 -4.03593 0.29640
C   2.93565 2.16594 -0.73523
C   3.40816 3.07202 -1.71124
C   1.54219 2.07400 -0.49898
C   2.51620 3.87632 -2.43755
H   4.48511 3.13077 -1.90393
C   0.65866 2.89452 -1.22010
H   1.13761 1.40429 0.28406
C   1.13604 3.79191 -2.19241
H   2.90049 4.56939 -3.19392
H -0.41258 2.84640 -0.99344
H   0.43863 4.42791 -2.74886

1-Li system

1-Li (κ^{3})
SCF (BP86) Energy = -864.815383418
Enthalpy 0K = -864.265671
Enthalpy 298K = -864.232005
Free Energy 298K = -864.329724
Lowest Frequency = 20.0014 cm^{-1}
Second Frequency = 23.9368 cm^{-1}
SCF (BP86-D3BJ) Energy = -864.935852562
SCF (Bnz) Energy = -864.821867771
SCF (BS2) Energy = -1150.65388071

Si -2.10693 2.62885 -0.48874
N   1.10817 -1.14799 0.12796
N -1.30996 -1.36275 1.91453
N -0.87895 -2.54512 -1.57516
N   3.92757 1.43779 -0.26575
C   1.15227 -1.66213 1.51610
H   2.13618 -1.48620 2.00027
H   1.02422 -2.75801 1.48424
C   1.50790 -2.14661 -0.88484
H   1.74985 -1.59686 -1.81081
C   1.79065 0.15851 -0.04209
H   1.35843 0.85297 0.69730
H   1.50564 0.56221 -1.02907
C   3.33346 0.13541 0.06027
H   3.65904 -0.22427 1.07027
H   3.72705 -0.59448 -0.67147
C   3.65887 2.44931 0.76000
H   2.57656 2.62756 0.86314
H   4.12167 3.40406 0.45878
H   4.06542 2.17261 1.76460
C   5.36839 1.31145 -0.48923
H   5.93177 0.96661 0.41294
H   5.78220 2.28893 -0.79092
H   5.56011 0.59351 -1.30539
C -1.72706 0.95165 -1.18907
H -0.89567 1.08566 -1.92370
H -2.60836 0.65271 -1.80459
C -3.78891 2.64774 0.44710
H -4.61226 2.36851 -0.23470
H -4.01951 3.64975 0.85065
H -3.80731 1.93530 1.28997
C -2.22690 4.08545 -1.75312
H -2.44255 5.05524 -1.26721
H -3.02591 3.89580 -2.49216
H -1.28286 4.19691 -2.31668
C -0.75478 3.20500 0.76477
H -0.67119 2.51281 1.62347
H -0.95894 4.21249 1.16943
H   0.23315 3.24400 0.26928
Li -1.06919 -0.78077 -0.23307
C   0.05370 -1.03976 2.39241
H   0.20128 -1.35638 3.45188
H   0.15575 0.05959 2.37199
C -2.29363 -0.43303 2.50610
H -3.29855 -0.67423 2.12394
H -2.05679 0.59611 2.19663
H -2.31367 -0.49325 3.61808
C -1.68203 -2.74579 2.25541
H -0.97488 -3.46381 1.80917
H -2.68621 -2.96391 1.85644
H -1.69980 -2.91876 3.35610
C   0.39627 -3.17135 -1.17243
H   0.76707 -3.89067 -1.94148
H   0.19748 -3.76698 -0.26202
C -1.97482 -3.52800 -1.53616
H -1.82738 -4.36429 -2.25721
H -2.92376 -3.02384 -1.78241
H -2.06012 -3.95583 -0.52348
C -0.79566 -1.95950 -2.92796
H -1.75495 -1.47829 -3.17099
H -0.56276 -2.72931 -3.69924
H -0.02790 -1.17245 -2.96099

H   2.42880 -2.70676 -0.60704

1'-Li (κ4)
SCF (BP86) Energy = -864.816498657
Enthalpy 0K = -864.266747
Enthalpy 298K = -864.233465
Free Energy 298K = -864.328771
Lowest Frequency = 16.2568 cm^{-1}
Second Frequency = 33.1197 cm^{-1}
SCF (BP86-D3BJ) Energy = -864.945854349
SCF (Bnz) Energy = -864.821503005
SCF (BS2) Energy = -1150.65380933

```
Si  3.54847 -0.28664 -0.07411
Li -0.16949 -0.09885 -0.02685
N  -2.67624  0.37881  0.11730
N  -0.58606  1.40993 -1.74315
N  -0.47645  0.81905  2.03829
N  -1.42029 -2.28389 -0.33322
C  -2.87943  1.56359 -0.73667
H  -2.60584  2.45899 -0.15387
H  -3.95022  1.69912 -1.01812
C  -2.03031  1.51671 -2.01797
H  -2.31894  0.63465 -2.61833
H  -2.27302  2.40964 -2.64287
C  -2.93312  0.65695  1.54176
H  -3.05341 -0.31013  2.05848
H  -3.89131  1.20733  1.69826
C  -1.79666  1.44825  2.21143
H  -1.73775  2.45962  1.76900
H  -2.05626  1.59812  3.28702
C  -3.43738 -0.78719 -0.36665
H  -3.46194 -0.74303 -1.46869
H  -4.50340 -0.75700 -0.03797
C  -2.82620 -2.12870  0.07036
H  -2.86322 -2.21113  1.17226
H  -3.47218 -2.95297 -0.32050
C   0.14949  0.97278 -2.94323
H   1.20799  0.82912 -2.67784
H   0.07660  1.70719 -3.77817
H  -0.24717  0.00536 -3.29208
C  -0.02622  2.69101 -1.28011
H  -0.54607  3.03420 -0.37112
H  -0.11294  3.49112 -2.05183
H   1.03731  2.54823 -1.03187
C   0.60620  1.76585  2.35596
H   0.50206  2.67325  1.73877
H   1.56987  1.29370  2.10967
H   0.60628  2.06831  3.42850
C  -0.31988 -0.38585  2.87132
H  -1.11546 -1.11471  2.64730
H  -0.35581 -0.15283  3.96093
H   0.64711 -0.85494  2.62864
C  -0.78921 -3.39605  0.39438
H   0.27732 -3.44164  0.12363
H  -1.26353 -4.37978  0.16858
H  -0.85734 -3.21933  1.48069
C  -1.28562 -2.51845 -1.77825
H  -1.73212 -1.68848 -2.34964
H  -1.77739 -3.46573 -2.10398
H  -0.21469 -2.56595 -2.03280
C   1.80220 -0.89457 -0.27036
H   1.76477 -1.85251  0.30183
H   1.68303 -1.19986 -1.33949
C   4.90965 -1.25690 -1.04570
H   4.92878 -2.31781 -0.73788
H   5.92297 -0.84269 -0.88793
H   4.70829 -1.23811 -2.13240
C   3.77986  1.54556 -0.63392
H   3.46895  1.68898 -1.68553
H   4.83319  1.87159 -0.56088
H   3.17636  2.23030 -0.01058
C   4.11481 -0.36091  1.76677
H   3.49994  0.27588  2.42756
H   5.16345 -0.03229  1.87896
H   4.04993 -1.39407  2.15313
```

3-Li
SCF (BP86) Energy = -2988.86476076
Enthalpy 0K = -2987.564348
Enthalpy 298K = -2987.471700
Free Energy 298K = -2987.697127
Lowest Frequency = 5.4797 cm^{-1}
Second Frequency = 12.0733 cm^{-1}
SCF (BP86-D3BJ) Energy = -2989.38389447
SCF (Bnz) Energy = -2988.87365058
SCF (BS2) Energy = -4132.08386252

```
Si -5.61311 -0.44198 -1.75150
Si -2.44828  2.90275  3.62759
Si  2.39329 -3.38882  3.35654
Si  5.69495  0.53146 -1.37194
O  -1.46131  0.00771 -0.77794
O  -0.10611  1.50153  1.31679
O   0.01283 -1.47857  1.32624
O   1.49222  0.03126 -0.68462
C  -2.54135  0.07763 -1.70285
C  -2.44207 -1.09820 -2.71418
C  -2.43976  1.45221 -2.41171
C   0.25087  2.52688  2.21714
C   0.01353  3.93966  1.62782
C   1.76955  2.32952  2.54957
C  -0.34051 -2.56652  2.15485
C  -0.25881 -3.91886  1.39784
C  -1.81292 -2.33277  2.63303
C   2.63312  0.02392 -1.53595
C   2.58554 -1.30424 -2.33368
C   2.58886  1.26671 -2.46747
Li  0.01821 -1.24465 -0.65014
Li  1.25083  0.00095  1.22704
Li -1.33208  0.01928  1.15308
Li  0.01228  1.29360 -0.64470
C  -2.78758  4.77080  3.47587
H  -2.18944  5.34576  4.20282
H  -2.55773  5.15846  2.47098
H  -3.85332  4.97044  3.68708
C  -3.01946  2.35927  5.36918
H  -2.47108  2.90350  6.15766
H  -4.09532  2.56060  5.51243
H  -2.85558  1.28015  5.52874
C  -3.46114  1.96318  2.31678
H  -3.06493  2.16552  1.30776
H  -3.44210  0.87393  2.50306
H  -4.52048  2.27359  2.33608
C  -0.58278  2.43341  3.54657
H  -0.09156  3.04399  4.33092
H  -0.52820  1.38679  3.90565
C   0.60686 -2.67244  3.40241
```

H	0.10926	-3.28801	4.17958
H	0.69524	-1.65743	3.83880
C	2.43291	-5.27994	3.13305
H	1.77220	-5.78163	3.86030
H	2.11693	-5.58740	2.12376
H	3.45897	-5.65290	3.30064
C	3.09567	-3.00598	5.09528
H	2.50472	-3.50310	5.88418
H	4.13688	-3.36082	5.18720
H	3.09114	-1.92330	5.31142
C	3.51616	-2.56384	2.06102
H	3.13063	-2.71756	1.04025
H	3.59949	-1.47825	2.24284
H	4.53514	-2.98676	2.10918
C	6.90273	0.04500	0.02786
H	6.65193	0.57062	0.96523
H	6.86675	-1.03878	0.23408
H	7.94343	0.30189	-0.23576
C	5.89880	2.39575	-1.71680
H	6.94542	2.62469	-1.98424
H	5.25343	2.73069	-2.54505
H	5.64042	2.99450	-0.82674
C	3.92954	0.15763	-0.67192
H	4.00416	-0.72859	-0.01460
H	3.74169	1.00924	0.01127
C	6.19637	-0.42436	-2.94613
H	7.21827	-0.12490	-3.23973
H	6.19535	-1.51610	-2.79747
H	5.52641	-0.19944	-3.79148
C	-5.78501	-2.28812	-2.19731
H	-5.08332	-2.58145	-2.99504
H	-5.58682	-2.93233	-1.32327
H	-6.80986	-2.50500	-2.54654
C	-6.89470	-0.03003	-0.39356
H	-6.68976	-0.59405	0.53271
H	-6.88042	1.04374	-0.13783
H	-7.91801	-0.28222	-0.72183
C	-6.04197	0.58958	-3.29907
H	-7.05941	0.32435	-3.63758
H	-6.02438	1.67387	-3.10405
H	-5.34970	0.38563	-4.13162
C	-3.89249	-0.09986	-0.93627
H	-4.01124	0.74750	-0.23497
H	-3.74090	-0.98347	-0.28695
C	4.52596	1.82636	3.10321
H	5.58160	1.64755	3.33141
C	3.52582	1.04634	3.70580
H	3.79814	0.25291	4.41014
C	2.16989	1.29531	3.43038
H	1.40866	0.69405	3.93975
C	4.15037	2.84315	2.21212
H	4.91624	3.46268	1.73281
C	2.79387	3.08399	1.93440
H	2.52731	3.89373	1.24943
C	0.47800	5.09607	2.29588
H	1.04838	4.98943	3.22543
C	0.23611	6.37820	1.78253
H	0.61427	7.25677	2.31688
C	-0.48852	6.53713	0.58825
H	-0.67781	7.53786	0.18554
C	-0.97194	5.40065	-0.07677
H	-1.54915	5.49956	-1.00175
C	-0.72120	4.11640	0.44143
H	-1.13951	3.24110	-0.06653
C	-3.18168	3.75619	-2.82463

H	-3.92183	4.54658	-2.65679
C	-2.07382	3.99224	-3.65163
H	-1.94072	4.96474	-4.13736
C	-1.13685	2.96385	-3.85173
H	-0.25849	3.12870	-4.48326
C	-3.35871	2.50212	-2.21169
H	-4.23265	2.34876	-1.57401
C	-1.31961	1.71716	-3.23530
H	-0.58598	0.92290	-3.41016
C	2.26228	2.51668	-1.89382
H	2.03336	2.56709	-0.82217
C	2.27316	3.69676	-2.65219
H	2.01832	4.64922	-2.17547
C	2.61209	3.65428	-4.01574
H	2.62567	4.57268	-4.61229
C	2.93553	2.42105	-4.60321
H	3.20025	2.37155	-5.66534
C	2.92615	1.24135	-3.83633
H	3.18297	0.29029	-4.31252
C	1.53077	-1.51708	-3.25305
H	0.81072	-0.71268	-3.43598
C	1.39333	-2.72653	-3.95002
H	0.56575	-2.85032	-4.65529
C	2.30862	-3.77146	-3.73502
H	2.21127	-4.71476	-4.28302
C	3.34933	-3.58900	-2.81262
H	4.07063	-4.39337	-2.63013
C	3.48314	-2.37054	-2.12190
H	4.30621	-2.25798	-1.41218
C	-2.12577	-2.38357	-2.22162
H	-1.92762	-2.51036	-1.15071
C	-2.09485	-3.50641	-3.06187
H	-1.84732	-4.48751	-2.64321
C	-2.38001	-3.36746	-4.43102
H	-2.36062	-4.24003	-5.09265
C	-2.69063	-2.09666	-4.94058
H	-2.91103	-1.97155	-6.00659
C	-2.72340	-0.97603	-4.09081
H	-2.96790	0.00572	-4.50751
C	0.51243	-4.04117	0.22632
H	1.07231	-3.17494	-0.14357
C	0.62673	-5.27058	-0.44829
H	1.23624	-5.32740	-1.35603
C	-0.03134	-6.40802	0.04410
H	0.05077	-7.36627	-0.47981
C	-0.79166	-6.30617	1.22214
H	-1.30580	-7.18739	1.62150
C	-0.89977	-5.07756	1.89019
H	-1.50308	-5.01261	2.80230
C	-4.22905	-2.44217	2.18033
H	-5.04551	-2.74008	1.51354
C	-2.89904	-2.67103	1.79219
H	-2.69998	-3.16031	0.83368
C	-4.51341	-1.85614	3.42438
H	-5.54949	-1.68937	3.73635
C	-3.45001	-1.49713	4.26659
H	-3.65306	-1.04723	5.24442
C	-2.12007	-1.72923	3.87328
H	-1.31358	-1.46057	4.56158

A-Li
SCF (BP86) Energy = -1441.43980885
Enthalpy 0K = -1440.703810
Enthalpy 298K = -1440.656893
Free Energy 298K = -1440.786796

Lowest Frequency = 11.4101 cm^{-1}
Second Frequency = 14.2586 cm^{-1}
SCF (BP86-D3BJ) Energy = -1441.62709442
SCF (Bnz) Energy = -1441.44559310
SCF (BS2) Energy = -1727.41995482

```
Si  0.74603  1.64671 -2.55926
N   2.67370 -1.18287  0.64328
N   0.78910 -0.74235  2.86846
N   0.47173 -2.94081 -0.45479
N   5.36172  1.40274 -0.28399
C   2.99958 -1.57583  2.02735
H   4.08921 -1.51858  2.24781
H   2.72249 -2.63542  2.15927
C   2.90045 -2.26255 -0.33700
H   2.85473 -1.80363 -1.34001
C   3.30756  0.09089  0.22939
H   3.06104  0.84943  0.99150
H   2.79783  0.42527 -0.68888
C   4.83571  0.05683 -0.00981
H   5.37321 -0.42768  0.84435
H   5.04160 -0.56719 -0.89794
C   5.51763  2.19578  0.93605
H   4.55626  2.28470  1.46710
H   5.84525  3.21585  0.67369
H   6.26641  1.76342  1.64586
C   6.62594  1.34962 -1.01943
H   7.45068  0.85047 -0.45328
H   6.95580  2.37506 -1.25953
H   6.48396  0.80700 -1.96922
C  -0.08646  0.76595 -1.14666
H  -0.87872  0.07725 -1.50295
H  -0.57476  1.51773 -0.49746
C   1.93762  3.02193 -1.94431
H   1.43982  3.66503 -1.19648
H   2.25057  3.67188 -2.78109
H   2.85406  2.61434 -1.48292
C  -0.48545  2.52385 -3.76187
H   0.03712  3.03236 -4.59326
H  -1.08317  3.28163 -3.22515
H  -1.19247  1.79818 -4.20193
C   1.77490  0.47474 -3.69059
H   2.59501 -0.01827 -3.13767
H   2.23427  1.03330 -4.52572
H   1.14493 -0.31631 -4.13570
Li  0.33946 -0.71457  0.50116
C   2.25020 -0.72394  3.06314
H   2.53932 -1.06390  4.08663
H   2.57964  0.32680  2.98167
C   0.15393  0.36739  3.59656
H  -0.92122  0.37686  3.36190
H   0.58819  1.32427  3.26196
H   0.28862  0.28795  4.70035
C   0.19730 -2.01325  3.31342
H   0.64759 -2.85899  2.76918
H  -0.88153 -2.00135  3.09165
H   0.33905 -2.18805  4.40600
C   1.86143 -3.38970 -0.24314
H   2.14510 -4.18874 -0.97028
H   1.91012 -3.85926  0.75680
C  -0.45874 -3.99374 -0.01626
H  -0.31416 -4.94972 -0.57176
H  -1.49291 -3.65371 -0.17937
H  -0.32210 -4.19605  1.05964
C   0.22420 -2.64399 -1.88005
H  -0.82717 -2.34355 -2.00758
H   0.42578 -3.52982 -2.52697
H   0.84459 -1.79835 -2.20979
H   3.91063 -2.72480 -0.24661
O  -1.69251 -0.36814  1.05120
C  -2.76691  0.11843  0.60831
C  -3.62829 -0.69761 -0.30633
C  -4.29427 -0.13809 -1.42166
C  -3.75430 -2.08515 -0.06535
C  -5.05516 -0.94908 -2.27551
H  -4.18018  0.92918 -1.63459
C  -4.53285 -2.89030 -0.90779
H  -3.24918 -2.51084  0.80724
C  -5.18251 -2.32543 -2.01910
H  -5.54622 -0.50615 -3.14838
H  -4.63778 -3.96010 -0.69664
H  -5.78463 -2.95484 -2.68274
C  -3.22195  1.47027  1.04252
C  -4.58425  1.85858  1.00763
C  -2.27535  2.37374  1.58575
C  -4.98036  3.11481  1.48775
H  -5.33586  1.16267  0.62364
C  -2.67196  3.63116  2.04994
H  -1.22558  2.06956  1.60576
C  -4.02775  4.00801  2.00476
H  -6.03869  3.39557  1.46206
H  -1.92395  4.32623  2.44625
H  -4.33831  4.99136  2.37326
```

TS(A-B)-Li
SCF (BP86) Energy = -1441.43630306
Enthalpy 0K = -1440.699996
Enthalpy 298K = -1440.654057
Free Energy 298K = -1440.780389
Lowest Frequency = -120.3950 cm^{-1}
Second Frequency = 12.8900 cm^{-1}
SCF (BP86-D3BJ) Energy = -1441.62844573
SCF (Bnz) Energy = -1441.44222786
SCF (BS2) Energy = -1727.41650411

```
Si  0.11163  2.57652 -1.62926
N   2.70816 -1.24935  0.18134
N   1.10898 -2.00280  2.54670
N   0.51347 -2.73051 -1.26852
N   4.90413  1.84556 -0.42617
C   3.27658 -2.04477  1.29044
H   4.36657 -1.87236  1.42614
H   3.17019 -3.11402  1.03938
C   2.88606 -1.89121 -1.13804
H   2.68485 -1.12197 -1.90338
C   3.15324  0.16738  0.18419
H   2.94592  0.57283  1.18952
H   2.48355  0.71702 -0.50229
C   4.62829  0.41945 -0.20444
H   5.32511 -0.01817  0.55669
H   4.84070 -0.09903 -1.15708
C   4.82722  2.63087  0.80761
H   3.81276  2.58630  1.23448
H   5.04274  3.68826  0.58041
H   5.55243  2.29214  1.58920
C   6.20737  2.02926 -1.06645
H   7.06405  1.67347 -0.44219
H   6.36637  3.10009 -1.27971
```

```
H   6.23343  1.48303 -2.02485
C  -0.24780  1.06685 -0.62185
H  -0.69751  0.17503 -1.06589
H  -0.39067  1.17851  0.45518
C   0.56281  4.02728 -0.46355
H  -0.25760  4.23332  0.24515
H   0.76278  4.95394 -1.02937
H   1.46683  3.79531  0.12599
C  -1.37749  3.14632 -2.70976
H  -1.11453  4.01669 -3.33805
H  -2.23412  3.43332 -2.07519
H  -1.71389  2.33880 -3.38390
C   1.57353  2.35353 -2.86089
H   2.53090  2.23506 -2.32274
H   1.67041  3.22821 -3.52976
H   1.42853  1.46473 -3.50186
Li  0.38434 -1.21459  0.48573
C   2.56420 -1.76248  2.62177
H   3.04496 -2.36420  3.42889
H   2.70907 -0.70238  2.89454
C   0.40832 -1.33103  3.65594
H  -0.67749 -1.42422  3.50219
H   0.66137 -0.25815  3.65314
H   0.68260 -1.75377  4.64932
C   0.79522 -3.44083  2.58207
H   1.29509 -3.96755  1.75291
H  -0.29233 -3.57692  2.46837
H   1.11451 -3.91934  3.53700
C   1.94512 -3.08664 -1.34811
H   2.19056 -3.56471 -2.32626
H   2.14026 -3.85394 -0.57624
C  -0.30009 -3.95159 -1.11828
H  -0.17084 -4.65432 -1.97297
H  -1.36261 -3.67021 -1.05632
H  -0.02090 -4.47850 -0.19065
C   0.08240 -2.01898 -2.49121
H  -0.99196 -1.78957 -2.41630
H   0.25364 -2.63366 -3.40465
H   0.62089 -1.06442 -2.58970
H   3.92820 -2.24414 -1.31063
O  -1.47204 -0.93733  1.10235
C  -2.41451 -0.20241  0.65073
C  -3.20571 -0.69637 -0.52798
C  -3.64660  0.13820 -1.58136
C  -3.52903 -2.07281 -0.58056
C  -4.38651 -0.38870 -2.64888
H  -3.35724  1.19333 -1.57587
C  -4.27713 -2.59763 -1.64624
H  -3.20038 -2.71691  0.24156
C  -4.70854 -1.75817 -2.68608
H  -4.70289  0.27082 -3.46425
H  -4.52929 -3.66399 -1.66119
H  -5.28644 -2.16647 -3.52194
C  -2.91705  0.94824  1.45237
C  -4.17478  1.56238  1.22076
C  -2.15909  1.38999  2.56773
C  -4.62812  2.60249  2.04324
H  -4.80715  1.21109  0.40092
C  -2.61263  2.42913  3.38378
H  -1.20185  0.90033  2.76353
C  -3.85041  3.04847  3.12471
H  -5.60254  3.06161  1.84311
H  -2.00190  2.76117  4.23095
H  -4.20703  3.86207  3.76501
```

B-Li
SCF (BP86) Energy = -1441.49416881
Enthalpy 0K = -1440.753913
Enthalpy 298K = -1440.708585
Free Energy 298K = -1440.834620
Lowest Frequency = 10.8163 cm^{-1}
Second Frequency = 14.2297 cm^{-1}
SCF (BP86-D3BJ) Energy = -1441.68986969
SCF (Bnz) Energy = -1441.49991013
SCF (BS2) Energy = -1727.47335401

```
Si -3.30369  2.04091 -1.41412
N   3.23440 -0.03837 -0.04637
N   2.12645 -1.84047  2.13472
N   2.08849 -2.32259 -1.53337
N   3.58743  3.73190 -0.68359
C   3.94742 -0.38256  1.20579
H   4.66242  0.40959  1.51258
H   4.55757 -1.28393  1.02067
C   3.87589 -0.57411 -1.26381
H   3.51122  0.01843 -2.12045
C   2.86951  1.39402 -0.15015
H   2.28333  1.64562  0.74907
H   2.17000  1.50380 -0.99664
C   4.04601  2.37769 -0.34745
H   4.71340  2.38272  0.55253
H   4.66624  2.02579 -1.19325
C   2.88355  4.37622  0.42826
H   1.97735  3.81046  0.69628
H   2.56362  5.38579  0.11996
H   3.51469  4.48150  1.34563
C   4.70779  4.56277 -1.12609
H   5.48679  4.71756 -0.33905
H   4.33433  5.55563 -1.43040
H   5.19586  4.09891 -2.00075
C  -1.64516  1.18553 -0.93410
H  -1.09851  0.93810 -1.86545
H  -1.05055  1.99950 -0.47468
C  -3.88747  3.22114 -0.03330
H  -4.13898  2.67229  0.88858
H  -4.78178  3.78378 -0.35470
H  -3.10146  3.95476  0.21621
C  -4.74675  0.87645 -1.87379
H  -5.61911  1.47631 -2.18891
H  -5.05816  0.25617 -1.01795
H  -4.48815  0.19715 -2.70264
C  -2.90640  3.10111 -2.96045
H  -2.07750  3.80252 -2.76121
H  -3.78171  3.69761 -3.27229
H  -2.60968  2.47087 -3.81717
Li  1.32568 -1.16243  0.14462
C   2.97195 -0.64766  2.36412
H   3.54166 -0.72929  3.32013
H   2.29386  0.21768  2.46740
C   0.93874 -1.80008  3.01502
H   0.36107 -2.72930  2.88008
H   0.29885 -0.95645  2.70856
H   1.21428 -1.71530  4.09028
C   2.88033 -3.08181  2.36879
H   3.76087 -3.13783  1.70745
H   2.23624 -3.94923  2.14970
H   3.23634 -3.17003  3.42130
C   3.54142 -2.06008 -1.48757
H   4.05392 -2.40747 -2.41647
```

H   3.95720 -2.65992 -0.65669
C   1.81664 -3.76949 -1.45425
H   2.24415 -4.33117 -2.31586
H   0.72696 -3.93406 -1.43448
H   2.24968 -4.17791 -0.52577
C   1.47000 -1.76983 -2.75478
H   0.39232 -1.99491 -2.74314
H   1.92223 -2.19588 -3.67965
H   1.57749 -0.67430 -2.77933
H   4.98325 -0.46359 -1.26050
O -0.10470 -0.15143 0.35696
C -1.45190 -0.02244 0.06190
C -1.91304 -1.37847 -0.54196
C -2.18727 -1.58189 -1.90996
C -1.92431 -2.51948 0.29662
C -2.44857 -2.86689 -2.42502
H -2.19866 -0.72781 -2.59327
C -2.19295 -3.80000 -0.20539
H -1.72772 -2.38310 1.36522
C -2.45155 -3.98432 -1.57760
H -2.66220 -2.98801 -3.49352
H -2.21148 -4.65913 0.47570
H -2.66681 -4.98267 -1.97380
C -2.26548 0.31269 1.34919
C -3.58639 -0.12130 1.58389
C -1.65570 1.12999 2.32271
C -4.27509 0.24959 2.75271
H -4.07792 -0.77197 0.85277
C -2.34100 1.51387 3.48504
H -0.62327 1.44646 2.14053
C -3.65712 1.07247 3.70765
H -5.29710 -0.11075 2.91775
H -1.84727 2.15645 4.22379
H -4.19292 1.36257 4.61818

TS(B-C)-Li

SCF (BP86) Energy = -1441.48760128
Enthalpy 0K = -1440.747604
Enthalpy 298K = -1440.703235
Free Energy 298K = -1440.825260
Lowest Frequency = -46.0964 cm^{-1}
Second Frequency = 15.7515 cm^{-1}
SCF (BP86-D3BJ) Energy = -1441.68433250
SCF (Bnz) Energy = -1441.49377341
SCF (BS2) Energy = -1727.46666012

Si -1.63335 2.87763 -1.08708
N   2.99489 -0.47744 0.04328
N   1.82457 -2.31788 2.17408
N   1.62715 -2.62383 -1.47664
N   3.64923 3.28319 -0.40241
C   3.70626 -0.92609 1.26372
H   4.47567 -0.19498 1.58931
H   4.25343 -1.85552 1.02788
C   3.54870 -1.01938 -1.21460
H   3.19799 -0.37126 -2.03637
C   2.76569 0.98531 0.00252
H   2.26899 1.26428 0.94681
H   2.02951 1.19400 -0.79277
C   4.00999 1.86652 -0.25312
H   4.77963 1.71833 0.54644
H   4.48046 1.54655 -1.20112
C   3.27395 3.89934 0.87350
H   2.40070 3.39042 1.31113
H   2.98975 4.95079 0.70178
H   4.10112 3.88345 1.62571
C   4.73452 4.03973 -1.03025
H   5.67428 4.05552 -0.42545
H   4.41463 5.08452 -1.18325
H   4.97059 3.60752 -2.01774
C -1.18861 1.00807 -1.11273
H -1.45408 0.66923 -2.13359
H -0.08349 1.00014 -1.09476
C -1.37895 3.73747 0.59505
H -2.11911 3.40754 1.34029
H -1.46478 4.83233 0.47667
H -0.37550 3.52019 0.99971
C -3.40151 3.23291 -1.71767
H -3.55328 4.31906 -1.84728
H -4.16097 2.86689 -1.00840
H -3.58475 2.75296 -2.69514
C -0.40310 3.67264 -2.32447
H   0.63813 3.57062 -1.96947
H -0.60830 4.75028 -2.45189
H -0.46689 3.20283 -3.32186
Li 1.01643 -1.48489 0.26259
C   2.73381 -1.17750 2.42894
H   3.31073 -1.32075 3.37322
H   2.10403 -0.28119 2.56997
C   0.64592 -2.24668 3.06746
H   0.04977 -3.16630 2.94585
H   0.01664 -1.39603 2.75447
H   0.93395 -2.16145 4.13889
C   2.52015 -3.60086 2.36239
H   3.39003 -3.67957 1.68932
H   1.83338 -4.43006 2.12627
H   2.88255 -3.73637 3.40742
C   3.09621 -2.46709 -1.47718
H   3.54998 -2.81504 -2.43569
H   3.49528 -3.12613 -0.68390
C   1.25471 -4.04946 -1.42465
H   1.60234 -4.61321 -2.31987
H   0.15749 -4.13260 -1.36053
H   1.69583 -4.51633 -0.52808
C   1.00117 -1.99607 -2.65784
H -0.09058 -2.12541 -2.59645
H   1.36854 -2.44208 -3.61008
H   1.20220 -0.91380 -2.67284
H   4.66055 -0.99134 -1.25100
O -0.42457 -0.50762 0.62958
C -1.56799 -0.09655 -0.02108
C -2.26699 -1.32476 -0.70388
C -3.02262 -1.27517 -1.89454
C -2.14877 -2.57694 -0.06182
C -3.60810 -2.43425 -2.43941
H -3.16511 -0.32206 -2.41564
C -2.73850 -3.73415 -0.59147
H -1.57597 -2.60754 0.87076
C -3.46608 -3.67098 -1.79411
H -4.17842 -2.36432 -3.37297
H -2.64007 -4.68925 -0.06119
H -3.92238 -4.57295 -2.21645
C -2.60780 0.47335 1.00694
C -3.97000 0.67797 0.70206
C -2.16972 0.78221 2.30789
C -4.85930 1.18998 1.66163
H -4.35020 0.41351 -0.29064
C -3.05237 1.29684 3.27156
H -1.11236 0.61107 2.53008

```
C  -4.40422  1.50514  2.95288
H  -5.91483  1.33200  1.40206
H  -2.68440  1.53326  4.27727
H  -5.09815  1.89953  3.70345
```

C-Li
SCF (BP86) Energy = -1441.48880618
Enthalpy 0K = -1440.748611
Enthalpy 298K = -1440.703367
Free Energy 298K = -1440.827301
Lowest Frequency = 11.8538 cm^{-1}
Second Frequency = 23.4402 cm^{-1}
SCF (BP86-D3BJ) Energy = -1441.68912649
SCF (Bnz) Energy = -1441.49420267
SCF (BS2) Energy = -1727.46743742

```
Si -0.09532  3.07186 -1.39710
N   2.42849 -1.32153  0.19621
N   0.29971 -2.60348  1.98028
N   0.51684 -2.68274 -1.67290
N   4.84793  1.67229  0.29487
C   2.70284 -2.11920  1.41677
H   3.65332 -1.81540  1.90201
H   2.83951 -3.17568  1.12900
C   2.85658 -1.98006 -1.05657
H   2.87180 -1.20547 -1.84345
C   2.91744  0.07581  0.30634
H   2.51014  0.48610  1.24530
H   2.45596  0.66548 -0.50256
C   4.45173  0.25875  0.24558
H   4.95570 -0.33559  1.05094
H   4.81810 -0.14839 -0.71471
C   4.63089  2.26800  1.61578
H   3.56039  2.26278  1.87412
H   4.95671  3.32137  1.59739
H   5.19538  1.74728  2.42886
C   6.24454  1.83420 -0.11289
H   6.96929  1.31399  0.56103
H   6.50434  2.90656 -0.12316
H   6.38528  1.43938 -1.13385
C  -1.68435  2.48009 -0.50493
H  -1.70336  3.13905  0.38506
H  -2.59405  2.76074 -1.06816
C   1.23165  3.39573 -0.06478
H   1.00194  4.31336  0.50469
H   2.24862  3.49171 -0.48165
H   1.21531  2.54321  0.63344
C  -0.51964  4.73486 -2.25040
H   0.37893  5.19240 -2.70077
H  -0.93693  5.45892 -1.52905
H  -1.26397  4.59986 -3.05475
C   0.60476  1.89656 -2.72513
H   0.79280  0.92178 -2.24924
H   1.54904  2.28913 -3.14146
H  -0.09781  1.73888 -3.56072
Li  0.18618 -1.24782  0.04806
C   1.56838 -2.00143  2.44702
H   1.89880 -2.45180  3.41386
H   1.37135 -0.93181  2.64136
C  -0.81589 -2.16370  2.84295
H  -1.74886 -2.64234  2.50233
H  -0.94634 -1.07424  2.76093
H  -0.65635 -2.44069  3.91009
C   0.37163 -4.07350  2.01401
H   1.17837 -4.44838  1.36373
H  -0.57994 -4.49592  1.65207
H   0.55272 -4.46160  3.04338
C   1.91478 -3.11990 -1.47585
H   2.32566 -3.60409 -2.39357
H   1.91065 -3.89854 -0.69170
C  -0.38316 -3.85124 -1.69882
H  -0.14450 -4.54842 -2.53438
H  -1.42147 -3.50316 -1.81789
H  -0.30390 -4.40585 -0.75002
C   0.36207 -1.93826 -2.93925
H  -0.67816 -1.58910 -3.03152
H   0.61323 -2.57130 -3.82130
H   1.01169 -1.05055 -2.94983
H   3.89005 -2.39024 -1.00175
O  -0.51511  0.41132  0.20777
C  -1.76750  0.98347  0.01258
C  -2.55450  0.07923 -0.97944
C  -2.74097  0.38832 -2.34263
C  -3.03512 -1.17389 -0.52925
C  -3.37363 -0.51099 -3.22133
H  -2.39545  1.35124 -2.73000
C  -3.68020 -2.07015 -1.39440
H  -2.92554 -1.42613  0.53153
C  -3.85048 -1.74497 -2.75345
H  -3.50650 -0.23545 -4.27402
H  -4.07097 -3.01777 -1.00447
H  -4.36019 -2.43798 -3.43153
C  -2.56450  1.10523  1.35208
C  -3.97183  1.17061  1.41527
C  -1.83959  1.26980  2.54927
C  -4.63249  1.36892  2.63974
H  -4.55893  1.05938  0.49670
C  -2.49337  1.47778  3.77407
H  -0.74644  1.23757  2.48081
C  -3.89707  1.52171  3.82642
H  -5.72791  1.40441  2.66564
H  -1.90703  1.60811  4.69168
H  -4.41218  1.67753  4.78074
```

TS(B-C′)-Li
SCF (BP86) Energy = -1441.48618817
Enthalpy 0K = -1440.746369
Enthalpy 298K = -1440.701925
Free Energy 298K = -1440.824702
Lowest Frequency = -43.4935 cm^{-1}
Second Frequency = 9.0259 cm^{-1}
SCF (BP86-D3BJ) Energy = -1441.68200752
SCF (Bnz) Energy = -1441.49253009
SCF (BS2) Energy = -1727.46562353

```
Si  3.07540 -0.17803  2.38728
N  -2.95972 -0.72341  0.10776
N  -1.78370 -2.17733 -2.30571
N  -1.47216 -3.03823  1.22462
N  -3.78854  2.81121  1.35321
C  -3.67164 -0.96853 -1.17071
H  -4.44253 -0.19554 -1.36800
H  -4.21786 -1.92498 -1.09109
C  -3.47594 -1.51519  1.24179
H  -3.15399 -1.01491  2.17161
C  -2.78816  0.71679  0.42155
H  -2.29339  1.18507 -0.44551
H  -2.06833  0.79948  1.25479
```

```
C  -4.07622  1.47721  0.81354
H  -4.78482  1.52721 -0.05329
H  -4.59711  0.91060  1.60850
C  -3.24968  3.72191  0.33805
H  -2.29669  3.34747 -0.06659
H  -3.04707  4.70176  0.80207
H  -3.95292  3.88612 -0.51602
C  -4.98255  3.38712  1.97213
H  -5.82298  3.55222  1.25299
H  -4.73047  4.36203  2.42364
H  -5.34784  2.72320  2.77508
C   1.55716  0.42784  1.38051
H   0.72709 -0.21900  1.72028
H   1.31830  1.42349  1.80264
C   2.46371 -0.21380  4.20549
H   2.12541  0.78356  4.53679
H   3.26922 -0.53599  4.88863
H   1.61776 -0.91133  4.33723
C   4.57163  1.00620  2.33185
H   5.32604  0.70014  3.07815
H   4.27247  2.04237  2.56876
H   5.05086  1.00673  1.34044
C   3.64515 -1.94247  1.93845
H   2.80840 -2.66004  2.00755
H   4.43424 -2.28184  2.63251
H   4.04073 -1.98785  0.91155
Li -0.94214 -1.54347 -0.29945
C  -2.70425 -1.01826 -2.36536
H  -3.28578 -1.01123 -3.31774
H  -2.08453 -0.10455 -2.35706
C  -0.61911 -1.95638 -3.19330
H  -0.00780 -2.87388 -3.21641
H  -0.00000 -1.15007 -2.76322
H  -0.92360 -1.71760 -4.23664
C  -2.47420 -3.41757 -2.69312
H  -3.33720 -3.61219 -2.03478
H  -1.78030 -4.26982 -2.60467
H  -2.84720 -3.38330 -3.74271
C  -2.94946 -2.96107  1.22491
H  -3.38992 -3.51762  2.08590
H  -3.30286 -3.46714  0.30816
C  -1.02484 -4.39862  0.87598
H  -1.36047 -5.16204  1.61395
H   0.07639 -4.42081  0.83260
H  -1.41724 -4.67605 -0.11591
C  -0.90722 -2.65508  2.53233
H   0.19160 -2.70847  2.48167
H  -1.25984 -3.32101  3.35260
H  -1.17677 -1.61725  2.78173
H  -4.58743 -1.55291  1.28135
O   0.43339 -0.48738 -0.57765
C   1.39053  0.43013 -0.21044
C   2.75378  0.09569 -0.92171
C   2.91782 -1.18674 -1.47999
C   3.81596  1.01362 -1.05998
C   4.10665 -1.55404 -2.13166
H   2.07460 -1.87829 -1.39035
C   5.00593  0.65516 -1.71609
H   3.70920  2.03165 -0.67143
C   5.16024 -0.63347 -2.25271
H   4.20834 -2.56110 -2.55396
H   5.81133  1.39185 -1.81765
H   6.08595 -0.91177 -2.76847
C   0.98553  1.86579 -0.69496
C   1.40305  3.06551 -0.07990
C   0.18020  1.97595 -1.84720
C   1.01890  4.32078 -0.58514
H   2.03970  3.03294  0.81110
C  -0.20426  3.22336 -2.36183
H  -0.13349  1.04603 -2.33021
C   0.21055  4.40803 -1.72843
H   1.35453  5.23321 -0.07885
H  -0.82572  3.27393 -3.26397
H  -0.08894  5.38486 -2.12360
```

C′-Li

SCF (BP86) Energy = -1441.49337520
Enthalpy 0K = -1440.753087
Enthalpy 298K = -1440.707756
Free Energy 298K = -1440.834007
Lowest Frequency = 3.4763 cm^{-1}
Second Frequency = 11.3412 cm^{-1}
SCF (BP86-D3BJ) Energy = -1441.69156360
SCF (Bnz) Energy = -1441.49925083
SCF (BS2) Energy = -1727.47224866

```
Si  3.75869 -0.67248  1.92365
N  -2.81397 -1.09595 -0.16690
N  -1.07806 -1.58020 -2.63814
N  -0.85588 -3.15312  0.70260
N  -4.98663  1.77263  1.20781
C  -3.32514 -1.33286 -1.53955
H  -4.31235 -0.85196 -1.70210
H  -3.49494 -2.41544 -1.67127
C  -3.16922 -2.17590  0.77979
H  -3.03619 -1.77461  1.79939
C  -3.18153  0.24966  0.35057
H  -2.88139  0.99014 -0.40971
H  -2.55731  0.46503  1.23448
C  -4.67171  0.42019  0.73352
H  -5.33452  0.12150 -0.11946
H  -4.90341 -0.27663  1.55966
C  -5.01082  2.75010  0.11768
H  -4.02164  2.82273 -0.36097
H  -5.25106  3.74608  0.52683
H  -5.77113  2.50977 -0.66702
C  -6.25863  1.78796  1.93196
H  -7.13548  1.49779  1.30154
H  -6.44803  2.80206  2.32383
H  -6.21146  1.09473  2.78951
C   2.27634  0.52713  1.69406
H   1.48271  0.17119  2.38000
H   2.62082  1.50101  2.09980
C   3.95048 -0.91222  3.81579
H   4.14530  0.04845  4.32385
H   4.79289 -1.58760  4.04744
H   3.04020 -1.34717  4.26474
C   5.40263  0.04828  1.27900
H   6.24326 -0.60603  1.57175
H   5.59208  1.04878  1.70401
H   5.40582  0.14540  0.18176
C   3.46613 -2.38413  1.12605
H   2.80363 -3.00340  1.75586
H   4.41336 -2.93536  0.99458
H   2.97989 -2.24824  0.14717
Li -0.50725 -1.34087 -0.47121
C  -2.35143 -0.82701 -2.61413
H  -2.85524 -0.85110 -3.60991
H  -2.10317  0.22954 -2.40464
```

C -0.06512 -0.83674 -3.41357
H 0.86865 -1.41955 -3.44530
H 0.15713 0.11529 -2.91021
H -0.39652 -0.64351 -4.45905
C -1.25509 -2.91566 -3.23185
H -1.99065 -3.50652 -2.66227
H -0.29456 -3.45581 -3.20802
H -1.60027 -2.86544 -4.29040
C -2.30048 -3.43267 0.61009
H -2.62719 -4.19027 1.36255
H -2.48543 -3.88297 -0.38300
C -0.06946 -4.32163 0.27153
H -0.23574 -5.21134 0.92094
H 1.00196 -4.06619 0.29777
H -0.34054 -4.59303 -0.76252
C -0.45519 -2.76455 2.06861
H 0.61506 -2.51187 2.06364
H -0.64484 -3.57699 2.80697
H -0.99870 -1.86185 2.38698
H -4.23588 -2.48473 0.70571
O 0.95918 -0.43256 -0.12030
C 1.61296 0.71411 0.27709
C 2.75111 1.12275 -0.70888
C 3.14293 0.23144 -1.72222
C 3.42690 2.35772 -0.60601
C 4.19091 0.55004 -2.60373
H 2.59782 -0.71570 -1.78703
C 4.47040 2.68427 -1.48508
H 3.12250 3.07659 0.16396
C 4.86030 1.77881 -2.48828
H 4.48477 -0.16157 -3.38478
H 4.98118 3.64948 -1.39010
H 5.67540 2.03312 -3.17486
C 0.56827 1.87990 0.30617
C -0.04880 2.34193 1.48703
C 0.13911 2.45083 -0.91540
C -1.04726 3.33245 1.45437
H 0.26503 1.93802 2.45470
C -0.85216 3.44182 -0.95754
H 0.62781 2.13435 -1.84344
C -1.45173 3.89303 0.23332
H -1.50030 3.67616 2.39121
H -1.13958 3.88420 -1.91902
H -2.20957 4.68344 0.20952

TS(C-D)-Li
SCF (BP86) Energy = -1441.44854249
Enthalpy 0K = -1440.710225
Enthalpy 298K = -1440.665438
Free Energy 298K = -1440.787976
Lowest Frequency = -397.6375 cm^{-1}
Second Frequency = 11.9532 cm^{-1}
SCF (BP86-D3BJ) Energy = -1441.65308822
SCF (Bnz) Energy = -1441.45420419
SCF (BS2) Energy = -1727.42970748

Si 0.78893 -0.05675 2.52206
N -3.07255 -0.01769 -0.97994
N -0.89763 2.17491 -0.94015
N -0.59492 -1.49218 -1.82400
N -6.36822 -0.68275 0.88703
C -3.17397 1.31406 -1.61240
H -4.22630 1.66405 -1.68512
H -2.81641 1.23597 -2.65323
C -3.09708 -1.12630 -1.94871
H -3.32784 -2.05038 -1.39042
C -4.03098 -0.20700 0.13057
H -3.88515 0.62772 0.83684
H -3.74630 -1.12062 0.68164
C -5.52324 -0.33133 -0.26221
H -5.88173 0.60199 -0.76639
H -5.62769 -1.14525 -1.00319
C -6.49270 0.41642 1.84752
H -5.50859 0.68710 2.26209
H -7.12745 0.09405 2.69002
H -6.94997 1.33632 1.40553
C -7.69303 -1.12136 0.44477
H -8.26937 -0.32851 -0.09267
H -8.28863 -1.43662 1.31869
H -7.59454 -1.98813 -0.23129
C 3.16505 -0.11481 2.35306
H 3.52919 0.78019 2.86578
H 3.55932 -1.04635 2.77018
C -1.16858 -0.07872 2.23999
H -1.54992 0.77340 1.64232
H -1.67596 -0.01526 3.22102
H -1.52278 -1.01428 1.76462
C 0.94749 1.61364 3.47298
H -0.03202 1.98402 3.82760
H 1.38996 2.39232 2.82796
H 1.61084 1.50011 4.34695
C 0.88147 -1.57476 3.71198
H 1.13435 -2.50169 3.16797
H -0.08380 -1.74493 4.22361
H 1.66463 -1.42830 4.47366
Li -0.38681 0.09878 -0.31256
C -2.36167 2.38418 -0.87076
H -2.63548 3.38961 -1.27035
H -2.64685 2.37968 0.19665
C -0.22396 3.06887 0.02950
H 0.86247 2.89949 -0.00775
H -0.57081 2.83699 1.04865
H -0.43205 4.14100 -0.18558
C -0.38910 2.47860 -2.29286
H -0.82174 1.79226 -3.03903
H 0.70497 2.35670 -2.30035
H -0.62906 3.52177 -2.60176
C -1.76932 -1.30391 -2.70015
H -1.88586 -2.16501 -3.40158
H -1.57017 -0.41690 -3.33033
C 0.62728 -1.55965 -2.65150
H 0.59641 -2.40289 -3.37782
H 1.50259 -1.69346 -1.99666
H 0.74293 -0.62018 -3.21845
C -0.71042 -2.73455 -1.03142
H 0.22086 -2.87717 -0.46440
H -0.87805 -3.62623 -1.67736
H -1.54091 -2.65825 -0.31181
H -3.90071 -1.02340 -2.71431
O 1.12684 -0.13055 0.81177
C 2.96538 -0.08338 0.92916
C 3.39224 -1.35953 0.22974
C 2.73464 -2.58541 0.48151
C 4.55884 -1.39579 -0.57032
C 3.21421 -3.79303 -0.04958
H 1.83994 -2.57280 1.10803
C 5.03342 -2.60130 -1.11057
H 5.10953 -0.46926 -0.75998
C 4.36352 -3.80904 -0.85671

H   2.68497 -4.72759 0.16974
H   5.93893 -2.59436 -1.72774
H   4.73566 -4.74979 -1.27621
C   3.22358 1.19755 0.15765
C   3.64864 2.38631 0.79080
C   3.07734 1.23636 -1.25055
C   3.90555 3.55793 0.05967
H   3.79926 2.39599 1.87323
C   3.35776 2.39595 -1.98908
H   2.75831 0.33005 -1.77339
C   3.76912 3.57106 -1.33678
H   4.23373 4.46008 0.58775
H   3.26216 2.37935 -3.08121
H   3.99010 4.47768 -1.90993

D-Li
SCF (BP86) Energy = -1441.52836149
Enthalpy 0K = -1440.789467
Enthalpy 298K = -1440.742659
Free Energy 298K = -1440.876883
Lowest Frequency = 7.1056 cm^{-1}
Second Frequency = 9.2423 cm^{-1}
SCF (BP86-D3BJ) Energy = -1441.71181353
SCF (Bnz) Energy = -1441.53504698
SCF (BS2) Energy = -1727.51389536

Si 1.41315 2.65949 1.23148
N   2.88554 -1.42109 -0.60668
N   0.55202 -2.00196 1.21719
N   0.54737 -0.69544 -2.27322
N   6.49233 -0.21199 -0.05372
C   2.69847 -2.69795 0.12151
H   3.66272 -3.20339 0.34198
H   2.13807 -3.38811 -0.53258
C   2.89490 -1.57873 -2.07584
H   3.34660 -0.66618 -2.50209
C   4.02271 -0.61367 -0.10432
H   3.87374 -0.47626 0.97977
H   3.94677 0.39382 -0.54986
C   5.43562 -1.17225 -0.39599
H   5.58764 -2.15366 0.12210
H   5.51989 -1.37507 -1.47978
C   6.61430 -0.00366 1.39143
H   5.68476 0.41748 1.80685
H   7.41993 0.72366 1.58724
H   6.85442 -0.94156 1.95138
C   7.77664 -0.63043 -0.61737
H   8.14515 -1.60570 -0.21360
H   8.54178 0.13457 -0.40047
H   7.69149 -0.72636 -1.71365
C -3.21346 0.65141 2.07951
H -3.35129 -0.03410 2.92169
H -2.53758 1.49807 2.22675
C   2.68608 2.27673 2.62392
H   2.21841 1.69445 3.43843
H   3.09983 3.19863 3.07154
H   3.53703 1.68789 2.23507
C   0.06784 3.76330 2.04090
H -0.42921 3.23097 2.87183
H -0.71244 4.04390 1.31200
H   0.49337 4.69620 2.45296
C   2.33395 3.75575 -0.05051
H   3.16903 3.20342 -0.51847
H   2.75553 4.66766 0.40999
H   1.65329 4.07610 -0.85950
Li 0.96086 -0.31215 -0.15012
C   1.92677 -2.50047 1.43614
H   1.93165 -3.45450 2.01438
H   2.45316 -1.75317 2.05568
C   0.02245 -1.37845 2.44740
H -0.99517 -1.00388 2.25216
H   0.64826 -0.51199 2.71000
H -0.01429 -2.09545 3.29887
C -0.34840 -3.08134 0.78007
H   0.01536 -3.53877 -0.15514
H -1.35179 -2.66731 0.59088
H -0.44222 -3.88822 1.54308
C   1.48294 -1.77012 -2.65595
H   1.56755 -1.87077 -3.76475
H   1.05645 -2.72253 -2.28968
C -0.83666 -1.06820 -2.61624
H -0.98127 -1.20908 -3.71172
H -1.52009 -0.27513 -2.27229
H -1.10573 -2.00907 -2.10756
C   0.88801 0.58660 -2.92145
H   0.15330 1.34502 -2.61346
H   0.88489 0.50470 -4.03266
H   1.87705 0.93760 -2.58988
H   3.52672 -2.42795 -2.42029
O   0.78305 1.29761 0.56083
C -3.85698 0.45614 0.89862
C -3.62199 1.37502 -0.25535
C -2.34193 1.93402 -0.49442
C -4.69336 1.74994 -1.10144
C -2.16195 2.86152 -1.53377
H -1.46722 1.64183 0.10745
C -4.50321 2.67303 -2.13994
H -5.68792 1.32650 -0.92641
C -3.23579 3.23714 -2.35860
H -1.16670 3.29173 -1.69130
H -5.35005 2.95787 -2.77392
H -3.08622 3.96118 -3.16693
C -4.82210 -0.67348 0.74219
C -5.66195 -1.06627 1.81063
C -4.90161 -1.40307 -0.46786
C -6.52873 -2.16080 1.68404
H -5.64477 -0.48635 2.73933
C -5.76702 -2.49936 -0.59428
H -4.27149 -1.10392 -1.31166
C -6.58314 -2.88533 0.48178
H -7.17479 -2.44062 2.52321
H -5.80524 -3.05385 -1.53833
H -7.26469 -3.73646 0.38046

Na system

[Na]**A**

SCF (BP86) Energy = -739.816200020
Enthalpy 0K = -739.498275
Enthalpy 298K = -739.472796
Free Energy 298K = -739.559110
Lowest Frequency = 7.3612 cm^{-1}
Second Frequency = 11.6345 cm^{-1}
SCF (BP86-D3BJ) Energy = -739.895551246
SCF (Bnz) Energy = -739.826277469
SCF (BS2) Energy = -1187.71811873

```
Si  4.78273 -0.55226 -0.05134
C   3.61914 -0.04291 -1.40258
H   3.66934  1.03593 -1.64339
H   3.75451 -0.61829 -2.33795
C   6.65938 -0.29333 -0.40655
H   6.97006 -0.86531 -1.29827
H   7.29011 -0.61691  0.44153
H   6.87695  0.77069 -0.60506
C   4.56867 -2.41943  0.35556
H   5.29586 -2.75647  1.11528
H   4.71805 -3.03961 -0.54621
H   3.55874 -2.64383  0.74727
C   4.40854  0.42488  1.56368
H   4.45392  1.51432  1.38657
H   5.13453  0.18709  2.36113
H   3.40174  0.19447  1.96056
Na  1.40689 -0.49936 -0.55317
O  -0.67979 -0.89647  0.20546
C  -1.74617 -0.22500  0.13392
C  -3.05544 -0.92590  0.09024
C  -4.26835 -0.30750  0.48281
C  -3.07508 -2.29075 -0.29026
C  -5.46734 -1.03247  0.47542
H  -4.26231  0.73156  0.82482
C  -4.27590 -3.00564 -0.30943
H  -2.12944 -2.76502 -0.56829
C  -5.47698 -2.37866  0.07227
H  -6.39647 -0.54812  0.79285
H  -4.28047 -4.05616 -0.61814
H  -6.41629 -2.94120  0.06280
C  -1.65868  1.26355  0.07946
C  -0.58393  1.90562  0.74477
C  -2.53798  2.04744 -0.70671
C  -0.40866  3.29147  0.64662
H   0.08031  1.30745  1.37790
C  -2.34747  3.43151 -0.81755
H  -3.34789  1.56143 -1.25908
C  -1.28872  4.05801 -0.13731
H   0.41390  3.77538  1.18286
H  -3.02370  4.02335 -1.44291
H  -1.14732  5.14035 -0.22054
```

[Na]**TS(A-B)**

SCF (BP86) Energy = -739.799955189
Enthalpy 0K = -739.481043
Enthalpy 298K = -739.457550
Free Energy 298K = -739.533279
Lowest Frequency = -87.6420 cm^{-1}
Second Frequency = 24.1286 cm^{-1}
SCF (BP86-D3BJ) Energy = -739.891013816
SCF (Bnz) Energy = -739.812527635
SCF (BS2) Energy = -1187.70193344

```
Si -2.40599  1.58836 -0.39363
C  -0.59307  1.31458  0.01711
H  -0.01746  1.41297 -0.91843
H  -0.19105  2.08201  0.70925
C  -2.88650  3.42315 -0.68364
H  -2.66330  4.03851  0.20517
H  -3.96132  3.53421 -0.91624
H  -2.31035  3.84081 -1.52676
C  -3.59026  1.01857  1.05018
H  -4.61715  1.34170  0.80736
H  -3.36691  1.48739  2.03091
H  -3.66380 -0.08231  1.17209
C  -2.94583  0.57306 -1.91756
H  -2.38604  0.89243 -2.81372
H  -4.02055  0.70625 -2.13138
H  -2.75391 -0.50351 -1.77214
Na -1.33898 -0.09168  1.98946
O   0.82280 -0.26355  2.16776
C   0.93025 -0.33880  0.88797
C   2.11435  0.34798  0.24812
C   2.72659 -0.07898 -0.95047
C   2.68942  1.43527  0.94496
C   3.85849  0.58371 -1.45192
H   2.33545 -0.94917 -1.48499
C   3.81284  2.10012  0.44128
H   2.23507  1.73116  1.89494
C   4.40175  1.68019 -0.76504
H   4.32310  0.23222 -2.37981
H   4.23789  2.94577  0.99342
H   5.28310  2.19728 -1.15937
C   0.33473 -1.57870  0.22204
C   0.16097 -2.71878  1.04206
C  -0.04979 -1.67219 -1.13625
C  -0.38638 -3.90766  0.52781
H   0.49038 -2.65533  2.08495
C  -0.58346 -2.86085 -1.65166
H   0.03603 -0.79090 -1.77759
C  -0.75939 -3.98495 -0.82191
H  -0.50510 -4.77872  1.18225
H  -0.87868 -2.90800 -2.70564
H  -1.18349 -4.90931 -1.22799
```

[Na]**B**

SCF (BP86) Energy = -739.828101984
Enthalpy 0K = -739.506505
Enthalpy 298K = -739.482886
Free Energy 298K = -739.558693
Lowest Frequency = 32.6989 cm^{-1}
Second Frequency = 37.6753 cm^{-1}
SCF (BP86-D3BJ) Energy = -739.922158472
SCF (Bnz) Energy = -739.846123522
SCF (BS2) Energy = -1187.73242458

```
Si  1.76521  2.04516  0.00320
C   0.65841  0.99941  1.18060
H   1.33434  0.63064  1.97658
H  -0.01020  1.70771  1.70911
C   0.94279  2.49569 -1.66286
H   0.74175  1.60945 -2.28702
H   1.60518  3.16627 -2.23843
H  -0.01693  3.01885 -1.51427
```

```
C   3.45215  1.21749 -0.32258
H   4.09863  1.87521 -0.92989
H   3.34202  0.25650 -0.84963
H   3.97552  1.01771  0.62850
C   2.09598  3.68647  0.93344
H   1.16126  4.24749  1.10892
H   2.77606  4.34103  0.36068
H   2.56141  3.49971  1.91681
Na -2.98903 -0.71784  1.77770
O  -0.87813 -0.70211  1.94657
C  -0.21877 -0.25364  0.81143
C   0.70899 -1.34989  0.22245
C   1.13623 -1.37924 -1.12082
C   1.15349 -2.36701  1.08781
C   1.98962 -2.39531 -1.58555
H   0.78549 -0.60929 -1.81807
C   2.01902 -3.37437  0.63393
H   0.78230 -2.33739  2.11749
C   2.43924 -3.39484 -0.70689
H   2.30028 -2.40718 -2.63667
H   2.36119 -4.15297  1.32590
H   3.10611 -4.18609 -1.06689
C  -1.40085  0.07273 -0.14897
C  -2.05178 -0.98320 -0.84563
C  -2.09490  1.30856 -0.06686
C  -3.35086 -0.83614 -1.36113
H  -1.52908 -1.93958 -0.95363
C  -3.39583  1.46030 -0.58066
H  -1.62102  2.15554  0.43884
C  -4.04630  0.38183 -1.21171
H  -3.82229 -1.67383 -1.88870
H  -3.90257  2.42861 -0.49339
H  -5.05770  0.49944 -1.61423
```

^{Na}**TS(B–C)**

SCF (BP86) Energy = -739.818843175
Enthalpy 0K = -739.497737
Enthalpy 298K = -739.474754
Free Energy 298K = -739.548963
Lowest Frequency = -54.5675 cm^{-1}
Second Frequency = 31.8162 cm^{-1}
SCF (BP86-D3BJ) Energy = -739.911086798
SCF (Bnz) Energy = -739.836936864
SCF (BS2) Energy = -1187.72358682

```
Si  2.42353 -1.54463 -0.04212
C   0.67486 -1.32311  0.72265
H   0.16040 -2.22365  0.33417
H   0.78683 -1.51504  1.80663
C   2.52473 -1.05910 -1.88589
H   1.75835 -1.59641 -2.47127
H   3.51086 -1.32856 -2.30313
H   2.37055  0.02066 -2.04224
C   2.75479 -3.42598  0.08193
H   3.75578 -3.68235 -0.30710
H   2.01152 -4.00177 -0.49608
H   2.70295 -3.77333  1.12842
C   3.85140 -0.70961  0.93171
H   3.95173  0.37420  0.75475
H   4.81031 -1.17350  0.63970
H   3.72537 -0.86376  2.01735
Na -0.52268  2.60591  1.85346
O  -0.47464  0.48453  1.92726
C  -0.36233 -0.10740  0.68299
C  -1.71135 -0.72071  0.19831
C  -1.88591 -1.25095 -1.09799
C  -2.79018 -0.77498  1.09619
C  -3.11290 -1.81078 -1.48693
H  -1.05340 -1.21860 -1.81241
C  -4.01689 -1.34641  0.71689
H  -2.61661 -0.36612  2.09755
C  -4.18516 -1.86205 -0.57812
H  -3.23370 -2.21051 -2.50047
H  -4.84450 -1.39135  1.43494
H  -5.14141 -2.30420 -0.87896
C   0.02070  1.10739 -0.22101
C  -0.96414  1.87437 -0.89759
C   1.29693  1.70990 -0.05687
C  -0.70660  3.19117 -1.32824
H  -1.95343  1.43666 -1.06465
C   1.56333  3.01719 -0.49353
H   2.07297  1.15342  0.47531
C   0.55223  3.78182 -1.11546
H  -1.49644  3.75574 -1.83784
H   2.56273  3.44470 -0.34987
H   0.75526  4.80258 -1.45556
```

^{Na}**C**

SCF (BP86) Energy = -739.833080019
Enthalpy 0K = -739.511498
Enthalpy 298K = -739.488010
Free Energy 298K = -739.563425
Lowest Frequency = 28.5912 cm^{-1}
Second Frequency = 39.0033 cm^{-1}
SCF (BP86-D3BJ) Energy = -739.927825913
SCF (Bnz) Energy = -739.848724385
SCF (BS2) Energy = -1187.73541643

```
Si -2.26302 -1.56814  0.19819
C  -0.58940 -1.07350  1.00114
H  -0.70268 -0.63950  2.01251
H  -0.07497 -2.03980  1.16412
C  -2.04906 -2.98300 -1.05668
H  -1.37071 -2.65001 -1.85674
H  -3.01564 -3.28975 -1.49409
H  -1.60514 -3.86972 -0.57157
C  -3.33030 -0.17162 -0.60823
H  -4.40613 -0.37950 -0.46950
H  -3.14754 -0.13765 -1.69824
H  -3.13785  0.81850 -0.15555
C  -3.34983 -2.23535  1.63633
H  -2.83365 -3.04588  2.18012
H  -4.30653 -2.64209  1.26330
H  -3.58751 -1.44579  2.37163
Na -1.19170  1.51842 -1.85961
O  -0.26743 -0.31650 -1.27013
C   0.26717 -0.20661  0.01311
C   1.74302 -0.67989  0.08091
C   2.52228 -0.54337  1.24887
C   2.31654 -1.29095 -1.04701
C   3.84497 -1.01016  1.28642
H   2.08972 -0.06322  2.13548
C   3.63949 -1.76367 -1.01225
H   1.68335 -1.38017 -1.93598
C   4.40910 -1.62427  0.15390
H   4.43886 -0.89466  2.20048
H   4.07216 -2.24200 -1.89900
H   5.44158 -1.98982  0.18293
```

```
C   0.21158  1.31961  0.34711
C   1.10656  2.18498 -0.34013
C  -0.84978  1.92676  1.05966
C   0.91861  3.57549 -0.35728
H   1.95332  1.73839 -0.87331
C  -1.04502  3.32318  1.04353
H  -1.54089  1.30232  1.63459
C  -0.17450  4.15577  0.32120
H   1.63304  4.21380 -0.89031
H  -1.87863  3.75894  1.60646
H  -0.32243  5.24064  0.31119
```

[Na]**TS(C-D)**

SCF (BP86) Energy = -739.800984435
Enthalpy 0K = -739.481739
Enthalpy 298K = -739.458315
Free Energy 298K = -739.533505
Lowest Frequency = -388.5742 cm^{-1}
Second Frequency = 30.5723 cm^{-1}
SCF (BP86-D3BJ) Energy = -739.898181545
SCF (Bnz) Energy = -739.818192422
SCF (BS2) Energy = -1187.70415213

```
Si -1.49843 -1.97629  0.04244
C  -0.14364 -0.74715  1.80845
H  -0.81321 -0.27162  2.53271
H   0.58438 -1.43302  2.25124
C  -2.39692 -2.45280 -1.63214
H  -3.07504 -1.65542 -2.00597
H  -3.03466 -3.34502 -1.48670
H  -1.68166 -2.68472 -2.44093
C  -3.04062 -1.67567  1.15653
H  -3.87326 -2.33768  0.85335
H  -3.40710 -0.63580  1.06545
H  -2.81284 -1.84964  2.21936
C  -0.54563 -3.57464  0.47830
H   0.50188 -3.48447  0.14408
H  -0.98697 -4.45754 -0.01881
H  -0.53111 -3.74620  1.56620
Na -1.65809  0.97710 -1.67741
O  -0.63027 -0.65014 -0.68833
C   0.27758 -0.00714  0.64626
C   1.73671 -0.21079  0.24569
C   2.73438  0.16260  1.17478
C   2.14409 -0.73974 -0.99530
C   4.09556  0.01598  0.87135
H   2.42371  0.55880  2.14754
C   3.50870 -0.90024 -1.29526
H   1.36849 -1.04452 -1.70236
C   4.48953 -0.51995 -0.36710
H   4.85118  0.31039  1.60822
H   3.80386 -1.33067 -2.25909
H   5.55219 -0.64611 -0.60133
C  -0.20037  1.43024  0.48676
C   0.48781  2.35143 -0.34670
C  -1.44298  1.85560  1.02991
C  -0.05669  3.61274 -0.65615
H   1.45602  2.06104 -0.76651
C  -1.98853  3.11133  0.71949
H  -1.99494  1.17725  1.68673
C  -1.30788  3.99769 -0.14184
H   0.50499  4.29788 -1.30156
H  -2.95039  3.40361  1.15600
H  -1.73297  4.97757 -0.38181
```

[Na]**D**

SCF (BP86) Energy = -739.875079461
Enthalpy 0K = -739.554378
Enthalpy 298K = -739.529499
Free Energy 298K = -739.612382
Lowest Frequency = 9.7903 cm^{-1}
Second Frequency = 11.2877 cm^{-1}
SCF (BP86-D3BJ) Energy = -739.958816792
SCF (Bnz) Energy = -739.889762973
SCF (BS2) Energy = -1187.78250852

```
Si -3.39704 -0.11770  0.17703
C   2.97840 -0.19466  2.21455
H   3.34646  0.71701  2.69565
H   3.15862 -1.14028  2.73339
C  -4.73925  1.11851 -0.43560
H  -4.58859  2.12262  0.00286
H  -5.75941  0.79087 -0.16440
H  -4.70483  1.22333 -1.53528
C  -3.63448 -0.27312  2.07571
H  -4.65380 -0.61076  2.33721
H  -3.46535  0.69789  2.57548
H  -2.91970 -0.99724  2.50488
C  -3.84897 -1.82033 -0.58389
H  -3.77668 -1.78852 -1.68564
H  -4.87767 -2.12894 -0.32344
H  -3.16392 -2.60855 -0.22391
Na -0.60725  1.93075 -0.63093
O  -1.88196  0.36573 -0.23323
C   2.34217 -0.17008  1.01630
C   1.95277 -1.43399  0.31997
C   2.89419 -2.48324  0.20473
C   0.65110 -1.61335 -0.20875
C   2.54888 -3.68992 -0.42068
H   3.90760 -2.33480  0.59401
C   0.31544 -2.82726 -0.82957
H  -0.14161 -0.84833 -0.10712
C   1.25608 -3.86455 -0.94122
H   3.29229 -4.48966 -0.50773
H  -0.70023 -2.95550 -1.21833
H   0.98404 -4.80549 -1.43170
C   2.08414  1.13256  0.33631
C   2.18508  1.24252 -1.07484
C   1.76245  2.30283  1.07116
C   2.00798  2.47904 -1.72117
H   2.41568  0.34658 -1.65902
C   1.58162  3.53973  0.42670
H   1.63646  2.22970  2.15593
C   1.70713  3.63723 -0.97451
H   2.11608  2.54119 -2.80932
H   1.34021  4.42893  1.01888
H   1.57976  4.60218 -1.47594
```

Li system

^{Li}**A**

SCF (BP86) Energy = -747.139619031
Enthalpy 0K = -746.820307
Enthalpy 298K = -746.795473
Free Energy 298K = -746.879294
Lowest Frequency = 6.8933 cm^{-1}
Second Frequency = 10.8070 cm^{-1}
SCF (BP86-D3BJ) Energy = -747.218509132
SCF (Bnz) Energy = -747.148344183
SCF (BS2) Energy = -1032.95138692

```
Si  4.43671 -0.64550 -0.00721
C   3.29347 -0.08322 -1.36227
H   3.34943  1.01069 -1.52809
H   3.51733 -0.58266 -2.32574
C   6.30507 -0.24314 -0.24829
H   6.68920 -0.71138 -1.17133
H   6.91954 -0.60398  0.59656
H   6.46290  0.84566 -0.34142
C   4.31199 -2.54929  0.22016
H   4.99487 -2.91474  1.00719
H   4.57032 -3.07592 -0.71565
H   3.28890 -2.86081  0.50162
C   3.92753  0.15963  1.66540
H   3.93452  1.26209  1.59354
H   4.61246 -0.12291  2.48426
H   2.90991 -0.14831  1.97224
Li  1.40526 -0.59700 -0.76794
O  -0.40622 -0.88989 -0.39196
C  -1.44988 -0.21241 -0.17066
C  -2.74764 -0.91665 -0.02068
C  -3.83910 -0.35608  0.68768
C  -2.87097 -2.22470 -0.55141
C  -5.02691 -1.08221  0.84439
H  -3.74045  0.63394  1.14206
C  -4.06348 -2.93873 -0.40435
H  -2.01631 -2.65512 -1.08096
C  -5.14535 -2.36936  0.29309
H  -5.85992 -0.64640  1.40514
H  -4.15373 -3.94315 -0.83026
H  -6.07677 -2.93234  0.41285
C  -1.35533  1.26938 -0.08488
C  -0.17027  1.85827  0.42274
C  -2.37477  2.11190 -0.59293
C  -0.01806  3.24976  0.43761
H   0.61520  1.22120  0.84299
C  -2.20920  3.50294 -0.59265
H  -3.27616  1.66862 -1.02638
C  -1.03548  4.07495 -0.07164
H   0.89555  3.69088  0.84767
H  -2.99437  4.14281 -1.00783
H  -0.91216  5.16272 -0.06589
```

^{Li}**TS(A-B)**

SCF (BP86) Energy = -747.131037539
Enthalpy 0K = -746.810457
Enthalpy 298K = -746.787562
Free Energy 298K = -746.861814
Lowest Frequency = -71.3115 cm^{-1}
Second Frequency = 22.6597 cm^{-1}
SCF (BP86-D3BJ) Energy = -747.220548330
SCF (Bnz) Energy = -747.138594950
SCF (BS2) Energy = -1032.94236354

```
Si  2.59067 -1.47335 -0.32376
C   0.71728 -1.31582 -0.29980
H   0.31883 -0.97740 -1.26550
H   0.19411 -2.25792 -0.03610
C   3.26444 -3.12576 -1.01578
H   2.85576 -3.98653 -0.45904
H   4.36655 -3.17283 -0.95659
H   2.97403 -3.24706 -2.07320
C   3.21275 -1.40212  1.52418
H   4.29717 -1.60243  1.55089
H   2.75959 -2.17544  2.17908
H   3.09596 -0.40904  2.00711
C   3.40451 -0.01481 -1.23753
H   3.18811 -0.06918 -2.31836
H   4.50123 -0.01917 -1.11514
H   3.01745  0.95054 -0.87106
Li  0.94553 -0.80028  1.68794
O  -0.58867 -0.02124  2.14036
C  -0.82218  0.22872  0.88813
C  -2.03901 -0.41224  0.26855
C  -2.78762  0.18100 -0.77163
C  -2.51209 -1.62204  0.82825
C  -3.94970 -0.43759 -1.26073
H  -2.48140  1.14616 -1.18327
C  -3.66582 -2.24088  0.33702
H  -1.95923 -2.05703  1.66637
C  -4.38905 -1.65358 -0.71739
H  -4.51876  0.04271 -2.06404
H  -4.00930 -3.18180  0.78050
H  -5.29378 -2.13594 -1.10221
C  -0.28535  1.53861  0.34526
C   0.02797  2.54241  1.29028
C  -0.08659  1.82480 -1.02572
C   0.51816  3.79181  0.88100
H  -0.13142  2.31868  2.34927
C   0.40401  3.07269 -1.43351
H  -0.28770  1.05501 -1.77590
C   0.70764  4.06362 -0.48277
H   0.74766  4.55665  1.63099
H   0.56018  3.26982 -2.49959
H   1.09222  5.03691 -0.80528
```

^{Li}**B**

SCF (BP86) Energy = -747.154811306
Enthalpy 0K = -746.831385
Enthalpy 298K = -746.808383
Free Energy 298K = -746.882198
Lowest Frequency = 29.9213 cm^{-1}
Second Frequency = 41.5365 cm^{-1}
SCF (BP86-D3BJ) Energy = -747.248081045
SCF (Bnz) Energy = -747.171260850
SCF (BS2) Energy = -1032.96710981

```
Si  1.35711  2.16346 -0.10083
C   0.47047  1.06829  1.21341
H   1.25378  0.82313  1.95679
H  -0.22996  1.72063  1.77077
C   0.36453  2.45263 -1.70849
H   0.21483  1.52407 -2.28349
H   0.90916  3.16107 -2.35746
H  -0.63089  2.88250 -1.50660
```

```
C   3.08370  1.48333 -0.53562
H   3.61489  2.17530 -1.21255
H   3.02404  0.49820 -1.02487
H   3.69683  1.36787  0.37481
C   1.59093  3.86531  0.74240
H   0.62148  4.33832  0.97756
H   2.15206  4.55842  0.09169
H   2.15133  3.76729  1.68824
Li -2.39833 -1.18043  2.04140
O  -0.71646 -0.78400  2.20909
C  -0.26549 -0.29193  0.97267
C   0.71296 -1.29169  0.30707
C   0.93861 -1.34492 -1.08371
C   1.42252 -2.18175  1.13580
C   1.85601 -2.25790 -1.63180
H   0.37979 -0.67729 -1.74962
C   2.34968 -3.08582  0.59429
H   1.21224 -2.14716  2.20922
C   2.56991 -3.12905 -0.79273
H   2.00863 -2.29081 -2.71652
H   2.89735 -3.76620  1.25651
H   3.28597 -3.84069 -1.21802
C  -1.58503 -0.15699  0.15471
C  -2.20610 -1.33896 -0.34175
C  -2.36499  1.02503  0.15786
C  -3.53910 -1.33638 -0.79521
H  -1.61646 -2.26249 -0.38315
C  -3.69232  1.03179 -0.30742
H  -1.92952  1.95351  0.53765
C  -4.29283 -0.14846 -0.77817
H  -3.98114 -2.26267 -1.17973
H  -4.26119  1.96822 -0.29893
H  -5.32675 -0.14144 -1.13753
```

^{Li}TS(B-C)

SCF (BP86) Energy = -747.145874946
Enthalpy 0K = -746.822835
Enthalpy 298K = -746.800557
Free Energy 298K = -746.872506
Lowest Frequency = -50.3840 cm^{-1}
Second Frequency = 32.7862 cm^{-1}
SCF (BP86-D3BJ) Energy = -747.237357816
SCF (Bnz) Energy = -747.162493608
SCF (BS2) Energy = -1032.95878756

```
Si  2.26437 -1.53462 -0.16073
C   0.53260 -1.34365  0.65284
H  -0.04269 -2.12402  0.11832
H   0.62964 -1.73087  1.68465
C   2.35952 -0.78980 -1.91523
H   1.55857 -1.19656 -2.55681
H   3.32420 -1.04122 -2.38973
H   2.25956  0.30745 -1.90696
C   2.49605 -3.42798 -0.30869
H   3.47473 -3.67552 -0.75562
H   1.71328 -3.88048 -0.94149
H   2.44680 -3.91572  0.68030
C   3.73973 -0.90724  0.89289
H   3.93188  0.17385  0.79306
H   4.66079 -1.42739  0.57552
H   3.58710 -1.13029  1.96307
Li  0.00985  1.97154  2.31275
O  -0.46033  0.31232  2.16779
C  -0.40303 -0.06789  0.82311
C  -1.80875 -0.46614  0.29222
C  -2.02016 -0.88071 -1.04082
C  -2.90817 -0.43277  1.16620
C  -3.29975 -1.24459 -1.48772
H  -1.17485 -0.91354 -1.73969
C  -4.18952 -0.80664  0.72609
H  -2.71624 -0.12101  2.19745
C  -4.39188 -1.21058 -0.60274
H  -3.44501 -1.55872 -2.52760
H  -5.03332 -0.78466  1.42553
H  -5.39022 -1.50033 -0.94839
C   0.08782  1.23159  0.09772
C  -0.76269  2.08347 -0.64618
C   1.37261  1.74403  0.43556
C  -0.35324  3.37300 -1.03527
H  -1.75958  1.72877 -0.92278
C   1.78227  3.03483  0.05310
H   2.05628  1.10772  1.00855
C   0.91564  3.86133 -0.68672
H  -1.03970  4.00145 -1.61373
H   2.78592  3.38641  0.31888
H   1.23039  4.86464 -0.99094
```

^{Li}C

SCF (BP86) Energy = -747.158602344
Enthalpy 0K = -746.835302
Enthalpy 298K = -746.812538
Free Energy 298K = -746.885658
Lowest Frequency = 33.6542 cm^{-1}
Second Frequency = 38.6081 cm^{-1}
SCF (BP86-D3BJ) Energy = -747.251691131
SCF (Bnz) Energy = -747.170974427
SCF (BS2) Energy = -1032.97023596

```
Si -2.45325 -1.29057  0.15172
C  -0.65633 -1.07014  0.80534
H  -0.61633 -0.70256  1.84806
H  -0.25942 -2.10208  0.84597
C  -2.58685 -2.86360 -0.90861
H  -1.88192 -2.78783 -1.75173
H  -3.60792 -3.01129 -1.30137
H  -2.32076 -3.75841 -0.31976
C  -3.19337  0.17303 -0.88198
H  -4.28569  0.23292 -0.73040
H  -3.05170 -0.00381 -1.96704
H  -2.79338  1.16153 -0.58431
C  -3.60170 -1.47186  1.67438
H  -3.25949 -2.29102  2.33047
H  -4.63970 -1.69741  1.37315
H  -3.62287 -0.55010  2.28238
Li -1.03392  0.86646 -1.99910
O  -0.17456 -0.56356 -1.49357
C   0.24941 -0.27685 -0.18894
C   1.73188 -0.67487  0.03583
C   2.41123 -0.37763  1.23551
C   2.41390 -1.37373 -0.97519
C   3.74308 -0.77752  1.42161
H   1.89615  0.17779  2.02897
C   3.74748 -1.77607 -0.79090
H   1.86232 -1.58861 -1.89572
C   4.41686 -1.48046  0.40736
H   4.25845 -0.53824  2.35880
H   4.26666 -2.32218 -1.58716
H   5.45729 -1.79188  0.55144
```

C	0.13729	1.27519	0.01748
C	0.80540	2.10008	-0.92828
C	-0.66932	1.91766	0.98101
C	0.65480	3.49666	-0.92129
H	1.47255	1.61909	-1.65461
C	-0.81938	3.31856	0.99355
H	-1.19015	1.32239	1.73737
C	-0.16743	4.11396	0.04043
H	1.19752	4.10585	-1.65295
H	-1.45358	3.78612	1.75500
H	-0.28591	5.20224	0.05131

^{Li}**TS(C-D)**

SCF (BP86) Energy = -747.119456435
Enthalpy 0K = -746.798530
Enthalpy 298K = -746.775812
Free Energy 298K = -746.848609
Lowest Frequency = -389.5431 cm^{-1}
Second Frequency = 30.7507 cm^{-1}
SCF (BP86-D3BJ) Energy = -747.214547242
SCF (Bnz) Energy = -747.135417901
SCF (BS2) Energy = -1032.93297345

Si	2.37634	-0.87555	0.02724
C	0.65681	-0.25518	-1.66148
H	0.99003	0.58562	-2.28023
H	0.38385	-1.14829	-2.23224
C	3.34384	-1.01615	1.71554
H	3.54382	-0.02807	2.17742
H	4.33028	-1.48976	1.55484
H	2.79866	-1.62398	2.45857
C	3.63309	0.16871	-0.99088
H	4.66894	-0.06816	-0.68505
H	3.49265	1.25319	-0.82332
H	3.52683	-0.01102	-2.07183
C	2.23864	-2.69515	-0.54184
H	1.25520	-3.10473	-0.25514
H	3.01680	-3.32253	-0.07142
H	2.32574	-2.77967	-1.63672
Li	0.85640	1.44877	1.59030
O	0.96430	-0.14882	0.82530
C	-0.13502	0.03781	-0.48501
C	-1.26127	-0.94859	-0.19043
C	-2.22097	-1.18848	-1.19962
C	-1.41049	-1.61060	1.04521
C	-3.29941	-2.05824	-0.97840
H	-2.10511	-0.68648	-2.16599
C	-2.48465	-2.49022	1.26377
H	-0.65273	-1.44742	1.81576
C	-3.43520	-2.71491	0.25617
H	-4.03021	-2.23033	-1.77625
H	-2.57312	-3.00676	2.22605
H	-4.27115	-3.40162	0.42738
C	-0.52156	1.49437	-0.25655
C	-1.78866	1.88585	0.24498
C	0.44814	2.52495	-0.44518
C	-2.07961	3.23260	0.52532
H	-2.55263	1.12053	0.40877
C	0.15546	3.86968	-0.15595
H	1.43204	2.25700	-0.84732
C	-1.11235	4.23377	0.33473
H	-3.07235	3.49746	0.90543
H	0.92050	4.63567	-0.32582
H	-1.34098	5.27995	0.56019

^{Li}**D**

SCF (BP86) Energy = -747.207917914
Enthalpy 0K = -746.885387
Enthalpy 298K = -746.861137
Free Energy 298K = -746.940805
Lowest Frequency = 12.7288 cm^{-1}
Second Frequency = 16.5089 cm^{-1}
SCF (BP86-D3BJ) Energy = -747.288803790
SCF (Bnz) Energy = -747.216929436
SCF (BS2) Energy = -1033.02340204

Si	3.25821	0.64690	0.18774
C	-3.20800	-0.84051	1.88676
H	-3.32263	-1.86348	2.25824
H	-3.77846	-0.05876	2.39587
C	4.68464	-0.04559	-0.88847
H	4.88832	-1.10340	-0.64354
H	5.62364	0.51794	-0.74143
H	4.42689	0.00591	-1.96113
C	3.83048	0.58046	2.01413
H	4.76373	1.15010	2.17334
H	4.01520	-0.46119	2.33147
H	3.06054	1.00186	2.68394
C	3.03981	2.49255	-0.27972
H	2.73690	2.59533	-1.33692
H	3.97322	3.06725	-0.14195
H	2.26155	2.96672	0.34423
Li	0.86815	-1.53388	-0.29808
O	1.85801	-0.19714	-0.04344
C	-2.40869	-0.54622	0.83024
C	-2.33782	0.83282	0.26292
C	-3.52030	1.59458	0.11354
C	-1.09826	1.40989	-0.10726
C	-3.47043	2.90510	-0.38128
H	-4.48383	1.13971	0.36810
C	-1.05795	2.72380	-0.59946
H	-0.14873	0.86242	0.01320
C	-2.23697	3.47430	-0.73887
H	-4.39681	3.47764	-0.49791
H	-0.09001	3.15948	-0.86803
H	-2.19633	4.49691	-1.12895
C	-1.61020	-1.62828	0.18319
C	-1.44035	-1.66248	-1.22547
C	-0.99889	-2.65311	0.95111
C	-0.70988	-2.69370	-1.84310
H	-1.87798	-0.86400	-1.83128
C	-0.26564	-3.68389	0.33499
H	-1.07138	-2.61227	2.04202
C	-0.11719	-3.71066	-1.06671
H	-0.59029	-2.69501	-2.93096
H	0.21136	-4.45227	0.95150
H	0.46544	-4.50319	-1.54570

No metal system

a
SCF (BP86) Energy = -739.585592153
Enthalpy 0K = -739.270807
Enthalpy 298K = -739.247010
Free Energy 298K = -739.326266
Lowest Frequency = 13.8855 cm^{-1}
Second Frequency = 17.4423 cm^{-1}
SCF (BP86-D3BJ) Energy = -739.661715885
SCF (Bnz) Energy = -739.622192802
SCF (BS2) Energy = -1025.41544738

```
Si  3.53567 -0.09185  0.14432
C   2.14541 -0.55922 -0.94164
H   2.00937 -0.00818 -1.88699
H   1.93020 -1.63171 -1.05739
C   5.33843 -0.58182 -0.41478
H   5.42472 -1.67625 -0.54401
H   6.11109 -0.26667  0.31412
H   5.58413 -0.11866 -1.38796
C   3.36420 -0.87808  1.89442
H   4.23095 -0.64947  2.54199
H   3.27807 -1.97771  1.82740
H   2.45243 -0.50786  2.39328
C   3.63980  1.81714  0.39362
H   3.72603  2.33901 -0.57742
H   4.51452  2.10935  1.00368
H   2.73141  2.19338  0.89434
O  -3.80143  0.07064  1.04707
C  -2.71956  0.04496  0.40640
C  -2.13548 -1.27758  0.02759
C  -1.14649 -1.49930 -0.96788
C  -2.68557 -2.41423  0.68353
C  -0.74219 -2.80749 -1.28829
H  -0.71098 -0.66878 -1.52327
C  -2.27154 -3.70747  0.36932
H  -3.45343 -2.22614  1.44055
C  -1.29272 -3.91348 -0.62855
H   0.02323 -2.95000 -2.05869
H  -2.70580 -4.56495  0.89804
H  -0.96505 -4.92859 -0.88183
C  -2.06574  1.35279  0.05690
C  -0.69939  1.52023 -0.28040
C  -2.88574  2.50775  0.15155
C  -0.19522  2.80401 -0.54791
H   0.02148  0.68328 -0.30360
C  -2.38088  3.77838 -0.13828
H  -3.92340  2.36126  0.46782
C  -1.02859  3.93344 -0.50102
H   0.86890  2.90701 -0.78935
H  -3.03810  4.65489 -0.07749
H  -0.63003  4.92747 -0.73774
```

TS(a-b)
SCF (BP86) Energy = -739.579021692
Enthalpy 0K = -739.265132
Enthalpy 298K = -739.242005
Free Energy 298K = -739.319959
Lowest Frequency = -29.6125 cm^{-1}
Second Frequency = 7.8641 cm^{-1}
SCF (BP86-D3BJ) Energy = -739.654887025
SCF (Bnz) Energy = -739.617182790
SCF (BS2) Energy = -1025.41076340

```
Si -3.45005 -0.21122  0.12564
C  -1.78409  0.50034 -0.06899
H  -1.47667  0.87179 -1.05931
H  -1.37719  1.12473  0.73991
C  -4.99060  0.98483  0.09439
H  -4.91342  1.73998  0.89789
H  -5.95176  0.44898  0.22547
H  -5.03813  1.53396 -0.86382
C  -3.63317 -1.14646  1.80238
H  -4.65599 -1.53924  1.95333
H  -3.40329 -0.47924  2.65278
H  -2.92747 -1.99347  1.85099
C  -3.84315 -1.48960 -1.26701
H  -3.72715 -1.03417 -2.26749
H  -4.87608 -1.87839 -1.19669
H  -3.15076 -2.34767 -1.21292
O   2.42779  0.27602  2.18352
C   2.00531  0.20041  1.00657
C   1.67235  1.44069  0.24441
C   1.56937  1.49099 -1.16824
C   1.59109  2.66014  0.96664
C   1.38272  2.71053 -1.82908
H   1.64131  0.56286 -1.74339
C   1.39646  3.87583  0.30344
H   1.69760  2.60926  2.05459
C   1.29699  3.91363 -1.09986
H   1.30696  2.72701 -2.92279
H   1.32610  4.80570  0.88130
H   1.14800  4.86667 -1.62021
C   1.98955 -1.14144  0.31933
C   0.88535 -1.62549 -0.42189
C   3.12153 -1.97186  0.48732
C   0.94260 -2.90256 -1.00226
H  -0.04088 -1.01127 -0.45539
C   3.16954 -3.24709 -0.09349
H   3.95860 -1.59496  1.08547
C   2.07831 -3.71789 -0.84427
H   0.07626 -3.27318 -1.56324
H   4.05652 -3.87782  0.04432
H   2.10492 -4.72250 -1.28425
```

b
SCF (BP86) Energy = -739.604925854
Enthalpy 0K = -739.287033
Enthalpy 298K = -739.265028
Free Energy 298K = -739.337628
Lowest Frequency = 13.0159 cm^{-1}
Second Frequency = 34.8344 cm^{-1}
SCF (BP86-D3BJ) Energy = -739.691050447
SCF (Bnz) Energy = -739.645594354
SCF (BS2) Energy = -1025.43570116

```
Si  0.40846 -2.49535  0.03092
C   0.20724 -1.04547  1.26350
H  -0.57376 -1.35978  1.98559
H   1.14327 -1.04328  1.85648
C   1.53211 -3.81749  0.86980
H   2.54010 -3.40858  1.06116
H   1.64847 -4.72161  0.24370
H   1.11444 -4.13257  1.84247
C   1.26429 -2.09588 -1.63752
H   1.50164 -3.03127 -2.17712
```

H   2.20243 -1.54031 -1.47370
H   0.63725 -1.47476 -2.29865
C -1.24997 -3.38919 -0.34574
H -1.68059 -3.80053 0.58463
H -1.10813 -4.22892 -1.05051
H -1.99558 -2.69934 -0.77365
O -0.09870 1.09712 2.31428
C -0.07996 0.55681 1.09956
C -1.44486 0.76229 0.34253
C -1.85783 0.14704 -0.85733
C -2.34998 1.63763 0.97451
C -3.12882 0.39834 -1.40937
H -1.18703 -0.54728 -1.37613
C -3.61541 1.90109 0.42941
H -1.97601 2.06043 1.91709
C -4.01508 1.27977 -0.76808
H -3.42724 -0.09891 -2.34190
H -4.30181 2.59046 0.94003
H -5.00740 1.47450 -1.19471
C   1.11201 1.15892 0.24099
C   1.16657 1.35623 -1.15404
C   2.20696 1.61450 1.00695
C   2.28085 1.96871 -1.76206
H   0.32145 1.05544 -1.78211
C   3.33366 2.19338 0.40783
H   2.08076 1.51907 2.09305
C   3.37645 2.37963 -0.98777
H   2.28637 2.12982 -2.84854
H   4.17785 2.52350 1.02858
H   4.24524 2.85401 -1.46191

TS(b-c)1
SCF (BP86) Energy = -739.603637809
Enthalpy 0K = -739.285804
Enthalpy 298K = -739.264665
Free Energy 298K = -739.334491
Lowest Frequency = -31.6358 cm^{-1}
Second Frequency = 22.6438 cm^{-1}
SCF (BP86-D3BJ) Energy = -739.689625967
SCF (Bnz) Energy = -739.644234765
SCF (BS2) Energy = -1025.43490839

Si -1.62804 1.95680 0.02953
C -0.50534 0.98807 1.25258
H   0.17246 1.72181 1.73161
H -1.19970 0.67950 2.05762
C -3.45188 1.76753 0.60521
H -3.74704 0.70398 0.57747
H -4.15441 2.33244 -0.03422
H -3.57963 2.11856 1.64452
C -1.64634 1.49489 -1.83198
H -2.41080 2.10465 -2.34841
H -1.89392 0.43069 -1.97308
H -0.67994 1.67951 -2.33132
C -1.19873 3.83236 0.10225
H -1.28089 4.21762 1.13401
H -1.86445 4.43775 -0.54020
H -0.15971 4.00529 -0.23004
O   0.63637 -0.85144 2.32669
C   0.39412 -0.36919 1.10983
C   1.73137 -0.01847 0.35986
C   1.90230 0.93013 -0.66846
C   2.87919 -0.68602 0.83473
C   3.17523 1.20136 -1.20871
H   1.03494 1.47920 -1.05105
C   4.14591 -0.44103 0.28746
H   2.69586 -1.36592 1.67662
C   4.30341 0.51169 -0.73816
H   3.28381 1.95883 -1.99651
H   5.02329 -0.98193 0.66744
H   5.29598 0.72216 -1.15663
C -0.42743 -1.40888 0.24452
C -0.35401 -1.59686 -1.15128
C -1.24159 -2.29112 0.98494
C -1.08466 -2.62049 -1.78715
H   0.29965 -0.95204 -1.74984
C -2.00127 -3.28760 0.35616
H -1.19199 -2.16386 2.07413
C -1.92263 -3.46299 -1.03943
H -0.99132 -2.76357 -2.87218
H -2.64272 -3.95000 0.95345
H -2.49379 -4.25869 -1.53457

INT(b-c)
SCF (BP86) Energy = -739.606258083
Enthalpy 0K = -739.288429
Enthalpy 298K = -739.266475
Free Energy 298K = -739.338091
Lowest Frequency = 32.9789 cm^{-1}
Second Frequency = 41.8834 cm^{-1}
SCF (BP86-D3BJ) Energy = -739.693677343
SCF (Bnz) Energy = -739.647125587
SCF (BS2) Energy = -1025.43755533

Si -1.85942 -1.71999 -0.04367
C -0.46021 -1.10636 -1.19022
H   0.20811 -1.96088 -1.41747
H -0.99689 -0.90673 -2.13966
C -3.32565 -0.49226 0.01648
H -3.02796 0.45481 0.49467
H -4.17894 -0.91667 0.57675
H -3.67412 -0.25325 -1.00338
C -1.41098 -2.16411 1.77059
H -2.30687 -2.53981 2.29841
H -1.03973 -1.28589 2.32393
H -0.64004 -2.95299 1.82229
C -2.54536 -3.35189 -0.80894
H -2.86967 -3.18979 -1.85213
H -3.41258 -3.74124 -0.24369
H -1.77233 -4.14080 -0.82384
O   0.72966 0.67464 -2.33871
C   0.48048 0.23065 -1.10980
C   1.81200 -0.13785 -0.35283
C   1.95450 -1.04973 0.71277
C   2.97745 0.47691 -0.85397
C   3.21935 -1.33310 1.26524
H   1.07236 -1.56134 1.11223
C   4.23691 0.21740 -0.29666
H   2.81205 1.12881 -1.72155
C   4.36693 -0.69585 0.76790
H   3.30694 -2.06024 2.08381
H   5.12966 0.71643 -0.69780
H   5.35309 -0.91677 1.19613
C -0.28527 1.31708 -0.24343
C -0.33750 1.39717 1.16378
C -0.92739 2.32603 -0.98720
C -1.03238 2.43987 1.80646
H   0.19497 0.65439 1.76976

C -1.64460 3.35313 -0.35612
H -0.78887 2.24476 -2.07413
C -1.70039 3.41736 1.04931
H -1.04461 2.49386 2.90343
H -2.15314 4.12024 -0.95632
H -2.24396 4.22915 1.54945

TS(b-c)2
SCF (BP86) Energy = -739.605364894
Enthalpy 0K = -739.287792
Enthalpy 298K = -739.266660
Free Energy 298K = -739.335923
Lowest Frequency = -41.4677 cm^{-1}
Second Frequency = 32.3593 cm^{-1}
SCF (BP86-D3BJ) Energy = -739.692288141
SCF (Bnz) Energy = -739.645942302
SCF (BS2) Energy = -1025.43653211

Si -2.13378 -1.50796 0.01543
C -0.45784 -1.35326 -0.87548
H   0.15477 -2.21368 -0.53457
H -0.67793 -1.56086 -1.93954
C -3.40983 -0.17792 -0.49126
H -3.08002 0.82559 -0.17874
H -4.39795 -0.38303 -0.04004
H -3.53322 -0.15862 -1.58805
C -2.03023 -1.54403 1.93113
H -3.01166 -1.79823 2.37165
H -1.72181 -0.56354 2.32785
H -1.30220 -2.29864 2.27893
C -2.88620 -3.20832 -0.49599
H -3.01505 -3.26701 -1.59134
H -3.87540 -3.37664 -0.03112
H -2.22831 -4.04369 -0.19677
O   0.64433 0.28387 -2.31948
C   0.48456 -0.01826 -1.03533
C   1.86433 -0.32963 -0.32631
C   2.05497 -1.05865 0.86622
C   3.00686 0.14463 -0.99919
C   3.34619 -1.29803 1.37454
H   1.18773 -1.46325 1.40411
C   4.29559 -0.07620 -0.49337
H   2.79410 0.65826 -1.94651
C   4.47397 -0.80315 0.69935
H   3.47144 -1.87989 2.29770
H   5.17249 0.30872 -1.03190
H   5.48123 -0.99174 1.09234
C -0.16067 1.20002 -0.23684
C -0.18091 1.39441 1.15994
C -0.70667 2.21163 -1.05037
C -0.75569 2.54902 1.72566
H   0.28147 0.65200 1.82064
C -1.29887 3.35645 -0.49563
H -0.60107 2.02310 -2.12809
C -1.32742 3.53250 0.90074
H -0.74720 2.68550 2.81547
H -1.73080 4.12620 -1.15009
H -1.77524 4.43261 1.34109

c
SCF (BP86) Energy = -739.627173943
Enthalpy 0K = -739.309014
Enthalpy 298K = -739.287221
Free Energy 298K = -739.359357
Lowest Frequency = 18.4822 cm^{-1}
Second Frequency = 23.7662 cm^{-1}
SCF (BP86-D3BJ) Energy = -739.711548279
SCF (Bnz) Energy = -739.664734219
SCF (BS2) Energy = -1025.45206177

Si 2.60128 0.00277 -0.08147
C   1.02644 0.00178 -1.23783
H   0.95876 0.89448 -1.88738
H   0.96020 -0.89022 -1.88848
C   3.98341 0.00368 -1.51606
H   3.89858 -0.88514 -2.17353
H   5.01472 0.00446 -1.10864
H   3.89731 0.89224 -2.17372
C   3.15269 -1.61648 0.81419
H   4.24693 -1.77303 0.76933
H   2.67408 -2.48931 0.33293
H   2.82448 -1.60942 1.86618
C   3.15084 1.62255 0.81437
H   2.67191 2.49502 0.33277
H   4.24500 1.77986 0.77010
H   2.82205 1.61530 1.86617
O   0.86375 0.00167 1.07540
C   0.02771 0.00050 -0.04177
C -0.90409 -1.25001 -0.04023
C -1.60376 -1.71250 -1.17418
C -1.04875 -1.96340 1.16530
C -2.42678 -2.84924 -1.10462
H -1.49318 -1.18563 -2.12936
C -1.87464 -3.09536 1.24339
H -0.46187 -1.59531 2.01399
C -2.57002 -3.54577 0.10753
H -2.95334 -3.19718 -2.00263
H -1.97272 -3.63615 2.19368
H -3.20998 -4.43499 0.16263
C -0.90747 1.24845 -0.04028
C -1.60866 1.70872 -1.17421
C -1.05374 1.96182 1.16506
C -2.43471 2.84327 -1.10479
H -1.49689 1.18185 -2.12925
C -1.88264 3.09159 1.24301
H -0.46567 1.59556 2.01372
C -2.57951 3.53979 0.10718
H -2.96243 3.18953 -2.00277
H -1.98192 3.63241 2.19315
H -3.22183 4.42731 0.16217

TS(c-d)
SCF (BP86) Energy = -739.602455130
Enthalpy 0K = -739.286042
Enthalpy 298K = -739.264184
Free Energy 298K = -739.336038
Lowest Frequency = -321.8445 cm^{-1}
Second Frequency = 24.8714 cm^{-1}
SCF (BP86-D3BJ) Energy = -739.687290891
SCF (Bnz) Energy = -739.639715833
SCF (BS2) Energy = -1025.43072900

Si -2.62828 0.00118 0.03145
C -0.49008 0.00046 -1.81961
H -0.69200 -0.93914 -2.33865
H -0.69128 0.94028 -2.33852
C -3.24955 -1.56692 -0.89097

```
H  -3.12213 -1.46237 -1.97955
H  -4.31657 -1.76377 -0.66629
H  -2.66962 -2.44989 -0.57092
C  -3.24903  1.56937 -0.89116
H  -4.31669  1.76500 -0.66849
H  -3.11929  1.46576 -1.97954
H  -2.67065  2.45268 -0.56920
C  -3.67277  0.00166  1.66140
H  -3.45541 -0.88847  2.27845
H  -4.75859  0.00280  1.44357
H  -3.45363  0.89092  2.27906
O  -1.07380  0.00072  0.71044
C   0.05764  0.00016 -0.48930
C   0.88532  1.25921 -0.14045
C   1.69466  1.85333 -1.13213
C   0.91434  1.82055  1.15518
C   2.49361  2.97328 -0.84957
H   1.65746  1.41074 -2.13446
C   1.71745  2.93558  1.44405
H   0.26669  1.37666  1.91516
C   2.51291  3.52085  0.44378
H   3.10475  3.42058 -1.64422
H   1.71561  3.35738  2.45750
H   3.13701  4.39454  0.66966
C   0.88402 -1.25978 -0.14056
C   1.69359 -1.85391 -1.13206
C   0.91153 -1.82209  1.15467
C   2.49132 -2.97477 -0.84970
H   1.65748 -1.41063 -2.13412
C   1.71336 -2.93810  1.44333
H   0.26373 -1.37810  1.91448
C   2.50908 -3.52334  0.44326
H   3.10270 -3.42202 -1.64419
H   1.71031 -3.36067  2.45646
H   3.13217 -4.39780  0.66896
```

d

SCF (BP86) Energy = -739.650935432
Enthalpy 0K = -739.333373
Enthalpy 298K = -739.310297
Free Energy 298K = -739.388672
Lowest Frequency = 10.0428 cm^{-1}
Second Frequency = 13.6957 cm^{-1}
SCF (BP86-D3BJ) Energy = -739.725952697
SCF (Bnz) Energy = -739.690834588
SCF (BS2) Energy = -1025.48356776

```
Si -3.42124 -0.00183  0.13293
C   2.76266  0.00182  2.17320
H   2.98790  0.93826  2.69446
H   2.98965 -0.93420  2.69443
C  -4.00800  1.51936  1.17766
H  -3.46713  1.56007  2.14065
H  -5.09173  1.47855  1.40095
H  -3.80894  2.46913  0.64862
C  -4.00938 -1.52193  1.17848
H  -5.09303 -1.47996  1.40197
H  -3.46836 -1.56276  2.14138
H  -3.81138 -2.47213  0.64980
C  -4.59501 -0.00166 -1.40775
H  -4.41274  0.88828 -2.03749
H  -5.66527 -0.00104 -1.12400
H  -4.41365 -0.89206 -2.03708
O  -1.84490 -0.00275 -0.23912
C   2.17728  0.00126  0.95020
C   1.91573 -1.30430  0.25610
C   2.99136 -2.21884  0.14387
C   0.65383 -1.66348 -0.27547
C   2.82388 -3.46530 -0.47757
H   3.97311 -1.92911  0.53664
C   0.49957 -2.91740 -0.89187
H  -0.24470 -1.01489 -0.19057
C   1.56995 -3.81990 -1.00137
H   3.67483 -4.15277 -0.55899
H  -0.48838 -3.18141 -1.28641
H   1.43111 -4.79133 -1.49178
C   1.91337  1.30623  0.25591
C   2.98687  2.22342  0.14497
C   0.65122  1.66233 -0.27719
C   2.81705  3.46951 -0.47658
H   3.96889  1.93607  0.53879
C   0.49460  2.91591 -0.89366
H  -0.24586  1.01159 -0.19329
C   1.56288  3.82106 -1.00183
H   3.66642  4.15905 -0.55700
H  -0.49354  3.17755 -1.28930
H   1.42221  4.79218 -1.49234
```

2.2 Li–C and Na–C Bonding and Topological Analyses

A series of chemical bonding analyses were explored by PBE/Def2-TZVPP calculations with the ADF program[29], including energy decomposition analyses (EDA)[30] and natural localized molecular orbitals (NLMOs)[31]. Mayer and Wiberg bond orders and topological analysis of electron density have been investigated by employing quantum theory of atoms in molecules (QTAIM) with Multiwfn software[32].

Table S7. The Mayer bond and WBIs of Li–C and Na–C bonds in complex **1**-Na and **1**-Li at the PBE/Def2-TZVPP level of theory.

Mayer Bond order	Na (2)-C (49)	0.259
	Li (38)-C (23)	0.442
Wiberg Bond order	Na (2)-C (49)	0.493
	Li (38)-C (23)	0.533

Table S8. NLMO analyses for **1**-Na and **1**-Li complexes at the PBE/ Def2-TZVPP level of theory.

	Species	NLMO
**1**-Na	$LP_{C(49)}$	89.60%C($sp^{5.75}$) + 4.24% Si($sp^{6.00}d^{4.16}f^{0.21}$) +3.41% Na($sp^{0.02}d^{0.03}$)
**1**-Li	$LP_{C(23)}$	90.92%C($sp^{4.77}$) +3.24% Si($sp^{8.14}d^{3.78}f^{0.19}$) +3.51% Li($sp^{0.05}d^{0.04}$)

Table S9. Calculated Topological Parameters in the Na–C and Li–C Bond Critical Points (BCPs) (a. u.) for M–C bond at the PBE/ Def2-TZVPP level of theory.

Complex	Bond	ρ	$\nabla^2\rho$	G(r)	V(r)	H(r)
**1**-Na	Na–C	0.023	0.090	0.0199	-0.0173	0.00255
**1**-Li	Li–C	0.307	0.129	0.0308	-0.0293	0.00149

The values of electron density at BCP greater than 0.20 au and $\nabla^2\rho$ less than 0 describe a typical covalent bond. ρ less than 0.10 au and accompanying positive $\nabla^2\rho$ reflect an ionic bond.

REFERENCES

1 Davison, N., Waddell, P.G., Dixon, C., Wills, C., Penfold, T.J., and Lu, E. (2022). A monomeric (trimethylsilyl)methyl lithium complex: synthesis, structure, decomposition

and preliminary reactivity studies. Dalton Transactions 51, 10707-10713. 10.1039/D1DT03532K.

2 Baillie, S.E., Clegg, W., García-Álvarez, P., Hevia, E., Kennedy, A.R., Klett, J., and Russo, L. (2011). Synthesis and characterization of an infinite sheet of metal–alkyl bonds: unfolding the elusive structure of an unsolvated alkali-metal trisalkylmagnesiate. Chemical Communications 47, 388-390. 10.1039/C0CC02164D.

3 Lynch, J.R., Kennedy, A.R., Barker, J., Reid, J., and Mulvey, R.E. (2022). Crystallographic Characterisation of Organolithium and Organomagnesium Intermediates in Reactions of Aldehydes and Ketones. Helv. Chim. Acta 105, e202200082. 10.1002/hlca.202200082

4 Anderson, D. E., Tortajada, A. and Hevia, E. (2023). Highly Reactive Hydrocarbon Soluble Alkylsodium Reagents for Benzylic Aroylation of Toluenes using Weinreb Amides. Angew. Chem. Int. Ed. 10.1002/anie.202218498

5 Aversa-Fleener, C.R., Chang, D.K., and Liberman-Martin, A.L. (2021). Carbodiphosphorane-Catalyzed Hydroboration of Ketones and Imines. Organometallics 40, 4050-4054. 10.1021/acs.organomet.1c00628.

6 Young, P.C., Hadfield, M.S., Arrowsmith, L., Macleod, K.M., Mudd, R.J., Jordan-Hore, J.A., and Lee, A.-L. (2012). Divergent Outcomes of Gold(I)-Catalyzed Indole Additions to 3,3-Disubstituted Cyclopropenes. Organic Letters 14, 898-901. 10.1021/ol203418u.

7 Colas, K., Martín-Montero, R., and Mendoza, A. (2017). Intermolecular Pummerer Coupling with Carbon Nucleophiles in Non-Electrophilic Media. Angewandte Chemie International Edition 56, 16042-16046. 10.1002/anie.201709715.

8 Stec, J., Thomas, E., Dixon, S., and Whitby, R.J. (2011). Tandem Insertion of Halocarbenoids and Lithium Acetylides into Zirconacycles: A Novel Rearrangement to Zirconium Alkenylidenates by β-Addition to an Alkynyl Zirconocene. Chemistry – A European Journal *17*, 4896-4904. 10.1002/chem.201002962.

9 Spangler, B., Fontaine, S.D., Shi, Y., Sambucetti, L., Mattis, A.N., Hann, B., Wells, J.A., and Renslo, A.R. (2016). A Novel Tumor-Activated Prodrug Strategy Targeting Ferrous Iron Is Effective in Multiple Preclinical Cancer Models. Journal of Medicinal Chemistry 59, 11161-11170. 10.1021/acs.jmedchem.6b01470.

10 Harthun, A., Giernoth, R., Elsevier, C.J., and Bargon, J. (1996). Rhodium- and palladium-catalysed proton exchange in styrene detected in situ by para-hydrogen induced polarization. Chemical Communications, 2483-2484. 10.1039/CC9960002483.

11 Camedda, N., Serafino, A., Maggi, R., Bigi, F., Cera, G., and Maestri, G. (2020). Functionalization of Alkenyl C–H Bonds with D2O via Pd(0)/Carboxylic Acid Catalysis. Synthesis 52, 1762-1772. 10.1055/s-0039-1690892.

12 Schäfer, M.; Stünkel, T.; Daniliuc, C. G.; Gilmour, R., Angew. Chem. Int. Ed. 2022, 61, e202205508.

13 Boutland, A. J.; Carroll, A.; Alvarez Lamsfus, C.; Stasch, A.; Maron, L.; Jones, C., J. Am. Chem. Soc. 2017, 139, 18190–18193.

14 Wu, L.; Wang, Y.; Wang, Y.; Shen, K.; Li, Y., Polymer, 2013, 54, 2958-2965.

15 Ahlbrecht, H.; Raab, W.; Vonderheid, C. Synthesis 1979, 1979 (02), 127-129.

16 Venkat Reddy, C. R.; Urgaonkar, S.; Verkade, J. G. Org. Lett. 2005, 7 (20), 4427-4430.

17 Hevia, E.; Kennedy, A. R.; Klett, J.; McCall, M. D. Chem. Commun. 2009, (22), 3240-3242.

18 Neufeld, R.; Stalke, D. Chem. Sci. 2015, 6 (6), 3354-3364.

19 Bachmann, S.; Neufeld, R.; Dzemski, M.; Stalke, D. Chem. Eur. J. 2016, 22 (25), 8462-8465.

20 Gaussian 16 Rev. C.01, M. J. Frisch, G. W. Trucks, H. B. Schlegel, G. E. Scuseria, M. A. Robb, J. R. Cheeseman, G. Scalmani, V. Barone, G. A. Petersson, H. Nakatsuji, X. Li, M. Caricato, A. V. Marenich, J. Bloino, B. G. Janesko, R. Gomperts, B. Mennucci, H. P. Hratchian, J. V. Ortiz, A. F. Izmaylov, J. L. Sonnenberg, Williams, F. Ding, F. Lipparini, F. Egidi, J. Goings, B. Peng, A. Petrone, T. Henderson, D. Ranasinghe, V. G. Zakrzewski, J. Gao, N. Rega, G. Zheng, W. Liang, M. Hada, M. Ehara, K. Toyota, R. Fukuda, J. Hasegawa, M. Ishida, T. Nakajima, Y. Honda, O. Kitao, H. Nakai, T. Vreven, K. Throssell, J. A. Montgomery Jr., J. E. Peralta, F. Ogliaro, M. J. Bearpark, J. J. Heyd, E. N. Brothers, K. N. Kudin, V. N. Staroverov, T. A. Keith, R. Kobayashi, J. Normand, K. Raghavachari, A. P. Rendell, J. C. Burant, S. S. Iyengar, J. Tomasi, M. Cossi, J. M. Millam, M. Klene, C. Adamo, R. Cammi, J. W. Ochterski, R. L. Martin, K. Morokuma, O. Farkas, J. B. Foresman and D. J. Fox, Wallingford, CT (2016)

21 Andrae, D., Häußermann, U., Dolg, M., Stoll, H., and Preuß, H. (1990). Energy-adjustedab initio pseudopotentials for the second and third row transition elements. Theoretica Chimica Acta 77, 123-141. 10.1007/BF01114537.

22 Hariharan, P.C., and Pople, J.A. (1973). The influence of polarization functions on molecular orbital hydrogenation energies. Theoretica Chimica Acta 28, 213-222. 10.1007/BF00533485.

23 Hehre, W.J., Ditchfield, R., and Pople, J.A. (1972). Self—Consistent Molecular Orbital Methods. XII. Further Extensions of Gaussian—Type Basis Sets for Use in Molecular Orbital Studies of Organic Molecules. The Journal of Chemical Physics 56, 2257-2261. 10.1063/1.1677527.

24 Becke, A.D. (1988). Density-functional exchange-energy approximation with correct asymptotic behavior. Physical Review A 38, 3098-3100. 10.1103/PhysRevA.38.3098.

25 Perdew, J.P. (1986). Density-functional approximation for the correlation energy of the inhomogeneous electron gas. Physical Review B 33, 8822-8824. 10.1103/PhysRevB.33.8822.

26 Tomasi, J., Mennucci, B., and Cammi, R. (2005). Quantum Mechanical Continuum Solvation Models. Chemical Reviews 105, 2999-3094. 10.1021/cr9904009.

27 Grimme, S., Ehrlich, S., and Goerigk, L. (2011). Effect of the damping function in dispersion corrected density functional theory. Journal of Computational Chemistry 32, 1456-1465. 10.1002/jcc.21759.

28 NBO 7.0. E. D. Glendening, J, K. Badenhoop, A. E. Reed, J. E. Carpenter, J. A. Bohmann, C. M. Morales, P. Karafiloglou, C. R. Landis, and F. Weinhold, Theoretical Chemistry Institute, University of Wisconsin, Madison, 2018.

29 Baerends, E.J., Ellis, D.E., and Ros, P. (1973). Self-consistent molecular Hartree—Fock—Slater calculations I. The computational procedure. Chem. Phys. 2, 41-51. 10.1016/0301-0104(73)80059-X

30 Ziegler, T., and Rauk, A. (1977). On the calculation of bonding energies by the Hartree Fock Slater method. Theo. Chem. Acta (Berl.) 46, 1-10.

31 Glendening, E.D., and Weinhold, F. (1998). Natural resonance theory: II. Natural bond order and valency. J. Compu. Chem. 19, 610-627. 10.1002/(SICI)1096-987X(19980430)19:6<610::AID-JCC4>3.0.CO;2-U

32 Lu, T., and Chen, F. (2012). Multiwfn: A multifunctional wavefunction analyzer. J. Compu. Chem. 33, 580-592. 10.1002/jcc.22885.
